# Understanding who volunteers globally through an examination of demographic variation in volunteering across 22 countries

**DOI:** 10.1038/s41598-025-05459-2

**Published:** 2025-07-13

**Authors:** Julia S. Nakamura, Cristina B. Gibson, Robert D. Woodberry, Matthew T. Lee, Young-Il Kim, Koichiro Shiba, R. Noah Padgett, Byron R. Johnson, Tyler J. VanderWeele

**Affiliations:** 1 Department of Psychology, University of British Columbia, 2136 West Mall, Vancouverr, V6T 1Z4 BC Canada; 2 Graziadio Business School, Pepperdine University, Malibu, USA; 3 Institute for Studies of Religion, Baylor University, Waco, USA; 4 Human Flourishing Program, Institute for Quantitative Social Science, Harvard University, Cambridge, USA; 5 College of Social Work, George Fox University, Newberg, USA; 6 School of Public Health, Boston University, Boston, USA; 7 Department of Epidemiology, Harvard T.H. Chan School of Public Health, Boston, USA; 8 Department of Biostatistics, Harvard T.H. Chan School of Public Health, Boston, USA

**Keywords:** Global Flourishing Study, Volunteering, Demographics, Cross-cultural, Flourishing, Prosocial behavior, International comparison, Public health, Human behaviour

## Abstract

Volunteering has been associated with enhanced individual and societal well-being around the world. While some prior research has assessed cultural and sociodemographic differences in volunteering, we know little about how volunteering differs across sociodemographic indicators cross-nationally. Using data from the Global Flourishing Study, a diverse and international sample of 202,898 individuals across 22 countries, we examined the distribution of volunteering across demographic factors (age, gender, marital status, employment status, religious service attendance, education, and immigration status) and across countries. We found variation in volunteering across demographic groups and countries. Unadjusted proportions of volunteering varied between countries: Nigeria showed the highest proportion (0.51) followed by Indonesia (0.46) and Kenya (0.40), while Japan (0.09), Poland (0.08), and Egypt (0.04) showed the lowest proportions of volunteering. Random effects meta-analyses showed that the proportion of people who volunteered differed between demographic groups (e.g., volunteering was higher among those with more education and religious service attendance). Because of the growing evidence of substantial contributions of volunteering to individual as well as societal well-being, it is increasingly important for organizations, governments, and public health officials alike to consider ways to ensure accessibility for able and willing volunteers.

**Supplementary Information:**

The online version contains supplementary material available at 10.1038/s41598-025-05459-2.

## Introduction

Volunteering is a popular pathway for people to serve their communities and promote desired social changes. One recent estimate by the United Nations Volunteers program indicated that over 800 million people 15 years and older around the world engage in volunteering at least monthly^1^. This includes rebuilding communities after natural disasters, delivering meals to those in need, advocating for policy change, and much more. We focus on formal volunteering (throughout, referred to as volunteering), broadly defined as, “an activity undertaken by an individual that is uncoerced, unpaid, structured by an organization, and directed toward a community concern”^2^ (as opposed to informal volunteering, which refers to “unpaid volunteering not coordinated by an organization or institution”)^3^. Although there is substantial variation between countries, one estimate suggests that the worldwide monthly formal volunteering rate amounts to roughly 24% of the population^4^. Volunteering (giving time) is a major pathway for prosociality (voluntary acts intended to benefit others, cf^5^).

A robust body of research has demonstrated that volunteering is associated with improved societal and individual well-being. For example, volunteer work contributes billions to well-studied economies annually^6,7^. Studies have also found associations between volunteering and positive psychological, behavioral, and long-term social impact on communities. The benefits of volunteering to at-risk communities facing endemic social problems such as poverty, crime, and unemployment may be especially important^8^. Volunteering has also been associated with improvements to numerous health and well-being outcomes for volunteers (e.g., a reduced risk of all-cause mortality and improved physical health, health behaviors, and psychosocial well-being^9,10^). Indeed, physicians and policymakers are being encouraged to “prescribe” volunteering to those that are willing and able to volunteer as a way of enhancing individual and societal health^11,12^.

Given the important contributions of volunteering to societal and individual well-being, it is important to understand how participation in volunteering differs across nations and sociodemographic groups. It is of practical importance for organizations, governments, and public health officials to better understand who volunteers and who does not. Should certain demographic groups have lower rates of participation in volunteering, interventionists may consider barriers and facilitators to participation for these groups to ensure accessibility for able and willing volunteers. Advancing our knowledge of where volunteering is (or is not) common internationally may prove to be a catalyst for enhancing volunteerism around the world. Rigorous cross-national research is needed to inform interventions and aid decision-makers in developing policies that advance volunteerism globally.

Prior literature has documented individual differences in the likelihood and extent of volunteering and civic engagement^13–15^. These findings suggest that the relationship between demographic characteristics and rates of volunteering are rarely straightforward, as differences observed tend to be based on the types of volunteering and interactions between different sociodemographic factors (e.g., findings on gender differences are influenced by education, marital status, and other demographic factors). In general, rates of volunteering appear to have a curvilinear relationship with age (inverted U-shape^13,16^), though continuity seems to be the predominant pattern for volunteering in later life: people tend to maintain the habits they exhibited before retirement^13^. Findings on gender and volunteering are mixed, with some studies finding that women volunteer more than men, others finding that men volunteer more than women, and others finding no differences^13,14,17–19^. Most studies on race or ethnicity and volunteering are from United States (US) samples. There is evidence that racial and ethnic minority groups in the US volunteer less than Whites^13^. However, findings are often inconsistent across studies^18^, and it is likely that racial and ethnic differences disappear when accounting for other sociodemographic factors (e.g., education) and other factors (e.g., being invited to participate)^13,14^. In general, there are also mixed findings on whether marital status is associated with volunteering^18^. Most studies have found an increased likelihood of volunteering for those who are married^13,18^; however, this association may fade when accounting for other factors (e.g., church attendance and parental status^13^). Generally, higher socioeconomic status is associated with more volunteering. While employment may associated with increased volunteerism^13,14^, there is evidence that part-time workers may volunteer more than those working full-time^13–15^ and that individuals not in the labor force may volunteer more hours than those working full-time^13^. Immigrants may be less likely to volunteer than native-born individuals^13,20^, but this is likely due to initial unfamiliarity with how the process works or practical barriers (e.g., language)^13^ as immigrant volunteering tends to increase over time^20^. Education^13,14,18,21^ and religious service attendance have been repeatedly associated with increased volunteering, even when volunteering or religious organizations is excluded^18,22–24^. In summary, although demographics continue to be a subject of interest for those studying volunteering, these factors are often examined in isolation and findings tend to be mixed. Consequently, we lack an integrated understanding of demographic variation cross-nationally on this important and influential activity.

Prior research shows significant variation in volunteering rates across countries^25^. While some countries (e.g., Liberia, Indonesia, Kenya) report high rates of adult volunteering (e.g., over half the population), others report lower rates (e.g., less than 10% in Egypt, Poland, and Bulgaria)^4^. National differences may be due to compositional (differing volunteer rates simply because populations are composed of different people) or contextual (due to characteristics of a country) factors^13^. With regard to the latter, differences in volunteering rates can be explained by *structural* factors (e.g., historical context, economic development) and *cultural* factors (e.g., religiosity, trust) that tend to coincide with national context, which interact with and influence each other^24^.

Regarding structural factors, government types, state involvement in the economy, ethnic and racial heterogeneity, economic development, democracy, social inequality, size of a country’s non-profit sector, industrialization, and other structural factors may be associated with volunteering rates. As one example, a study examining 23 European countries found that as Gross Domestic Product increased, so did volunteering rates; they also found that countries with higher income inequality had less volunteering^26^. Likewise, culture may also be associated with volunteering. The logic is fairly straightforward. For example, when citizens place a high value on universalism ("understanding, appreciation, tolerance, and protection for the welfare of all people and for nature"^27^), they may be more likely to volunteer to improve the welfare of others or the environment. Many other cultural factors have been examined in prior research on volunteering, including cultural orientations such as uncertainty avoidance and future orientation (for a fuller discussion of structural and cultural influences on volunteering, see^28–30^). Because a variety of structural and cultural factors influence volunteering and may interact, it can be difficult to predict the general influence of national context on volunteering rates. Importantly, studies in a variety of countries have found similar motives and values involved in volunteering despite cultural differences^24^.

While existing studies have made meaningful contributions to our understanding of volunteering in adulthood cross-nationally, many previous studies include a limited number of demographic variables^21^, and comparisons across countries have often been hampered by inconsistencies in how these variables have been operationalized. The meaning and practice of volunteering can vary across cultural and institutional contexts based on the degree of formality, the types of organizations involved, or the extent to which activities are motivated by altruism versus obligation. Some groups might engage in relatively little formal volunteering with organizations, but instead provide a great deal of informal support for their families or neighbors. A group with low rates of volunteering would therefore not necessarily be lacking in prosociality if informal support is high. However, the coordinated nature of formal volunteering might fill gaps left by less coordinated informal support systems. Cross-national comparisons of volunteering are valuable for identifying broad patterns and trends of this vital form of support, while also highlighting the unique factors that may shape volunteering in specific countries.

Though many studies assess rates of volunteering globally^4,25^, few studies also look at variation across multiple sociodemographic factors simultaneously. The current study includes a broad array of demographic factors and consistent measurement across 22 nations. The selection of demographic variables was informed by a diverse team of survey researchers drawn from around the world, including experienced global survey experts affiliated with Gallup. Targeted feedback on the variables was solicited from scholars based in Brazil, New Zealand, Australia, South Africa, China, the United Kingdom (UK) and the US, as well as members of professional and academic societies within the behavioral and social sciences (cf^31^.). This extensive process was designed to ensure that the survey would include the most important demographic variables that are commonly used in cross-national comparisons across demographic factors in social research. The current study explores how rates of volunteering are ordered across a diverse set of countries, with nationally representative samples^31^, and how these rates vary across a more comprehensive set of demographic variables than has been utilized in previous research. Our approach not only provides insights into the generalizability of how sociodemographic factors relate to volunteering, but also reveals critical variations in how volunteering is embedded in different countries, contributing to a richer understanding of its role globally, thus enhancing its relevance for policy and practice.

Specifically, we examine the distribution of formal volunteering across countries and demographic groups, using data from a diverse and international sample of 202,898 individuals across 22 countries. First, we assess how the proportion of participants who volunteered in the past month varies across countries. Second, we assess how the proportion of participants who volunteered in the past month varies across different demographic factors, including age, gender, marital status, employment status, religious service attendance, education, immigration status, and where available, race and ethnicity, and religious affiliation. We report the distributions of volunteering in each country and globally via meta-analysis. We hypothesize that volunteering will exhibit variations across different demographic categories, and that these differences across demographic categories will themselves vary by country.

## Methods

The description of the methods below have been adapted from other materials^32^. Further methodological detail is available elsewhere^31,33–39^.

### Study population

The Global Flourishing Study (GFS) is a study of 202,898 participants from 22 geographically and culturally diverse countries, with nationally representative sampling within each country, concerning the distribution of determinants of well-being. Wave 1 of the data included the following countries and territories: Argentina, Australia, Brazil, Egypt, Germany, Hong Kong, India, Indonesia, Israel, Japan, Kenya, Mexico, Nigeria, the Philippines, Poland, South Africa, Spain, Sweden, Tanzania, Turkey, UK, and the US. The countries were selected to (a) maximize coverage of the world’s population, (b) ensure geographic, cultural, and religious diversity, and (c) prioritize feasibility and existing data collection infrastructure. Data collection was carried out by Gallup Inc. Data for Wave 1 were collected principally during 2023, with some countries beginning data collection in 2022 and exact dates varying by country^35^. Four additional waves of panel data on the participants will be collected annually from 2024 to 2027. The precise sampling design varied by country in order to ensure nationally representative samples within each country, and further details are available elsewhere^35^. Survey items included aspects of well-being such as happiness, health, meaning, character, relationships, and financial stability^40^, along with other demographic, social, economic, political, religious, personality, childhood, community, health, and well-being variables. The data are publicly available through the Center for Open Science (https://www.cos.io/gfs). During the translation process, Gallup adhered to the TRAPD model (translation, review, adjudication, pretesting, and documentation) for cross-cultural survey research (ccsg.isr.umich.edu/chapters/translation/overview).

### Measures

*Demographics variables.* Continuous age was classified as 18–24, 25–29, 30–39, 40–49, 50–59, 60–69, 70–79, and 80 or older. Gender was assessed as male, female, or ‘other’. Marital status was assessed as single/never married, married, separated, divorced, widowed, and domestic partner. Employment was assessed as employed, self-employed, retired, student, homemaker, unemployed and searching, and ‘other’. Education was assessed as up to 8 years, 9–15 years, and 16 + years. Religious service attendance was assessed as more than once/week, once/week, one-to-three times/month, a few times/year, or never. Immigration status was dichotomously assessed with: “Were you born in this country, or not?” Response options included 'born in this country’ or 'born in another country.' Religious tradition/affiliation was characterized using the categories of Christianity, Islam, Hinduism, Buddhism, Judaism, Sikhism, Baha’i, Jainism, Shinto, Taoism, Confucianism, Primal/Animist/Folk religion, Spiritism, African-Derived, some other religion, or no religion/atheist/agnostic; precise response categories varied by country^36^. Racial/ethnic identity was assessed in some, but not all, countries, with response categories varying by country. For additional details see the COS GFS codebook or other materials^31^.

*Outcome variable.* Volunteering was assessed with a single item, which asked, “In the past month, have you volunteered your time to an organization?” Response options were binary (yes/no).

### Statistical analysis

We estimated descriptive statistics for the full sample, weighted to be nationally representative within each country for each of the demographic variables. Nationally representative proportions for volunteering were estimated separately for each country and are presented in order from highest to lowest along with 95% confidence intervals and standard deviations. Variation in proportions for volunteering across demographic categories were estimated, with all analyses initially conducted by country (see Supplementary Material). The primary outcome was the global proportions of volunteering obtained by conducting random effects meta-analyses of country-specific proportions of volunteering in each specific demographic category^41,42^ along with 95% confidence intervals, standard errors, lower and upper limits of a 95% prediction interval across countries, heterogeneity (τ), and I^2^ for evidence concerning variation within a particular demographic variable across countries^43^. Forest plots of estimates to help illustrate the heterogeneity of the proportions of volunteering by country are available in the online supplement. All meta-analyses were conducted in **R**^44^ using the metafor package^45^. Within each country, we conducted a global test of variation of volunteering across categories of each particular demographic variable. Next, a harmonic mean *p*-value (pooled, or global *p*-value) was used to combine *p*-values across different countries to test the null hypothesis of no differences in the proportion for each construct indicator among subgroups in all countries, against the alternative hypothesis that in at least one country the proportion differs among subgroups defined by that demographic variable^37^. This pooled *p*-value^46^ across countries reported evidence for variation within any country. Bonferroni corrected *p*-value thresholds are provided based on the number of demographic variables^47,48^. Religious affiliation/tradition and race/ethnicity were used, when available, as additional demographic grouping variables to test for differences in volunteering within country (for country-specific demographic analyses), but these estimates were not included in the meta-analyses since the availability of these response categories varied by country (i.e., because only some countries asked about religious affiliation/tradition and race/ethnicity, and response options differed between countries, these variables could not be included in meta-analyses). Thus, in some countries, *p*-values were constructed after adjustment for religious affiliation/tradition and race/ethnicity (when applicable), while not in other countries. As a supplementary analysis, we conducted population weighted meta-analyses. All analyses were pre-registered with COS prior to data access (https://doi.org/10.17605/OSF.IO/6NDS5); all code to reproduce analyses are openly available in an online repository^34^.

### Missing data

We imputed missing data on all variables using multivariate imputation by chained equations and five imputed datasets were used^49–52^ (for rationale for five imputed datasets, cf^37^). To account for variation in the assessment of certain variables across countries (e.g., religious affiliation/tradition and race/ethnicity), we conducted the imputation process separately in each country. This within-country imputation approach ensured that the imputation models accurately reflected country-specific contexts and assessment methods. We included sampling weights in the imputation model to account for missingness related to probability of study inclusion.

### Accounting for complex sampling design

The GFS used different sampling schemes across countries based on availability of existing panels and recruitment needs^35^. All analyses accounted for the complex survey design components by including weights, primary sampling units, and strata. Additional methodological details, including accounting for the complex sampling design, are provided elsewhere^37,38^.

## Results

Table 1 provides nationally representative descriptive statistics on demographic characteristics of the overall sample (all 22 countries combined). The sample was generally evenly distributed across age groups (apart from participants aged over 80) and gender apart from ‘other’ gender identities (*N* = 103,488 female participants [51%]). The sample primarily reported: being married (*N* = 107,354 [53%]), being employed for an employer (*N* = 78,815 [39%]), attending any religious services (*N* = 126,879 [63%]), having 9–15 years of education (*N* = 115,097 [57%]), and being native to their home country (*N* = 190,998 [94%]). The Supplementary Material provides nationally representative descriptive statistics across demographic categories by country (see Supplementary Tables S1a–S22a).

**Table 1:** Nationally representative descriptive statistics of the observed sample.

Characteristic	*N* = 202,898^1^
Age group	
18–24	27,007 (13%)
25–29	20,700 (10%)
30–39	40,256 (20%)
40–49	34,464 (17%)
50–59	31,793 (16%)
60–69	27,763 (14%)
70–79	16,776 (8.3%)
80 or older	4,119 (2.0%)
Missing	20 (< 0.1%)
Gender	
Male	98,411 (49%)
Female	103,488 (51%)
Other	602 (0.3%)
Missing	397 (0.2%)
Marital status	
Married	107,354 (53%)
Separated	5,195 (2.6%)
Divorced	11,654 (5.7%)
Widowed	9,823 (4.8%)
Never	52,115 (26%)
Domestic partner	14,931 (7.4%)
Missing	1,826 (0.9%)
Employment	
Employed for an employer	78,815 (39%)
Self-employed	36,362 (18%)
Retired	29,303 (14%)
Student	10,726 (5.3%)
Homemaker	21,677 (11%)
Unemployed and looking for a job	16,790 (8.3%)
None of these/other	8,431 (4.2%)
Missing	793 (0.4%)
Religious service attendance	
> 1/week	26,537 (13%)
1/week	39,157 (19%)
1–3/month	19,749 (9.7%)
A few times a year	41,436 (20%)
Never	75,297 (37%)
Missing	722 (0.4%)
Education	
Up to 8 years	45,078 (22%)
9–15 years	115,097 (57%)
16 + years	42,578 (21%)
Missing	146 (< 0.1%)
Immigration	
Born in this country	190,998 (94%)
Born in another country	9,791 (4.8%)
Missing	2,110 (1.0%)
Country	
Argentina	6,724 (3.3%)
Australia	3,844 (1.9%)
Brazil	13,204 (6.5%)
Egypt	4,729 (2.3%)
Germany	9,506 (4.7%)
Hong Kong	3,012 (1.5%)
India	12,765 (6.3%)
Indonesia	6,992 (3.4%)
Israel	3,669 (1.8%)
Japan	20,543 (10%)
Kenya	11,389 (5.6%)
Mexico	5,776 (2.8%)
Nigeria	6,827 (3.4%)
Philippines	5,292 (2.6%)
Poland	10,389 (5.1%)
South Africa	2,651 (1.3%)
Spain	6,290 (3.1%)
Sweden	15,068 (7.4%)
Tanzania	9,075 (4.5%)
Turkey	1,473 (0.7%)
United Kingdom	5,368 (2.6%)
United States	38,312 (19%)

^1^n (%).

Consistent with our expectations, Table 2 shows variation in the ordered proportions of volunteering between countries. Nigeria had the highest proportion for volunteering (0.51, 95% CI [0.48, 0.53]), followed by Indonesia (0.46, 95% CI [0.44, 0.48]) and Kenya (0.40, 95% CI [0.38, 0.41]) while Japan (0.09, 95% CI [0.08, 0.09]), Poland (0.08, 95% CI [0.07, 0.09]), and Egypt (0.04, 95% CI [0.04, 0.05]) had the lowest ordered proportions of volunteering – all under 10%. Countries in-between these ranged from 11% (Tanzania; 0.11, 95% CI [0.10, 0.12] and Sweden; 0.11, 95% CI [0.11, 0.12]) to 35% (Hong Kong; 0.35, 95% CI [0.32, 0.37]). Meta-analyzing across these 22 countries, the overall estimated proportion of the population who reported volunteering was 0.24 (95% CI [0.19, 0.29]).

**Table 2:** Ordered proportions of volunteering.

Country	Proportion	95% CI	SE
Nigeria	0.51	(0.48, 0.53)	0.01
Indonesia	0.46	(0.44, 0.48)	0.01
Kenya	0.40	(0.38, 0.41)	0.01
Hong Kong	0.35	(0.32, 0.37)	0.01
Australia	0.34	(0.32, 0.36)	0.01
United Kingdom	0.30	(0.28, 0.31)	0.01
India	0.29	(0.27, 0.31)	0.01
United States	0.27	(0.26, 0.28)	< 0.01
Philippines	0.26	(0.24, 0.27)	0.01
South Africa	0.25	(0.22, 0.27)	0.01
Argentina	0.21	(0.20, 0.22)	0.01
Germany	0.21	(0.20, 0.22)	0.01
Israel	0.21	(0.18, 0.23)	0.01
Mexico	0.21	(0.20, 0.23)	0.01
Brazil	0.19	(0.18, 0.19)	< 0.01
Spain	0.17	(0.16, 0.19)	0.01
Turkey	0.15	(0.13, 0.17)	0.01
Sweden	0.11	(0.11, 0.12)	<0.01
Tanzania	0.11	(0.10, 0.12)	0.01
Japan	0.09	(0.08, 0.09)	< 0.01
Poland	0.08	(0.07, 0.09)	0.01
Egypt	0.04	(0.04, 0.05)	< 0.01

Table 3 presents random effects meta-analytic proportions across countries, which shows between-country variation in volunteering rates across all demographic factors. In general, we found few differences in volunteering across age groups, marital status and immigration status. Specifically, volunteering appeared stable across age groups (proportion range of 0.19–0.22 for most age groups, except for the 80 or older age group (0.11, 95% CI [0.05, 0.23]); though there was uncertainty in the point estimates for this age group). Rates of volunteering amongst categories of marital status (range: 0.21–0.22) and immigration status (range: 0.19–0.21) were quite similar.

**Table 3:** Random effects meta-analysis of volunteering proportions by demographic category.

Variable	Category	Proportion	95% CI of Proportion	SE Analogue (CI Width/4)	Prediction Interval				
					LL	UL	τ	I^2	Global p-value
Age group	18–24	0.22	(0.18,0.28)	0.03	0.07	0.46	0.12	94.4	< 0.001**
	25–29	0.21	(0.16,0.27)	0.03	0.04	0.50	0.13	95.3	
	30–39	0.20	(0.15,0.26)	0.03	0.04	0.52	0.12	95.4	
	40–49	0.22	(0.16,0.28)	0.03	0.04	0.55	0.14	95.9	
	50–59	0.22	(0.16,0.29)	0.03	0.04	0.56	0.14	96.1	
	60–69	0.21	(0.16,0.27)	0.03	0.07	0.51	0.12	95.2	
	70–79	0.19	(0.14,0.25)	0.03	0.05	0.46	0.13	95.9	
	80 or older	0.11	(0.05,0.23)	0.04	0.00	0.44	0.20	99.2	
Gender	Male	0.23	(0.18,0.29)	0.03	0.06	0.57	0.13	95.4	< 0.001**
	Female	0.20	(0.15,0.25)	0.03	0.04	0.43	0.12	95.1	
	Other	0.08	(0.02,0.30)	0.07	0.00	0.74	0.24	99.7	
Marital status	Married	0.22	(0.17,0.29)	0.03	0.05	0.51	0.14	95.7	< 0.001**
	Separated	0.21	(0.18,0.26)	0.02	0.07	0.39	0.09	91.6	
	Divorced	0.21	(0.15,0.28)	0.03	0.03	0.59	0.15	96.7	
	Widowed	0.21	(0.16,0.27)	0.03	0.02	0.43	0.13	95.9	
	Domestic partner	0.21	(0.15,0.29)	0.03	0.06	0.79	0.16	96.9	
	Single, never married	0.21	(0.16,0.26)	0.02	0.06	0.49	0.11	94.3	
Employment status	Employed for an employer	0.22	(0.17,0.28)	0.03	0.05	0.55	0.13	95.3	< 0.001**
	Self-employed	0.26	(0.20,0.32)	0.03	0.05	0.52	0.14	95.2	
	Retired	0.21	(0.16,0.27)	0.03	0.07	0.53	0.12	95.3	
	Student	0.24	(0.19,0.29)	0.03	0.08	0.58	0.12	94.0	
	Homemaker	0.18	(0.13,0.24)	0.03	0.02	0.44	0.12	96.0	
	Unemployed and looking for a job	0.18	(0.13,0.24)	0.03	0.03	0.50	0.13	96.2	
	None of these/other	0.16	(0.10,0.26)	0.04	0.00	0.49	0.19	98.5	
Education	Up to 8 years	0.17	(0.12,0.24)	0.03	0.03	0.47	0.13	96.6	< 0.001**
	9–15 years	0.21	(0.16,0.26)	0.03	0.06	0.51	0.12	94.9	
	16 + years	0.27	(0.22,0.34)	0.03	0.08	0.65	0.14	94.8	
Religious service attendance	> 1/week	0.37	(0.28,0.47)	0.05	0.06	0.82	0.23	97.4	< 0.001**
	1/week	0.30	(0.23,0.38)	0.04	0.06	0.60	0.18	96.5	
	1–3/month	0.26	(0.20,0.33)	0.03	0.05	0.51	0.15	95.7	
	A few times a year	0.20	(0.16,0.25)	0.02	0.07	0.45	0.10	92.5	
	Never	0.15	(0.11,0.20)	0.02	0.03	0.46	0.10	95.1	
Immigration status	Born in this country	0.21	(0.17,0.27)	0.03	0.05	0.50	0.13	95.1	< 0.001**
	Born in another country	0.19	(0.12,0.29)	0.04	0.00	0.52	0.21	98.4	

*p < .05; **p < .007 (Bonferroni corrected threshold).

*Proportion*: Estimated overall proportion in the category.

*95% CI of Proportion*: The 95% CI for the estimated overall proportion of people who volunteered for each demographic category.

*SE Analogue (CI Width/4)*: Standard error for the estimated overall proportion for each demographic group.

*Prediction interval*: Reflects how the country-specific proportion vary. LL: Lower limit of the 95% prediction interval. UP: Upper limit of the 95% prediction interval.

τ (*tau; h*eterogeneity): Measures the standard deviation of the distribution of proportions across countries. It is an estimate of how much the proportion in that demographic category varies across countries.

*I^2*: Estimates how much of the variability in proportions is due to heterogeneity across countries vs. sampling variability. Given that the sample sizes of this study are large, the I^2 is high.

*Global p-value*: Tests the null hypothesis that the demographic category does not matter in any of the 22 countries

In contrast, we saw differences in volunteering based on gender, employment status, religious service attendance, and education. Males (0.23, 95% CI [0.18, 0.29]) and females (0.20, 95% CI [0.15, 0.25]) volunteered more than ‘other’ gender identities (0.08, 95% CI [0.02, 0.30]). Rates of volunteering were highest for self-employed participants (0.26, 95% CI [0.20, 0.32]) and lowest for homemakers (0.18, 95% CI [0.13, 0.24]), those unemployed and looking for a job (0.18, 95% CI [0.13, 0.24]), and those who did not fall into any employment status category (0.16, 95% CI [0.10, 0.26]). More education seemed to be associated with more volunteering—when comparing those with up to 8 years of education (0.17, 95% CI [0.12, 0.24]) as compared to those with 16 + years of education (0.27, 95% CI [0.22, 0.34]). More religious service attendance also seemed to be associated with more volunteering (e.g., for those who never attend religious services (0.15, 95% CI [0.11, 0.20]) as compared to those who attend religious services more than once per week (0.37, 95% CI [0.28, 0.47]). Further, the “tau” estimate shows how much the proportion of people who volunteered in that demographic category varied across countries. The gender category ‘other’ (0.24) had the highest “tau” estimate, suggesting that the proportion of this category may have varied more across countries. Proportions of people volunteering across religious affiliation categories and race and ethnicity can be found in Tables S1b-S22b (see Supplementary Material).

In terms of country differences, Supplementary Tables S1b–S22b provide the proportions of volunteering for each demographic category for each country separately. Supplementary Figures S1 provide forest plots for demographic factors by country. As we expected, differences across demographic categories varied by country.

Proportions across age groups were quite different between countries. In some countries, younger and middle-aged adults were more likely to volunteer (e.g., Argentina, India, Israel, Turkey, UK); in other countries, older adults were more likely to volunteer (e.g., Australia); in some, there was evidence of slight U-shaped associations (e.g., with more volunteering for younger and older adults in some countries like Japan); in several others there were no clear trends. For many groups, confidence intervals overlapped.

Although rates of volunteering were similar for males and females in the overall sample, there were differences in magnitude and direction across countries. We saw little evidence of differences between genders in some countries (e.g., Argentina, Brazil, Israel, and South Africa). In four countries (Australia, Poland, Sweden, and the US), females volunteered more than males, though confidence intervals overlapped. In remaining countries, males volunteered more than females, with some of the largest differences (male vs. female) seen in Nigeria (0.58, 95% CI [0.55, 0.61] vs. 0.43, 95% CI [0.40, 0.47]), Germany (0.26, 95% CI [0.25, 0.28] vs. 0.16, 95% CI [0.15, 0.17]), Indonesia (0.50, 95% CI [0.47, 0.52] vs. 0.42, 95% CI [0.40, 0.44]), and Spain (0.22, 95% CI [0.20, 0.24] vs. 0.12, 95% CI [0.11, 0.14]).

For marital status, findings were very mixed. Several countries (e.g., India, Indonesia, Kenya, South Africa, Spain, Tanzania) showed no differences across categories of marital status. In others, married (Hong Kong, Mexico, US), divorced (e.g., Argentina, Philippines), widowed (e.g., Japan, Germany, Poland, Sweden) and separated (e.g., Egypt, Japan) participants had higher proportions of volunteers. In several countries (e.g., Brazil, Hong Kong, Philippines, US), those who reported domestic partners had the lowest rates of volunteering. Across many groups, confidence intervals overlapped.

For employment, several countries showed that self-employed (e.g., Argentina, Germany, Hong Kong, Kenya, Mexico, South Africa) or student participants (e.g., Egypt, Indonesia) or both (e.g., Brazil) had the highest proportion of volunteers. In several countries, volunteering rates were mostly consistent across employment categories, but there were specific groups that had lower proportions of volunteering, such as unemployed individuals (e.g., in Australia, Japan), homemakers (e.g., Nigeria), or retired participants (e.g., India). One country, Spain, showed no differences across employment categories. For many groups, confidence intervals overlapped.

Globally more religious service attendance was associated with more volunteering, and this was generally true in all countries except for Nigeria, Poland, and Turkey. And although more education was associated with more volunteering in general, this was not true in Hong Kong (where less education was associated with a higher proportion of volunteering), India (no differences) and Mexico (no differences). For some groups, confidence intervals overlapped. 

Rates of volunteering across immigration status, religious affiliation, and race also varied between countries, though for many groups, confidence intervals overlapped. For immigration, some countries showed no differences between immigrants and native-born volunteering rates (e.g., Argentina, Brazil, Germany, Philippines), some showed that native persons had higher proportions of volunteering (e.g., Egypt, Hong Kong, India, Israel, US), and others showed that immigrant participants had higher proportions of volunteering (e.g., Poland, South Africa, Turkey, UK, Mexico, Japan), though some of these findings may be an artefact of methodological constraints (e.g., very low immigrant sample sizes in Egypt). Findings across religious groups were mixed, however there were some general patterns. Comparing only religious groups that made up at least 1% of the sample, the ‘non-religious’ sample was sufficiently large in 16 countries. In 11 the non-religious were the least likely to volunteer, in three the second least likely to (South Africa, Sweden, UK), and in two (Poland and Turkey) they were the most likely to volunteer. Conversely, the ‘Christian’ sample was sufficiently large in 21 countries. In 13 they were the most likely to volunteer and in several others they were close to the top. Small minority religious groups also often had the highest proportion of volunteers. Findings across racial and ethnic groups varied between countries and are difficult to compare between countries due to the presence of different racial and ethnic groups between countries.

Supplementary Table S23 shows the results of an alternative meta-analysis wherein each country’s results were weighted by the 2023 population size and the population-weighted meta-analysis effectively treats each person in the 22 countries equally. The results from this meta-analysis were mostly similar to the main meta-analysis results (e.g., those with more education and more religious service attendance volunteered more).

## Discussion

Using data from a diverse and international sample of 202,898 individuals across 22 countries, we examined the distribution of volunteering across countries and demographic factors, including age, gender, marital status, employment status, religious service attendance, education, immigration status, and where available, race and ethnicity, and religious affiliation.

Unadjusted proportions of participants reporting volunteering varied between countries. Nigeria, Indonesia, and Kenya all reported volunteering rates of 40% or greater, while Japan, Poland, and Egypt all reported rates of volunteering less than 10%, mostly similar to prior research^4,25^. Some countries (e.g., Indonesia and Kenya) having higher volunteering rates aligns with prior research, in which Indonesia has consistently ranked first or second for volunteering rates (63% in 2021 and 61% in 2022) with Kenya ranking second highest in 2021 (52%) and fourth highest in 2022 (51%)^4,53^. Nigeria has promoted a “massive National Youth Services Corps” since the early 1970s, which both organizes the volunteering of young people and encourages the habit of service more generally^54^.

Turning to countries with comparatively low volunteering rates, it is helpful to note that Egypt, which had a volunteer rate of 4%, has shown similarly low volunteer rates in prior work^4,25^. Government restrictions on charitable organizations in Egypt likely reduce the opportunities to volunteer, suggesting that recent legal policies might be more impactful than long-standing cultural patterns in some countries^55^. In other cases, volunteering with organizations as conceptualized in western societies may be less common than other prosocial behaviors. For example, in Japan, there was previously no Japanese word for volunteer (the Japanized *borantia*, the term used in our study, was popularized in the early 1980s^56^). A 1969 national poll used the term *shakai hoshi* (social service) and not *borantia* (volunteer) and showed that more than 30% of adults engaged in social services^56^. The Japanese may engage in other forms of helping (e.g., informal helping or helping one another through neighbourhood associations)^56^ rather than creating formal volunteer organizations and volunteering within them.

Other structural and cultural factors may also explain why some countries do not have higher rates of volunteering. As one example, consider the extent to which governments provide social services – in Sweden, where the government plays a central role in providing social services, citizens may perceive less of a need to volunteer. Other events and nation-specific contexts may further influence volunteering rates. For example, within the US, volunteering has been declining for over a decade, and fell 7 percentage points in just one year (September 2020-21)^57^. That was a challenging year globally due to the COVID-19 pandemic. That year, new work from home policies made it increasingly difficult for companies to organize volunteers, and closing face-to-face worship services may have hampered recruitment via religious organizations. Conversely, rates of volunteering in Poland increased during the first few weeks of full-scale war in Ukraine^58^. It is beyond the scope of our paper to explore all such nuances, but our findings may enhance the discussion of country-specific investigations.

Social capital and civic sphere vitality are other potential contextual factors related to volunteering rates across countries. Different forms of social capital—bonding (strong ties within close-knit groups), bridging (connections across diverse groups), and linking (relationships between individuals and institutions)—shape the networks and trust that facilitate volunteering^59^. They may also influence the social consequences of volunteering^60–62^. For example, individuals in countries with high levels of bonding social capital (e.g., Nigeria) may see volunteering rooted in familial or community traditions, while those with strong bridging social capital (e.g., United Kingdom) might be more likely to engage in cross-group volunteer initiatives, though our data cannot speak to these differences which may be reflected in the types of volunteer work being done, as well as engagement in other prosocial behaviors not evaluated in this study. Further, civic sphere vitality encompasses the health of public spaces, social trust, and citizen engagement^63^. Vibrant civic spheres may provide opportunities for individuals to connect, identify local challenges, and organize or participate in volunteer efforts. Strengthening social capital and revitalizing the civic sphere may be essential for fostering sustainable volunteering cultures, particularly during periods of social or economic strain (e.g., disruptions such as the COVID-19 pandemic which may have reduced social interactions and trust in institutions and limited opportunities for volunteerism). 

Countries’ differences in volunteering may also be influenced by historical context. Some scholars argue that volunteering is higher in former British, Dutch, and US colonies because they often had more religious liberty, greater exposure to non-state-supported missionaries, and greater religious competition^64–72^.The pattern in our data seem to be consistent with such theoretical perspectives: the ten countries with the highest proportion of volunteering (Table 2) were all former British, Dutch, or US colonies (plus the UK), while the twelve countries with the lowest proportion of volunteering were mostly Spanish and Portuguese colonies, non-colonies, or European societies that had a strong state church and religious conformity. However, our data do not allow us to test this theory rigorously or to rule out other possible explanations. Another theory focuses on the role of religious minorities in organizing civil society, particularly in repressive environments^73,74^. Scholars have argued that minority groups have higher commitment, greater bonds of trust, and greater empathy for other minorities, and these characteristics make it easier to organize collective action in repressive environments and instill greater willingness to protect repressed groups (though our data do not focus on high-cost collective action). We see some evidence of this in our data (e.g., in several countries, religious minorities volunteered more than or as much as religious majorities); however in other places, majority groups (e.g., Christians) volunteered more than religious minorities. This prior work^73,74^ implies that all minorities, not just religious ones, could be more likely to volunteer, but we did not find this pattern in our sample. The non-religious consistently volunteered less than religious groups, immigrants did not consistently volunteer more than non-immigrants, and racial/ethnic minorities did not consistently volunteer more than dominant racial/ethnic communities.

With regard to demography, in the overall sample, age had little association with volunteering until after ‘retirement age.’ For age groups 18–24 to 60–69, the proportion who volunteered hovered from 20 to 22%. However, later in life (70–79 and 80 or older) rates seemed to decline, consistent with prior research. Volunteering may decline in these oldest age groups because of physical or cognitive impairments later in life^75^, or because older age groups are caring for grandchildren at home. However, when we look at individual countries in Supplemental Tables S1b-S22b, the pattern is more complex. In some Sub-Saharan African countries (e.g., Kenya, Nigeria), those in the middle-aged groups volunteered the most. In several countries (e.g., India, Israel, Spain, Turkey, and the UK), volunteering decreased with age; in Australia it increased with age. In Japan, the oldest and youngest volunteered the most, perhaps because of the high expectations on working hours and long commutes for working-age adults. In the US, volunteering was higher for 18–24 year-olds (when most are students), dropped when people move into the work force, and gradually increased over time until declining again for those over 80. In other societies, the pattern is more complex, with specific age groups volunteering more or less. It may be that cultural exposures shape a cohorts long-term engagement in volunteering. For example, age groups that volunteered more often may have been involved in major protest/democratization movements. For example, in Hong Kong those who were 18–24 and 50–59 volunteered more; they were students during the two major democratization/protest movements of 1989 (Tiananmen) and 2014 (Umbrella Movement). In Poland, those 60–69 were more likely to volunteer. They were students or young workers during the mobilizations and protests link to the Solidarity Trade Union 1980–1989 that helped spur the fall of the Communist regime.

Regarding gender, males volunteered more in 14 countries, females more in four countries, and there was no significant difference in the remaining four countries. Proportions for those who reported their gender identity as ‘other’ should be interpreted with caution because sample sizes are low in most countries. Observed differences between males and females may reflect the fact that males often have more resources (e.g., financial) that facilitate volunteering^76,77^. Likewise, females may do more “invisible work” and informal volunteering in their homes (e.g., cleaning, taking care of children, care for relatives and friends, etc.) that may make them less available to volunteer. Countries where more females report volunteering than males (e.g., Australia, Poland, Sweden, and the US) may have more progressive gender roles or different societal expectations.

Rates of volunteering across marital statuses did not differ in the overall sample, but did in many countries. In several countries, people who were divorced, widowed, or separated volunteered the most (or were in the group of marital statuses that volunteered the most). One possible explanation is that these groups seek out social connection in sanctioned and safe settings through volunteering. Previous research suggests that married individuals are most likely to volunteer^18^, but this may because most of the research is based in the US and Europe. In our sample, married individuals were most likely to volunteer only in the US, Hong Kong, and Mexico.

In the overall sample and several individual countries, self-employed and student participants tended to volunteer more. Conversely, homemakers and retired people were seldom in the top group and often in the bottom group. Countries where homemakers volunteered more (e.g., Australia, Sweden, and the US) were wealthier societies in regions with relatively high gender equality^78^. Thus, while flexibility of time may foster volunteering, it is not the only factor.

Consistent with prior research, education was positively associated with volunteering in most societies^13,14,18,21^. However, in Hong Kong, there was a negative association, and in India and Mexico the association was not significant. Religious service attendance was also positively associated with volunteering in most societies^18,22–24^ However, in Turkey, Nigeria, and Poland the association was not significant. Potential explanations for this include the limited variation in religious service attendance in Nigeria (only 1.1% never attend religious services) and the inverse relationship between education and religiosity in Poland and Turkey^79,80^. Because the models do not control for education, any positive association between religious service attendance and volunteering may be counteracted by the association between education and volunteering—future waves of data in the GFS will give further insight into associations through longitudinal study designs and robust covariate adjustment^32^. Those who were non-religious tended to have lower rates of volunteering.

The association between immigrant status and volunteerism varied by country: in some countries it was not significant, in some immigrants volunteered more, and in some non-immigrants volunteered more. Differences between immigrants and non-immigrants may become less pronounced over time as immigrants settle into their host countries^20^.

It should be noted that these are descriptive analyses and should not be interpreted causally (e.g., while religious service attendance may influence one’s likelihood of volunteering, it is also possible that volunteering influences one’s likelihood of attending religious services)^75,81^. These demographic descriptive statistics simply inform us of the proportion of people who volunteered in each demographic category.

We view this national variance in demographic differences as illuminating possible pathways for increasing volunteering. For example, in countries where volunteering is lower (e.g., Tanzania), we might look to demographic groups where it is higher (e.g., in those employed for an employer vs. unemployed) and leverage their experiences as ambassadorial for the causes often addressed by volunteers (e.g., uncover how and why employed persons may be more likely to volunteer). Similarly, although we can only offer speculations based on the patterns we uncovered, these findings may have potential policy implications. For instance, when volunteering is culturally normative, where places of employment or educational settings organize or allow for volunteering without detracting from advancement of personal goals, where there is greater religious liberty and protection of religious minorities, and when volunteering is structured such that it meets social needs for connection, it is possible that volunteering will increase.

Our study has several limitations. First, item translation between languages may lead to different interpretations of our volunteering item. Rates of volunteering may appear higher or lower than they actually are due to translation and interpretation of the volunteering item. Second, national contexts may influence what is defined as volunteering, which makes cross-national comparisons challenging^24^. Third, these are descriptive analyses (without adjustment for other sociodemographic factors) and should not be interpreted causally. Fourth, these descriptive statistics capture patterns at a certain point in time—rates of volunteering in countries and across demographic groups may change over time (e.g., in Scandinavia, gender differences in volunteering have been decreasing in recent years^82^) and our findings may be influenced by seasonal effects. For all the reasons above, we must be careful about interpreting specific rankings; confidence intervals moreover sometimes overlap. Our study also has several strengths, including: comparison of a large number of demographic variables across many countries, consistency in operationalization of variables, and nationally representative samples^31^.

In this study, we examined the distribution of volunteering across countries and demographic groups, using data from a diverse and international sample of 202,898 individuals across 22 countries. As we continue to identify the substantial contributions of volunteering to the well-being of individuals and societies, it is important to understand how participation in these activities differs across national contexts and sociodemographic groups. Given that certain demographic groups have lower rates of participation in volunteering, with further research, it may be possible to remove barriers and increase facilitators to participation for these groups to ensure accessibility for able and willing volunteers. Further, better understanding where volunteering is (or is not) common internationally may provide space for organizations to enhance volunteerism worldwide.

## Electronic supplementary material

Below is the link to the electronic supplementary material.

Supplementary Material 1

## Data Availability

Data for Wave 1 of the Global Flourishing Study is available through the Center for Open Science upon submission of a pre-registration (https://doi.org/10.17605/OSF.IO/3JTZ8). Please see https://doi.org/10.17605/OSF.IO/3JTZ8 for more information about data access. All analyses were pre-registered with COS prior to data access (https://doi.org/10.17605/OSF.IO/6NDS5); all code to reproduce analyses are openly available in an online repository (https://doi.org/10.17605/OSF.IO/VBYPE).
