# Supplementary material for: Understanding who volunteers globally through an examination of demographic variation in volunteering across 22 countries
Source: Sci Rep. 2025 Jul 13;15:25299. doi: 10.1038/s41598-025-05459-2 (PMC12256606; doi:10.1038/s41598-025-05459-2)

**Supplementary Material**

***Understanding who volunteers globally through an examination of demographic variation in volunteering across 22 countries***

This online supplement to the Global Flourishing Study paper on volunteering has several important caveats to interpretation. First, estimating the within country group proportions can be unstable if the group size is small (<1%) of the country sample size. In such cases, the uncertainty in the estimate leads to a multiple imputation adjusted degrees of freedom less than 1. This means there is not enough information to evaluate the uncertainty in the estimate. We flagged such cases with a “*”. Secondly, comparing results across countries should be done with caution due to possible measurement non-invariance and differences in translation.

**Supplementary Tables**

**Table S1a.** Nationally representative descriptive statistics for Argentina

**Table S1b.** Proportions by demographic category for Argentina

**Table S2a.** Nationally representative descriptive statistics for Australia

**Table S2b.** Proportions by demographic category for Australia

**Table S3a.** Nationally representative descriptive statistics for Brazil

**Table S3b.** Proportions by demographic category for Brazil

**Table S4a.** Nationally representative descriptive statistics for Egypt

**Table S4b.** Proportions by demographic category for Egypt

**Table S5a.** Nationally representative descriptive statistics for Germany

**Table S5b.** Proportions by demographic category for Germany

**Table S6a.** Nationally representative descriptive statistics for Hong Kong

**Table S6b.** Proportions by demographic category for Hong Kong

**Table S7a.** Nationally representative descriptive statistics for India

**Table S7b.** Proportions by demographic category for India

**Table S8a.** Nationally representative descriptive statistics for Indonesia

**Table S8b.** Proportions by demographic category for Indonesia

**Table S9a.** Nationally representative descriptive statistics for Israel

**Table S9b.** Proportions by demographic category for Israel

**Table S10a.** Nationally representative descriptive statistics for Japan

**Table S10b.** Proportions by demographic category for Japan

**Table S11a.** Nationally representative descriptive statistics for Kenya

**Table S11b.** Proportions by demographic category for Kenya

**Table S12a.** Nationally representative descriptive statistics for Mexico

**Table S12b.** Proportions by demographic category for Mexico

**Table S13a.** Nationally representative descriptive statistics for Nigeria

**Table S13b.** Proportions by demographic category for Nigeria

**Table S14a.** Nationally representative descriptive statistics for Philippines

**Table S14b.** Proportions by demographic category for Philippines

**Table S15a.** Nationally representative descriptive statistics for Poland

**Table S15b.** Proportions by demographic category for Poland

**Table S16a.** Nationally representative descriptive statistics for South Africa

**Table S16b.** Proportions by demographic category for South Africa

**Table S17a.** Nationally representative descriptive statistics for Spain

**Table S17b.** Proportions by demographic category for Spain

**Table S18a.** Nationally representative descriptive statistics for Sweden

**Table S18b.** Proportions by demographic category for Sweden

**Table S19a.** Nationally representative descriptive statistics for Tanzania

**Table S19b.** Proportions by demographic category for Tanzania

**Table S20a.** Nationally representative descriptive statistics for Turkey

**Table S20b.** Proportions by demographic category for Turkey

**Table S21a.** Nationally representative descriptive statistics for United Kingdom

**Table S21b.** Proportions by demographic category for United Kingdom

**Table S22a.** Nationally representative descriptive statistics for United States

**Table S22b.** Proportions by demographic category for United States

**Table S23.** Population weighted meta-analysis of results demographic group proportions

**Supplementary Figures**

**Figure S1.** Forest plot for ‘Age group’ – ‘18-24’

**Figure S2.** Forest plot for ‘Age group’ – ‘25-29’

**Figure S3.** Forest plot for ‘Age group’ – ‘30-39’

**Figure S4.** Forest plot for ‘Age group’ – ‘40-49’

**Figure S5.** Forest plot for ‘Age group’ – ‘50-59’

**Figure S6.** Forest plot for ‘Age group’ – ‘60-69’

**Figure S7.** Forest plot for ‘Age group’ – ‘70-79’

**Figure S8.** Forest plot for ‘Age group’ – ‘80 or older’

**Figure S9.** Forest plot for ‘Gender’ – ‘Male’

**Figure S10.** Forest plot for ‘Gender’ – ‘Female’

**Figure S11.** Forest plot for ‘Gender’ – ‘Other’

**Figure S12.** Forest plot for ‘Marital status’ – ‘Married’

**Figure S13.** Forest plot for ‘Marital status’ – ‘Separated’

**Figure S14.** Forest plot for ‘Marital status’ – ‘Divorced’

**Figure S15.** Forest plot for ‘Marital status’ – ‘Widowed’

**Figure S16.** Forest plot for ‘Marital status’ – ‘Single, never married’

**Figure S17.** Forest plot for ‘Marital status’ – ‘Domestic partner’

**Figure S18.** Forest plot for ‘Employment status’ – ‘Employed for an employer’

**Figure S19.** Forest plot for ‘Employment status’ – ‘Self-employed’

**Figure S20.** Forest plot for ‘Employment status’ – ‘Retired’

**Figure S21.** Forest plot for ‘Employment status’ – ‘Student’

**Figure S22.** Forest plot for ‘Employment status’ – ‘Homemaker’

**Figure S23.** Forest plot for ‘Employment status’ – ‘Unemployed and looking for a job’

**Figure S24.** Forest plot for ‘Employment status’ – ‘None of these/other’

**Figure S25.** Forest plot for ‘Religious service attendance’ – ‘>1/week’

**Figure S26.** Forest plot for ‘Religious service attendance’ – ‘1/week’

**Figure S27.** Forest plot for ‘Religious service attendance’ – ‘1-3/month’

**Figure S28.** Forest plot for ‘Religious service attendance’ – ‘A few times a year’

**Figure S29.** Forest plot for ‘Religious service attendance’ – ‘Never’

**Figure S30.** Forest plot for ‘Education’ – ‘Up to 8 years’

**Figure S31.** Forest plot for ‘Education’ – ‘9-15 years’

**Figure S32.** Forest plot for ‘Education’ – ‘16+ years’

**Figure S33.** Forest plot for ‘Immigration status’ – ‘Born in this country’

**Figure S34.** Forest plot for ‘Immigration status’ – ‘Born in another country’

**Figure S35.** Forest plot for ‘Age group’ – ‘(Ref: 18-24) 25-29’

**Figure S36.** Forest plot for ‘Age group’ – ‘(Ref: 18-24) 30-39’

**Figure S37.** Forest plot for ‘Age group’ – ‘(Ref: 18-24) 40-49’

**Figure S38.** Forest plot for ‘Age group’ – ‘(Ref: 18-24) 50-59’

**Figure S39.** Forest plot for ‘Age group’ – ‘(Ref: 18-24) 60-69’

**Figure S40.** Forest plot for ‘Age group’ – ‘(Ref: 18-24) 70-79’

**Figure S41.** Forest plot for ‘Age group’ – ‘(Ref: 18-24) 80 or older’

**Figure S42.** Forest plot for ‘Age group’ – ‘(Ref: 25-29) 30-39’

**Figure S43.** Forest plot for ‘Age group’ – ‘(Ref: 25-29) 40-49’

**Figure S44.** Forest plot for ‘Age group’ – ‘(Ref: 25-29) 50-59’

**Figure S45.** Forest plot for ‘Age group’ – ‘(Ref: 25-29) 60-69’

**Figure S46.** Forest plot for ‘Age group’ – ‘(Ref: 25-29) 70-79’

**Figure S47.** Forest plot for ‘Age group’ – ‘(Ref: 25-29) 80 or older’

**Figure S48.** Forest plot for ‘Age group’ – ‘(Ref: 30-39) 40-49’

**Figure S49.** Forest plot for ‘Age group’ – ‘(Ref: 30-39) 50-59’

**Figure S50.** Forest plot for ‘Age group’ – ‘(Ref: 30-39) 60-69’

**Figure S51.** Forest plot for ‘Age group’ – ‘(Ref: 30-39) 70-79’

**Figure S52.** Forest plot for ‘Age group’ – ‘(Ref: 30-39) 80 or older’

**Figure S53.** Forest plot for ‘Age group’ – ‘(Ref: 40-49) 50-59’

**Figure S54.** Forest plot for ‘Age group’ – ‘(Ref: 40-49) 60-69’

**Figure S55.** Forest plot for ‘Age group’ – ‘(Ref: 40-49) 70-79’

**Figure S56.** Forest plot for ‘Age group’ – ‘(Ref: 40-49) 80 or older’

**Figure S57.** Forest plot for ‘Age group’ – ‘(Ref: 50-59) 60-69’

**Figure S58.** Forest plot for ‘Age group’ – ‘(Ref: 50-59) 70-79’

**Figure S59.** Forest plot for ‘Age group’ – ‘(Ref: 50-59) 80 or older’

**Figure S60.** Forest plot for ‘Age group’ – ‘(Ref: 60-69) 70-79’

**Figure S61.** Forest plot for ‘Age group’ – ‘(Ref: 60-69) 80 or older’

**Figure S62.** Forest plot for ‘Age group’ – ‘(Ref: 70-79) 80 or older’

**Figure S63.** Forest plot for ‘Gender’ – ‘(Ref: Male) Female’

**Figure S64.** Forest plot for ‘Gender’ – ‘(Ref: Male) Other’

**Figure S65.** Forest plot for ‘Gender’ – ‘(Ref: Female) Other’

**Figure S66.** Forest plot for ‘Marital status’ – ‘(Ref: Married) Separated’

**Figure S67.** Forest plot for ‘Marital status’ – ‘(Ref: Married) Divorced’

**Figure S68.** Forest plot for ‘Marital status’ – ‘(Ref: Married) Widowed’

**Figure S69.** Forest plot for ‘Marital status’ – ‘(Ref: Married) Single, never married’

**Figure S70.** Forest plot for ‘Marital status’ – ‘(Ref: Married) Domestic partner’

**Figure S71.** Forest plot for ‘Marital status’ – ‘(Ref: Separated) Divorced’

**Figure S72.** Forest plot for ‘Marital status’ – ‘(Ref: Separated) Widowed’

**Figure S73.** Forest plot for ‘Marital status’ – ‘(Ref: Separated) Single, never married’

**Figure S74.** Forest plot for ‘Marital status’ – ‘(Ref: Separated) Domestic partner’

**Figure S75.** Forest plot for ‘Marital status’ – ‘(Ref: Divorced) Widowed’

**Figure S76.** Forest plot for ‘Marital status’ – ‘(Ref: Divorced) Single, never married’

**Figure S77.** Forest plot for ‘Marital status’ – ‘(Ref: Divorced) Domestic partner’

**Figure S78.** Forest plot for ‘Marital status’ – ‘(Ref: Widowed) Single, never married’

**Figure S79.** Forest plot for ‘Marital status’ – ‘(Ref: Widowed) Domestic partner’

**Figure S80.** Forest plot for ‘Marital status’ – ‘(Ref: Single, never married) Domestic partner’

**Figure S81.** Forest plot for ‘Employment status’ – ‘(Ref: Employed for an employer) Self-employed’

**Figure S82.** Forest plot for ‘Employment status’ – ‘(Ref: Employed for an employer) Retired’

**Figure S83.** Forest plot for ‘Employment status’ – ‘(Ref: Employed for an employer) Student’

**Figure S84.** Forest plot for ‘Employment status’ – ‘(Ref: Employed for an employer) Homemaker’

**Figure S85.** Forest plot for ‘Employment status’ – ‘(Ref: Employed for an employer) Unemployed and looking for a job’

**Figure S86.** Forest plot for ‘Employment status’ – ‘(Ref: Employed for an employer) None of these/other’

**Figure S87.** Forest plot for ‘Employment status’ – ‘(Ref: Self-employed) Retired’

**Figure S88.** Forest plot for ‘Employment status’ – ‘(Ref: Self-employed) Student’

**Figure S89.** Forest plot for ‘Employment status’ – ‘(Ref: Self-employed) Homemaker’

**Figure S90.** Forest plot for ‘Employment status’ – ‘(Ref: Self-employed) Unemployed and looking for a job’

**Figure S91.** Forest plot for ‘Employment status’ – ‘(Ref: Self-employed) None of these/other’

**Figure S92.** Forest plot for ‘Employment status’ – ‘(Ref: Retired) Student’

**Figure S93.** Forest plot for ‘Employment status’ – ‘(Ref: Retired) Homemaker’

**Figure S94.** Forest plot for ‘Employment status’ – ‘(Ref: Retired) Unemployed and looking for a job’

**Figure S95.** Forest plot for ‘Employment status’ – ‘(Ref: Retired) None of these/other’

**Figure S96.** Forest plot for ‘Employment status’ – ‘(Ref: Student) Homemaker’

**Figure S97.** Forest plot for ‘Employment status’ – ‘(Ref: Student) Unemployed and looking for a job’

**Figure S98.** Forest plot for ‘Employment status’ – ‘(Ref: Student) None of these/other’

**Figure S99.** Forest plot for ‘Employment status’ – ‘(Ref: Homemaker) Unemployed and looking for a job’

**Figure S100.** Forest plot for ‘Employment status’ – ‘(Ref: Homemaker) None of these/other’

**Figure S101.** Forest plot for ‘Employment status’ – ‘(Ref: Unemployed and looking for a job) None of these/other’

**Figure S102.** Forest plot for ‘Religious service attendance’ – ‘(Ref: >1/week) 1/week’

**Figure S103.** Forest plot for ‘Religious service attendance’ – ‘(Ref: >1/week) 1-3/month’

**Figure S104.** Forest plot for ‘Religious service attendance’ – ‘(Ref: >1/week) A few times a year’

**Figure S105.** Forest plot for ‘Religious service attendance’ – ‘(Ref: >1/week) Never’

**Figure S106.** Forest plot for ‘Religious service attendance’ – ‘(Ref: 1/week) 1-3/month’

**Figure S107.** Forest plot for ‘Religious service attendance’ – ‘(Ref: 1/week) A few times a year’

**Figure S108.** Forest plot for ‘Religious service attendance’ – ‘(Ref: 1/week) Never’

**Figure S109.** Forest plot for ‘Religious service attendance’ – ‘(Ref: 1-3/month) A few times a year’

**Figure S110.** Forest plot for ‘Religious service attendance’ – ‘(Ref: 1-3/month) Never’

**Figure S111.** Forest plot for ‘Religious service attendance’ – ‘(Ref: A few times a year) Never’

**Figure S112.** Forest plot for ‘Education’ – ‘(Ref: Up to 8 years) 9-15 years’

**Figure S113.** Forest plot for ‘Education’ – ‘(Ref: Up to 8 years) 16+ years’

**Figure S114.** Forest plot for ‘Education’ – ‘(Ref: 9-15 years) 16+ years’

**Figure S115.** Forest plot for ‘Immigration status’ – ‘(Ref: Born in this country) Born in another country’

***Table S1a. Nationally representative descriptive statistics for Argentina***

| **Characteristic** | **N = 6,724**^1^ |
| --- | --- |
| **Age group** |  |
| 18-24 | 1,108 (16%) |
| 25-29 | 719 (11%) |
| 30-39 | 1,432 (21%) |
| 40-49 | 1,254 (19%) |
| 50-59 | 1,014 (15%) |
| 60-69 | 730 (11%) |
| 70-79 | 356 (5.3%) |
| 80 or older | 112 (1.7%) |
| Missing | 0 (0%) |
| **Gender** |  |
| Male | 3,143 (47%) |
| Female | 3,542 (53%) |
| Other | 21 (0.3%) |
| Missing | 18 (0.3%) |
| **Marital status** |  |
| Married | 1,565 (23%) |
| Separated | 455 (6.8%) |
| Divorced | 321 (4.8%) |
| Widowed | 401 (6.0%) |
| Never | 2,381 (35%) |
| Domestic Partner | 1,514 (23%) |
| Missing | 88 (1.3%) |
| **Employment** |  |
| Employed for an employer | 2,440 (36%) |
| Self-employed | 1,748 (26%) |
| Retired | 773 (11%) |
| Student | 354 (5.3%) |
| Homemaker | 639 (9.5%) |
| Unemployed and looking for a job | 569 (8.5%) |
| None of these/other | 179 (2.7%) |
| Missing | 22 (0.3%) |
| **Religious service attendance** |  |
| >1/week | 532 (7.9%) |
| 1/week | 773 (12%) |
| 1-3/month | 461 (6.8%) |
| A few times a year | 1,949 (29%) |
| Never | 2,982 (44%) |
| Missing | 27 (0.4%) |
| **Education** |  |
| up to 8 years | 2,263 (34%) |
| 9-15 years | 3,823 (57%) |
| 16+years | 635 (9.4%) |
| Missing | 3 (<0.1%) |
| **Immigration** |  |
| Born in this country | 6,346 (94%) |
| Born in another country | 348 (5.2%) |
| Missing | 29 (0.4%) |
| **Religious affiliation** |  |
| Christianity | 4,992 (74%) |
| Islam | 9 (0.1%) |
| Hinduism | 6 (<0.1%) |
| Buddhism | 35 (0.5%) |
| Judaism | 40 (0.6%) |
| Sikhism | 0 (<0.1%) |
| Taoism | 2 (<0.1%) |
| Confucianism | 0 (<0.1%) |
| Primal, Animist, or Folk religion | 19 (0.3%) |
| Some other religion | 156 (2.3%) |
| No religion/Atheist/Agnostic | 1,352 (20%) |
| Missing | 111 (1.7%) |
| **Race and ethnicity** |  |
| Asian | 43 (0.6%) |
| Black | 95 (1.4%) |
| Indigenous | 129 (1.9%) |
| Mestizo(a) | 1,801 (27%) |
| Mullato(a) | 75 (1.1%) |
| White | 3,406 (51%) |
| Other | 104 (1.5%) |
| Missing | 1,070 (16%) |
| ^1^n (%) | |

***Table S1b. Proportions by demographic category for Argentina***

| Variable | Category | Proportion | SE | 95% CI | Global p-value |
| --- | --- | --- | --- | --- | --- |
| Age group | 18-24 | 0.22 | 0.02 | (0.19, 0.26) | <0.001 |
|  | 25-29 | 0.22 | 0.02 | (0.18, 0.26) |  |
|  | 30-39 | 0.24 | 0.02 | (0.21, 0.27) |  |
|  | 40-49 | 0.21 | 0.01 | (0.18, 0.24) |  |
|  | 50-59 | 0.23 | 0.02 | (0.19, 0.27) |  |
|  | 60-69 | 0.16 | 0.02 | (0.12, 0.20) |  |
|  | 70-79 | 0.10 | 0.02 | (0.06, 0.14) |  |
|  | 80 or older | 0.13 | 0.05 | (0.03, 0.23) |  |
| Gender | Male | 0.21 | 0.01 | (0.19, 0.23) | 1.000 |
|  | Female | 0.21 | 0.01 | (0.19, 0.23) |  |
|  | Other | 0.20 | 0.09 | (0.01, 0.39) |  |
| Marital status | Married | 0.24 | 0.02 | (0.21, 0.27) | <0.001 |
|  | Separated | 0.23 | 0.03 | (0.18, 0.29) |  |
|  | Divorced | 0.27 | 0.03 | (0.21, 0.33) |  |
|  | Widowed | 0.14 | 0.03 | (0.09, 0.20) |  |
|  | Never | 0.20 | 0.01 | (0.18, 0.23) |  |
|  | Domestic Partner | 0.19 | 0.01 | (0.16, 0.22) |  |
| Employment | Employed for an employer | 0.22 | 0.01 | (0.19, 0.24) | <0.001 |
|  | Self-employed | 0.25 | 0.02 | (0.22, 0.28) |  |
|  | Retired | 0.11 | 0.01 | (0.09, 0.14) |  |
|  | Student | 0.21 | 0.03 | (0.15, 0.26) |  |
|  | Homemaker | 0.20 | 0.02 | (0.16, 0.24) |  |
|  | Unemployed and looking for a job | 0.21 | 0.02 | (0.16, 0.26) |  |
|  | None of these/other | 0.17 | 0.03 | (0.11, 0.24) |  |
| Religious service attendance | >1/week | 0.41 | 0.03 | (0.35, 0.47) | <0.001 |
|  | 1/week | 0.33 | 0.03 | (0.28, 0.38) |  |
|  | 1-3/month | 0.25 | 0.03 | (0.19, 0.30) |  |
|  | A few times a year | 0.20 | 0.01 | (0.17, 0.22) |  |
|  | Never | 0.14 | 0.01 | (0.13, 0.16) |  |
| Education | up to 8 years | 0.22 | 0.02 | (0.19, 0.25) | <0.001 |
|  | 9-15 years | 0.19 | 0.01 | (0.18, 0.21) |  |
|  | 16+years | 0.26 | 0.02 | (0.23, 0.30) |  |
| Immigration status | Born in this country | 0.21 | 0.01 | (0.20, 0.22) | 1.000 |
|  | Born in another country | 0.21 | 0.03 | (0.15, 0.27) |  |
| Religious affiliation | Christianity | 0.22 | 0.01 | (0.20, 0.23) | <0.001 |
|  | Islam | 0.55 | 0.25 | (-0.06, 1.16) |  |
|  | Hinduism | 0.53 | 0.30 | (-0.30, 1.36) |  |
|  | Buddhism | 0.23 | 0.07 | (0.08, 0.37) |  |
|  | Judaism | 0.17 | 0.06 | (0.05, 0.29) |  |
|  | Taoism | 0.70 | * | * |  |
|  | Primal, Animist, or Folk religion | 0.42 | 0.18 | (0.04, 0.79) |  |
|  | Some other religion | 0.22 | 0.05 | (0.12, 0.33) |  |
|  | No religion/Atheist/Agnostic | 0.17 | 0.01 | (0.15, 0.20) |  |
| Race and ethnicity | Asian | 0.31 | 0.11 | (0.09, 0.54) | 0.002 |
|  | Black | 0.22 | 0.09 | (0.02, 0.42) |  |
|  | Indigenous | 0.35 | 0.06 | (0.23, 0.48) |  |
|  | Mestizo(a) | 0.21 | 0.01 | (0.19, 0.24) |  |
|  | Mullato(a) | 0.29 | 0.08 | (0.13, 0.45) |  |
|  | White | 0.20 | 0.01 | (0.18, 0.22) |  |
|  | Other | 0.18 | 0.05 | (0.08, 0.27) |  |

***Table S2a. Nationally representative descriptive statistics for Australia***

| **Characteristic** | **N = 3,844**^1^ |
| --- | --- |
| **Age group** |  |
| 18-24 | 345 (9.0%) |
| 25-29 | 282 (7.3%) |
| 30-39 | 641 (17%) |
| 40-49 | 618 (16%) |
| 50-59 | 691 (18%) |
| 60-69 | 589 (15%) |
| 70-79 | 498 (13%) |
| 80 or older | 178 (4.6%) |
| Missing | 2 (<0.1%) |
| **Gender** |  |
| Male | 1,861 (48%) |
| Female | 1,941 (50%) |
| Other | 36 (0.9%) |
| Missing | 6 (0.2%) |
| **Marital status** |  |
| Married | 1,797 (47%) |
| Separated | 158 (4.1%) |
| Divorced | 332 (8.6%) |
| Widowed | 215 (5.6%) |
| Never | 855 (22%) |
| Domestic Partner | 450 (12%) |
| Missing | 38 (1.0%) |
| **Employment** |  |
| Employed for an employer | 1,881 (49%) |
| Self-employed | 380 (9.9%) |
| Retired | 912 (24%) |
| Student | 190 (5.0%) |
| Homemaker | 137 (3.6%) |
| Unemployed and looking for a job | 134 (3.5%) |
| None of these/other | 206 (5.4%) |
| Missing | 4 (0.1%) |
| **Religious service attendance** |  |
| >1/week | 162 (4.2%) |
| 1/week | 299 (7.8%) |
| 1-3/month | 135 (3.5%) |
| A few times a year | 656 (17%) |
| Never | 2,584 (67%) |
| Missing | 7 (0.2%) |
| **Education** |  |
| up to 8 years | 70 (1.8%) |
| 9-15 years | 2,434 (63%) |
| 16+years | 1,330 (35%) |
| Missing | 10 (0.3%) |
| **Immigration** |  |
| Born in this country | 2,953 (77%) |
| Born in another country | 885 (23%) |
| Missing | 6 (0.2%) |
| **Religious affiliation** |  |
| Christianity | 1,592 (41%) |
| Islam | 45 (1.2%) |
| Hinduism | 31 (0.8%) |
| Buddhism | 36 (0.9%) |
| Judaism | 26 (0.7%) |
| Sikhism | 8 (0.2%) |
| Baha’i | 7 (0.2%) |
| Taoism | 5 (0.1%) |
| Primal, Animist, or Folk religion | 23 (0.6%) |
| Some other religion | 39 (1.0%) |
| No religion/Atheist/Agnostic | 2,020 (53%) |
| Missing | 15 (0.4%) |
| **Race and ethnicity** |  |
| Aboriginal | 53 (1.4%) |
| Australian | 1,946 (51%) |
| Australian British/European | 1,047 (27%) |
| Chinese | 75 (1.9%) |
| Indian | 58 (1.5%) |
| Japanese | 1 (<0.1%) |
| Malay | 11 (0.3%) |
| Sinhalese | 1 (<0.1%) |
| Spanish | 2 (<0.1%) |
| Sri Lankan Moor | 1 (<0.1%) |
| Sri Lankan Tamil | 7 (0.2%) |
| Vietnamese | 7 (0.2%) |
| Russian | 7 (0.2%) |
| Samoan | 4 (0.1%) |
| New Zealander | 91 (2.4%) |
| Other European | 357 (9.3%) |
| Other | 163 (4.2%) |
| Missing | 14 (0.4%) |
| ^1^n (%) | |

***Table S2b. Proportions by demographic category for Australia***

| Variable | Category | Proportion | SE | 95% CI | Global p-value |
| --- | --- | --- | --- | --- | --- |
| Age group | 18-24 | 0.25 | 0.03 | (0.18, 0.31) | <0.001 |
|  | 25-29 | 0.26 | 0.04 | (0.18, 0.33) |  |
|  | 30-39 | 0.28 | 0.02 | (0.23, 0.33) |  |
|  | 40-49 | 0.38 | 0.02 | (0.33, 0.43) |  |
|  | 50-59 | 0.31 | 0.02 | (0.27, 0.34) |  |
|  | 60-69 | 0.38 | 0.02 | (0.34, 0.42) |  |
|  | 70-79 | 0.43 | 0.02 | (0.38, 0.48) |  |
|  | 80 or older | 0.44 | 0.04 | (0.36, 0.52) |  |
| Gender | Male | 0.33 | 0.01 | (0.30, 0.35) | <0.001 |
|  | Female | 0.35 | 0.01 | (0.32, 0.38) |  |
|  | Other | 0.16 | 0.07 | (0.02, 0.29) |  |
| Marital status | Married | 0.39 | 0.01 | (0.36, 0.41) | <0.001 |
|  | Separated | 0.25 | 0.04 | (0.17, 0.34) |  |
|  | Divorced | 0.37 | 0.03 | (0.31, 0.43) |  |
|  | Widowed | 0.41 | 0.04 | (0.34, 0.49) |  |
|  | Never | 0.25 | 0.02 | (0.21, 0.29) |  |
|  | Domestic Partner | 0.28 | 0.03 | (0.23, 0.34) |  |
| Employment | Employed for an employer | 0.29 | 0.01 | (0.27, 0.32) | <0.001 |
|  | Self-employed | 0.40 | 0.03 | (0.34, 0.45) |  |
|  | Retired | 0.41 | 0.02 | (0.38, 0.45) |  |
|  | Student | 0.35 | 0.05 | (0.24, 0.45) |  |
|  | Homemaker | 0.44 | 0.06 | (0.33, 0.56) |  |
|  | Unemployed and looking for a job | 0.22 | 0.05 | (0.12, 0.31) |  |
|  | None of these/other | 0.30 | 0.04 | (0.22, 0.38) |  |
| Religious service attendance | >1/week | 0.74 | 0.04 | (0.65, 0.82) | <0.001 |
|  | 1/week | 0.60 | 0.03 | (0.54, 0.67) |  |
|  | 1-3/month | 0.52 | 0.05 | (0.41, 0.62) |  |
|  | A few times a year | 0.31 | 0.02 | (0.27, 0.35) |  |
|  | Never | 0.28 | 0.01 | (0.26, 0.30) |  |
| Education | up to 8 years | 0.25 | 0.08 | (0.10, 0.41) | <0.001 |
|  | 9-15 years | 0.30 | 0.01 | (0.28, 0.33) |  |
|  | 16+years | 0.40 | 0.01 | (0.38, 0.43) |  |
| Immigration status | Born in this country | 0.34 | 0.01 | (0.32, 0.37) | 0.052 |
|  | Born in another country | 0.31 | 0.02 | (0.28, 0.35) |  |
| Religious affiliation | Christianity | 0.41 | 0.01 | (0.38, 0.43) | <0.001 |
|  | Islam | 0.32 | 0.09 | (0.15, 0.49) |  |
|  | Hinduism | 0.20 | 0.08 | (0.04, 0.36) |  |
|  | Buddhism | 0.33 | 0.10 | (0.13, 0.52) |  |
|  | Judaism | 0.32 | 0.09 | (0.14, 0.51) |  |
|  | Sikhism | 0.00 | * | * |  |
|  | Baha’i | 0.81 | 0.15 | (0.42, 1.19) |  |
|  | Taoism | 0.40 | 0.28 | (-0.62, 1.42) |  |
|  | Primal, Animist, or Folk religion | 0.48 | 0.15 | (0.16, 0.80) |  |
|  | Some other religion | 0.49 | 0.11 | (0.26, 0.71) |  |
|  | No religion/Atheist/Agnostic | 0.28 | 0.01 | (0.26, 0.30) |  |
| Race and ethnicity | Aboriginal | 0.46 | 0.10 | (0.27, 0.66) | *** |
|  | Australian | 0.34 | 0.01 | (0.32, 0.37) |  |
|  | Australian British/European | 0.34 | 0.02 | (0.30, 0.37) |  |
|  | Chinese | 0.22 | 0.06 | (0.09, 0.34) |  |
|  | Indian | 0.21 | 0.07 | (0.08, 0.34) |  |
|  | Japanese | 0.00 | * | * |  |
|  | Malay | 0.00 | * | * |  |
|  | Sinhalese | 0.61 | * | * |  |
|  | Spanish | 0.00 | * | * |  |
|  | Sri Lankan Moor | 0.00 | * | * |  |
|  | Sri Lankan Tamil | 0.06 | 0.07 | (-0.11, 0.24) |  |
|  | Vietnamese | 0.12 | 0.12 | (-0.19, 0.44) |  |
|  | Russian | 0.37 | 0.22 | (-0.19, 0.94) |  |
|  | Samoan | 0.00 | * | * |  |
|  | New Zealander | 0.28 | 0.06 | (0.16, 0.40) |  |
|  | Other European | 0.39 | 0.03 | (0.33, 0.45) |  |
|  | Other | 0.30 | 0.05 | (0.21, 0.39) |  |

***p-value could not be computed due to sparsity of the race and ethnicity variable.

***Table S3a. Nationally representative descriptive statistics for Brazil***

| **Characteristic** | **N = 13,204**^1^ |
| --- | --- |
| **Age group** |  |
| 18-24 | 1,986 (15%) |
| 25-29 | 1,468 (11%) |
| 30-39 | 2,908 (22%) |
| 40-49 | 2,638 (20%) |
| 50-59 | 2,131 (16%) |
| 60-69 | 1,435 (11%) |
| 70-79 | 510 (3.9%) |
| 80 or older | 126 (1.0%) |
| Missing | 0 (0%) |
| **Gender** |  |
| Male | 6,320 (48%) |
| Female | 6,820 (52%) |
| Other | 35 (0.3%) |
| Missing | 30 (0.2%) |
| **Marital status** |  |
| Married | 4,646 (35%) |
| Separated | 594 (4.5%) |
| Divorced | 865 (6.5%) |
| Widowed | 408 (3.1%) |
| Never | 4,347 (33%) |
| Domestic Partner | 2,081 (16%) |
| Missing | 263 (2.0%) |
| **Employment** |  |
| Employed for an employer | 3,756 (28%) |
| Self-employed | 2,918 (22%) |
| Retired | 1,536 (12%) |
| Student | 624 (4.7%) |
| Homemaker | 1,305 (9.9%) |
| Unemployed and looking for a job | 2,419 (18%) |
| None of these/other | 448 (3.4%) |
| Missing | 199 (1.5%) |
| **Religious service attendance** |  |
| >1/week | 2,386 (18%) |
| 1/week | 2,272 (17%) |
| 1-3/month | 1,398 (11%) |
| A few times a year | 3,978 (30%) |
| Never | 3,110 (24%) |
| Missing | 61 (0.5%) |
| **Education** |  |
| up to 8 years | 3,139 (24%) |
| 9-15 years | 7,665 (58%) |
| 16+years | 2,390 (18%) |
| Missing | 10 (<0.1%) |
| **Immigration** |  |
| Born in this country | 12,688 (96%) |
| Born in another country | 153 (1.2%) |
| Missing | 363 (2.7%) |
| **Religious affiliation** |  |
| Christianity | 9,911 (75%) |
| Islam | 6 (<0.1%) |
| Hinduism | 1 (<0.1%) |
| Buddhism | 37 (0.3%) |
| Judaism | 31 (0.2%) |
| Baha’i | 2 (<0.1%) |
| Jainism | 2 (<0.1%) |
| Shinto | 1 (<0.1%) |
| Taoism | 1 (<0.1%) |
| Confucianism | 6 (<0.1%) |
| Primal, Animist, or Folk religion | 15 (0.1%) |
| Spiritism | 696 (5.3%) |
| Umbanda, Candomblé, and other African-derived religions | 525 (4.0%) |
| Some other religion | 144 (1.1%) |
| No religion/Atheist/Agnostic | 1,712 (13%) |
| Missing | 113 (0.9%) |
| **Race and ethnicity** |  |
| Branca | 5,169 (39%) |
| Preta | 1,615 (12%) |
| Parda | 5,125 (39%) |
| Amarela | 238 (1.8%) |
| Indígena | 131 (1.0%) |
| Other | 61 (0.5%) |
| Missing | 865 (6.6%) |
| ^1^n (%) | |

***Table S3b. Proportions by demographic category for Brazil***

| Variable | Category | Proportion | SE | 95% CI | Global p-value |
| --- | --- | --- | --- | --- | --- |
| Age group | 18-24 | 0.21 | 0.01 | (0.19, 0.23) | <0.001 |
|  | 25-29 | 0.18 | 0.01 | (0.15, 0.20) |  |
|  | 30-39 | 0.17 | 0.01 | (0.16, 0.19) |  |
|  | 40-49 | 0.19 | 0.01 | (0.17, 0.21) |  |
|  | 50-59 | 0.18 | 0.01 | (0.16, 0.21) |  |
|  | 60-69 | 0.19 | 0.02 | (0.16, 0.23) |  |
|  | 70-79 | 0.14 | 0.03 | (0.09, 0.19) |  |
|  | 80 or older | 0.20 | 0.06 | (0.08, 0.33) |  |
| Gender | Male | 0.18 | 0.01 | (0.17, 0.20) | 0.982 |
|  | Female | 0.19 | 0.01 | (0.18, 0.20) |  |
|  | Other | 0.17 | 0.05 | (0.07, 0.26) |  |
| Marital status | Married | 0.21 | 0.01 | (0.20, 0.23) | <0.001 |
|  | Separated | 0.22 | 0.02 | (0.17, 0.27) |  |
|  | Divorced | 0.16 | 0.02 | (0.13, 0.19) |  |
|  | Widowed | 0.16 | 0.02 | (0.11, 0.20) |  |
|  | Never | 0.18 | 0.01 | (0.16, 0.19) |  |
|  | Domestic Partner | 0.14 | 0.01 | (0.13, 0.16) |  |
| Employment | Employed for an employer | 0.19 | 0.01 | (0.17, 0.20) | <0.001 |
|  | Self-employed | 0.22 | 0.01 | (0.20, 0.24) |  |
|  | Retired | 0.18 | 0.02 | (0.14, 0.21) |  |
|  | Student | 0.25 | 0.02 | (0.21, 0.29) |  |
|  | Homemaker | 0.16 | 0.01 | (0.13, 0.18) |  |
|  | Unemployed and looking for a job | 0.15 | 0.01 | (0.13, 0.17) |  |
|  | None of these/other | 0.16 | 0.02 | (0.12, 0.21) |  |
| Religious service attendance | >1/week | 0.34 | 0.01 | (0.31, 0.37) | <0.001 |
|  | 1/week | 0.23 | 0.01 | (0.21, 0.25) |  |
|  | 1-3/month | 0.23 | 0.01 | (0.20, 0.25) |  |
|  | A few times a year | 0.14 | 0.01 | (0.13, 0.16) |  |
|  | Never | 0.07 | 0.01 | (0.06, 0.08) |  |
| Education | up to 8 years | 0.18 | 0.01 | (0.16, 0.20) | <0.001 |
|  | 9-15 years | 0.17 | 0.01 | (0.16, 0.18) |  |
|  | 16+years | 0.24 | 0.01 | (0.22, 0.26) |  |
| Immigration status | Born in this country | 0.19 | 0.00 | (0.18, 0.19) | 0.123 |
|  | Born in another country | 0.25 | 0.05 | (0.15, 0.34) |  |
| Religious affiliation | Christianity | 0.19 | 0.01 | (0.18, 0.20) | <0.001 |
|  | Islam | 0.52 | 0.17 | (0.06, 0.97) |  |
|  | Hinduism | 0.50 | * | * |  |
|  | Buddhism | 0.22 | 0.09 | (0.05, 0.40) |  |
|  | Judaism | 0.33 | 0.12 | (0.08, 0.58) |  |
|  | Baha’i | 1.00 | * | * |  |
|  | Jainism | 0.19 | * | * |  |
|  | Shinto | 0.00 | * | * |  |
|  | Taoism | 0.48 | * | * |  |
|  | Confucianism | 0.00 | * | * |  |
|  | Primal, Animist, or Folk religion | 0.11 | 0.07 | (-0.04, 0.26) |  |
|  | Spiritism | 0.24 | 0.02 | (0.20, 0.27) |  |
|  | Umbanda, Candomblé, and other African-derived religions | 0.21 | 0.02 | (0.17, 0.24) |  |
|  | Some other religion | 0.25 | 0.05 | (0.15, 0.35) |  |
|  | No religion/Atheist/Agnostic | 0.11 | 0.01 | (0.09, 0.13) |  |
| Race and ethnicity | Branca | 0.18 | 0.01 | (0.16, 0.19) | <0.001 |
|  | Preta | 0.22 | 0.01 | (0.19, 0.24) |  |
|  | Parda | 0.18 | 0.01 | (0.17, 0.19) |  |
|  | Amarela | 0.21 | 0.03 | (0.15, 0.28) |  |
|  | Indígena | 0.20 | 0.04 | (0.11, 0.29) |  |
|  | Other | 0.13 | 0.04 | (0.05, 0.21) |  |

***Table S4a. Nationally representative descriptive statistics for Egypt***

| **Characteristic** | **N = 4,729**^1^ |
| --- | --- |
| **Age group** |  |
| 18-24 | 960 (20%) |
| 25-29 | 607 (13%) |
| 30-39 | 1,204 (25%) |
| 40-49 | 897 (19%) |
| 50-59 | 613 (13%) |
| 60-69 | 387 (8.2%) |
| 70-79 | 54 (1.1%) |
| 80 or older | 7 (0.2%) |
| Missing | 0 (0%) |
| **Gender** |  |
| Male | 2,394 (51%) |
| Female | 2,334 (49%) |
| Other | 0 (0%) |
| Missing | 0 (<0.1%) |
| **Marital status** |  |
| Married | 3,387 (72%) |
| Separated | 39 (0.8%) |
| Divorced | 101 (2.1%) |
| Widowed | 238 (5.0%) |
| Never | 947 (20%) |
| Domestic Partner | 0 (0%) |
| Missing | 17 (0.4%) |
| **Employment** |  |
| Employed for an employer | 1,267 (27%) |
| Self-employed | 892 (19%) |
| Retired | 253 (5.4%) |
| Student | 297 (6.3%) |
| Homemaker | 1,772 (37%) |
| Unemployed and looking for a job | 224 (4.7%) |
| None of these/other | 21 (0.4%) |
| Missing | 3 (<0.1%) |
| **Religious service attendance** |  |
| >1/week | 839 (18%) |
| 1/week | 960 (20%) |
| 1-3/month | 368 (7.8%) |
| A few times a year | 458 (9.7%) |
| Never | 2,091 (44%) |
| Missing | 12 (0.3%) |
| **Education** |  |
| up to 8 years | 2,486 (53%) |
| 9-15 years | 1,599 (34%) |
| 16+years | 643 (14%) |
| Missing | 1 (<0.1%) |
| **Immigration** |  |
| Born in this country | 4,713 (100%) |
| Born in another country | 16 (0.3%) |
| Missing | 1 (<0.1%) |
| **Religious affiliation** |  |
| Christianity | 120 (2.5%) |
| Islam | 4,607 (97%) |
| Taoism | 0 (<0.1%) |
| Missing | 1 (<0.1%) |
| **Race and ethnicity** |  |
| Arab | 4,585 (97%) |
| Turkish | 9 (0.2%) |
| Greek | 1 (<0.1%) |
| Bedouin Arab | 4 (<0.1%) |
| Nubian | 27 (0.6%) |
| Missing | 102 (2.2%) |
| ^1^n (%) | |

***Table S4b. Proportions by demographic category for Egypt***

| Variable | Category | Proportion | SE | 95% CI | Global p-value |
| --- | --- | --- | --- | --- | --- |
| Age group | 18-24 | 0.06 | 0.01 | (0.04, 0.08) | <0.001 |
|  | 25-29 | 0.04 | 0.01 | (0.02, 0.05) |  |
|  | 30-39 | 0.04 | 0.01 | (0.02, 0.05) |  |
|  | 40-49 | 0.04 | 0.01 | (0.02, 0.05) |  |
|  | 50-59 | 0.04 | 0.01 | (0.02, 0.06) |  |
|  | 60-69 | 0.07 | 0.02 | (0.04, 0.11) |  |
|  | 70-79 | 0.04 | 0.03 | (-0.01, 0.10) |  |
|  | 80 or older | 0.00 | * | * |  |
| Gender | Male | 0.06 | 0.01 | (0.04, 0.07) | <0.001 |
|  | Female | 0.03 | 0.00 | (0.02, 0.04) |  |
| Marital status | Married | 0.04 | 0.00 | (0.03, 0.05) | <0.001 |
|  | Separated | 0.12 | 0.07 | (-0.02, 0.26) |  |
|  | Divorced | 0.03 | 0.02 | (-0.02, 0.07) |  |
|  | Widowed | 0.02 | 0.01 | (0.01, 0.03) |  |
|  | Never | 0.06 | 0.01 | (0.04, 0.07) |  |
| Employment | Employed for an employer | 0.04 | 0.01 | (0.03, 0.06) | <0.001 |
|  | Self-employed | 0.05 | 0.01 | (0.03, 0.07) |  |
|  | Retired | 0.08 | 0.02 | (0.03, 0.12) |  |
|  | Student | 0.11 | 0.02 | (0.07, 0.16) |  |
|  | Homemaker | 0.02 | 0.00 | (0.01, 0.03) |  |
|  | Unemployed and looking for a job | 0.08 | 0.03 | (0.03, 0.13) |  |
|  | None of these/other | 0.00 | * | * |  |
| Religious service attendance | >1/week | 0.06 | 0.01 | (0.04, 0.08) | <0.001 |
|  | 1/week | 0.05 | 0.01 | (0.03, 0.07) |  |
|  | 1-3/month | 0.05 | 0.01 | (0.02, 0.08) |  |
|  | A few times a year | 0.07 | 0.01 | (0.05, 0.09) |  |
|  | Never | 0.03 | 0.00 | (0.02, 0.04) |  |
| Education | up to 8 years | 0.03 | 0.00 | (0.02, 0.04) | <0.001 |
|  | 9-15 years | 0.06 | 0.01 | (0.04, 0.07) |  |
|  | 16+years | 0.07 | 0.01 | (0.05, 0.10) |  |
| Immigration status | Born in this country | 0.04 | 0.00 | (0.04, 0.05) | <0.001 |
|  | Born in another country | 0.00 | * | * |  |
| Religious affiliation | Christianity | 0.08 | 0.03 | (0.03, 0.13) | 0.023 |
|  | Islam | 0.04 | 0.00 | (0.04, 0.05) |  |
| Race and ethnicity | Arab | 0.04 | 0.00 | (0.04, 0.05) | <0.001 |
|  | Turkish | 0.31 | 0.17 | (-0.10, 0.72) |  |
|  | Bedouin Arab | 0.00 | * | * |  |
|  | Nubian | 0.04 | 0.01 | (0.01, 0.07) |  |

***Table S5a. Nationally representative descriptive statistics for Germany***

| **Characteristic** | **N = 9,506**^1^ |
| --- | --- |
| **Age group** |  |
| 18-24 | 829 (8.7%) |
| 25-29 | 774 (8.1%) |
| 30-39 | 1,438 (15%) |
| 40-49 | 1,494 (16%) |
| 50-59 | 1,729 (18%) |
| 60-69 | 1,915 (20%) |
| 70-79 | 1,137 (12%) |
| 80 or older | 190 (2.0%) |
| Missing | 0 (0%) |
| **Gender** |  |
| Male | 4,641 (49%) |
| Female | 4,843 (51%) |
| Other | 11 (0.1%) |
| Missing | 11 (0.1%) |
| **Marital status** |  |
| Married | 4,784 (50%) |
| Separated | 219 (2.3%) |
| Divorced | 767 (8.1%) |
| Widowed | 409 (4.3%) |
| Never | 2,627 (28%) |
| Domestic Partner | 619 (6.5%) |
| Missing | 81 (0.9%) |
| **Employment** |  |
| Employed for an employer | 4,950 (52%) |
| Self-employed | 712 (7.5%) |
| Retired | 2,480 (26%) |
| Student | 605 (6.4%) |
| Homemaker | 251 (2.6%) |
| Unemployed and looking for a job | 288 (3.0%) |
| None of these/other | 204 (2.1%) |
| Missing | 14 (0.2%) |
| **Religious service attendance** |  |
| >1/week | 285 (3.0%) |
| 1/week | 424 (4.5%) |
| 1-3/month | 550 (5.8%) |
| A few times a year | 2,362 (25%) |
| Never | 5,876 (62%) |
| Missing | 9 (<0.1%) |
| **Education** |  |
| up to 8 years | 235 (2.5%) |
| 9-15 years | 6,094 (64%) |
| 16+years | 3,164 (33%) |
| Missing | 13 (0.1%) |
| **Immigration** |  |
| Born in this country | 8,722 (92%) |
| Born in another country | 744 (7.8%) |
| Missing | 40 (0.4%) |
| **Religious affiliation** |  |
| Christianity | 5,052 (53%) |
| Islam | 351 (3.7%) |
| Hinduism | 12 (0.1%) |
| Buddhism | 51 (0.5%) |
| Judaism | 19 (0.2%) |
| Sikhism | 5 (<0.1%) |
| Baha’i | 3 (<0.1%) |
| Shinto | 2 (<0.1%) |
| Taoism | 0 (<0.1%) |
| Confucianism | 4 (<0.1%) |
| Primal, Animist, or Folk religion | 34 (0.4%) |
| Some other religion | 60 (0.6%) |
| No religion/Atheist/Agnostic | 3,815 (40%) |
| Missing | 99 (1.0%) |
| **Race and ethnicity** |  |
| Missing | 9,506 (100%) |
| ^1^n (%) | |

***Table S5b. Proportions by demographic category for Germany***

| Variable | Category | Proportion | SE | 95% CI | Global p-value |
| --- | --- | --- | --- | --- | --- |
| Age group | 18-24 | 0.21 | 0.02 | (0.17, 0.24) | 0.048 |
|  | 25-29 | 0.25 | 0.02 | (0.21, 0.29) |  |
|  | 30-39 | 0.23 | 0.01 | (0.20, 0.25) |  |
|  | 40-49 | 0.22 | 0.01 | (0.19, 0.25) |  |
|  | 50-59 | 0.19 | 0.01 | (0.17, 0.22) |  |
|  | 60-69 | 0.20 | 0.01 | (0.17, 0.22) |  |
|  | 70-79 | 0.19 | 0.02 | (0.16, 0.23) |  |
|  | 80 or older | 0.21 | 0.03 | (0.14, 0.28) |  |
| Gender | Male | 0.26 | 0.01 | (0.25, 0.28) | <0.001 |
|  | Female | 0.16 | 0.01 | (0.15, 0.17) |  |
|  | Other | 0.35 | 0.19 | (-0.08, 0.78) |  |
| Marital status | Married | 0.22 | 0.01 | (0.20, 0.23) | 0.008 |
|  | Separated | 0.18 | 0.03 | (0.12, 0.24) |  |
|  | Divorced | 0.17 | 0.02 | (0.14, 0.20) |  |
|  | Widowed | 0.25 | 0.03 | (0.19, 0.30) |  |
|  | Never | 0.21 | 0.01 | (0.19, 0.23) |  |
|  | Domestic Partner | 0.22 | 0.02 | (0.18, 0.26) |  |
| Employment | Employed for an employer | 0.20 | 0.01 | (0.19, 0.22) | <0.001 |
|  | Self-employed | 0.30 | 0.02 | (0.25, 0.34) |  |
|  | Retired | 0.21 | 0.01 | (0.19, 0.23) |  |
|  | Student | 0.21 | 0.02 | (0.17, 0.26) |  |
|  | Homemaker | 0.19 | 0.03 | (0.13, 0.25) |  |
|  | Unemployed and looking for a job | 0.17 | 0.03 | (0.12, 0.22) |  |
|  | None of these/other | 0.18 | 0.03 | (0.13, 0.24) |  |
| Religious service attendance | >1/week | 0.31 | 0.04 | (0.24, 0.38) | <0.001 |
|  | 1/week | 0.39 | 0.03 | (0.33, 0.45) |  |
|  | 1-3/month | 0.34 | 0.03 | (0.28, 0.39) |  |
|  | A few times a year | 0.25 | 0.01 | (0.23, 0.27) |  |
|  | Never | 0.16 | 0.01 | (0.15, 0.18) |  |
| Education | up to 8 years | 0.16 | 0.03 | (0.11, 0.22) | <0.001 |
|  | 9-15 years | 0.19 | 0.01 | (0.18, 0.20) |  |
|  | 16+years | 0.25 | 0.01 | (0.23, 0.27) |  |
| Immigration status | Born in this country | 0.21 | 0.01 | (0.20, 0.22) | 0.320 |
|  | Born in another country | 0.19 | 0.02 | (0.15, 0.23) |  |
| Religious affiliation | Christianity | 0.28 | 0.01 | (0.26, 0.30) | <0.001 |
|  | Islam | 0.20 | 0.03 | (0.14, 0.26) |  |
|  | Hinduism | 0.25 | 0.16 | (-0.12, 0.62) |  |
|  | Buddhism | 0.20 | 0.07 | (0.05, 0.35) |  |
|  | Judaism | 0.28 | 0.14 | (-0.01, 0.58) |  |
|  | Sikhism | 0.26 | 0.23 | (-0.48, 1.00) |  |
|  | Baha’i | 0.00 | * | * |  |
|  | Confucianism | 0.41 | 0.34 | (-1.09, 1.91) |  |
|  | Primal, Animist, or Folk religion | 0.34 | 0.11 | (0.11, 0.57) |  |
|  | Some other religion | 0.30 | 0.09 | (0.13, 0.48) |  |
|  | No religion/Atheist/Agnostic | 0.11 | 0.01 | (0.10, 0.13) |  |

***Table S6a. Nationally representative descriptive statistics for Hong Kong***

| **Characteristic** | **N = 3,012**^1^ |
| --- | --- |
| **Age group** |  |
| 18-24 | 217 (7.2%) |
| 25-29 | 198 (6.6%) |
| 30-39 | 507 (17%) |
| 40-49 | 580 (19%) |
| 50-59 | 711 (24%) |
| 60-69 | 620 (21%) |
| 70-79 | 164 (5.5%) |
| 80 or older | 15 (0.5%) |
| Missing | 0 (0%) |
| **Gender** |  |
| Male | 1,390 (46%) |
| Female | 1,620 (54%) |
| Other | 2 (<0.1%) |
| Missing | 0 (0%) |
| **Marital status** |  |
| Married | 2,080 (69%) |
| Separated | 21 (0.7%) |
| Divorced | 105 (3.5%) |
| Widowed | 45 (1.5%) |
| Never | 723 (24%) |
| Domestic Partner | 37 (1.2%) |
| Missing | 1 (<0.1%) |
| **Employment** |  |
| Employed for an employer | 2,056 (68%) |
| Self-employed | 245 (8.1%) |
| Retired | 423 (14%) |
| Student | 55 (1.8%) |
| Homemaker | 114 (3.8%) |
| Unemployed and looking for a job | 62 (2.0%) |
| None of these/other | 39 (1.3%) |
| Missing | 18 (0.6%) |
| **Religious service attendance** |  |
| >1/week | 237 (7.9%) |
| 1/week | 567 (19%) |
| 1-3/month | 332 (11%) |
| A few times a year | 543 (18%) |
| Never | 1,332 (44%) |
| Missing | 1 (<0.1%) |
| **Education** |  |
| up to 8 years | 433 (14%) |
| 9-15 years | 2,031 (67%) |
| 16+years | 547 (18%) |
| Missing | 0 (0%) |
| **Immigration** |  |
| Born in this country | 2,637 (88%) |
| Born in another country | 321 (11%) |
| Missing | 53 (1.8%) |
| **Religious affiliation** |  |
| Christianity | 757 (25%) |
| Islam | 86 (2.8%) |
| Hinduism | 20 (0.7%) |
| Buddhism | 349 (12%) |
| Judaism | 10 (0.3%) |
| Sikhism | 2 (<0.1%) |
| Baha’i | 3 (<0.1%) |
| Jainism | 1 (<0.1%) |
| Shinto | 19 (0.6%) |
| Taoism | 97 (3.2%) |
| Confucianism | 11 (0.4%) |
| Primal, Animist, or Folk religion | 27 (0.9%) |
| Chinese folk/traditional religion | 106 (3.5%) |
| Some other religion | 4 (0.1%) |
| No religion/Atheist/Agnostic | 1,518 (50%) |
| Missing | 5 (0.2%) |
| **Race and ethnicity** |  |
| White | 15 (0.5%) |
| Other | 4 (0.1%) |
| Chinese (Cantonese) | 1,930 (64%) |
| Chinese (Chaoshan) | 201 (6.7%) |
| Chinese (Fujianese) | 117 (3.9%) |
| Chinese (Hakka) | 121 (4.0%) |
| Chinese (Shanghainese) | 89 (2.9%) |
| Chinese (Other ethnicity) | 264 (8.8%) |
| East Asian (Korean, Japanese) | 10 (0.3%) |
| Southeast Asian (Filipino, Indonesian, Thailand) | 46 (1.5%) |
| South Asian (Indian, Nepalese, Pakistani) | 17 (0.6%) |
| Taiwanese | 14 (0.4%) |
| Missing | 184 (6.1%) |
| ^1^n (%) | |

***Table S6b. Proportions by demographic category for Hong Kong***

| Variable | Category | Proportion | SE | 95% CI | Global p-value |
| --- | --- | --- | --- | --- | --- |
| Age group | 18-24 | 0.40 | 0.03 | (0.34, 0.46) | <0.001 |
|  | 25-29 | 0.33 | 0.04 | (0.26, 0.41) |  |
|  | 30-39 | 0.23 | 0.02 | (0.19, 0.27) |  |
|  | 40-49 | 0.32 | 0.02 | (0.27, 0.37) |  |
|  | 50-59 | 0.45 | 0.02 | (0.41, 0.49) |  |
|  | 60-69 | 0.37 | 0.03 | (0.31, 0.44) |  |
|  | 70-79 | 0.25 | 0.07 | (0.11, 0.40) |  |
|  | 80 or older | 0.00 | * | * |  |
| Gender | Male | 0.36 | 0.02 | (0.32, 0.39) | <0.001 |
|  | Female | 0.34 | 0.02 | (0.31, 0.37) |  |
|  | Other | 0.00 | * | * |  |
| Marital status | Married | 0.42 | 0.02 | (0.39, 0.45) | <0.001 |
|  | Separated | 0.33 | 0.21 | (-0.11, 0.78) |  |
|  | Divorced | 0.26 | 0.08 | (0.09, 0.43) |  |
|  | Widowed | 0.29 | 0.14 | (0.02, 0.57) |  |
|  | Never | 0.16 | 0.02 | (0.13, 0.19) |  |
|  | Domestic Partner | 0.12 | 0.06 | (0.00, 0.24) |  |
| Employment | Employed for an employer | 0.36 | 0.01 | (0.33, 0.38) | <0.001 |
|  | Self-employed | 0.49 | 0.04 | (0.41, 0.57) |  |
|  | Retired | 0.32 | 0.05 | (0.23, 0.41) |  |
|  | Student | 0.23 | 0.05 | (0.13, 0.34) |  |
|  | Homemaker | 0.20 | 0.07 | (0.06, 0.34) |  |
|  | Unemployed and looking for a job | 0.03 | 0.03 | (-0.02, 0.08) |  |
|  | None of these/other | 0.24 | 0.15 | (-0.08, 0.55) |  |
| Religious service attendance | >1/week | 0.82 | 0.03 | (0.76, 0.88) | <0.001 |
|  | 1/week | 0.55 | 0.03 | (0.48, 0.61) |  |
|  | 1-3/month | 0.52 | 0.04 | (0.45, 0.60) |  |
|  | A few times a year | 0.27 | 0.03 | (0.21, 0.32) |  |
|  | Never | 0.17 | 0.01 | (0.14, 0.20) |  |
| Education | up to 8 years | 0.43 | 0.05 | (0.34, 0.52) | <0.001 |
|  | 9-15 years | 0.36 | 0.01 | (0.33, 0.39) |  |
|  | 16+years | 0.24 | 0.02 | (0.19, 0.28) |  |
| Immigration status | Born in this country | 0.36 | 0.01 | (0.33, 0.38) | <0.001 |
|  | Born in another country | 0.27 | 0.04 | (0.18, 0.35) |  |
| Religious affiliation | Christianity | 0.45 | 0.03 | (0.40, 0.50) | <0.001 |
|  | Islam | 0.71 | 0.09 | (0.54, 0.89) |  |
|  | Hinduism | 0.63 | 0.15 | (0.32, 0.94) |  |
|  | Buddhism | 0.58 | 0.03 | (0.51, 0.65) |  |
|  | Judaism | 0.57 | 0.15 | (0.22, 0.93) |  |
|  | Sikhism | 0.82 | * | * |  |
|  | Baha’i | 1.00 | * | * |  |
|  | Shinto | 1.00 | * | * |  |
|  | Taoism | 0.29 | 0.07 | (0.14, 0.43) |  |
|  | Confucianism | 0.63 | 0.15 | (0.28, 0.97) |  |
|  | Primal, Animist, or Folk religion | 0.24 | 0.08 | (0.08, 0.41) |  |
|  | Chinese folk/traditional religion | 0.58 | 0.07 | (0.44, 0.71) |  |
|  | Some other religion | 0.91 | 0.10 | (0.35, 1.47) |  |
|  | No religion/Atheist/Agnostic | 0.20 | 0.01 | (0.17, 0.22) |  |
| Race and ethnicity | White | 0.27 | 0.14 | (-0.04, 0.57) | <0.001 |
|  | Other | 0.27 | 0.22 | (-0.51, 1.05) |  |
|  | Chinese (Cantonese) | 0.32 | 0.01 | (0.30, 0.35) |  |
|  | Chinese (Chaoshan) | 0.40 | 0.04 | (0.32, 0.48) |  |
|  | Chinese (Fujianese) | 0.45 | 0.05 | (0.35, 0.55) |  |
|  | Chinese (Hakka) | 0.34 | 0.05 | (0.23, 0.44) |  |
|  | Chinese (Shanghainese) | 0.45 | 0.09 | (0.26, 0.64) |  |
|  | Chinese (Other ethnicity) | 0.42 | 0.04 | (0.33, 0.50) |  |
|  | East Asian (Korean, Japanese) | 0.82 | 0.15 | (0.47, 1.17) |  |
|  | Southeast Asian (Filipino, Indonesian, Thailand) | 0.43 | 0.14 | (0.15, 0.72) |  |
|  | South Asian (Indian, Nepalese, Pakistani) | 0.09 | 0.07 | (-0.06, 0.25) |  |
|  | Taiwanese | 0.27 | 0.14 | (-0.05, 0.59) |  |

***Table S7a. Nationally representative descriptive statistics for India***

| **Characteristic** | **N = 12,765**^1^ |
| --- | --- |
| **Age group** |  |
| 18-24 | 2,543 (20%) |
| 25-29 | 1,640 (13%) |
| 30-39 | 3,109 (24%) |
| 40-49 | 2,275 (18%) |
| 50-59 | 1,574 (12%) |
| 60-69 | 1,188 (9.3%) |
| 70-79 | 370 (2.9%) |
| 80 or older | 67 (0.5%) |
| Missing | 0 (0%) |
| **Gender** |  |
| Male | 6,473 (51%) |
| Female | 6,292 (49%) |
| Other | 0 (0%) |
| Missing | 0 (0%) |
| **Marital status** |  |
| Married | 9,848 (77%) |
| Separated | 45 (0.4%) |
| Divorced | 25 (0.2%) |
| Widowed | 445 (3.5%) |
| Never | 2,065 (16%) |
| Domestic Partner | 269 (2.1%) |
| Missing | 69 (0.5%) |
| **Employment** |  |
| Employed for an employer | 2,660 (21%) |
| Self-employed | 3,401 (27%) |
| Retired | 286 (2.2%) |
| Student | 532 (4.2%) |
| Homemaker | 4,221 (33%) |
| Unemployed and looking for a job | 902 (7.1%) |
| None of these/other | 715 (5.6%) |
| Missing | 48 (0.4%) |
| **Religious service attendance** |  |
| >1/week | 2,875 (23%) |
| 1/week | 3,166 (25%) |
| 1-3/month | 2,740 (21%) |
| A few times a year | 2,090 (16%) |
| Never | 1,823 (14%) |
| Missing | 71 (0.6%) |
| **Education** |  |
| up to 8 years | 11,422 (89%) |
| 9-15 years | 1,194 (9.4%) |
| 16+years | 145 (1.1%) |
| Missing | 4 (<0.1%) |
| **Immigration** |  |
| Born in this country | 12,629 (99%) |
| Born in another country | 110 (0.9%) |
| Missing | 26 (0.2%) |
| **Religious affiliation** |  |
| Christianity | 306 (2.4%) |
| Islam | 1,555 (12%) |
| Hinduism | 10,362 (81%) |
| Buddhism | 230 (1.8%) |
| Sikhism | 127 (1.0%) |
| Jainism | 10 (<0.1%) |
| Shinto | 1 (<0.1%) |
| Primal, Animist, or Folk religion | 30 (0.2%) |
| Some other religion | 67 (0.5%) |
| No religion/Atheist/Agnostic | 13 (0.1%) |
| Missing | 62 (0.5%) |
| **Race and ethnicity** |  |
| General | 3,538 (28%) |
| Other backward caste | 4,177 (33%) |
| Schedule caste | 3,599 (28%) |
| Schedule tribe | 1,185 (9.3%) |
| Missing | 267 (2.1%) |
| ^1^n (%) | |

***Table S7b. Proportions by demographic category for India***

| Variable | Category | Proportion | SE | 95% CI | Global p-value |
| --- | --- | --- | --- | --- | --- |
| Age group | 18-24 | 0.30 | 0.02 | (0.26, 0.33) | <0.001 |
|  | 25-29 | 0.30 | 0.02 | (0.27, 0.33) |  |
|  | 30-39 | 0.31 | 0.01 | (0.28, 0.34) |  |
|  | 40-49 | 0.30 | 0.01 | (0.28, 0.33) |  |
|  | 50-59 | 0.27 | 0.02 | (0.24, 0.30) |  |
|  | 60-69 | 0.25 | 0.02 | (0.22, 0.29) |  |
|  | 70-79 | 0.24 | 0.03 | (0.19, 0.29) |  |
|  | 80 or older | 0.23 | 0.06 | (0.10, 0.36) |  |
| Gender | Male | 0.32 | 0.01 | (0.30, 0.34) | <0.001 |
|  | Female | 0.26 | 0.01 | (0.24, 0.29) |  |
| Marital status | Married | 0.29 | 0.01 | (0.27, 0.31) | 0.133 |
|  | Separated | 0.29 | 0.08 | (0.12, 0.46) |  |
|  | Divorced | 0.39 | 0.13 | (0.13, 0.65) |  |
|  | Widowed | 0.27 | 0.03 | (0.22, 0.33) |  |
|  | Never | 0.30 | 0.02 | (0.27, 0.33) |  |
|  | Domestic Partner | 0.19 | 0.04 | (0.10, 0.28) |  |
| Employment | Employed for an employer | 0.30 | 0.02 | (0.27, 0.34) | <0.001 |
|  | Self-employed | 0.31 | 0.02 | (0.28, 0.34) |  |
|  | Retired | 0.20 | 0.03 | (0.15, 0.26) |  |
|  | Student | 0.32 | 0.02 | (0.27, 0.37) |  |
|  | Homemaker | 0.27 | 0.01 | (0.25, 0.30) |  |
|  | Unemployed and looking for a job | 0.30 | 0.02 | (0.26, 0.35) |  |
|  | None of these/other | 0.30 | 0.03 | (0.23, 0.37) |  |
| Religious service attendance | >1/week | 0.33 | 0.01 | (0.30, 0.36) | <0.001 |
|  | 1/week | 0.32 | 0.01 | (0.29, 0.35) |  |
|  | 1-3/month | 0.28 | 0.01 | (0.25, 0.30) |  |
|  | A few times a year | 0.28 | 0.02 | (0.25, 0.31) |  |
|  | Never | 0.22 | 0.01 | (0.19, 0.24) |  |
| Education | up to 8 years | 0.29 | 0.01 | (0.27, 0.31) | 0.892 |
|  | 9-15 years | 0.30 | 0.02 | (0.27, 0.34) |  |
|  | 16+years | 0.32 | 0.04 | (0.25, 0.40) |  |
| Immigration status | Born in this country | 0.29 | 0.01 | (0.27, 0.31) | <0.001 |
|  | Born in another country | 0.16 | 0.04 | (0.08, 0.24) |  |
| Religious affiliation | Christianity | 0.24 | 0.05 | (0.13, 0.34) | <0.001 |
|  | Islam | 0.32 | 0.03 | (0.26, 0.38) |  |
|  | Hinduism | 0.29 | 0.01 | (0.27, 0.31) |  |
|  | Buddhism | 0.31 | 0.04 | (0.23, 0.39) |  |
|  | Sikhism | 0.18 | 0.06 | (0.06, 0.29) |  |
|  | Jainism | 0.45 | 0.13 | (0.14, 0.76) |  |
|  | Primal, Animist, or Folk religion | 0.31 | 0.11 | (0.08, 0.55) |  |
|  | Some other religion | 0.19 | 0.07 | (0.04, 0.34) |  |
|  | No religion/Atheist/Agnostic | 0.07 | 0.07 | (-0.09, 0.23) |  |
| Race and ethnicity | General | 0.26 | 0.01 | (0.23, 0.29) | <0.001 |
|  | Other backward caste | 0.31 | 0.01 | (0.28, 0.33) |  |
|  | Schedule caste | 0.28 | 0.02 | (0.25, 0.31) |  |
|  | Schedule tribe | 0.37 | 0.03 | (0.31, 0.43) |  |

***Table S8a. Nationally representative descriptive statistics for Indonesia***

| **Characteristic** | **N = 6,992**^1^ |
| --- | --- |
| **Age group** |  |
| 18-24 | 1,216 (17%) |
| 25-29 | 849 (12%) |
| 30-39 | 1,591 (23%) |
| 40-49 | 1,576 (23%) |
| 50-59 | 1,169 (17%) |
| 60-69 | 490 (7.0%) |
| 70-79 | 83 (1.2%) |
| 80 or older | 17 (0.2%) |
| Missing | 0 (0%) |
| **Gender** |  |
| Male | 3,461 (50%) |
| Female | 3,513 (50%) |
| Other | 7 (<0.1%) |
| Missing | 11 (0.2%) |
| **Marital status** |  |
| Married | 4,846 (69%) |
| Separated | 81 (1.2%) |
| Divorced | 196 (2.8%) |
| Widowed | 425 (6.1%) |
| Never | 1,381 (20%) |
| Domestic Partner | 18 (0.3%) |
| Missing | 45 (0.6%) |
| **Employment** |  |
| Employed for an employer | 1,323 (19%) |
| Self-employed | 2,187 (31%) |
| Retired | 78 (1.1%) |
| Student | 272 (3.9%) |
| Homemaker | 2,138 (31%) |
| Unemployed and looking for a job | 529 (7.6%) |
| None of these/other | 448 (6.4%) |
| Missing | 18 (0.3%) |
| **Religious service attendance** |  |
| >1/week | 2,667 (38%) |
| 1/week | 2,529 (36%) |
| 1-3/month | 786 (11%) |
| A few times a year | 659 (9.4%) |
| Never | 332 (4.8%) |
| Missing | 18 (0.3%) |
| **Education** |  |
| up to 8 years | 3,079 (44%) |
| 9-15 years | 3,491 (50%) |
| 16+years | 419 (6.0%) |
| Missing | 2 (<0.1%) |
| **Immigration** |  |
| Born in this country | 6,958 (100%) |
| Born in another country | 34 (0.5%) |
| Missing | 0 (0%) |
| **Religious affiliation** |  |
| Christianity | 504 (7.2%) |
| Islam | 6,406 (92%) |
| Hinduism | 73 (1.0%) |
| Buddhism | 3 (<0.1%) |
| Taoism | 1 (<0.1%) |
| Some other religion | 1 (<0.1%) |
| Missing | 4 (<0.1%) |
| **Race and ethnicity** |  |
| Banjar/Melayu Banjar | 320 (4.6%) |
| Betawi | 251 (3.6%) |
| Bugis | 243 (3.5%) |
| Jawa | 2,846 (41%) |
| Madura | 262 (3.7%) |
| Minangkabau | 273 (3.9%) |
| Sunda/Parahyangan | 1,172 (17%) |
| Bali | 69 (1.0%) |
| Batak | 165 (2.4%) |
| Makasar | 91 (1.3%) |
| Other | 1,262 (18%) |
| Missing | 38 (0.5%) |
| ^1^n (%) | |

***Table S8b. Proportions by demographic category for Indonesia***

| Variable | Category | Proportion | SE | 95% CI | Global p-value |
| --- | --- | --- | --- | --- | --- |
| Age group | 18-24 | 0.46 | 0.02 | (0.43, 0.50) | 0.001 |
|  | 25-29 | 0.43 | 0.02 | (0.39, 0.47) |  |
|  | 30-39 | 0.43 | 0.02 | (0.40, 0.46) |  |
|  | 40-49 | 0.47 | 0.02 | (0.44, 0.50) |  |
|  | 50-59 | 0.50 | 0.02 | (0.46, 0.54) |  |
|  | 60-69 | 0.49 | 0.04 | (0.42, 0.56) |  |
|  | 70-79 | 0.36 | 0.08 | (0.20, 0.52) |  |
|  | 80 or older | 0.43 | 0.20 | (-0.01, 0.87) |  |
| Gender | Male | 0.50 | 0.01 | (0.47, 0.52) | <0.001 |
|  | Female | 0.42 | 0.01 | (0.40, 0.44) |  |
|  | Other | 0.75 | 0.19 | (0.25, 1.24) |  |
| Marital status | Married | 0.46 | 0.01 | (0.44, 0.49) | 0.388 |
|  | Separated | 0.39 | 0.07 | (0.25, 0.52) |  |
|  | Divorced | 0.49 | 0.05 | (0.40, 0.58) |  |
|  | Widowed | 0.40 | 0.03 | (0.33, 0.46) |  |
|  | Never | 0.46 | 0.02 | (0.43, 0.49) |  |
|  | Domestic Partner | 0.59 | 0.17 | (0.24, 0.94) |  |
| Employment | Employed for an employer | 0.47 | 0.02 | (0.43, 0.51) | <0.001 |
|  | Self-employed | 0.48 | 0.02 | (0.45, 0.51) |  |
|  | Retired | 0.50 | 0.07 | (0.36, 0.64) |  |
|  | Student | 0.59 | 0.03 | (0.53, 0.66) |  |
|  | Homemaker | 0.41 | 0.01 | (0.38, 0.44) |  |
|  | Unemployed and looking for a job | 0.46 | 0.03 | (0.41, 0.51) |  |
|  | None of these/other | 0.50 | 0.03 | (0.43, 0.56) |  |
| Religious service attendance | >1/week | 0.52 | 0.01 | (0.49, 0.55) | <0.001 |
|  | 1/week | 0.45 | 0.01 | (0.43, 0.48) |  |
|  | 1-3/month | 0.39 | 0.02 | (0.34, 0.44) |  |
|  | A few times a year | 0.35 | 0.02 | (0.31, 0.40) |  |
|  | Never | 0.39 | 0.04 | (0.32, 0.47) |  |
| Education | up to 8 years | 0.47 | 0.01 | (0.44, 0.50) | <0.001 |
|  | 9-15 years | 0.44 | 0.01 | (0.42, 0.46) |  |
|  | 16+years | 0.51 | 0.02 | (0.46, 0.56) |  |
| Immigration status | Born in this country | 0.46 | 0.01 | (0.44, 0.48) | 0.529 |
|  | Born in another country | 0.52 | 0.08 | (0.36, 0.69) |  |
| Religious affiliation | Christianity | 0.49 | 0.02 | (0.44, 0.53) | 0.731 |
|  | Islam | 0.46 | 0.01 | (0.44, 0.48) |  |
|  | Hinduism | 0.43 | 0.06 | (0.32, 0.54) |  |
|  | Buddhism | 0.25 | 0.26 | (-2.18, 2.68) |  |
| Race and ethnicity | Banjar/Melayu Banjar | 0.42 | 0.03 | (0.36, 0.49) | <0.001 |
|  | Betawi | 0.43 | 0.04 | (0.35, 0.52) |  |
|  | Bugis | 0.45 | 0.04 | (0.36, 0.53) |  |
|  | Jawa | 0.48 | 0.01 | (0.45, 0.50) |  |
|  | Madura | 0.55 | 0.05 | (0.46, 0.64) |  |
|  | Minangkabau | 0.48 | 0.05 | (0.37, 0.59) |  |
|  | Sunda/Parahyangan | 0.42 | 0.02 | (0.38, 0.47) |  |
|  | Bali | 0.39 | 0.07 | (0.25, 0.52) |  |
|  | Batak | 0.43 | 0.05 | (0.34, 0.53) |  |
|  | Makasar | 0.35 | 0.04 | (0.27, 0.43) |  |
|  | Other | 0.46 | 0.02 | (0.42, 0.51) |  |

***Table S9a. Nationally representative descriptive statistics for Israel***

| **Characteristic** | **N = 3,669**^1^ |
| --- | --- |
| **Age group** |  |
| 18-24 | 553 (15%) |
| 25-29 | 407 (11%) |
| 30-39 | 666 (18%) |
| 40-49 | 616 (17%) |
| 50-59 | 542 (15%) |
| 60-69 | 469 (13%) |
| 70-79 | 336 (9.2%) |
| 80 or older | 79 (2.2%) |
| Missing | 0 (0%) |
| **Gender** |  |
| Male | 1,791 (49%) |
| Female | 1,872 (51%) |
| Other | 0 (<0.1%) |
| Missing | 6 (0.2%) |
| **Marital status** |  |
| Married | 2,056 (56%) |
| Separated | 48 (1.3%) |
| Divorced | 258 (7.0%) |
| Widowed | 212 (5.8%) |
| Never | 834 (23%) |
| Domestic Partner | 193 (5.3%) |
| Missing | 69 (1.9%) |
| **Employment** |  |
| Employed for an employer | 1,793 (49%) |
| Self-employed | 424 (12%) |
| Retired | 576 (16%) |
| Student | 388 (11%) |
| Homemaker | 211 (5.7%) |
| Unemployed and looking for a job | 148 (4.0%) |
| None of these/other | 118 (3.2%) |
| Missing | 10 (0.3%) |
| **Religious service attendance** |  |
| >1/week | 649 (18%) |
| 1/week | 495 (14%) |
| 1-3/month | 374 (10%) |
| A few times a year | 1,014 (28%) |
| Never | 1,122 (31%) |
| Missing | 14 (0.4%) |
| **Education** |  |
| up to 8 years | 224 (6.1%) |
| 9-15 years | 1,517 (41%) |
| 16+years | 1,926 (52%) |
| Missing | 2 (<0.1%) |
| **Immigration** |  |
| Born in this country | 2,796 (76%) |
| Born in another country | 868 (24%) |
| Missing | 5 (0.1%) |
| **Religious affiliation** |  |
| Christianity | 39 (1.1%) |
| Islam | 656 (18%) |
| Judaism | 2,897 (79%) |
| Baha’i | 2 (<0.1%) |
| Taoism | 1 (<0.1%) |
| Primal, Animist, or Folk religion | 1 (<0.1%) |
| Some other religion | 5 (0.1%) |
| No religion/Atheist/Agnostic | 64 (1.7%) |
| Missing | 4 (0.1%) |
| **Race and ethnicity** |  |
| Arab | 674 (18%) |
| Jewish | 2,926 (80%) |
| Other | 39 (1.1%) |
| Missing | 30 (0.8%) |
| ^1^n (%) | |

***Table S9b. Proportions by demographic category for Israel***

| Variable | Category | Proportion | SE | 95% CI | Global p-value |
| --- | --- | --- | --- | --- | --- |
| Age group | 18-24 | 0.31 | 0.02 | (0.26, 0.35) | <0.001 |
|  | 25-29 | 0.22 | 0.02 | (0.17, 0.27) |  |
|  | 30-39 | 0.20 | 0.02 | (0.16, 0.24) |  |
|  | 40-49 | 0.22 | 0.02 | (0.18, 0.26) |  |
|  | 50-59 | 0.20 | 0.02 | (0.15, 0.25) |  |
|  | 60-69 | 0.15 | 0.02 | (0.10, 0.19) |  |
|  | 70-79 | 0.13 | 0.02 | (0.08, 0.17) |  |
|  | 80 or older | 0.11 | 0.04 | (0.03, 0.19) |  |
| Gender | Male | 0.21 | 0.01 | (0.18, 0.23) | 1.000 |
|  | Female | 0.21 | 0.01 | (0.18, 0.23) |  |
| Marital status | Married | 0.20 | 0.01 | (0.17, 0.23) | <0.001 |
|  | Separated | 0.26 | 0.06 | (0.14, 0.39) |  |
|  | Divorced | 0.18 | 0.03 | (0.12, 0.24) |  |
|  | Widowed | 0.11 | 0.03 | (0.07, 0.16) |  |
|  | Never | 0.25 | 0.02 | (0.22, 0.29) |  |
|  | Domestic Partner | 0.21 | 0.04 | (0.13, 0.29) |  |
| Employment | Employed for an employer | 0.21 | 0.01 | (0.19, 0.24) | <0.001 |
|  | Self-employed | 0.19 | 0.03 | (0.14, 0.25) |  |
|  | Retired | 0.12 | 0.02 | (0.08, 0.16) |  |
|  | Student | 0.29 | 0.03 | (0.23, 0.34) |  |
|  | Homemaker | 0.13 | 0.03 | (0.07, 0.19) |  |
|  | Unemployed and looking for a job | 0.27 | 0.06 | (0.15, 0.39) |  |
|  | None of these/other | 0.30 | 0.05 | (0.20, 0.39) |  |
| Religious service attendance | >1/week | 0.31 | 0.02 | (0.27, 0.35) | <0.001 |
|  | 1/week | 0.21 | 0.02 | (0.16, 0.26) |  |
|  | 1-3/month | 0.21 | 0.02 | (0.17, 0.26) |  |
|  | A few times a year | 0.20 | 0.02 | (0.16, 0.23) |  |
|  | Never | 0.15 | 0.02 | (0.12, 0.18) |  |
| Education | up to 8 years | 0.03 | 0.02 | (0.00, 0.07) | <0.001 |
|  | 9-15 years | 0.19 | 0.01 | (0.16, 0.21) |  |
|  | 16+years | 0.24 | 0.02 | (0.21, 0.27) |  |
| Immigration status | Born in this country | 0.22 | 0.01 | (0.20, 0.25) | <0.001 |
|  | Born in another country | 0.15 | 0.02 | (0.12, 0.18) |  |
| Religious affiliation | Christianity | 0.13 | 0.06 | (0.01, 0.24) | <0.001 |
|  | Islam | 0.16 | 0.03 | (0.11, 0.22) |  |
|  | Judaism | 0.22 | 0.01 | (0.19, 0.24) |  |
|  | Baha’i | 0.00 | * | * |  |
|  | Some other religion | 0.49 | 0.27 | (-0.37, 1.34) |  |
|  | No religion/Atheist/Agnostic | 0.11 | 0.04 | (0.02, 0.20) |  |
| Race and ethnicity | Arab | 0.16 | 0.03 | (0.11, 0.22) | 0.015 |
|  | Jewish | 0.22 | 0.01 | (0.19, 0.24) |  |
|  | Other | 0.16 | 0.07 | (0.01, 0.31) |  |

***Table S10a. Nationally representative descriptive statistics for Japan***

| **Characteristic** | **N = 20,543**^1^ |
| --- | --- |
| **Age group** |  |
| 18-24 | 1,589 (7.7%) |
| 25-29 | 806 (3.9%) |
| 30-39 | 2,851 (14%) |
| 40-49 | 3,363 (16%) |
| 50-59 | 3,770 (18%) |
| 60-69 | 4,118 (20%) |
| 70-79 | 3,554 (17%) |
| 80 or older | 493 (2.4%) |
| Missing | 0 (0%) |
| **Gender** |  |
| Male | 9,847 (48%) |
| Female | 10,602 (52%) |
| Other | 28 (0.1%) |
| Missing | 66 (0.3%) |
| **Marital status** |  |
| Married | 11,837 (58%) |
| Separated | 190 (0.9%) |
| Divorced | 2,126 (10%) |
| Widowed | 1,179 (5.7%) |
| Never | 5,004 (24%) |
| Domestic Partner | 144 (0.7%) |
| Missing | 64 (0.3%) |
| **Employment** |  |
| Employed for an employer | 10,853 (53%) |
| Self-employed | 1,748 (8.5%) |
| Retired | 2,535 (12%) |
| Student | 491 (2.4%) |
| Homemaker | 1,276 (6.2%) |
| Unemployed and looking for a job | 622 (3.0%) |
| None of these/other | 2,983 (15%) |
| Missing | 36 (0.2%) |
| **Religious service attendance** |  |
| >1/week | 316 (1.5%) |
| 1/week | 348 (1.7%) |
| 1-3/month | 862 (4.2%) |
| A few times a year | 3,112 (15%) |
| Never | 15,788 (77%) |
| Missing | 117 (0.6%) |
| **Education** |  |
| up to 8 years | 567 (2.8%) |
| 9-15 years | 14,893 (72%) |
| 16+years | 5,083 (25%) |
| Missing | 0 (0%) |
| **Immigration** |  |
| Born in this country | 19,548 (95%) |
| Born in another country | 158 (0.8%) |
| Missing | 837 (4.1%) |
| **Religious affiliation** |  |
| Christianity | 381 (1.9%) |
| Islam | 10 (<0.1%) |
| Hinduism | 5 (<0.1%) |
| Buddhism | 6,709 (33%) |
| Judaism | 10 (<0.1%) |
| Sikhism | 6 (<0.1%) |
| Baha’i | 2 (<0.1%) |
| Jainism | 11 (<0.1%) |
| Shinto | 469 (2.3%) |
| Taoism | 7 (<0.1%) |
| Confucianism | 17 (<0.1%) |
| Primal, Animist, or Folk religion | 19 (<0.1%) |
| Some other religion | 46 (0.2%) |
| No religion/Atheist/Agnostic | 12,497 (61%) |
| Missing | 355 (1.7%) |
| **Race and ethnicity** |  |
| Missing | 20,543 (100%) |
| ^1^n (%) | |

***Table S10b. Proportions by demographic category for Japan***

| Variable | Category | Proportion | SE | 95% CI | Global p-value |
| --- | --- | --- | --- | --- | --- |
| Age group | 18-24 | 0.10 | 0.01 | (0.08, 0.12) | <0.001 |
|  | 25-29 | 0.08 | 0.01 | (0.06, 0.11) |  |
|  | 30-39 | 0.07 | 0.01 | (0.06, 0.08) |  |
|  | 40-49 | 0.06 | 0.01 | (0.05, 0.07) |  |
|  | 50-59 | 0.06 | 0.00 | (0.05, 0.07) |  |
|  | 60-69 | 0.09 | 0.01 | (0.08, 0.10) |  |
|  | 70-79 | 0.14 | 0.01 | (0.13, 0.16) |  |
|  | 80 or older | 0.15 | 0.02 | (0.12, 0.19) |  |
| Gender | Male | 0.10 | 0.00 | (0.09, 0.11) | <0.001 |
|  | Female | 0.08 | 0.00 | (0.07, 0.08) |  |
|  | Other | 0.03 | 0.02 | (-0.02, 0.07) |  |
| Marital status | Married | 0.10 | 0.00 | (0.09, 0.10) | <0.001 |
|  | Separated | 0.15 | 0.04 | (0.08, 0.22) |  |
|  | Divorced | 0.07 | 0.01 | (0.06, 0.09) |  |
|  | Widowed | 0.15 | 0.01 | (0.12, 0.17) |  |
|  | Never | 0.06 | 0.00 | (0.06, 0.07) |  |
|  | Domestic Partner | 0.06 | 0.03 | (0.01, 0.11) |  |
| Employment | Employed for an employer | 0.07 | 0.00 | (0.07, 0.08) | <0.001 |
|  | Self-employed | 0.15 | 0.01 | (0.13, 0.17) |  |
|  | Retired | 0.12 | 0.01 | (0.11, 0.14) |  |
|  | Student | 0.11 | 0.01 | (0.08, 0.14) |  |
|  | Homemaker | 0.08 | 0.01 | (0.07, 0.10) |  |
|  | Unemployed and looking for a job | 0.03 | 0.01 | (0.02, 0.04) |  |
|  | None of these/other | 0.09 | 0.01 | (0.08, 0.10) |  |
| Religious service attendance | >1/week | 0.36 | 0.03 | (0.29, 0.42) | <0.001 |
|  | 1/week | 0.36 | 0.03 | (0.30, 0.43) |  |
|  | 1-3/month | 0.17 | 0.02 | (0.14, 0.20) |  |
|  | A few times a year | 0.14 | 0.01 | (0.13, 0.15) |  |
|  | Never | 0.06 | 0.00 | (0.06, 0.07) |  |
| Education | up to 8 years | 0.07 | 0.01 | (0.04, 0.09) | <0.001 |
|  | 9-15 years | 0.07 | 0.00 | (0.07, 0.08) |  |
|  | 16+years | 0.13 | 0.01 | (0.12, 0.14) |  |
| Immigration status | Born in this country | 0.09 | 0.00 | (0.08, 0.09) | 0.003 |
|  | Born in another country | 0.15 | 0.03 | (0.08, 0.21) |  |
| Religious affiliation | Christianity | 0.28 | 0.03 | (0.23, 0.34) | <0.001 |
|  | Islam | 0.47 | 0.20 | (0.00, 0.94) |  |
|  | Hinduism | 0.86 | 0.12 | (0.48, 1.23) |  |
|  | Buddhism | 0.13 | 0.00 | (0.12, 0.14) |  |
|  | Judaism | 0.58 | 0.26 | (-0.02, 1.18) |  |
|  | Sikhism | 0.43 | 0.21 | (-0.18, 1.05) |  |
|  | Baha’i | 0.16 | * | * |  |
|  | Jainism | 0.71 | 0.23 | (0.19, 1.24) |  |
|  | Shinto | 0.16 | 0.02 | (0.12, 0.20) |  |
|  | Taoism | 0.47 | 0.19 | (-0.01, 0.94) |  |
|  | Confucianism | 0.20 | 0.11 | (-0.03, 0.43) |  |
|  | Primal, Animist, or Folk religion | 0.19 | 0.13 | (-0.07, 0.46) |  |
|  | Some other religion | 0.13 | 0.06 | (0.02, 0.25) |  |
|  | No religion/Atheist/Agnostic | 0.06 | 0.00 | (0.05, 0.06) |  |

***Table S11a. Nationally representative descriptive statistics for Kenya***

| **Characteristic** | **N = 11,389**^1^ |
| --- | --- |
| **Age group** |  |
| 18-24 | 2,868 (25%) |
| 25-29 | 2,035 (18%) |
| 30-39 | 2,564 (23%) |
| 40-49 | 1,708 (15%) |
| 50-59 | 1,072 (9.4%) |
| 60-69 | 710 (6.2%) |
| 70-79 | 360 (3.2%) |
| 80 or older | 67 (0.6%) |
| Missing | 5 (<0.1%) |
| **Gender** |  |
| Male | 5,567 (49%) |
| Female | 5,813 (51%) |
| Other | 2 (<0.1%) |
| Missing | 7 (<0.1%) |
| **Marital status** |  |
| Married | 6,626 (58%) |
| Separated | 467 (4.1%) |
| Divorced | 111 (1.0%) |
| Widowed | 464 (4.1%) |
| Never | 3,531 (31%) |
| Domestic Partner | 146 (1.3%) |
| Missing | 43 (0.4%) |
| **Employment** |  |
| Employed for an employer | 1,467 (13%) |
| Self-employed | 3,630 (32%) |
| Retired | 319 (2.8%) |
| Student | 1,136 (10.0%) |
| Homemaker | 1,537 (13%) |
| Unemployed and looking for a job | 3,153 (28%) |
| None of these/other | 138 (1.2%) |
| Missing | 9 (<0.1%) |
| **Religious service attendance** |  |
| >1/week | 2,774 (24%) |
| 1/week | 6,063 (53%) |
| 1-3/month | 1,219 (11%) |
| A few times a year | 855 (7.5%) |
| Never | 465 (4.1%) |
| Missing | 13 (0.1%) |
| **Education** |  |
| up to 8 years | 4,485 (39%) |
| 9-15 years | 6,115 (54%) |
| 16+years | 783 (6.9%) |
| Missing | 6 (<0.1%) |
| **Immigration** |  |
| Born in this country | 11,270 (99%) |
| Born in another country | 117 (1.0%) |
| Missing | 2 (<0.1%) |
| **Religious affiliation** |  |
| Christianity | 10,334 (91%) |
| Islam | 918 (8.1%) |
| Buddhism | 1 (<0.1%) |
| Judaism | 3 (<0.1%) |
| Baha’i | 1 (<0.1%) |
| Jainism | 1 (<0.1%) |
| Confucianism | 3 (<0.1%) |
| Primal, Animist, or Folk religion | 7 (<0.1%) |
| Some other religion | 5 (<0.1%) |
| No religion/Atheist/Agnostic | 108 (0.9%) |
| Missing | 9 (<0.1%) |
| **Race and ethnicity** |  |
| Luhya | 1,943 (17%) |
| Luo | 1,120 (9.8%) |
| Kalenjin | 1,377 (12%) |
| Kamba | 1,299 (11%) |
| Kikuyu | 2,119 (19%) |
| Kisii | 789 (6.9%) |
| Maasai | 237 (2.1%) |
| Meru | 630 (5.5%) |
| Kenyan Somali/Somali | 396 (3.5%) |
| Miji Kenda tribes | 708 (6.2%) |
| Embu | 197 (1.7%) |
| Other | 548 (4.8%) |
| Missing | 27 (0.2%) |
| ^1^n (%) | |

***Table S11b. Proportions by demographic category for Kenya***

| Variable | Category | Proportion | SE | 95% CI | Global p-value |
| --- | --- | --- | --- | --- | --- |
| Age group | 18-24 | 0.36 | 0.01 | (0.34, 0.38) | <0.001 |
|  | 25-29 | 0.40 | 0.01 | (0.37, 0.42) |  |
|  | 30-39 | 0.39 | 0.01 | (0.36, 0.42) |  |
|  | 40-49 | 0.41 | 0.02 | (0.37, 0.45) |  |
|  | 50-59 | 0.46 | 0.02 | (0.42, 0.51) |  |
|  | 60-69 | 0.40 | 0.03 | (0.34, 0.46) |  |
|  | 70-79 | 0.40 | 0.04 | (0.31, 0.48) |  |
|  | 80 or older | 0.36 | 0.10 | (0.15, 0.57) |  |
| Gender | Male | 0.41 | 0.01 | (0.39, 0.44) | <0.001 |
|  | Female | 0.38 | 0.01 | (0.36, 0.40) |  |
|  | Other | 0.19 | * | * |  |
| Marital status | Married | 0.40 | 0.01 | (0.38, 0.42) | 0.600 |
|  | Separated | 0.40 | 0.03 | (0.34, 0.46) |  |
|  | Divorced | 0.44 | 0.06 | (0.32, 0.56) |  |
|  | Widowed | 0.41 | 0.04 | (0.34, 0.48) |  |
|  | Never | 0.38 | 0.01 | (0.36, 0.40) |  |
|  | Domestic Partner | 0.46 | 0.05 | (0.36, 0.57) |  |
| Employment | Employed for an employer | 0.37 | 0.02 | (0.34, 0.41) | <0.001 |
|  | Self-employed | 0.44 | 0.01 | (0.41, 0.46) |  |
|  | Retired | 0.41 | 0.04 | (0.33, 0.49) |  |
|  | Student | 0.39 | 0.02 | (0.36, 0.42) |  |
|  | Homemaker | 0.39 | 0.02 | (0.36, 0.43) |  |
|  | Unemployed and looking for a job | 0.37 | 0.01 | (0.34, 0.39) |  |
|  | None of these/other | 0.30 | 0.05 | (0.20, 0.40) |  |
| Religious service attendance | >1/week | 0.42 | 0.02 | (0.39, 0.45) | <0.001 |
|  | 1/week | 0.40 | 0.01 | (0.38, 0.42) |  |
|  | 1-3/month | 0.37 | 0.02 | (0.33, 0.40) |  |
|  | A few times a year | 0.34 | 0.02 | (0.30, 0.38) |  |
|  | Never | 0.36 | 0.03 | (0.29, 0.42) |  |
| Education | up to 8 years | 0.38 | 0.01 | (0.35, 0.41) | <0.001 |
|  | 9-15 years | 0.40 | 0.01 | (0.38, 0.42) |  |
|  | 16+years | 0.45 | 0.02 | (0.40, 0.50) |  |
| Immigration status | Born in this country | 0.40 | 0.01 | (0.38, 0.42) | 0.002 |
|  | Born in another country | 0.30 | 0.05 | (0.20, 0.40) |  |
| Religious affiliation | Christianity | 0.40 | 0.01 | (0.38, 0.42) | <0.001 |
|  | Islam | 0.38 | 0.03 | (0.31, 0.44) |  |
|  | Judaism | 0.83 | * | * |  |
|  | Primal, Animist, or Folk religion | 0.32 | 0.16 | (-0.10, 0.74) |  |
|  | Some other religion | 0.25 | 0.19 | (-0.33, 0.84) |  |
|  | No religion/Atheist/Agnostic | 0.21 | 0.05 | (0.11, 0.30) |  |
| Race and ethnicity | Luhya | 0.30 | 0.01 | (0.27, 0.33) | <0.001 |
|  | Luo | 0.43 | 0.02 | (0.39, 0.48) |  |
|  | Kalenjin | 0.45 | 0.02 | (0.41, 0.49) |  |
|  | Kamba | 0.34 | 0.03 | (0.29, 0.39) |  |
|  | Kikuyu | 0.43 | 0.02 | (0.39, 0.47) |  |
|  | Kisii | 0.35 | 0.03 | (0.29, 0.41) |  |
|  | Maasai | 0.67 | 0.05 | (0.57, 0.78) |  |
|  | Meru | 0.61 | 0.03 | (0.54, 0.67) |  |
|  | Kenyan Somali/Somali | 0.52 | 0.06 | (0.39, 0.64) |  |
|  | Miji Kenda tribes | 0.21 | 0.03 | (0.16, 0.27) |  |
|  | Embu | 0.41 | 0.05 | (0.31, 0.51) |  |
|  | Other | 0.37 | 0.03 | (0.31, 0.44) |  |

***Table S12a. Nationally representative descriptive statistics for Mexico***

| **Characteristic** | **N = 5,776**^1^ |
| --- | --- |
| **Age group** |  |
| 18-24 | 986 (17%) |
| 25-29 | 623 (11%) |
| 30-39 | 1,312 (23%) |
| 40-49 | 1,027 (18%) |
| 50-59 | 873 (15%) |
| 60-69 | 611 (11%) |
| 70-79 | 277 (4.8%) |
| 80 or older | 68 (1.2%) |
| Missing | 0 (0%) |
| **Gender** |  |
| Male | 2,755 (48%) |
| Female | 2,997 (52%) |
| Other | 3 (<0.1%) |
| Missing | 21 (0.4%) |
| **Marital status** |  |
| Married | 2,089 (36%) |
| Separated | 403 (7.0%) |
| Divorced | 230 (4.0%) |
| Widowed | 347 (6.0%) |
| Never | 1,432 (25%) |
| Domestic Partner | 1,109 (19%) |
| Missing | 166 (2.9%) |
| **Employment** |  |
| Employed for an employer | 1,921 (33%) |
| Self-employed | 1,091 (19%) |
| Retired | 386 (6.7%) |
| Student | 247 (4.3%) |
| Homemaker | 1,257 (22%) |
| Unemployed and looking for a job | 564 (9.8%) |
| None of these/other | 169 (2.9%) |
| Missing | 141 (2.4%) |
| **Religious service attendance** |  |
| >1/week | 609 (11%) |
| 1/week | 1,261 (22%) |
| 1-3/month | 676 (12%) |
| A few times a year | 2,054 (36%) |
| Never | 1,134 (20%) |
| Missing | 43 (0.7%) |
| **Education** |  |
| up to 8 years | 1,291 (22%) |
| 9-15 years | 3,180 (55%) |
| 16+years | 1,304 (23%) |
| Missing | 1 (<0.1%) |
| **Immigration** |  |
| Born in this country | 5,517 (96%) |
| Born in another country | 108 (1.9%) |
| Missing | 151 (2.6%) |
| **Religious affiliation** |  |
| Christianity | 4,844 (84%) |
| Islam | 2 (<0.1%) |
| Hinduism | 3 (<0.1%) |
| Buddhism | 6 (0.1%) |
| Judaism | 7 (0.1%) |
| Baha’i | 1 (<0.1%) |
| Jainism | 1 (<0.1%) |
| Shinto | 2 (<0.1%) |
| Taoism | 4 (<0.1%) |
| Confucianism | 1 (<0.1%) |
| Primal, Animist, or Folk religion | 20 (0.3%) |
| Some other religion | 41 (0.7%) |
| No religion/Atheist/Agnostic | 770 (13%) |
| Missing | 75 (1.3%) |
| **Race and ethnicity** |  |
| Black | 108 (1.9%) |
| Indigenous | 594 (10%) |
| White | 1,116 (19%) |
| Mestizo | 2,762 (48%) |
| Mulatto | 63 (1.1%) |
| Other | 339 (5.9%) |
| Missing | 794 (14%) |
| ^1^n (%) | |

***Table S12b. Proportions by demographic category for Mexico***

| Variable | Category | Proportion | SE | 95% CI | Global p-value |
| --- | --- | --- | --- | --- | --- |
| Age group | 18-24 | 0.21 | 0.02 | (0.18, 0.24) | 0.386 |
|  | 25-29 | 0.20 | 0.02 | (0.16, 0.24) |  |
|  | 30-39 | 0.20 | 0.01 | (0.18, 0.23) |  |
|  | 40-49 | 0.24 | 0.02 | (0.20, 0.27) |  |
|  | 50-59 | 0.21 | 0.02 | (0.17, 0.24) |  |
|  | 60-69 | 0.25 | 0.02 | (0.20, 0.30) |  |
|  | 70-79 | 0.19 | 0.03 | (0.12, 0.26) |  |
|  | 80 or older | 0.13 | 0.06 | (0.00, 0.25) |  |
| Gender | Male | 0.25 | 0.01 | (0.23, 0.27) | <0.001 |
|  | Female | 0.18 | 0.01 | (0.17, 0.20) |  |
|  | Other | 0.21 | 0.18 | (-0.84, 1.26) |  |
| Marital status | Married | 0.25 | 0.01 | (0.22, 0.27) | <0.001 |
|  | Separated | 0.17 | 0.02 | (0.12, 0.21) |  |
|  | Divorced | 0.19 | 0.03 | (0.13, 0.25) |  |
|  | Widowed | 0.22 | 0.03 | (0.16, 0.28) |  |
|  | Never | 0.21 | 0.01 | (0.18, 0.24) |  |
|  | Domestic Partner | 0.18 | 0.01 | (0.15, 0.21) |  |
| Employment | Employed for an employer | 0.23 | 0.01 | (0.20, 0.25) | <0.001 |
|  | Self-employed | 0.29 | 0.02 | (0.26, 0.33) |  |
|  | Retired | 0.18 | 0.03 | (0.13, 0.24) |  |
|  | Student | 0.24 | 0.03 | (0.17, 0.31) |  |
|  | Homemaker | 0.15 | 0.01 | (0.12, 0.17) |  |
|  | Unemployed and looking for a job | 0.19 | 0.02 | (0.15, 0.23) |  |
|  | None of these/other | 0.17 | 0.04 | (0.10, 0.24) |  |
| Religious service attendance | >1/week | 0.39 | 0.03 | (0.33, 0.44) | <0.001 |
|  | 1/week | 0.25 | 0.02 | (0.22, 0.28) |  |
|  | 1-3/month | 0.25 | 0.02 | (0.21, 0.29) |  |
|  | A few times a year | 0.17 | 0.01 | (0.15, 0.19) |  |
|  | Never | 0.15 | 0.01 | (0.12, 0.17) |  |
| Education | up to 8 years | 0.23 | 0.01 | (0.20, 0.25) | 0.103 |
|  | 9-15 years | 0.20 | 0.01 | (0.19, 0.22) |  |
|  | 16+years | 0.23 | 0.02 | (0.20, 0.26) |  |
| Immigration status | Born in this country | 0.21 | 0.01 | (0.20, 0.23) | 0.037 |
|  | Born in another country | 0.29 | 0.05 | (0.18, 0.40) |  |
| Religious affiliation | Christianity | 0.22 | 0.01 | (0.20, 0.23) | <0.001 |
|  | Islam | 0.00 | * | * |  |
|  | Hinduism | 0.80 | 0.19 | (-1.37, 2.97) |  |
|  | Buddhism | 0.10 | 0.09 | (-0.16, 0.36) |  |
|  | Judaism | 0.34 | 0.20 | (-0.21, 0.89) |  |
|  | Jainism | 0.00 | * | * |  |
|  | Shinto | 0.00 | * | * |  |
|  | Taoism | 1.00 | 0.00 | (1.00, 1.00) |  |
|  | Primal, Animist, or Folk religion | 0.11 | 0.07 | (-0.03, 0.25) |  |
|  | Some other religion | 0.28 | 0.08 | (0.13, 0.44) |  |
|  | No religion/Atheist/Agnostic | 0.19 | 0.02 | (0.16, 0.23) |  |
| Race and ethnicity | Black | 0.26 | 0.05 | (0.16, 0.37) | 0.124 |
|  | Indigenous | 0.23 | 0.02 | (0.19, 0.27) |  |
|  | White | 0.22 | 0.02 | (0.19, 0.25) |  |
|  | Mestizo | 0.20 | 0.01 | (0.18, 0.22) |  |
|  | Mulatto | 0.18 | 0.05 | (0.08, 0.27) |  |
|  | Other | 0.26 | 0.03 | (0.20, 0.32) |  |

***Table S13a. Nationally representative descriptive statistics for Nigeria***

| **Characteristic** | **N = 6,827**^1^ |
| --- | --- |
| **Age group** |  |
| 18-24 | 1,533 (22%) |
| 25-29 | 1,193 (17%) |
| 30-39 | 1,943 (28%) |
| 40-49 | 1,059 (16%) |
| 50-59 | 619 (9.1%) |
| 60-69 | 296 (4.3%) |
| 70-79 | 133 (2.0%) |
| 80 or older | 50 (0.7%) |
| Missing | 0 (0%) |
| **Gender** |  |
| Male | 3,371 (49%) |
| Female | 3,456 (51%) |
| Other | 0 (<0.1%) |
| Missing | 0 (0%) |
| **Marital status** |  |
| Married | 4,065 (60%) |
| Separated | 117 (1.7%) |
| Divorced | 71 (1.0%) |
| Widowed | 231 (3.4%) |
| Never | 2,289 (34%) |
| Domestic Partner | 12 (0.2%) |
| Missing | 42 (0.6%) |
| **Employment** |  |
| Employed for an employer | 699 (10%) |
| Self-employed | 3,898 (57%) |
| Retired | 178 (2.6%) |
| Student | 650 (9.5%) |
| Homemaker | 499 (7.3%) |
| Unemployed and looking for a job | 684 (10%) |
| None of these/other | 211 (3.1%) |
| Missing | 8 (0.1%) |
| **Religious service attendance** |  |
| >1/week | 4,049 (59%) |
| 1/week | 1,895 (28%) |
| 1-3/month | 531 (7.8%) |
| A few times a year | 254 (3.7%) |
| Never | 77 (1.1%) |
| Missing | 20 (0.3%) |
| **Education** |  |
| up to 8 years | 2,575 (38%) |
| 9-15 years | 4,120 (60%) |
| 16+years | 130 (1.9%) |
| Missing | 2 (<0.1%) |
| **Immigration** |  |
| Born in this country | 6,779 (99%) |
| Born in another country | 47 (0.7%) |
| Missing | 1 (<0.1%) |
| **Religious affiliation** |  |
| Christianity | 3,476 (51%) |
| Islam | 3,302 (48%) |
| Shinto | 1 (<0.1%) |
| Confucianism | 0 (<0.1%) |
| Primal, Animist, or Folk religion | 24 (0.3%) |
| Some other religion | 1 (<0.1%) |
| No religion/Atheist/Agnostic | 15 (0.2%) |
| Missing | 9 (0.1%) |
| **Race and ethnicity** |  |
| Hausa | 2,342 (34%) |
| Yoruba | 1,230 (18%) |
| Igbo (Ibo) | 1,111 (16%) |
| Edo | 116 (1.7%) |
| Urhobo | 38 (0.6%) |
| Fulani | 266 (3.9%) |
| Kanuri | 31 (0.5%) |
| Tiv | 198 (2.9%) |
| Efik | 48 (0.7%) |
| Ijaw | 110 (1.6%) |
| Igala | 77 (1.1%) |
| Ibibio | 180 (2.6%) |
| Idoma | 61 (0.9%) |
| Other | 1,014 (15%) |
| Missing | 4 (<0.1%) |
| ^1^n (%) | |

***Table S13b. Proportions by demographic category for Nigeria***

| Variable | Category | Proportion | SE | 95% CI | Global p-value |
| --- | --- | --- | --- | --- | --- |
| Age group | 18-24 | 0.43 | 0.02 | (0.39, 0.47) | <0.001 |
|  | 25-29 | 0.50 | 0.02 | (0.46, 0.54) |  |
|  | 30-39 | 0.53 | 0.02 | (0.49, 0.57) |  |
|  | 40-49 | 0.55 | 0.03 | (0.50, 0.61) |  |
|  | 50-59 | 0.57 | 0.04 | (0.49, 0.65) |  |
|  | 60-69 | 0.52 | 0.06 | (0.40, 0.64) |  |
|  | 70-79 | 0.46 | 0.08 | (0.30, 0.63) |  |
|  | 80 or older | 0.34 | 0.17 | (0.01, 0.68) |  |
| Gender | Male | 0.58 | 0.02 | (0.55, 0.61) | <0.001 |
|  | Female | 0.43 | 0.02 | (0.40, 0.47) |  |
| Marital status | Married | 0.52 | 0.02 | (0.49, 0.55) | 0.013 |
|  | Separated | 0.39 | 0.06 | (0.26, 0.52) |  |
|  | Divorced | 0.59 | 0.09 | (0.42, 0.76) |  |
|  | Widowed | 0.44 | 0.08 | (0.28, 0.59) |  |
|  | Never | 0.49 | 0.02 | (0.46, 0.53) |  |
|  | Domestic Partner | 0.80 | 0.16 | (0.43, 1.17) |  |
| Employment | Employed for an employer | 0.56 | 0.02 | (0.51, 0.61) | <0.001 |
|  | Self-employed | 0.52 | 0.02 | (0.49, 0.55) |  |
|  | Retired | 0.54 | 0.07 | (0.41, 0.67) |  |
|  | Student | 0.47 | 0.03 | (0.41, 0.53) |  |
|  | Homemaker | 0.36 | 0.04 | (0.27, 0.45) |  |
|  | Unemployed and looking for a job | 0.51 | 0.03 | (0.44, 0.57) |  |
|  | None of these/other | 0.44 | 0.05 | (0.33, 0.54) |  |
| Religious service attendance | >1/week | 0.51 | 0.02 | (0.48, 0.55) | 0.915 |
|  | 1/week | 0.50 | 0.02 | (0.46, 0.53) |  |
|  | 1-3/month | 0.50 | 0.04 | (0.43, 0.57) |  |
|  | A few times a year | 0.45 | 0.04 | (0.37, 0.54) |  |
|  | Never | 0.46 | 0.08 | (0.30, 0.63) |  |
| Education | up to 8 years | 0.47 | 0.02 | (0.43, 0.52) | <0.001 |
|  | 9-15 years | 0.52 | 0.01 | (0.49, 0.55) |  |
|  | 16+years | 0.65 | 0.04 | (0.58, 0.73) |  |
| Immigration status | Born in this country | 0.51 | 0.01 | (0.48, 0.53) | 0.068 |
|  | Born in another country | 0.35 | 0.11 | (0.12, 0.58) |  |
| Religious affiliation | Christianity | 0.52 | 0.02 | (0.49, 0.56) | <0.001 |
|  | Islam | 0.48 | 0.02 | (0.45, 0.52) |  |
|  | Primal, Animist, or Folk religion | 0.76 | 0.12 | (0.51, 1.02) |  |
|  | No religion/Atheist/Agnostic | 0.60 | 0.23 | (0.10, 1.10) |  |
| Race and ethnicity | Hausa | 0.49 | 0.02 | (0.45, 0.54) | <0.001 |
|  | Yoruba | 0.48 | 0.04 | (0.41, 0.55) |  |
|  | Igbo (Ibo) | 0.58 | 0.03 | (0.52, 0.64) |  |
|  | Edo | 0.47 | 0.06 | (0.35, 0.58) |  |
|  | Urhobo | 0.62 | 0.12 | (0.38, 0.85) |  |
|  | Fulani | 0.35 | 0.04 | (0.27, 0.44) |  |
|  | Kanuri | 0.43 | 0.16 | (0.09, 0.76) |  |
|  | Tiv | 0.43 | 0.05 | (0.33, 0.53) |  |
|  | Efik | 0.35 | 0.09 | (0.17, 0.54) |  |
|  | Ijaw | 0.58 | 0.04 | (0.51, 0.65) |  |
|  | Igala | 0.39 | 0.06 | (0.28, 0.51) |  |
|  | Ibibio | 0.60 | 0.06 | (0.47, 0.72) |  |
|  | Idoma | 0.34 | 0.07 | (0.19, 0.48) |  |
|  | Other | 0.54 | 0.03 | (0.47, 0.61) |  |

***Table S14a. Nationally representative descriptive statistics for Philippines***

| **Characteristic** | **N = 5,292**^1^ |
| --- | --- |
| **Age group** |  |
| 18-24 | 1,073 (20%) |
| 25-29 | 695 (13%) |
| 30-39 | 1,160 (22%) |
| 40-49 | 972 (18%) |
| 50-59 | 732 (14%) |
| 60-69 | 495 (9.4%) |
| 70-79 | 143 (2.7%) |
| 80 or older | 23 (0.4%) |
| Missing | 0 (0%) |
| **Gender** |  |
| Male | 2,625 (50%) |
| Female | 2,643 (50%) |
| Other | 13 (0.2%) |
| Missing | 11 (0.2%) |
| **Marital status** |  |
| Married | 2,385 (45%) |
| Separated | 249 (4.7%) |
| Divorced | 9 (0.2%) |
| Widowed | 274 (5.2%) |
| Never | 1,206 (23%) |
| Domestic Partner | 1,152 (22%) |
| Missing | 16 (0.3%) |
| **Employment** |  |
| Employed for an employer | 1,350 (26%) |
| Self-employed | 1,379 (26%) |
| Retired | 158 (3.0%) |
| Student | 585 (11%) |
| Homemaker | 1,049 (20%) |
| Unemployed and looking for a job | 658 (12%) |
| None of these/other | 113 (2.1%) |
| Missing | 0 (0%) |
| **Religious service attendance** |  |
| >1/week | 844 (16%) |
| 1/week | 1,929 (36%) |
| 1-3/month | 1,374 (26%) |
| A few times a year | 929 (18%) |
| Never | 210 (4.0%) |
| Missing | 6 (0.1%) |
| **Education** |  |
| up to 8 years | 1,188 (22%) |
| 9-15 years | 3,722 (70%) |
| 16+years | 381 (7.2%) |
| Missing | 1 (<0.1%) |
| **Immigration** |  |
| Born in this country | 5,284 (100%) |
| Born in another country | 8 (0.1%) |
| Missing | 0 (0%) |
| **Religious affiliation** |  |
| Christianity | 4,914 (93%) |
| Islam | 297 (5.6%) |
| Buddhism | 4 (<0.1%) |
| Judaism | 4 (<0.1%) |
| Baha’i | 1 (<0.1%) |
| Primal, Animist, or Folk religion | 5 (<0.1%) |
| Some other religion | 35 (0.7%) |
| No religion/Atheist/Agnostic | 23 (0.4%) |
| Missing | 9 (0.2%) |
| **Race and ethnicity** |  |
| Tagalog | 1,691 (32%) |
| Cebuano | 656 (12%) |
| Ilocano/Ilokano | 429 (8.1%) |
| Visayan/Bisaya | 739 (14%) |
| Ilonggo/Hiligaynon | 428 (8.1%) |
| Bicolano/Bikolano | 300 (5.7%) |
| Waray | 216 (4.1%) |
| Tausug | 94 (1.8%) |
| Maranao | 39 (0.7%) |
| Maguindanaoan | 84 (1.6%) |
| Chinese-Filipino | 3 (<0.1%) |
| Kapampangan | 107 (2.0%) |
| Pangasinense | 107 (2.0%) |
| Zamboangueno | 51 (1.0%) |
| Masbateno | 54 (1.0%) |
| Aeta | 1 (<0.1%) |
| Igorot | 42 (0.8%) |
| Mangyan | 2 (<0.1%) |
| Badjao | 2 (<0.1%) |
| Other | 244 (4.6%) |
| Missing | 3 (<0.1%) |
| ^1^n (%) | |

***Table S14b. Proportions by demographic category for Philippines***

| Variable | Category | Proportion | SE | 95% CI | Global p-value |
| --- | --- | --- | --- | --- | --- |
| Age group | 18-24 | 0.23 | 0.02 | (0.19, 0.26) | 0.152 |
|  | 25-29 | 0.23 | 0.02 | (0.19, 0.28) |  |
|  | 30-39 | 0.25 | 0.02 | (0.22, 0.28) |  |
|  | 40-49 | 0.27 | 0.02 | (0.24, 0.31) |  |
|  | 50-59 | 0.26 | 0.02 | (0.23, 0.30) |  |
|  | 60-69 | 0.27 | 0.03 | (0.22, 0.33) |  |
|  | 70-79 | 0.40 | 0.06 | (0.28, 0.53) |  |
|  | 80 or older | 0.35 | 0.12 | (0.11, 0.59) |  |
| Gender | Male | 0.28 | 0.01 | (0.25, 0.30) | <0.001 |
|  | Female | 0.23 | 0.01 | (0.21, 0.25) |  |
|  | Other | 0.50 | 0.13 | (0.21, 0.80) |  |
| Marital status | Married | 0.27 | 0.01 | (0.24, 0.29) | <0.001 |
|  | Separated | 0.27 | 0.03 | (0.20, 0.34) |  |
|  | Divorced | 0.34 | 0.15 | (-0.01, 0.69) |  |
|  | Widowed | 0.32 | 0.03 | (0.25, 0.38) |  |
|  | Never | 0.26 | 0.02 | (0.23, 0.29) |  |
|  | Domestic Partner | 0.21 | 0.01 | (0.18, 0.24) |  |
| Employment | Employed for an employer | 0.26 | 0.02 | (0.22, 0.29) | <0.001 |
|  | Self-employed | 0.30 | 0.02 | (0.27, 0.34) |  |
|  | Retired | 0.33 | 0.06 | (0.22, 0.45) |  |
|  | Student | 0.30 | 0.03 | (0.25, 0.35) |  |
|  | Homemaker | 0.18 | 0.01 | (0.15, 0.20) |  |
|  | Unemployed and looking for a job | 0.23 | 0.02 | (0.18, 0.27) |  |
|  | None of these/other | 0.19 | 0.04 | (0.11, 0.27) |  |
| Religious service attendance | >1/week | 0.31 | 0.02 | (0.27, 0.35) | <0.001 |
|  | 1/week | 0.29 | 0.01 | (0.26, 0.31) |  |
|  | 1-3/month | 0.23 | 0.01 | (0.20, 0.26) |  |
|  | A few times a year | 0.19 | 0.02 | (0.16, 0.22) |  |
|  | Never | 0.21 | 0.04 | (0.13, 0.28) |  |
| Education | up to 8 years | 0.23 | 0.02 | (0.19, 0.26) | 0.024 |
|  | 9-15 years | 0.26 | 0.01 | (0.24, 0.28) |  |
|  | 16+years | 0.28 | 0.03 | (0.22, 0.35) |  |
| Immigration status | Born in this country | 0.26 | 0.01 | (0.24, 0.27) | 0.936 |
|  | Born in another country | 0.18 | 0.16 | (-0.23, 0.59) |  |
| Religious affiliation | Christianity | 0.26 | 0.01 | (0.24, 0.27) | <0.001 |
|  | Islam | 0.23 | 0.03 | (0.18, 0.28) |  |
|  | Buddhism | 0.89 | 0.12 | (0.41, 1.36) |  |
|  | Judaism | 0.83 | 0.00 | (0.83, 0.83) |  |
|  | Primal, Animist, or Folk religion | 0.22 | 0.18 | (-0.36, 0.80) |  |
|  | Some other religion | 0.26 | 0.06 | (0.13, 0.39) |  |
|  | No religion/Atheist/Agnostic | 0.20 | 0.10 | (-0.01, 0.41) |  |
| Race and ethnicity | Tagalog | 0.22 | 0.01 | (0.19, 0.24) | *** |
|  | Cebuano | 0.34 | 0.03 | (0.29, 0.39) |  |
|  | Ilocano/Ilokano | 0.28 | 0.02 | (0.23, 0.32) |  |
|  | Visayan/Bisaya | 0.31 | 0.03 | (0.25, 0.36) |  |
|  | Ilonggo/Hiligaynon | 0.20 | 0.03 | (0.15, 0.25) |  |
|  | Bicolano/Bikolano | 0.24 | 0.03 | (0.18, 0.31) |  |
|  | Waray | 0.21 | 0.03 | (0.14, 0.27) |  |
|  | Tausug | 0.23 | 0.03 | (0.16, 0.29) |  |
|  | Maranao | 0.24 | 0.06 | (0.13, 0.35) |  |
|  | Maguindanaoan | 0.24 | 0.04 | (0.16, 0.32) |  |
|  | Chinese-Filipino | 0.00 | * | * |  |
|  | Kapampangan | 0.26 | 0.05 | (0.15, 0.36) |  |
|  | Pangasinense | 0.21 | 0.06 | (0.09, 0.33) |  |
|  | Zamboangueno | 0.28 | 0.08 | (0.12, 0.43) |  |
|  | Masbateno | 0.18 | 0.06 | (0.06, 0.29) |  |
|  | Aeta | 0.73 | * | * |  |
|  | Igorot | 0.36 | 0.11 | (0.13, 0.60) |  |
|  | Mangyan | 0.00 | * | * |  |
|  | Badjao | 0.00 | * | * |  |
|  | Other | 0.29 | 0.04 | (0.21, 0.37) |  |

***p-value could not be computed due to sparsity of the race and ethnicity variable.

***Table S15a. Nationally representative descriptive statistics for Poland***

| **Characteristic** | **N = 10,389**^1^ |
| --- | --- |
| **Age group** |  |
| 18-24 | 955 (9.2%) |
| 25-29 | 761 (7.3%) |
| 30-39 | 2,159 (21%) |
| 40-49 | 1,956 (19%) |
| 50-59 | 1,670 (16%) |
| 60-69 | 1,909 (18%) |
| 70-79 | 833 (8.0%) |
| 80 or older | 145 (1.4%) |
| Missing | 1 (<0.1%) |
| **Gender** |  |
| Male | 4,974 (48%) |
| Female | 5,387 (52%) |
| Other | 3 (<0.1%) |
| Missing | 26 (0.2%) |
| **Marital status** |  |
| Married | 6,065 (58%) |
| Separated | 111 (1.1%) |
| Divorced | 529 (5.1%) |
| Widowed | 990 (9.5%) |
| Never | 1,811 (17%) |
| Domestic Partner | 504 (4.8%) |
| Missing | 379 (3.6%) |
| **Employment** |  |
| Employed for an employer | 5,837 (56%) |
| Self-employed | 686 (6.6%) |
| Retired | 2,434 (23%) |
| Student | 515 (5.0%) |
| Homemaker | 338 (3.3%) |
| Unemployed and looking for a job | 284 (2.7%) |
| None of these/other | 169 (1.6%) |
| Missing | 126 (1.2%) |
| **Religious service attendance** |  |
| >1/week | 305 (2.9%) |
| 1/week | 3,263 (31%) |
| 1-3/month | 2,081 (20%) |
| A few times a year | 3,064 (29%) |
| Never | 1,597 (15%) |
| Missing | 78 (0.8%) |
| **Education** |  |
| up to 8 years | 1,238 (12%) |
| 9-15 years | 6,130 (59%) |
| 16+years | 3,020 (29%) |
| Missing | 1 (<0.1%) |
| **Immigration** |  |
| Born in this country | 10,258 (99%) |
| Born in another country | 108 (1.0%) |
| Missing | 23 (0.2%) |
| **Religious affiliation** |  |
| Christianity | 9,378 (90%) |
| Islam | 2 (<0.1%) |
| Buddhism | 2 (<0.1%) |
| Sikhism | 1 (<0.1%) |
| Jainism | 3 (<0.1%) |
| Shinto | 1 (<0.1%) |
| Primal, Animist, or Folk religion | 11 (0.1%) |
| No religion/Atheist/Agnostic | 942 (9.1%) |
| Missing | 50 (0.5%) |
| **Race and ethnicity** |  |
| Polish | 10,309 (99%) |
| German | 4 (<0.1%) |
| Belarussian | 2 (<0.1%) |
| Ukrainian | 38 (0.4%) |
| Silesia | 14 (0.1%) |
| Kashubians | 3 (<0.1%) |
| Other | 4 (<0.1%) |
| Missing | 14 (0.1%) |
| ^1^n (%) | |

***Table S15b. Proportions by demographic category for Poland***

| Variable | Category | Proportion | SE | 95% CI | Global p-value |
| --- | --- | --- | --- | --- | --- |
| Age group | 18-24 | 0.08 | 0.01 | (0.06, 0.11) | 0.004 |
|  | 25-29 | 0.07 | 0.01 | (0.05, 0.09) |  |
|  | 30-39 | 0.07 | 0.01 | (0.06, 0.09) |  |
|  | 40-49 | 0.08 | 0.01 | (0.07, 0.10) |  |
|  | 50-59 | 0.07 | 0.01 | (0.05, 0.09) |  |
|  | 60-69 | 0.11 | 0.01 | (0.09, 0.14) |  |
|  | 70-79 | 0.08 | 0.02 | (0.05, 0.12) |  |
|  | 80 or older | 0.09 | 0.06 | (-0.02, 0.20) |  |
| Gender | Male | 0.07 | 0.01 | (0.06, 0.08) | <0.001 |
|  | Female | 0.10 | 0.01 | (0.08, 0.11) |  |
|  | Other | 0.00 | * | * |  |
| Marital status | Married | 0.07 | 0.01 | (0.06, 0.09) | <0.001 |
|  | Separated | 0.07 | 0.03 | (0.02, 0.12) |  |
|  | Divorced | 0.07 | 0.01 | (0.04, 0.09) |  |
|  | Widowed | 0.13 | 0.02 | (0.09, 0.17) |  |
|  | Never | 0.09 | 0.01 | (0.07, 0.11) |  |
|  | Domestic Partner | 0.08 | 0.01 | (0.05, 0.11) |  |
| Employment | Employed for an employer | 0.07 | 0.01 | (0.06, 0.08) | <0.001 |
|  | Self-employed | 0.11 | 0.02 | (0.07, 0.14) |  |
|  | Retired | 0.11 | 0.01 | (0.08, 0.13) |  |
|  | Student | 0.08 | 0.02 | (0.05, 0.11) |  |
|  | Homemaker | 0.06 | 0.01 | (0.03, 0.09) |  |
|  | Unemployed and looking for a job | 0.06 | 0.02 | (0.03, 0.09) |  |
|  | None of these/other | 0.11 | 0.04 | (0.04, 0.18) |  |
| Religious service attendance | >1/week | 0.11 | 0.03 | (0.04, 0.18) | 0.983 |
|  | 1/week | 0.08 | 0.01 | (0.06, 0.09) |  |
|  | 1-3/month | 0.09 | 0.01 | (0.07, 0.11) |  |
|  | A few times a year | 0.09 | 0.01 | (0.07, 0.10) |  |
|  | Never | 0.08 | 0.01 | (0.06, 0.10) |  |
| Education | up to 8 years | 0.08 | 0.02 | (0.03, 0.13) | <0.001 |
|  | 9-15 years | 0.07 | 0.01 | (0.06, 0.08) |  |
|  | 16+years | 0.11 | 0.01 | (0.09, 0.12) |  |
| Immigration status | Born in this country | 0.08 | 0.01 | (0.07, 0.09) | <0.001 |
|  | Born in another country | 0.22 | 0.05 | (0.11, 0.33) |  |
| Religious affiliation | Christianity | 0.08 | 0.01 | (0.07, 0.09) | <0.001 |
|  | Islam | 0.00 | * | * |  |
|  | Buddhism | 0.70 | * | * |  |
|  | Primal, Animist, or Folk religion | 0.15 | 0.10 | (-0.08, 0.38) |  |
|  | No religion/Atheist/Agnostic | 0.10 | 0.01 | (0.07, 0.12) |  |
| Race and ethnicity | Polish | 0.08 | 0.01 | (0.07, 0.09) | 0.078 |
|  | German | 0.28 | 0.25 | (-1.62, 2.18) |  |
|  | Belarussian | 0.00 | * | * |  |
|  | Ukrainian | 0.13 | 0.05 | (0.02, 0.23) |  |
|  | Silesia | 0.00 | * | * |  |
|  | Kashubians | 0.18 | 0.18 | (-1.35, 1.71) |  |
|  | Other | 0.45 | 0.24 | (-0.47, 1.37) |  |

***Table S16a. Nationally representative descriptive statistics for South Africa***

| **Characteristic** | **N = 2,651**^1^ |
| --- | --- |
| **Age group** |  |
| 18-24 | 461 (17%) |
| 25-29 | 364 (14%) |
| 30-39 | 655 (25%) |
| 40-49 | 522 (20%) |
| 50-59 | 309 (12%) |
| 60-69 | 195 (7.4%) |
| 70-79 | 120 (4.5%) |
| 80 or older | 17 (0.6%) |
| Missing | 9 (0.3%) |
| **Gender** |  |
| Male | 1,288 (49%) |
| Female | 1,356 (51%) |
| Other | 2 (<0.1%) |
| Missing | 4 (0.2%) |
| **Marital status** |  |
| Married | 539 (20%) |
| Separated | 76 (2.9%) |
| Divorced | 51 (1.9%) |
| Widowed | 133 (5.0%) |
| Never | 1,561 (59%) |
| Domestic Partner | 264 (10.0%) |
| Missing | 28 (1.0%) |
| **Employment** |  |
| Employed for an employer | 569 (21%) |
| Self-employed | 412 (16%) |
| Retired | 243 (9.2%) |
| Student | 204 (7.7%) |
| Homemaker | 137 (5.2%) |
| Unemployed and looking for a job | 1,008 (38%) |
| None of these/other | 74 (2.8%) |
| Missing | 3 (0.1%) |
| **Religious service attendance** |  |
| >1/week | 414 (16%) |
| 1/week | 891 (34%) |
| 1-3/month | 574 (22%) |
| A few times a year | 431 (16%) |
| Never | 334 (13%) |
| Missing | 7 (0.3%) |
| **Education** |  |
| up to 8 years | 668 (25%) |
| 9-15 years | 1,796 (68%) |
| 16+years | 183 (6.9%) |
| Missing | 4 (0.2%) |
| **Immigration** |  |
| Born in this country | 2,511 (95%) |
| Born in another country | 139 (5.2%) |
| Missing | 1 (<0.1%) |
| **Religious affiliation** |  |
| Christianity | 2,163 (82%) |
| Islam | 62 (2.3%) |
| Hinduism | 1 (<0.1%) |
| Buddhism | 12 (0.5%) |
| Jainism | 2 (<0.1%) |
| Shinto | 2 (<0.1%) |
| Taoism | 1 (<0.1%) |
| Primal, Animist, or Folk religion | 127 (4.8%) |
| Some other religion | 5 (0.2%) |
| No religion/Atheist/Agnostic | 253 (9.6%) |
| Missing | 23 (0.9%) |
| **Race and ethnicity** |  |
| Black | 2,381 (90%) |
| White | 8 (0.3%) |
| Asian/Indian | 6 (0.2%) |
| Colored | 252 (9.5%) |
| Other | 1 (<0.1%) |
| Missing | 3 (0.1%) |
| ^1^n (%) | |

***Table S16b. Proportions by demographic category for South Africa***

| Variable | Category | Proportion | SE | 95% CI | Global p-value |
| --- | --- | --- | --- | --- | --- |
| Age group | 18-24 | 0.25 | 0.02 | (0.20, 0.29) | <0.001 |
|  | 25-29 | 0.25 | 0.02 | (0.20, 0.30) |  |
|  | 30-39 | 0.24 | 0.02 | (0.20, 0.28) |  |
|  | 40-49 | 0.25 | 0.02 | (0.20, 0.30) |  |
|  | 50-59 | 0.27 | 0.04 | (0.20, 0.35) |  |
|  | 60-69 | 0.23 | 0.05 | (0.13, 0.33) |  |
|  | 70-79 | 0.26 | 0.08 | (0.11, 0.41) |  |
|  | 80 or older | 0.02 | 0.03 | (-0.03, 0.08) |  |
| Gender | Male | 0.27 | 0.02 | (0.23, 0.30) | 0.133 |
|  | Female | 0.23 | 0.02 | (0.20, 0.26) |  |
|  | Other | 0.42 | * | * |  |
| Marital status | Married | 0.25 | 0.03 | (0.20, 0.31) | 0.702 |
|  | Separated | 0.16 | 0.05 | (0.07, 0.26) |  |
|  | Divorced | 0.32 | 0.09 | (0.14, 0.50) |  |
|  | Widowed | 0.23 | 0.06 | (0.11, 0.35) |  |
|  | Never | 0.25 | 0.01 | (0.22, 0.28) |  |
|  | Domestic Partner | 0.24 | 0.03 | (0.18, 0.31) |  |
| Employment | Employed for an employer | 0.20 | 0.02 | (0.16, 0.24) | 0.003 |
|  | Self-employed | 0.30 | 0.03 | (0.24, 0.36) |  |
|  | Retired | 0.22 | 0.05 | (0.13, 0.32) |  |
|  | Student | 0.21 | 0.03 | (0.15, 0.27) |  |
|  | Homemaker | 0.24 | 0.05 | (0.14, 0.33) |  |
|  | Unemployed and looking for a job | 0.27 | 0.02 | (0.24, 0.30) |  |
|  | None of these/other | 0.25 | 0.08 | (0.09, 0.41) |  |
| Religious service attendance | >1/week | 0.27 | 0.03 | (0.20, 0.33) | 0.003 |
|  | 1/week | 0.27 | 0.02 | (0.23, 0.31) |  |
|  | 1-3/month | 0.25 | 0.02 | (0.21, 0.29) |  |
|  | A few times a year | 0.24 | 0.02 | (0.19, 0.29) |  |
|  | Never | 0.18 | 0.03 | (0.12, 0.23) |  |
| Education | up to 8 years | 0.22 | 0.03 | (0.16, 0.28) | <0.001 |
|  | 9-15 years | 0.25 | 0.01 | (0.22, 0.27) |  |
|  | 16+years | 0.37 | 0.04 | (0.30, 0.44) |  |
| Immigration status | Born in this country | 0.25 | 0.01 | (0.23, 0.28) | <0.001 |
|  | Born in another country | 0.12 | 0.04 | (0.04, 0.19) |  |
| Religious affiliation | Christianity | 0.26 | 0.01 | (0.23, 0.28) | <0.001 |
|  | Islam | 0.17 | 0.04 | (0.08, 0.26) |  |
|  | Hinduism | 1.00 | * | * |  |
|  | Buddhism | 0.44 | 0.21 | (-0.04, 0.91) |  |
|  | Shinto | 0.30 | * | * |  |
|  | Primal, Animist, or Folk religion | 0.20 | 0.05 | (0.10, 0.30) |  |
|  | Some other religion | 0.23 | 0.20 | (-0.40, 0.85) |  |
|  | No religion/Atheist/Agnostic | 0.18 | 0.03 | (0.13, 0.24) |  |
| Race and ethnicity | Black | 0.25 | 0.01 | (0.23, 0.27) | 0.560 |
|  | White | 0.41 | 0.10 | (0.16, 0.67) |  |
|  | Asian/Indian | 0.14 | 0.18 | (-0.37, 0.66) |  |
|  | Colored | 0.24 | 0.06 | (0.13, 0.35) |  |

***Table S17a. Nationally representative descriptive statistics for Spain***

| **Characteristic** | **N = 6,290**^1^ |
| --- | --- |
| **Age group** |  |
| 18-24 | 594 (9.4%) |
| 25-29 | 450 (7.2%) |
| 30-39 | 1,111 (18%) |
| 40-49 | 1,396 (22%) |
| 50-59 | 1,252 (20%) |
| 60-69 | 977 (16%) |
| 70-79 | 467 (7.4%) |
| 80 or older | 43 (0.7%) |
| Missing | 0 (0%) |
| **Gender** |  |
| Male | 3,142 (50%) |
| Female | 3,119 (50%) |
| Other | 6 (0.1%) |
| Missing | 22 (0.4%) |
| **Marital status** |  |
| Married | 2,947 (47%) |
| Separated | 237 (3.8%) |
| Divorced | 518 (8.2%) |
| Widowed | 189 (3.0%) |
| Never | 1,742 (28%) |
| Domestic Partner | 589 (9.4%) |
| Missing | 67 (1.1%) |
| **Employment** |  |
| Employed for an employer | 2,862 (45%) |
| Self-employed | 576 (9.2%) |
| Retired | 1,278 (20%) |
| Student | 448 (7.1%) |
| Homemaker | 345 (5.5%) |
| Unemployed and looking for a job | 646 (10%) |
| None of these/other | 123 (2.0%) |
| Missing | 11 (0.2%) |
| **Religious service attendance** |  |
| >1/week | 317 (5.0%) |
| 1/week | 662 (11%) |
| 1-3/month | 437 (6.9%) |
| A few times a year | 1,972 (31%) |
| Never | 2,875 (46%) |
| Missing | 27 (0.4%) |
| **Education** |  |
| up to 8 years | 802 (13%) |
| 9-15 years | 4,145 (66%) |
| 16+years | 1,341 (21%) |
| Missing | 2 (<0.1%) |
| **Immigration** |  |
| Born in this country | 5,479 (87%) |
| Born in another country | 788 (13%) |
| Missing | 23 (0.4%) |
| **Religious affiliation** |  |
| Christianity | 4,074 (65%) |
| Islam | 135 (2.1%) |
| Hinduism | 7 (0.1%) |
| Buddhism | 36 (0.6%) |
| Judaism | 4 (<0.1%) |
| Sikhism | 3 (<0.1%) |
| Baha’i | 2 (<0.1%) |
| Jainism | 1 (<0.1%) |
| Taoism | 5 (<0.1%) |
| Confucianism | 3 (<0.1%) |
| Primal, Animist, or Folk religion | 7 (0.1%) |
| Some other religion | 27 (0.4%) |
| No religion/Atheist/Agnostic | 1,932 (31%) |
| Missing | 55 (0.9%) |
| **Race and ethnicity** |  |
| Missing | 6,290 (100%) |
| ^1^n (%) | |

***Table S17b. Proportions by demographic category for Spain***

| Variable | Category | Proportion | SE | 95% CI | Global p-value |
| --- | --- | --- | --- | --- | --- |
| Age group | 18-24 | 0.19 | 0.02 | (0.15, 0.22) | 0.001 |
|  | 25-29 | 0.22 | 0.02 | (0.17, 0.26) |  |
|  | 30-39 | 0.20 | 0.01 | (0.17, 0.23) |  |
|  | 40-49 | 0.17 | 0.01 | (0.14, 0.19) |  |
|  | 50-59 | 0.17 | 0.01 | (0.14, 0.20) |  |
|  | 60-69 | 0.14 | 0.02 | (0.10, 0.18) |  |
|  | 70-79 | 0.16 | 0.03 | (0.10, 0.22) |  |
|  | 80 or older | 0.09 | 0.06 | (-0.03, 0.21) |  |
| Gender | Male | 0.22 | 0.01 | (0.20, 0.24) | <0.001 |
|  | Female | 0.12 | 0.01 | (0.11, 0.14) |  |
|  | Other | 0.26 | 0.15 | (-0.15, 0.68) |  |
| Marital status | Married | 0.17 | 0.01 | (0.15, 0.19) | 1.000 |
|  | Separated | 0.21 | 0.04 | (0.14, 0.28) |  |
|  | Divorced | 0.17 | 0.02 | (0.13, 0.22) |  |
|  | Widowed | 0.18 | 0.05 | (0.09, 0.27) |  |
|  | Never | 0.17 | 0.01 | (0.15, 0.20) |  |
|  | Domestic Partner | 0.18 | 0.02 | (0.14, 0.21) |  |
| Employment | Employed for an employer | 0.17 | 0.01 | (0.16, 0.19) | 0.286 |
|  | Self-employed | 0.21 | 0.02 | (0.17, 0.25) |  |
|  | Retired | 0.18 | 0.02 | (0.14, 0.22) |  |
|  | Student | 0.18 | 0.02 | (0.14, 0.22) |  |
|  | Homemaker | 0.14 | 0.03 | (0.09, 0.20) |  |
|  | Unemployed and looking for a job | 0.15 | 0.02 | (0.12, 0.18) |  |
|  | None of these/other | 0.20 | 0.04 | (0.13, 0.28) |  |
| Religious service attendance | >1/week | 0.31 | 0.03 | (0.24, 0.37) | <0.001 |
|  | 1/week | 0.32 | 0.03 | (0.27, 0.37) |  |
|  | 1-3/month | 0.23 | 0.03 | (0.18, 0.28) |  |
|  | A few times a year | 0.15 | 0.01 | (0.13, 0.18) |  |
|  | Never | 0.13 | 0.01 | (0.12, 0.15) |  |
| Education | up to 8 years | 0.16 | 0.02 | (0.12, 0.20) | <0.001 |
|  | 9-15 years | 0.17 | 0.01 | (0.15, 0.18) |  |
|  | 16+years | 0.20 | 0.01 | (0.18, 0.23) |  |
| Immigration status | Born in this country | 0.17 | 0.01 | (0.16, 0.18) | 0.081 |
|  | Born in another country | 0.19 | 0.02 | (0.16, 0.23) |  |
| Religious affiliation | Christianity | 0.20 | 0.01 | (0.18, 0.22) | <0.001 |
|  | Islam | 0.24 | 0.05 | (0.15, 0.34) |  |
|  | Hinduism | 0.28 | 0.15 | (-0.12, 0.69) |  |
|  | Buddhism | 0.33 | 0.12 | (0.07, 0.58) |  |
|  | Judaism | 0.61 | 0.23 | (-0.27, 1.49) |  |
|  | Sikhism | 0.00 |  |  |  |
|  | Baha’i | 0.40 |  |  |  |
|  | Taoism | 0.16 | 0.16 | (-0.42, 0.73) |  |
|  | Confucianism | 0.00 |  |  |  |
|  | Primal, Animist, or Folk religion | 0.12 | 0.08 | (-0.09, 0.34) |  |
|  | Some other religion | 0.23 | 0.08 | (0.06, 0.41) |  |
|  | No religion/Atheist/Agnostic | 0.11 | 0.01 | (0.09, 0.13) |  |

***Table S18a. Nationally representative descriptive statistics for Sweden***

| **Characteristic** | **N = 15,068**^1^ |
| --- | --- |
| **Age group** |  |
| 18-24 | 1,515 (10%) |
| 25-29 | 1,399 (9.3%) |
| 30-39 | 2,398 (16%) |
| 40-49 | 2,221 (15%) |
| 50-59 | 2,493 (17%) |
| 60-69 | 2,168 (14%) |
| 70-79 | 2,253 (15%) |
| 80 or older | 621 (4.1%) |
| Missing | 0 (0%) |
| **Gender** |  |
| Male | 7,536 (50%) |
| Female | 7,493 (50%) |
| Other | 27 (0.2%) |
| Missing | 12 (<0.1%) |
| **Marital status** |  |
| Married | 6,408 (43%) |
| Separated | 426 (2.8%) |
| Divorced | 801 (5.3%) |
| Widowed | 433 (2.9%) |
| Never | 3,854 (26%) |
| Domestic Partner | 3,073 (20%) |
| Missing | 72 (0.5%) |
| **Employment** |  |
| Employed for an employer | 7,907 (52%) |
| Self-employed | 1,243 (8.3%) |
| Retired | 3,832 (25%) |
| Student | 1,332 (8.8%) |
| Homemaker | 75 (0.5%) |
| Unemployed and looking for a job | 324 (2.2%) |
| None of these/other | 337 (2.2%) |
| Missing | 18 (0.1%) |
| **Religious service attendance** |  |
| >1/week | 236 (1.6%) |
| 1/week | 434 (2.9%) |
| 1-3/month | 486 (3.2%) |
| A few times a year | 3,950 (26%) |
| Never | 9,918 (66%) |
| Missing | 45 (0.3%) |
| **Education** |  |
| up to 8 years | 252 (1.7%) |
| 9-15 years | 10,790 (72%) |
| 16+years | 4,026 (27%) |
| Missing | 0 (0%) |
| **Immigration** |  |
| Born in this country | 13,922 (92%) |
| Born in another country | 1,052 (7.0%) |
| Missing | 94 (0.6%) |
| **Religious affiliation** |  |
| Christianity | 8,346 (55%) |
| Islam | 470 (3.1%) |
| Hinduism | 22 (0.1%) |
| Buddhism | 110 (0.7%) |
| Judaism | 54 (0.4%) |
| Sikhism | 4 (<0.1%) |
| Baha’i | 6 (<0.1%) |
| Shinto | 0 (<0.1%) |
| Taoism | 4 (<0.1%) |
| Primal, Animist, or Folk religion | 83 (0.5%) |
| Some other religion | 198 (1.3%) |
| No religion/Atheist/Agnostic | 5,697 (38%) |
| Missing | 74 (0.5%) |
| **Race and ethnicity** |  |
| Missing | 15,068 (100%) |
| ^1^n (%) | |

***Table S18b. Proportions by demographic category for Sweden***

| Variable | Category | Proportion | SE | 95% CI | Global p-value |
| --- | --- | --- | --- | --- | --- |
| Age group | 18-24 | 0.13 | 0.01 | (0.11, 0.16) | <0.001 |
|  | 25-29 | 0.11 | 0.01 | (0.09, 0.13) |  |
|  | 30-39 | 0.09 | 0.01 | (0.08, 0.11) |  |
|  | 40-49 | 0.12 | 0.01 | (0.10, 0.14) |  |
|  | 50-59 | 0.12 | 0.01 | (0.10, 0.14) |  |
|  | 60-69 | 0.09 | 0.01 | (0.08, 0.11) |  |
|  | 70-79 | 0.13 | 0.01 | (0.11, 0.15) |  |
|  | 80 or older | 0.14 | 0.02 | (0.10, 0.18) |  |
| Gender | Male | 0.11 | 0.00 | (0.10, 0.12) | 0.034 |
|  | Female | 0.12 | 0.01 | (0.11, 0.13) |  |
|  | Other | 0.25 | 0.09 | (0.07, 0.43) |  |
| Marital status | Married | 0.12 | 0.01 | (0.11, 0.13) | <0.001 |
|  | Separated | 0.14 | 0.02 | (0.09, 0.18) |  |
|  | Divorced | 0.13 | 0.02 | (0.10, 0.17) |  |
|  | Widowed | 0.19 | 0.02 | (0.14, 0.24) |  |
|  | Never | 0.12 | 0.01 | (0.10, 0.13) |  |
|  | Domestic Partner | 0.08 | 0.01 | (0.07, 0.10) |  |
| Employment | Employed for an employer | 0.10 | 0.00 | (0.09, 0.11) | <0.001 |
|  | Self-employed | 0.14 | 0.02 | (0.11, 0.17) |  |
|  | Retired | 0.13 | 0.01 | (0.11, 0.14) |  |
|  | Student | 0.13 | 0.01 | (0.11, 0.16) |  |
|  | Homemaker | 0.17 | 0.06 | (0.06, 0.28) |  |
|  | Unemployed and looking for a job | 0.13 | 0.02 | (0.08, 0.17) |  |
|  | None of these/other | 0.12 | 0.02 | (0.08, 0.17) |  |
| Religious service attendance | >1/week | 0.54 | 0.05 | (0.45, 0.63) | <0.001 |
|  | 1/week | 0.43 | 0.03 | (0.36, 0.49) |  |
|  | 1-3/month | 0.32 | 0.03 | (0.27, 0.38) |  |
|  | A few times a year | 0.14 | 0.01 | (0.13, 0.16) |  |
|  | Never | 0.07 | 0.00 | (0.06, 0.08) |  |
| Education | up to 8 years | 0.08 | 0.02 | (0.03, 0.12) | <0.001 |
|  | 9-15 years | 0.10 | 0.00 | (0.09, 0.11) |  |
|  | 16+years | 0.15 | 0.01 | (0.14, 0.17) |  |
| Immigration status | Born in this country | 0.11 | 0.00 | (0.10, 0.12) | <0.001 |
|  | Born in another country | 0.14 | 0.01 | (0.11, 0.17) |  |
| Religious affiliation | Christianity | 0.14 | 0.01 | (0.12, 0.15) | *** |
|  | Islam | 0.13 | 0.02 | (0.08, 0.18) |  |
|  | Hinduism | 0.00 | * | * |  |
|  | Buddhism | 0.14 | 0.05 | (0.03, 0.24) |  |
|  | Judaism | 0.00 | * | * |  |
|  | Sikhism | 0.00 | * | * |  |
|  | Baha’i | 1.00 | * | * |  |
|  | Taoism | 0.00 | * | * |  |
|  | Primal, Animist, or Folk religion | 0.10 | 0.05 | (-0.01, 0.21) |  |
|  | Some other religion | 0.07 | 0.03 | (0.01, 0.13) |  |
|  | No religion/Atheist/Agnostic | 0.08 | 0.00 | (0.07, 0.09) |  |

***p-value could not be computed due to sparsity of the religious affiliation variable.

***Table S19a. Nationally representative descriptive statistics for Tanzania***

| **Characteristic** | **N = 9,075**^1^ |
| --- | --- |
| **Age group** |  |
| 18-24 | 2,284 (25%) |
| 25-29 | 1,349 (15%) |
| 30-39 | 2,060 (23%) |
| 40-49 | 1,503 (17%) |
| 50-59 | 912 (10%) |
| 60-69 | 575 (6.3%) |
| 70-79 | 297 (3.3%) |
| 80 or older | 93 (1.0%) |
| Missing | 2 (<0.1%) |
| **Gender** |  |
| Male | 4,299 (47%) |
| Female | 4,776 (53%) |
| Other | 0 (0%) |
| Missing | 0 (0%) |
| **Marital status** |  |
| Married | 5,577 (61%) |
| Separated | 404 (4.5%) |
| Divorced | 103 (1.1%) |
| Widowed | 450 (5.0%) |
| Never | 2,260 (25%) |
| Domestic Partner | 275 (3.0%) |
| Missing | 7 (<0.1%) |
| **Employment** |  |
| Employed for an employer | 513 (5.6%) |
| Self-employed | 4,625 (51%) |
| Retired | 139 (1.5%) |
| Student | 319 (3.5%) |
| Homemaker | 1,796 (20%) |
| Unemployed and looking for a job | 1,491 (16%) |
| None of these/other | 186 (2.1%) |
| Missing | 6 (<0.1%) |
| **Religious service attendance** |  |
| >1/week | 2,622 (29%) |
| 1/week | 4,268 (47%) |
| 1-3/month | 1,082 (12%) |
| A few times a year | 814 (9.0%) |
| Never | 288 (3.2%) |
| Missing | 1 (<0.1%) |
| **Education** |  |
| up to 8 years | 6,699 (74%) |
| 9-15 years | 2,252 (25%) |
| 16+years | 122 (1.3%) |
| Missing | 2 (<0.1%) |
| **Immigration** |  |
| Born in this country | 9,048 (100%) |
| Born in another country | 25 (0.3%) |
| Missing | 1 (<0.1%) |
| **Religious affiliation** |  |
| Christianity | 5,647 (62%) |
| Islam | 3,189 (35%) |
| Taoism | 1 (<0.1%) |
| Primal, Animist, or Folk religion | 12 (0.1%) |
| No religion/Atheist/Agnostic | 216 (2.4%) |
| Missing | 10 (0.1%) |
| **Race and ethnicity** |  |
| Indian | 3 (<0.1%) |
| Arab | 11 (0.1%) |
| African | 9,060 (100%) |
| Missing | 2 (<0.1%) |
| ^1^n (%) | |

***Table S19b. Proportions by demographic category for Tanzania***

| Variable | Category | Proportion | SE | 95% CI | Global p-value |
| --- | --- | --- | --- | --- | --- |
| Age group | 18-24 | 0.09 | 0.01 | (0.07, 0.11) | <0.001 |
|  | 25-29 | 0.11 | 0.01 | (0.09, 0.13) |  |
|  | 30-39 | 0.12 | 0.01 | (0.10, 0.14) |  |
|  | 40-49 | 0.11 | 0.01 | (0.09, 0.13) |  |
|  | 50-59 | 0.12 | 0.01 | (0.10, 0.15) |  |
|  | 60-69 | 0.16 | 0.02 | (0.12, 0.21) |  |
|  | 70-79 | 0.07 | 0.02 | (0.03, 0.11) |  |
|  | 80 or older | 0.09 | 0.04 | (0.02, 0.16) |  |
| Gender | Male | 0.14 | 0.01 | (0.12, 0.15) | <0.001 |
|  | Female | 0.09 | 0.01 | (0.07, 0.10) |  |
| Marital status | Married | 0.11 | 0.01 | (0.10, 0.13) | 0.597 |
|  | Separated | 0.09 | 0.01 | (0.06, 0.12) |  |
|  | Divorced | 0.07 | 0.02 | (0.02, 0.12) |  |
|  | Widowed | 0.10 | 0.02 | (0.06, 0.14) |  |
|  | Never | 0.11 | 0.01 | (0.09, 0.13) |  |
|  | Domestic Partner | 0.11 | 0.02 | (0.07, 0.15) |  |
| Employment | Employed for an employer | 0.19 | 0.02 | (0.14, 0.24) | <0.001 |
|  | Self-employed | 0.12 | 0.01 | (0.10, 0.14) |  |
|  | Retired | 0.15 | 0.04 | (0.07, 0.22) |  |
|  | Student | 0.11 | 0.02 | (0.08, 0.15) |  |
|  | Homemaker | 0.07 | 0.01 | (0.05, 0.08) |  |
|  | Unemployed and looking for a job | 0.11 | 0.01 | (0.09, 0.13) |  |
|  | None of these/other | 0.06 | 0.02 | (0.03, 0.10) |  |
| Religious service attendance | >1/week | 0.14 | 0.01 | (0.12, 0.16) | <0.001 |
|  | 1/week | 0.10 | 0.01 | (0.09, 0.12) |  |
|  | 1-3/month | 0.09 | 0.01 | (0.07, 0.12) |  |
|  | A few times a year | 0.08 | 0.01 | (0.05, 0.10) |  |
|  | Never | 0.07 | 0.02 | (0.03, 0.10) |  |
| Education | up to 8 years | 0.10 | 0.01 | (0.09, 0.11) | <0.001 |
|  | 9-15 years | 0.13 | 0.01 | (0.11, 0.15) |  |
|  | 16+years | 0.27 | 0.05 | (0.17, 0.36) |  |
| Immigration status | Born in this country | 0.11 | 0.01 | (0.10, 0.12) | 0.948 |
|  | Born in another country | 0.14 | 0.07 | (0.00, 0.28) |  |
| Religious affiliation | Christianity | 0.12 | 0.01 | (0.11, 0.14) | <0.001 |
|  | Islam | 0.09 | 0.01 | (0.07, 0.10) |  |
|  | Primal, Animist, or Folk religion | 0.22 | 0.18 | (-0.19, 0.62) |  |
|  | No religion/Atheist/Agnostic | 0.05 | 0.02 | (0.02, 0.08) |  |
| Race and ethnicity | Indian | 0.00 | * | * | <0.001 |
|  | Arab | 0.00 | * | * |  |
|  | African | 0.11 | 0.01 | (0.10, 0.12) |  |

***Table S20a. Nationally representative descriptive statistics for Turkey***

| **Characteristic** | **N = 1,473**^1^ |
| --- | --- |
| **Age group** |  |
| 18-24 | 222 (15%) |
| 25-29 | 152 (10%) |
| 30-39 | 315 (21%) |
| 40-49 | 312 (21%) |
| 50-59 | 225 (15%) |
| 60-69 | 164 (11%) |
| 70-79 | 65 (4.4%) |
| 80 or older | 18 (1.2%) |
| Missing | 0 (0%) |
| **Gender** |  |
| Male | 754 (51%) |
| Female | 719 (49%) |
| Other | 0 (0%) |
| Missing | 0 (0%) |
| **Marital status** |  |
| Married | 936 (64%) |
| Separated | 13 (0.9%) |
| Divorced | 64 (4.3%) |
| Widowed | 64 (4.3%) |
| Never | 379 (26%) |
| Domestic Partner | 0 (0%) |
| Missing | 17 (1.1%) |
| **Employment** |  |
| Employed for an employer | 413 (28%) |
| Self-employed | 255 (17%) |
| Retired | 205 (14%) |
| Student | 107 (7.3%) |
| Homemaker | 347 (24%) |
| Unemployed and looking for a job | 87 (5.9%) |
| None of these/other | 59 (4.0%) |
| Missing | 0 (0%) |
| **Religious service attendance** |  |
| >1/week | 493 (33%) |
| 1/week | 271 (18%) |
| 1-3/month | 174 (12%) |
| A few times a year | 255 (17%) |
| Never | 274 (19%) |
| Missing | 6 (0.4%) |
| **Education** |  |
| up to 8 years | 436 (30%) |
| 9-15 years | 711 (48%) |
| 16+years | 326 (22%) |
| Missing | 0 (0%) |
| **Immigration** |  |
| Born in this country | 1,415 (96%) |
| Born in another country | 58 (4.0%) |
| Missing | 0 (0%) |
| **Religious affiliation** |  |
| Christianity | 2 (0.1%) |
| Islam | 1,381 (94%) |
| Buddhism | 1 (<0.1%) |
| Judaism | 1 (<0.1%) |
| Sikhism | 1 (<0.1%) |
| Primal, Animist, or Folk religion | 1 (<0.1%) |
| Some other religion | 1 (<0.1%) |
| No religion/Atheist/Agnostic | 66 (4.5%) |
| Missing | 19 (1.3%) |
| **Race and ethnicity** |  |
| Arab | 51 (3.5%) |
| Turkish | 1,030 (70%) |
| Greek | 1 (<0.1%) |
| Kurdish/Zaza | 252 (17%) |
| Laz | 25 (1.7%) |
| Circassian | 19 (1.3%) |
| Bosnian | 5 (0.3%) |
| Armenian | 1 (<0.1%) |
| Georgian | 4 (0.3%) |
| Uyghur | 1 (<0.1%) |
| Albanian | 8 (0.5%) |
| Azeri | 9 (0.6%) |
| Other | 58 (3.9%) |
| Missing | 9 (0.6%) |
| ^1^n (%) | |

***Table S20b. Proportions by demographic category for Turkey***

| Variable | Category | Proportion | SE | 95% CI | Global p-value |
| --- | --- | --- | --- | --- | --- |
| Age group | 18-24 | 0.19 | 0.03 | (0.14, 0.24) | <0.001 |
|  | 25-29 | 0.18 | 0.03 | (0.12, 0.25) |  |
|  | 30-39 | 0.16 | 0.02 | (0.12, 0.20) |  |
|  | 40-49 | 0.14 | 0.02 | (0.10, 0.18) |  |
|  | 50-59 | 0.17 | 0.03 | (0.10, 0.24) |  |
|  | 60-69 | 0.07 | 0.03 | (0.02, 0.12) |  |
|  | 70-79 | 0.05 | 0.04 | (-0.03, 0.13) |  |
|  | 80 or older | 0.07 | 0.07 | (-0.08, 0.21) |  |
| Gender | Male | 0.16 | 0.01 | (0.14, 0.19) | 0.026 |
|  | Female | 0.13 | 0.02 | (0.10, 0.16) |  |
| Marital status | Married | 0.12 | 0.01 | (0.09, 0.14) | <0.001 |
|  | Separated | 0.21 | 0.11 | (-0.04, 0.46) |  |
|  | Divorced | 0.15 | 0.05 | (0.05, 0.25) |  |
|  | Widowed | 0.10 | 0.05 | (0.00, 0.21) |  |
|  | Never | 0.23 | 0.02 | (0.19, 0.28) |  |
| Employment | Employed for an employer | 0.21 | 0.02 | (0.16, 0.25) | <0.001 |
|  | Self-employed | 0.20 | 0.02 | (0.15, 0.25) |  |
|  | Retired | 0.06 | 0.02 | (0.03, 0.10) |  |
|  | Student | 0.22 | 0.04 | (0.15, 0.29) |  |
|  | Homemaker | 0.07 | 0.02 | (0.03, 0.11) |  |
|  | Unemployed and looking for a job | 0.17 | 0.04 | (0.09, 0.25) |  |
|  | None of these/other | 0.11 | 0.05 | (0.02, 0.20) |  |
| Religious service attendance | >1/week | 0.14 | 0.02 | (0.11, 0.18) | 0.908 |
|  | 1/week | 0.14 | 0.03 | (0.09, 0.20) |  |
|  | 1-3/month | 0.13 | 0.02 | (0.08, 0.18) |  |
|  | A few times a year | 0.18 | 0.02 | (0.13, 0.23) |  |
|  | Never | 0.15 | 0.02 | (0.11, 0.20) |  |
| Education | up to 8 years | 0.07 | 0.02 | (0.03, 0.11) | <0.001 |
|  | 9-15 years | 0.14 | 0.01 | (0.12, 0.17) |  |
|  | 16+years | 0.26 | 0.02 | (0.22, 0.30) |  |
| Immigration status | Born in this country | 0.14 | 0.01 | (0.12, 0.16) | 0.016 |
|  | Born in another country | 0.26 | 0.07 | (0.12, 0.40) |  |
| Religious affiliation | Christianity | 0.25 | * | * | <0.001 |
|  | Islam | 0.14 | 0.01 | (0.12, 0.16) |  |
|  | Sikhism | 0.00 | * | * |  |
|  | Primal, Animist, or Folk religion | 0.00 | * | * |  |
|  | Some other religion | 0.04 | * | * |  |
|  | No religion/Atheist/Agnostic | 0.27 | 0.05 | (0.17, 0.36) |  |
| Race and ethnicity | Arab | 0.16 | 0.06 | (0.04, 0.27) | 0.662 |
|  | Turkish | 0.15 | 0.01 | (0.12, 0.17) |  |
|  | Greek | 0.00 | * | * |  |
|  | Kurdish/Zaza | 0.14 | 0.02 | (0.09, 0.18) |  |
|  | Laz | 0.22 | 0.09 | (0.04, 0.41) |  |
|  | Circassian | 0.05 | 0.03 | (-0.03, 0.12) |  |
|  | Bosnian | 0.24 | 0.17 | (-0.34, 0.82) |  |
|  | Armenian | 0.00 | * | * |  |
|  | Georgian | 0.00 | * | * |  |
|  | Albanian | 0.19 | 0.17 | (-0.25, 0.63) |  |
|  | Azeri | 0.21 | 0.12 | (-0.08, 0.49) |  |
|  | Other | 0.25 | 0.08 | (0.10, 0.40) |  |

***Table S21a. Nationally representative descriptive statistics for United Kingdom***

| **Characteristic** | **N = 5,368**^1^ |
| --- | --- |
| **Age group** |  |
| 18-24 | 490 (9.1%) |
| 25-29 | 391 (7.3%) |
| 30-39 | 946 (18%) |
| 40-49 | 827 (15%) |
| 50-59 | 949 (18%) |
| 60-69 | 889 (17%) |
| 70-79 | 711 (13%) |
| 80 or older | 163 (3.0%) |
| Missing | 1 (<0.1%) |
| **Gender** |  |
| Male | 2,557 (48%) |
| Female | 2,789 (52%) |
| Other | 14 (0.3%) |
| Missing | 9 (0.2%) |
| **Marital status** |  |
| Married | 2,510 (47%) |
| Separated | 114 (2.1%) |
| Divorced | 435 (8.1%) |
| Widowed | 294 (5.5%) |
| Never | 1,456 (27%) |
| Domestic Partner | 512 (9.5%) |
| Missing | 48 (0.9%) |
| **Employment** |  |
| Employed for an employer | 2,798 (52%) |
| Self-employed | 469 (8.7%) |
| Retired | 1,262 (24%) |
| Student | 229 (4.3%) |
| Homemaker | 184 (3.4%) |
| Unemployed and looking for a job | 215 (4.0%) |
| None of these/other | 201 (3.7%) |
| Missing | 11 (0.2%) |
| **Religious service attendance** |  |
| >1/week | 291 (5.4%) |
| 1/week | 499 (9.3%) |
| 1-3/month | 293 (5.5%) |
| A few times a year | 1,165 (22%) |
| Never | 3,110 (58%) |
| Missing | 10 (0.2%) |
| **Education** |  |
| up to 8 years | 1,314 (24%) |
| 9-15 years | 2,072 (39%) |
| 16+years | 1,974 (37%) |
| Missing | 8 (0.2%) |
| **Immigration** |  |
| Born in this country | 4,659 (87%) |
| Born in another country | 682 (13%) |
| Missing | 27 (0.5%) |
| **Religious affiliation** |  |
| Christianity | 2,750 (51%) |
| Islam | 218 (4.1%) |
| Hinduism | 61 (1.1%) |
| Buddhism | 30 (0.6%) |
| Judaism | 44 (0.8%) |
| Sikhism | 29 (0.5%) |
| Baha’i | 6 (0.1%) |
| Jainism | 4 (<0.1%) |
| Taoism | 4 (<0.1%) |
| Confucianism | 2 (<0.1%) |
| Primal, Animist, or Folk religion | 36 (0.7%) |
| Some other religion | 61 (1.1%) |
| No religion/Atheist/Agnostic | 2,099 (39%) |
| Missing | 25 (0.5%) |
| **Race and ethnicity** |  |
| Asian | 426 (7.9%) |
| Black | 152 (2.8%) |
| White | 4,647 (87%) |
| Other | 96 (1.8%) |
| Missing | 47 (0.9%) |
| ^1^n (%) | |

***Table S21b. Proportions by demographic category for United Kingdom***

| Variable | Category | Proportion | SE | 95% CI | Global p-value |
| --- | --- | --- | --- | --- | --- |
| Age group | 18-24 | 0.37 | 0.04 | (0.30, 0.44) | <0.001 |
|  | 25-29 | 0.36 | 0.03 | (0.29, 0.42) |  |
|  | 30-39 | 0.33 | 0.02 | (0.28, 0.37) |  |
|  | 40-49 | 0.31 | 0.02 | (0.26, 0.35) |  |
|  | 50-59 | 0.29 | 0.02 | (0.25, 0.33) |  |
|  | 60-69 | 0.22 | 0.02 | (0.19, 0.26) |  |
|  | 70-79 | 0.27 | 0.02 | (0.23, 0.31) |  |
|  | 80 or older | 0.26 | 0.04 | (0.17, 0.34) |  |
| Gender | Male | 0.32 | 0.01 | (0.29, 0.34) | <0.001 |
|  | Female | 0.28 | 0.01 | (0.26, 0.30) |  |
|  | Other | 0.00 | * | * |  |
| Marital status | Married | 0.32 | 0.01 | (0.29, 0.34) | 0.001 |
|  | Separated | 0.27 | 0.05 | (0.17, 0.37) |  |
|  | Divorced | 0.25 | 0.03 | (0.19, 0.30) |  |
|  | Widowed | 0.35 | 0.04 | (0.28, 0.42) |  |
|  | Never | 0.28 | 0.02 | (0.25, 0.32) |  |
|  | Domestic Partner | 0.25 | 0.03 | (0.20, 0.31) |  |
| Employment | Employed for an employer | 0.33 | 0.01 | (0.30, 0.35) | <0.001 |
|  | Self-employed | 0.36 | 0.03 | (0.30, 0.42) |  |
|  | Retired | 0.25 | 0.02 | (0.22, 0.28) |  |
|  | Student | 0.26 | 0.04 | (0.17, 0.34) |  |
|  | Homemaker | 0.25 | 0.05 | (0.15, 0.36) |  |
|  | Unemployed and looking for a job | 0.23 | 0.04 | (0.14, 0.31) |  |
|  | None of these/other | 0.15 | 0.03 | (0.09, 0.20) |  |
| Religious service attendance | >1/week | 0.54 | 0.04 | (0.46, 0.62) | <0.001 |
|  | 1/week | 0.48 | 0.03 | (0.42, 0.55) |  |
|  | 1-3/month | 0.42 | 0.04 | (0.34, 0.50) |  |
|  | A few times a year | 0.33 | 0.02 | (0.29, 0.36) |  |
|  | Never | 0.22 | 0.01 | (0.20, 0.24) |  |
| Education | up to 8 years | 0.19 | 0.02 | (0.15, 0.23) | <0.001 |
|  | 9-15 years | 0.27 | 0.01 | (0.25, 0.30) |  |
|  | 16+years | 0.39 | 0.01 | (0.36, 0.42) |  |
| Immigration status | Born in this country | 0.29 | 0.01 | (0.27, 0.31) | 0.002 |
|  | Born in another country | 0.34 | 0.03 | (0.29, 0.40) |  |
| Religious affiliation | Christianity | 0.32 | 0.01 | (0.30, 0.35) | <0.001 |
|  | Islam | 0.33 | 0.05 | (0.23, 0.42) |  |
|  | Hinduism | 0.39 | 0.10 | (0.19, 0.58) |  |
|  | Buddhism | 0.36 | 0.12 | (0.12, 0.60) |  |
|  | Judaism | 0.36 | 0.10 | (0.15, 0.56) |  |
|  | Sikhism | 0.54 | 0.15 | (0.23, 0.85) |  |
|  | Baha’i | 0.00 | * | * |  |
|  | Taoism | 0.86 | 0.14 | (0.29, 1.43) |  |
|  | Confucianism | 0.00 | * | * |  |
|  | Primal, Animist, or Folk religion | 0.48 | 0.13 | (0.21, 0.75) |  |
|  | Some other religion | 0.20 | 0.06 | (0.09, 0.32) |  |
|  | No religion/Atheist/Agnostic | 0.25 | 0.01 | (0.23, 0.28) |  |
| Race and ethnicity | Asian | 0.35 | 0.04 | (0.28, 0.42) | <0.001 |
|  | Black | 0.45 | 0.05 | (0.34, 0.55) |  |
|  | White | 0.29 | 0.01 | (0.27, 0.30) |  |
|  | Other | 0.30 | 0.07 | (0.16, 0.44) |  |

***Table S22a. Nationally representative descriptive statistics for United States***

| **Characteristic** | **N = 38,312**^1^ |
| --- | --- |
| **Age group** |  |
| 18-24 | 2,682 (7.0%) |
| 25-29 | 3,540 (9.2%) |
| 30-39 | 7,284 (19%) |
| 40-49 | 5,649 (15%) |
| 50-59 | 6,745 (18%) |
| 60-69 | 6,832 (18%) |
| 70-79 | 4,054 (11%) |
| 80 or older | 1,525 (4.0%) |
| Missing | 0 (0%) |
| **Gender** |  |
| Male | 18,222 (48%) |
| Female | 19,562 (51%) |
| Other | 392 (1.0%) |
| Missing | 136 (0.4%) |
| **Marital status** |  |
| Married | 20,360 (53%) |
| Separated | 727 (1.9%) |
| Divorced | 3,636 (9.5%) |
| Widowed | 1,978 (5.2%) |
| Never | 9,431 (25%) |
| Domestic Partner | 1,971 (5.1%) |
| Missing | 207 (0.5%) |
| **Employment** |  |
| Employed for an employer | 19,502 (51%) |
| Self-employed | 3,445 (9.0%) |
| Retired | 9,016 (24%) |
| Student | 1,145 (3.0%) |
| Homemaker | 2,049 (5.3%) |
| Unemployed and looking for a job | 1,777 (4.6%) |
| None of these/other | 1,292 (3.4%) |
| Missing | 87 (0.2%) |
| **Religious service attendance** |  |
| >1/week | 2,633 (6.9%) |
| 1/week | 5,887 (15%) |
| 1-3/month | 2,819 (7.4%) |
| A few times a year | 8,870 (23%) |
| Never | 17,975 (47%) |
| Missing | 128 (0.3%) |
| **Education** |  |
| up to 8 years | 210 (0.5%) |
| 9-15 years | 25,322 (66%) |
| 16+years | 12,705 (33%) |
| Missing | 75 (0.2%) |
| **Immigration** |  |
| Born in this country | 34,865 (91%) |
| Born in another country | 3,020 (7.9%) |
| Missing | 427 (1.1%) |
| **Religious affiliation** |  |
| Christianity | 22,954 (60%) |
| Islam | 205 (0.5%) |
| Hinduism | 167 (0.4%) |
| Buddhism | 336 (0.9%) |
| Judaism | 638 (1.7%) |
| Sikhism | 24 (<0.1%) |
| Baha’i | 13 (<0.1%) |
| Jainism | 18 (<0.1%) |
| Shinto | 12 (<0.1%) |
| Taoism | 93 (0.2%) |
| Confucianism | 8 (<0.1%) |
| Primal, Animist, or Folk religion | 240 (0.6%) |
| Some other religion | 1,267 (3.3%) |
| No religion/Atheist/Agnostic | 11,870 (31%) |
| Missing | 467 (1.2%) |
| **Race and ethnicity** |  |
| Asian | 2,466 (6.4%) |
| Black | 4,501 (12%) |
| White | 23,605 (62%) |
| Other | 997 (2.6%) |
| Hispanic | 6,724 (18%) |
| Missing | 20 (<0.1%) |
| ^1^n (%) | |

***Table S22b. Proportions by demographic category for United States***

| Variable | Category | Proportion | SE | 95% CI | Global p-value |
| --- | --- | --- | --- | --- | --- |
| Age group | 18-24 | 0.30 | 0.04 | (0.23, 0.37) | <0.001 |
|  | 25-29 | 0.23 | 0.02 | (0.18, 0.27) |  |
|  | 30-39 | 0.23 | 0.01 | (0.20, 0.25) |  |
|  | 40-49 | 0.28 | 0.01 | (0.26, 0.31) |  |
|  | 50-59 | 0.27 | 0.01 | (0.25, 0.29) |  |
|  | 60-69 | 0.30 | 0.01 | (0.28, 0.31) |  |
|  | 70-79 | 0.32 | 0.01 | (0.30, 0.34) |  |
|  | 80 or older | 0.27 | 0.02 | (0.24, 0.30) |  |
| Gender | Male | 0.26 | 0.01 | (0.25, 0.27) | <0.001 |
|  | Female | 0.28 | 0.01 | (0.27, 0.30) |  |
|  | Other | 0.26 | 0.07 | (0.13, 0.39) |  |
| Marital status | Married | 0.31 | 0.01 | (0.30, 0.32) | <0.001 |
|  | Separated | 0.22 | 0.04 | (0.13, 0.30) |  |
|  | Divorced | 0.23 | 0.01 | (0.21, 0.25) |  |
|  | Widowed | 0.27 | 0.01 | (0.24, 0.30) |  |
|  | Never | 0.23 | 0.01 | (0.20, 0.26) |  |
|  | Domestic Partner | 0.16 | 0.02 | (0.13, 0.20) |  |
| Employment | Employed for an employer | 0.26 | 0.01 | (0.25, 0.28) | <0.001 |
|  | Self-employed | 0.31 | 0.02 | (0.28, 0.35) |  |
|  | Retired | 0.29 | 0.01 | (0.28, 0.31) |  |
|  | Student | 0.30 | 0.04 | (0.21, 0.39) |  |
|  | Homemaker | 0.30 | 0.02 | (0.26, 0.33) |  |
|  | Unemployed and looking for a job | 0.19 | 0.04 | (0.12, 0.26) |  |
|  | None of these/other | 0.18 | 0.02 | (0.13, 0.23) |  |
| Religious service attendance | >1/week | 0.64 | 0.02 | (0.60, 0.68) | <0.001 |
|  | 1/week | 0.45 | 0.01 | (0.43, 0.48) |  |
|  | 1-3/month | 0.37 | 0.02 | (0.33, 0.41) |  |
|  | A few times a year | 0.22 | 0.01 | (0.20, 0.24) |  |
|  | Never | 0.17 | 0.01 | (0.16, 0.18) |  |
| Education | up to 8 years | 0.18 | 0.08 | (0.02, 0.35) | <0.001 |
|  | 9-15 years | 0.23 | 0.01 | (0.22, 0.24) |  |
|  | 16+years | 0.36 | 0.00 | (0.35, 0.37) |  |
| Immigration status | Born in this country | 0.28 | 0.01 | (0.27, 0.29) | <0.001 |
|  | Born in another country | 0.21 | 0.02 | (0.18, 0.24) |  |
| Religious affiliation | Christianity | 0.31 | 0.01 | (0.30, 0.33) | <0.001 |
|  | Islam | 0.19 | 0.07 | (0.05, 0.33) |  |
|  | Hinduism | 0.33 | 0.07 | (0.19, 0.48) |  |
|  | Buddhism | 0.26 | 0.05 | (0.17, 0.36) |  |
|  | Judaism | 0.33 | 0.03 | (0.28, 0.38) |  |
|  | Sikhism | 0.30 | 0.17 | (-0.06, 0.66) |  |
|  | Baha’i | 0.41 | 0.14 | (0.09, 0.72) |  |
|  | Jainism | 0.17 | 0.14 | (-0.13, 0.47) |  |
|  | Shinto | 0.11 | 0.10 | (-0.11, 0.33) |  |
|  | Taoism | 0.29 | 0.17 | (-0.06, 0.63) |  |
|  | Confucianism | 0.20 | 0.18 | (-0.23, 0.63) |  |
|  | Primal, Animist, or Folk religion | 0.11 | 0.03 | (0.04, 0.17) |  |
|  | Some other religion | 0.31 | 0.04 | (0.24, 0.38) |  |
|  | No religion/Atheist/Agnostic | 0.19 | 0.01 | (0.17, 0.20) |  |
| Race and ethnicity | Asian | 0.26 | 0.02 | (0.22, 0.30) | <0.001 |
|  | Black | 0.24 | 0.02 | (0.21, 0.27) |  |
|  | White | 0.30 | 0.00 | (0.29, 0.30) |  |
|  | Other | 0.33 | 0.02 | (0.28, 0.38) |  |
|  | Hispanic | 0.21 | 0.02 | (0.18, 0.24) |  |

***Table S23. Population weighted meta-analysis of results demographic group proportions***

| Variable | Category | Proportion | 95% CI of Proportion | SE Analogue (CI Width/4) |
| --- | --- | --- | --- | --- |
| Age group |  |  |  |  |
|  | 18-24 | 0.27 | (0.24,0.29) | 0.01 |
|  | 25-29 | 0.25 | (0.23,0.28) | 0.01 |
|  | 30-39 | 0.25 | (0.23,0.28) | 0.01 |
|  | 40-49 | 0.26 | (0.24,0.29) | 0.01 |
|  | 50-59 | 0.25 | (0.23,0.27) | 0.01 |
|  | 60-69 | 0.25 | (0.23,0.27) | 0.01 |
|  | 70-79 | 0.23 | (0.21,0.25) | 0.01 |
|  | 80 or older | 0.19 | (0.17,0.21) | 0.01 |
| Gender |  |  |  |  |
|  | Male | 0.28 | (0.26,0.30) | 0.01 |
|  | Female | 0.24 | (0.21,0.26) | 0.01 |
|  | Other | 0.18 | (0.16,0.20) | 0.01 |
| Marital status |  |  |  |  |
|  | Married | 0.27 | (0.24,0.29) | 0.01 |
|  | Separated | 0.25 | (0.23,0.27) | 0.01 |
|  | Divorced | 0.29 | (0.26,0.31) | 0.01 |
|  | Widowed | 0.24 | (0.22,0.27) | 0.01 |
|  | Domestic partner | 0.22 | (0.20,0.24) | 0.01 |
|  | Single, never married | 0.26 | (0.23,0.28) | 0.01 |
| Employment status |  |  |  |  |
|  | Employed for an employer | 0.26 | (0.24,0.29) | 0.01 |
|  | Self-employed | 0.29 | (0.27,0.32) | 0.01 |
|  | Retired | 0.23 | (0.21,0.25) | 0.01 |
|  | Student | 0.29 | (0.27,0.32) | 0.01 |
|  | Homemaker | 0.23 | (0.20,0.25) | 0.01 |
|  | Unemployed and looking for a job | 0.24 | (0.22,0.26) | 0.01 |
|  | None of these/other | 0.21 | (0.19,0.24) | 0.01 |
| Education |  |  |  |  |
|  | Up to 8 years | 0.23 | (0.21,0.25) | 0.01 |
|  | 9-15 years | 0.25 | (0.23,0.28) | 0.01 |
|  | 16+ years | 0.31 | (0.29,0.34) | 0.01 |
| Religious service attendance |  |  |  |  |
|  | >1/week | 0.37 | (0.35,0.40) | 0.01 |
|  | 1/week | 0.32 | (0.30,0.35) | 0.01 |
|  | 1-3/month | 0.28 | (0.25,0.30) | 0.01 |
|  | A few times a year | 0.24 | (0.22,0.26) | 0.01 |
|  | Never | 0.18 | (0.17,0.20) | 0.01 |
| Immigration status |  |  |  |  |
|  | Born in this country | 0.26 | (0.24,0.28) | 0.01 |
|  | Born in another country | 0.19 | (0.17,0.21) | 0.01 |


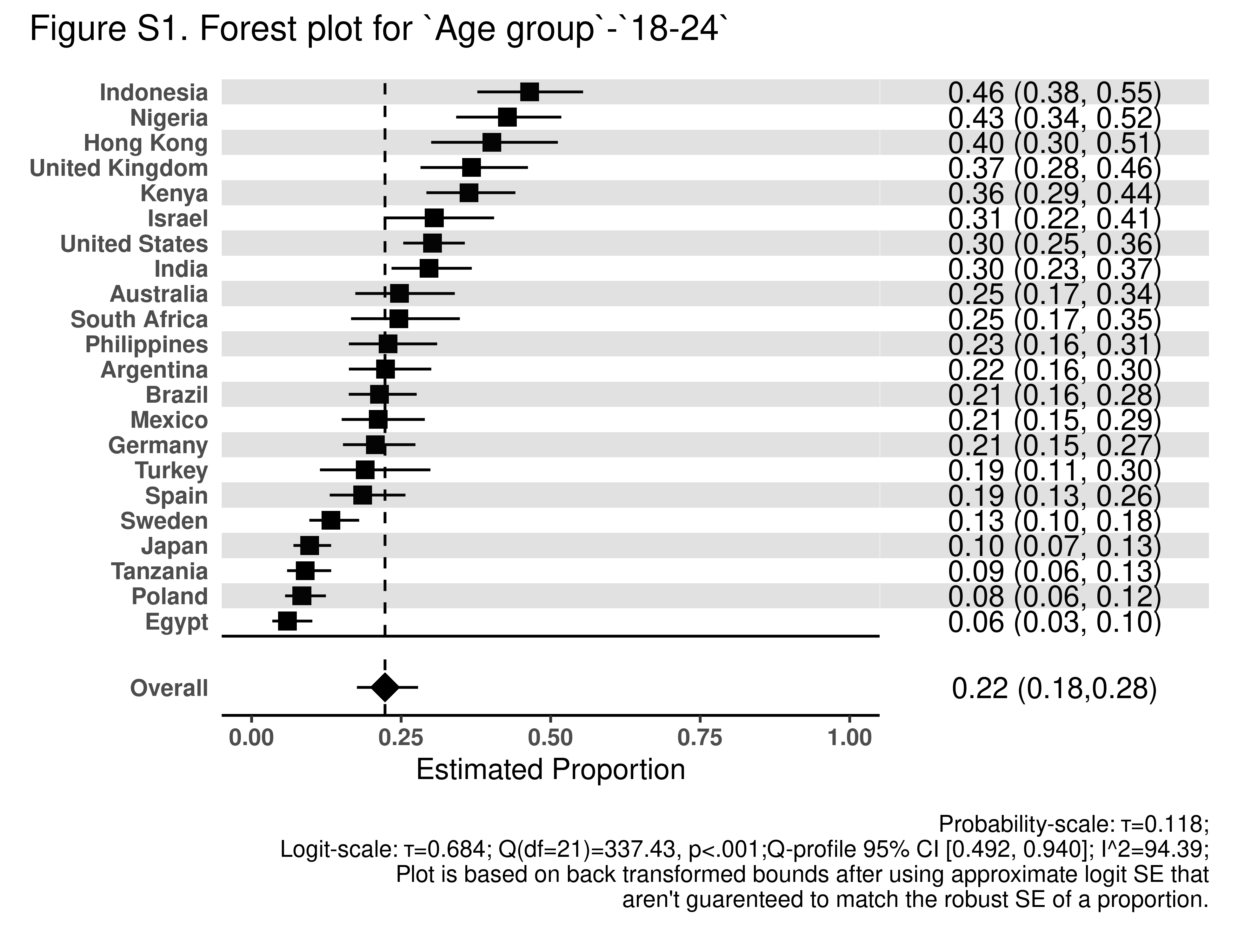

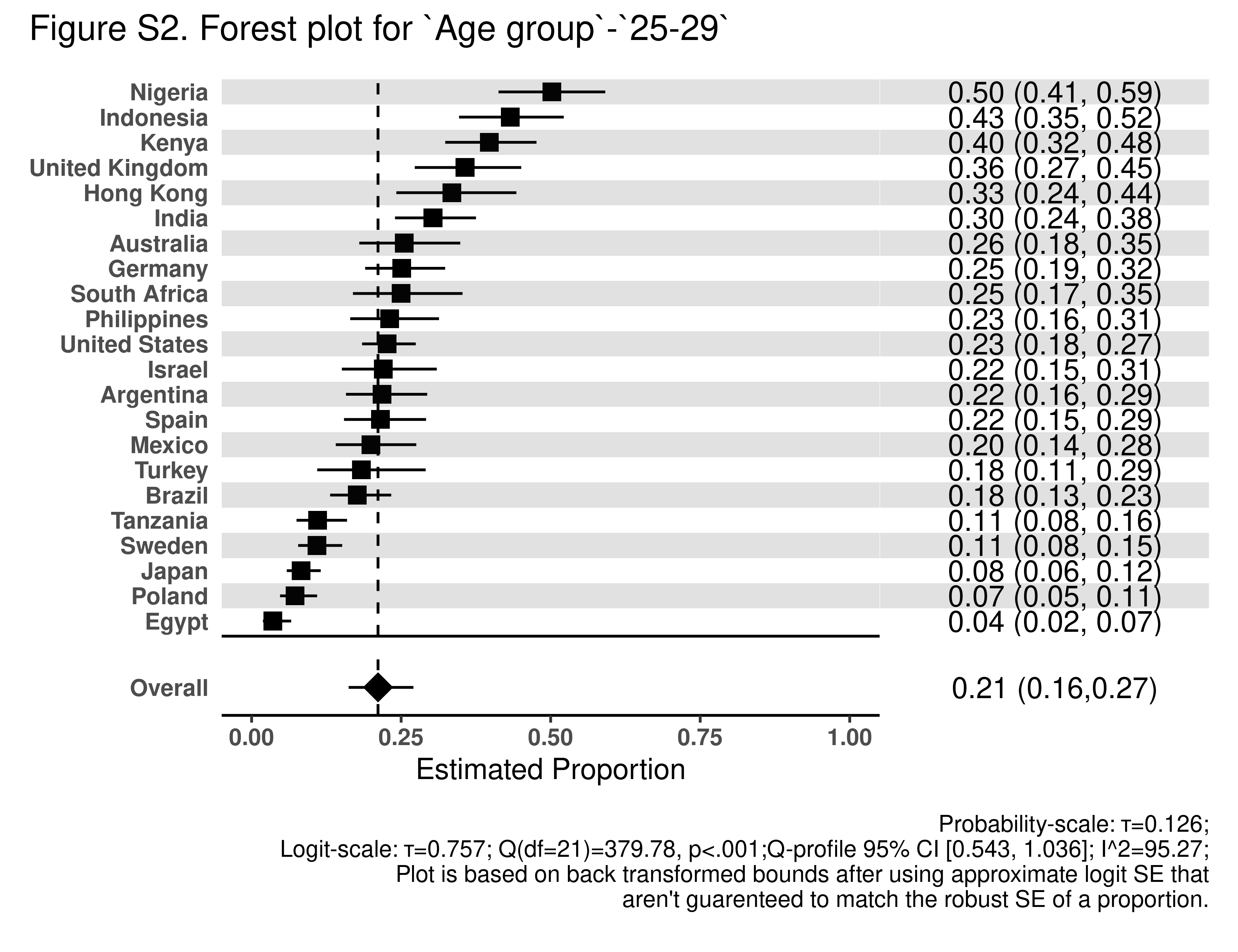

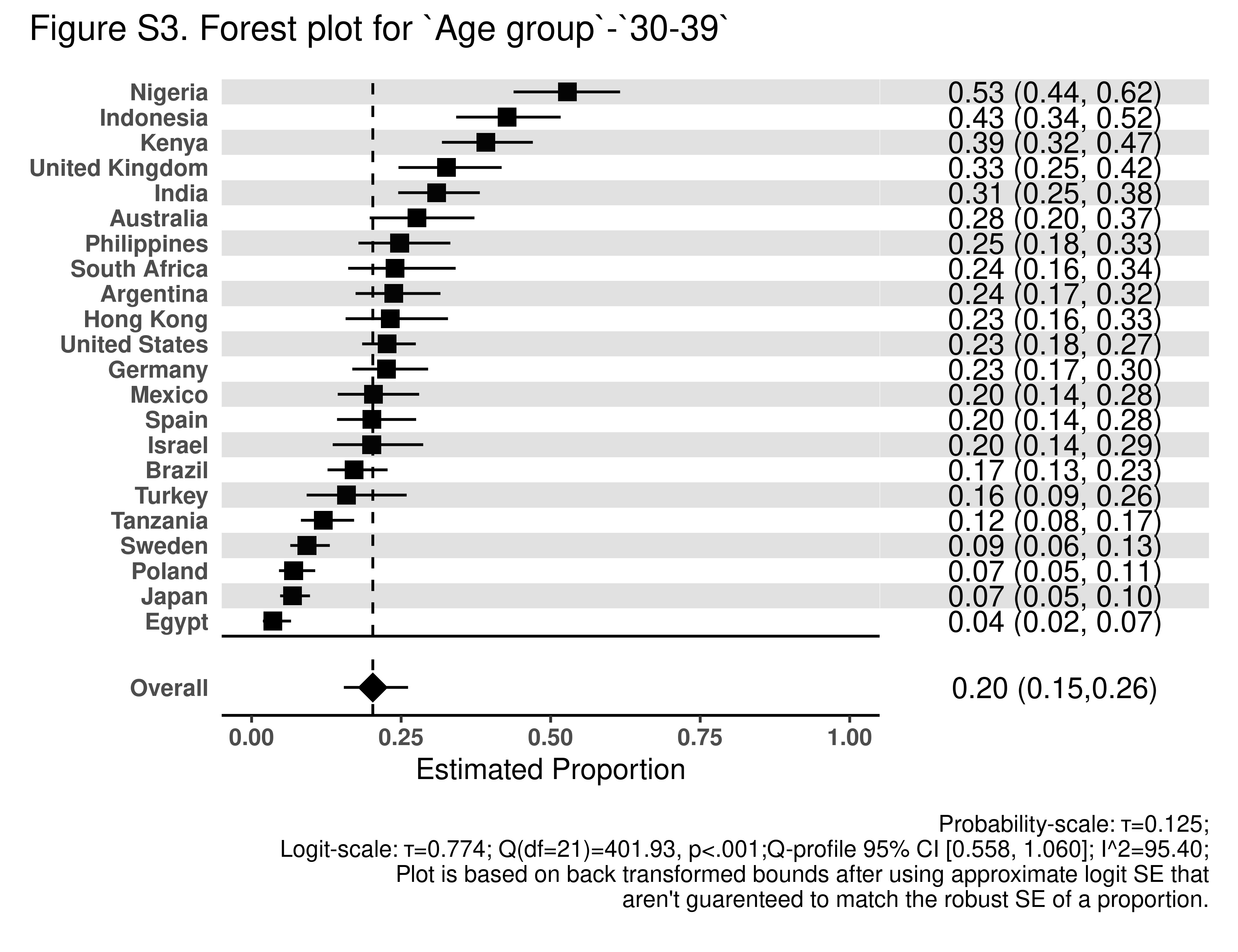

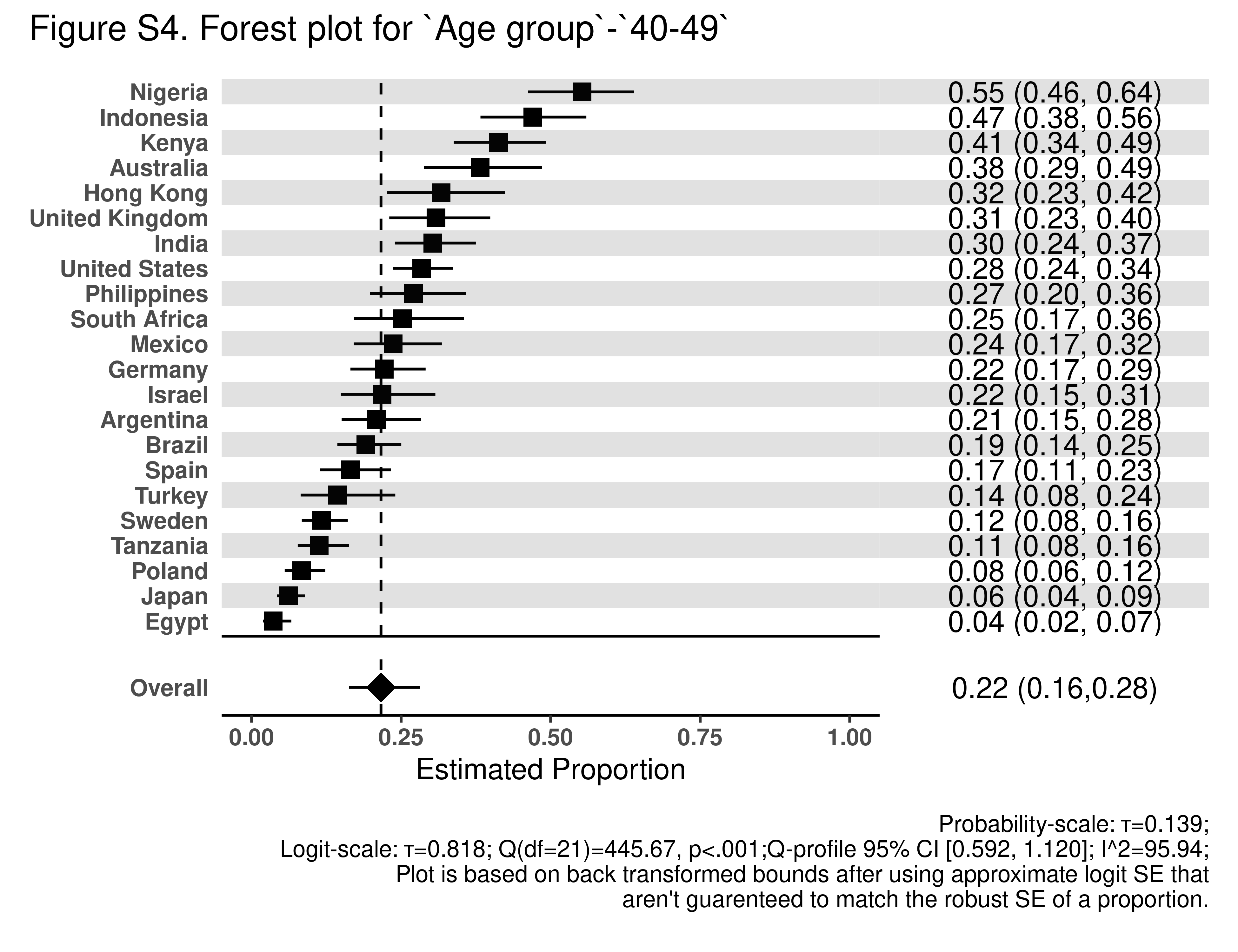

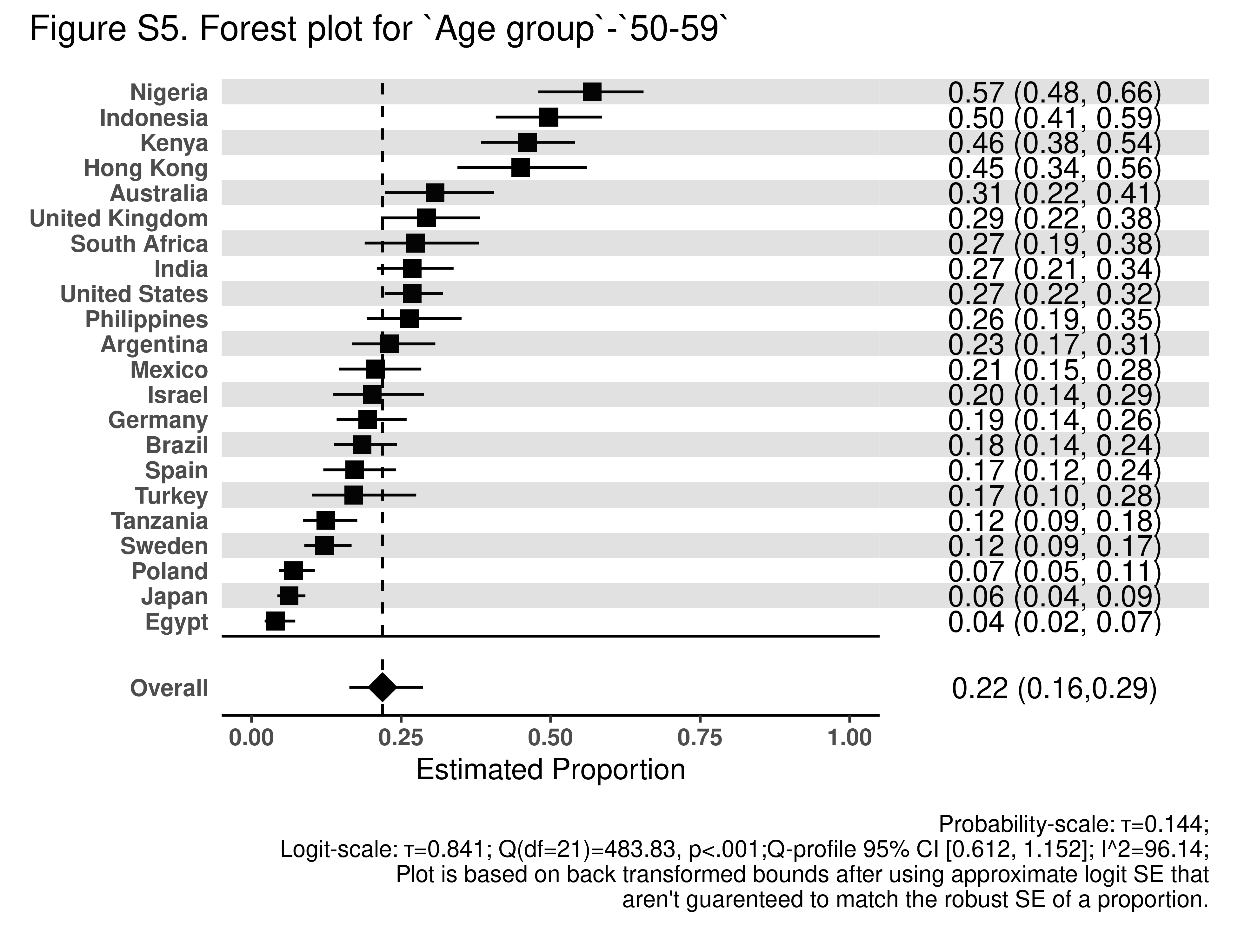

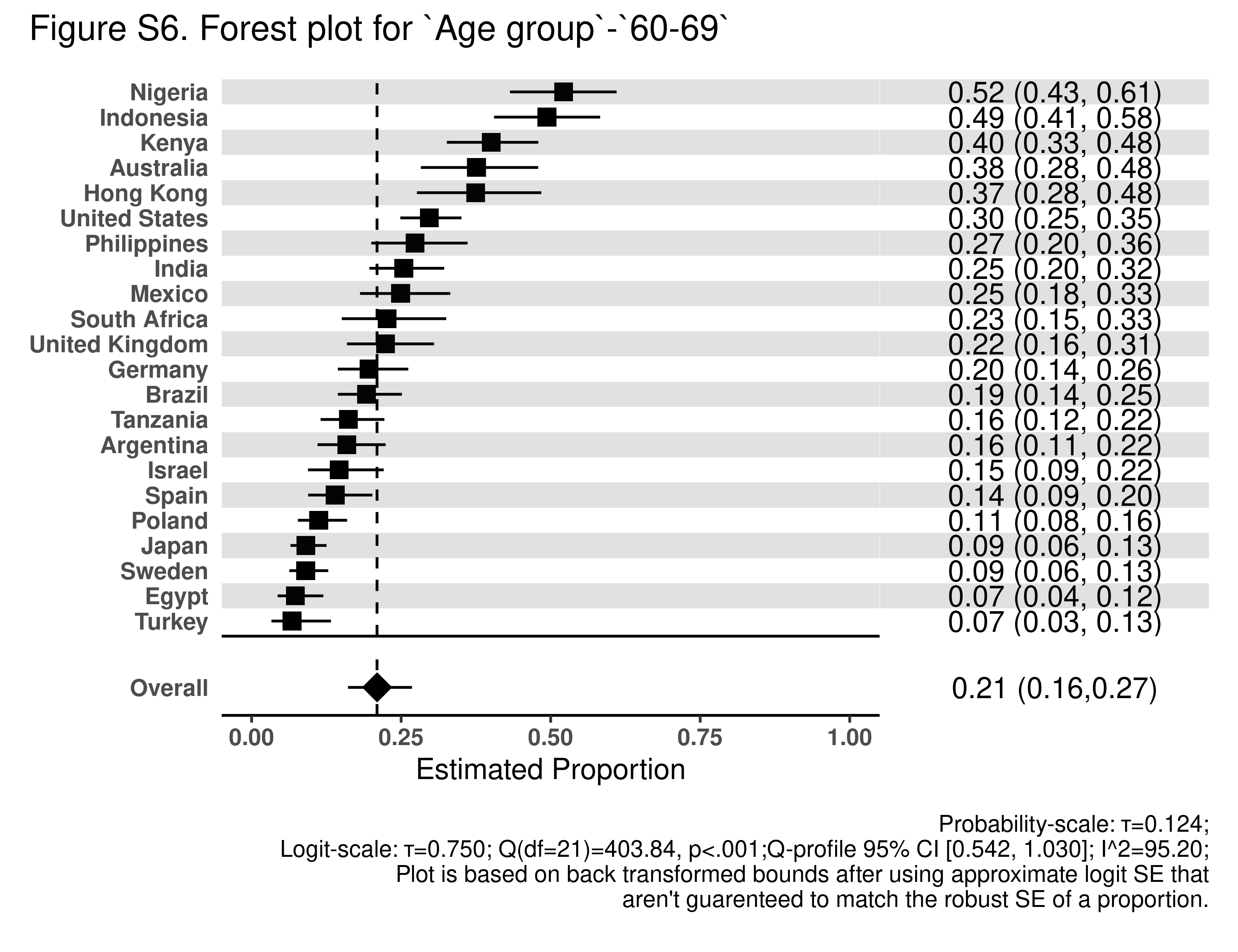

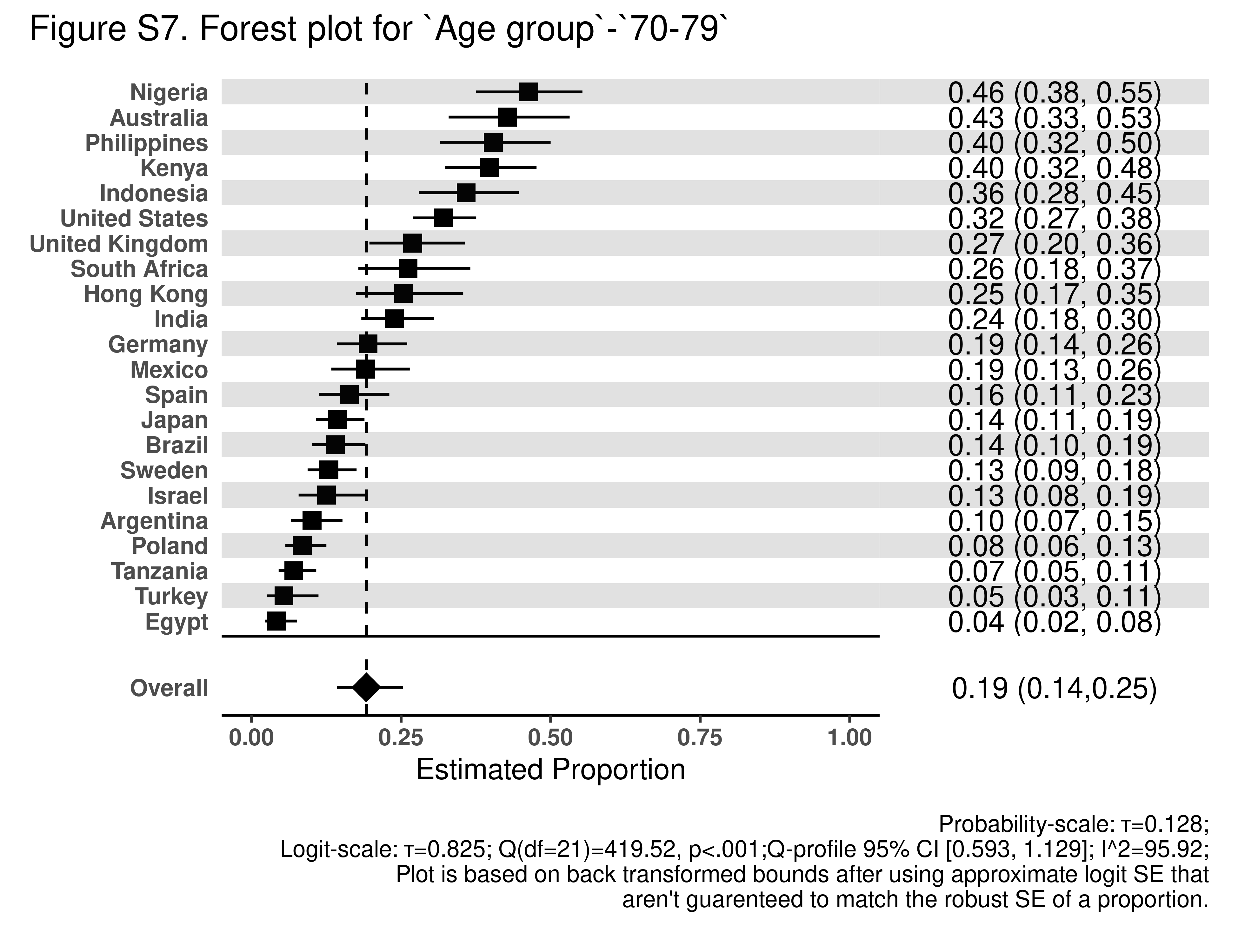

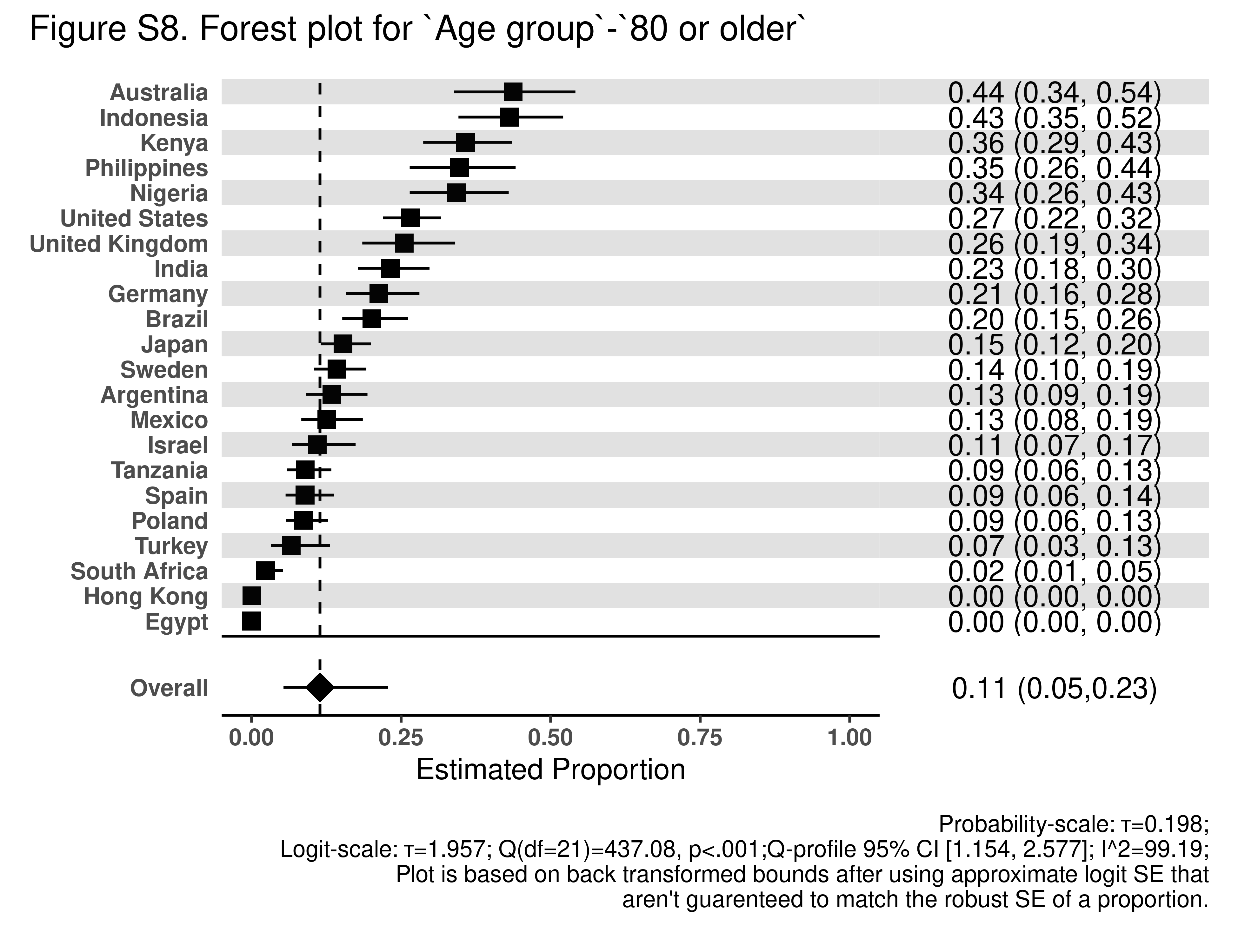

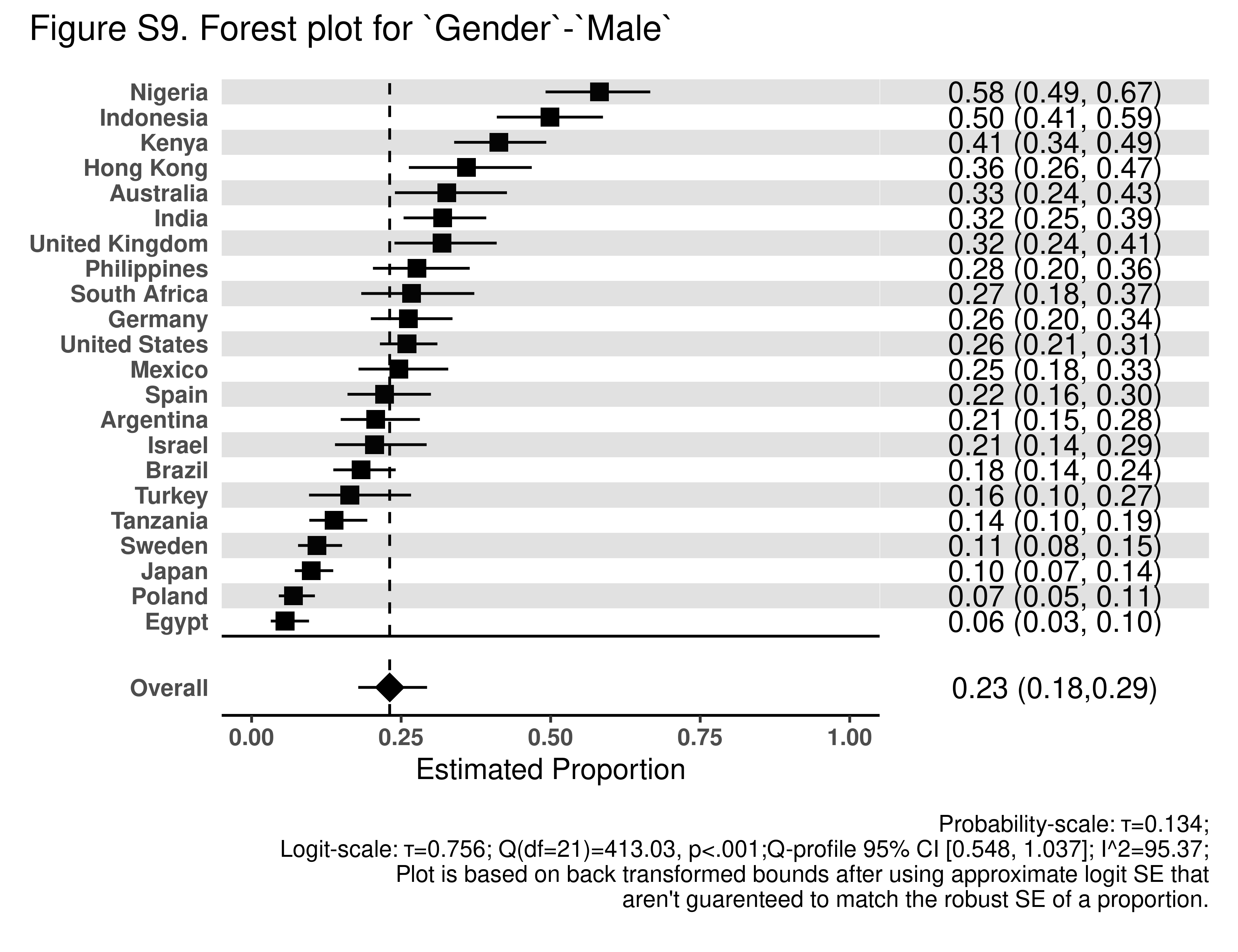

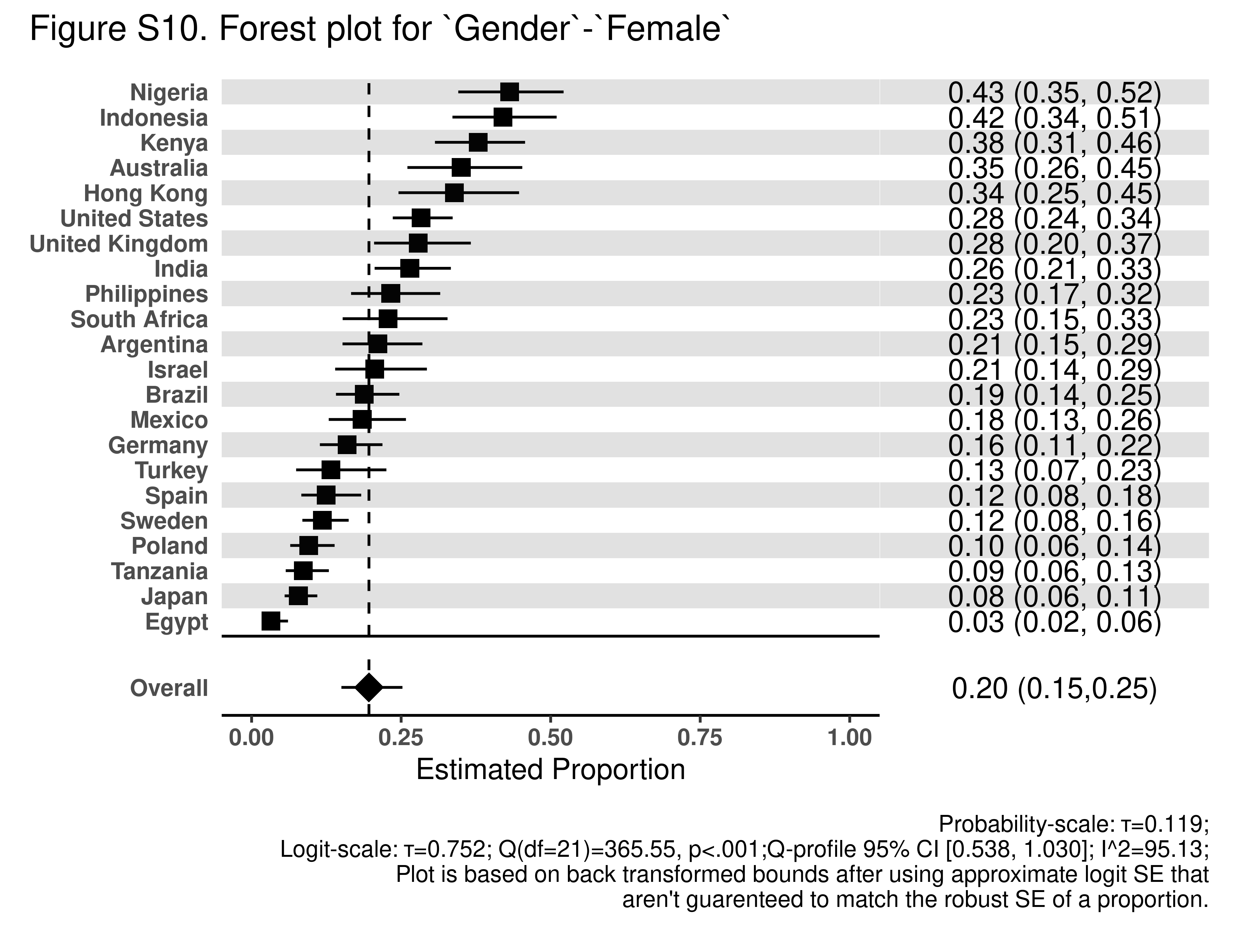

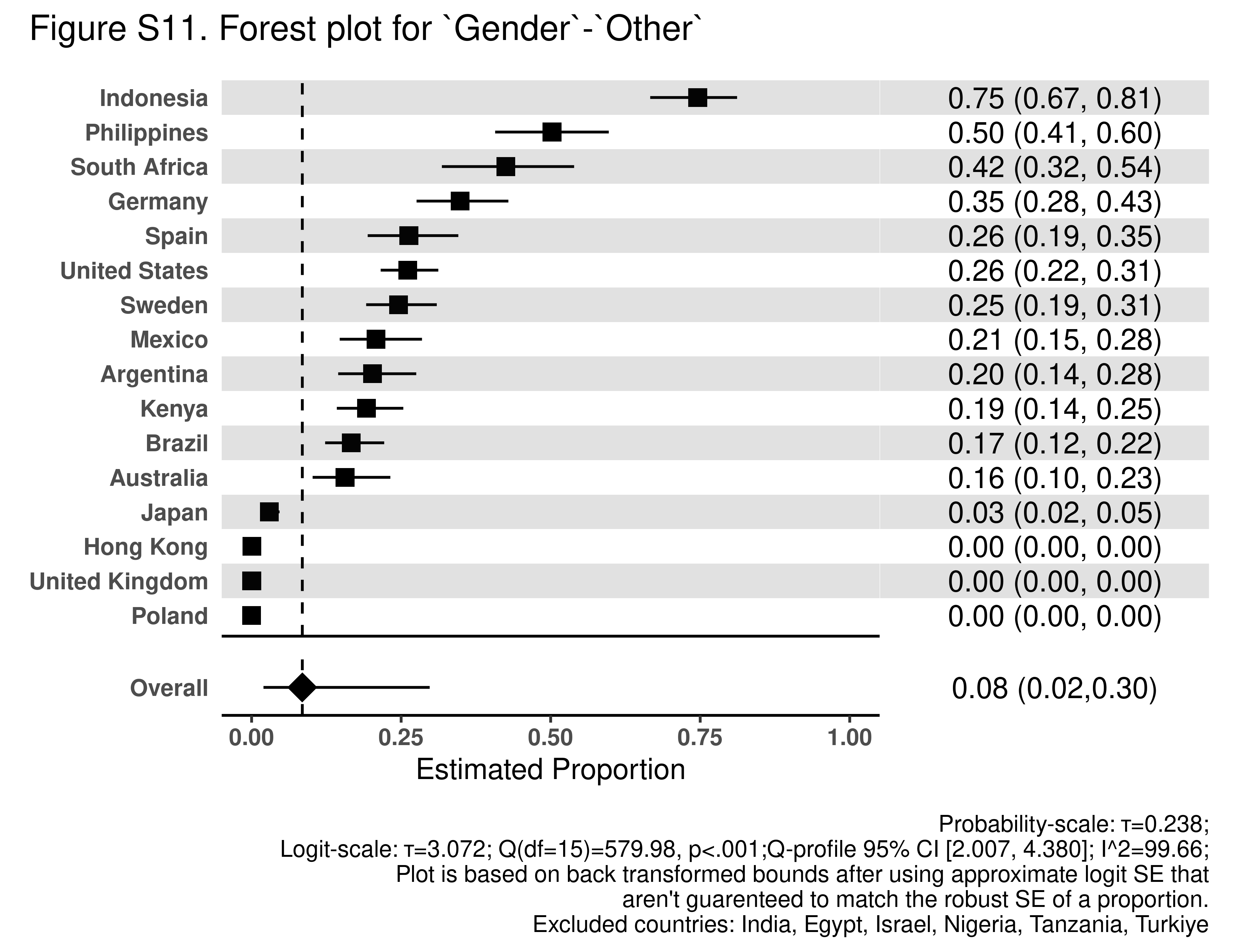

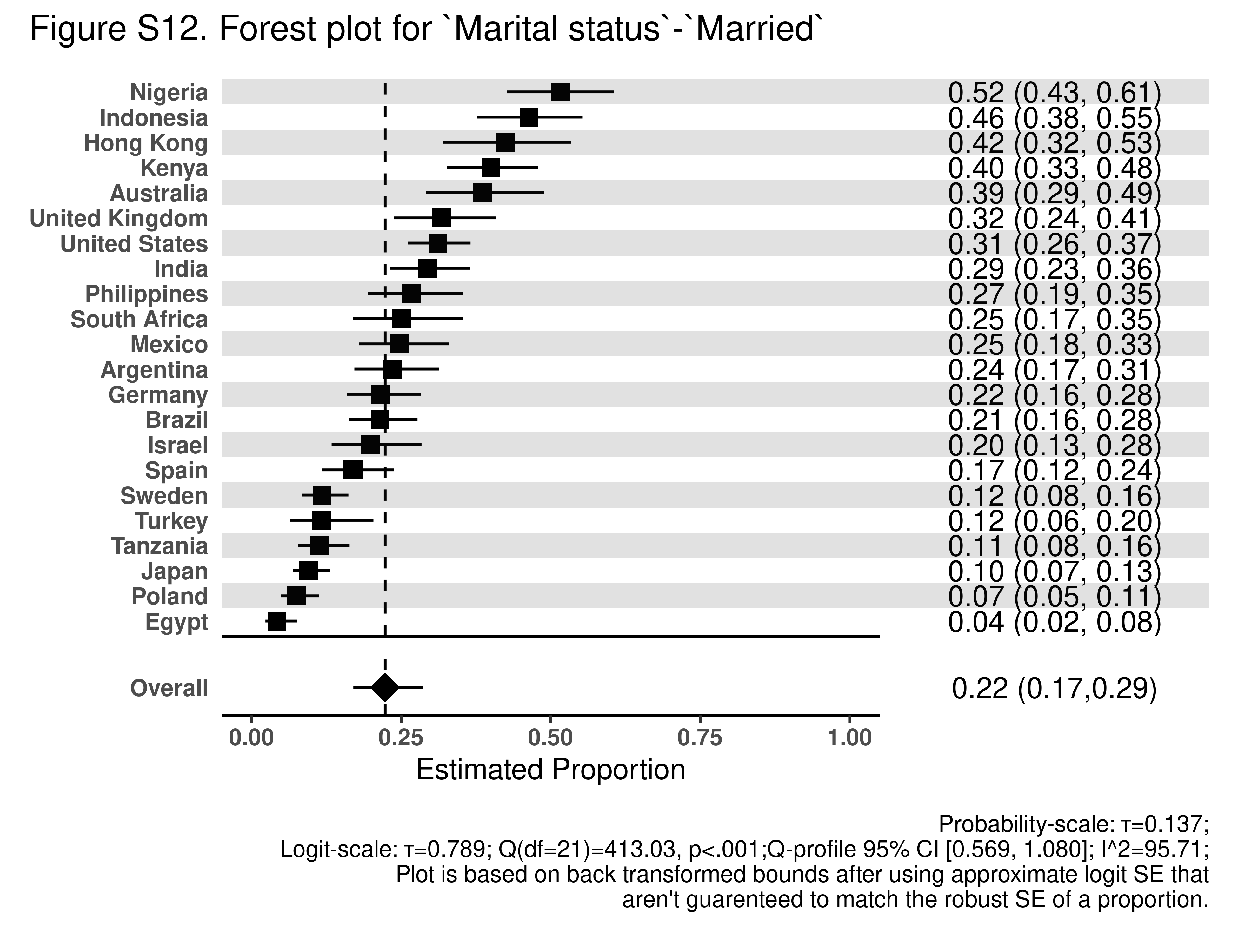

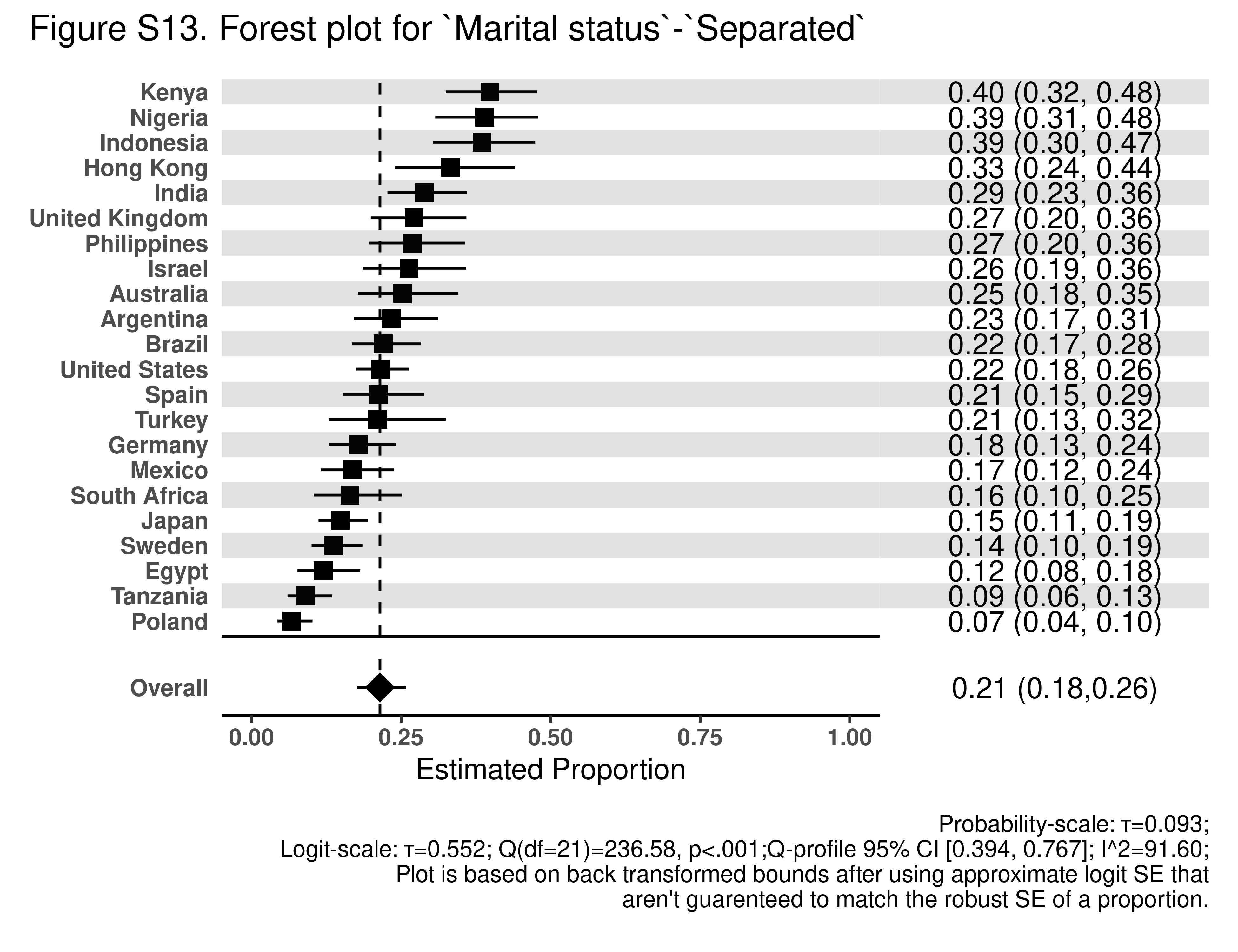

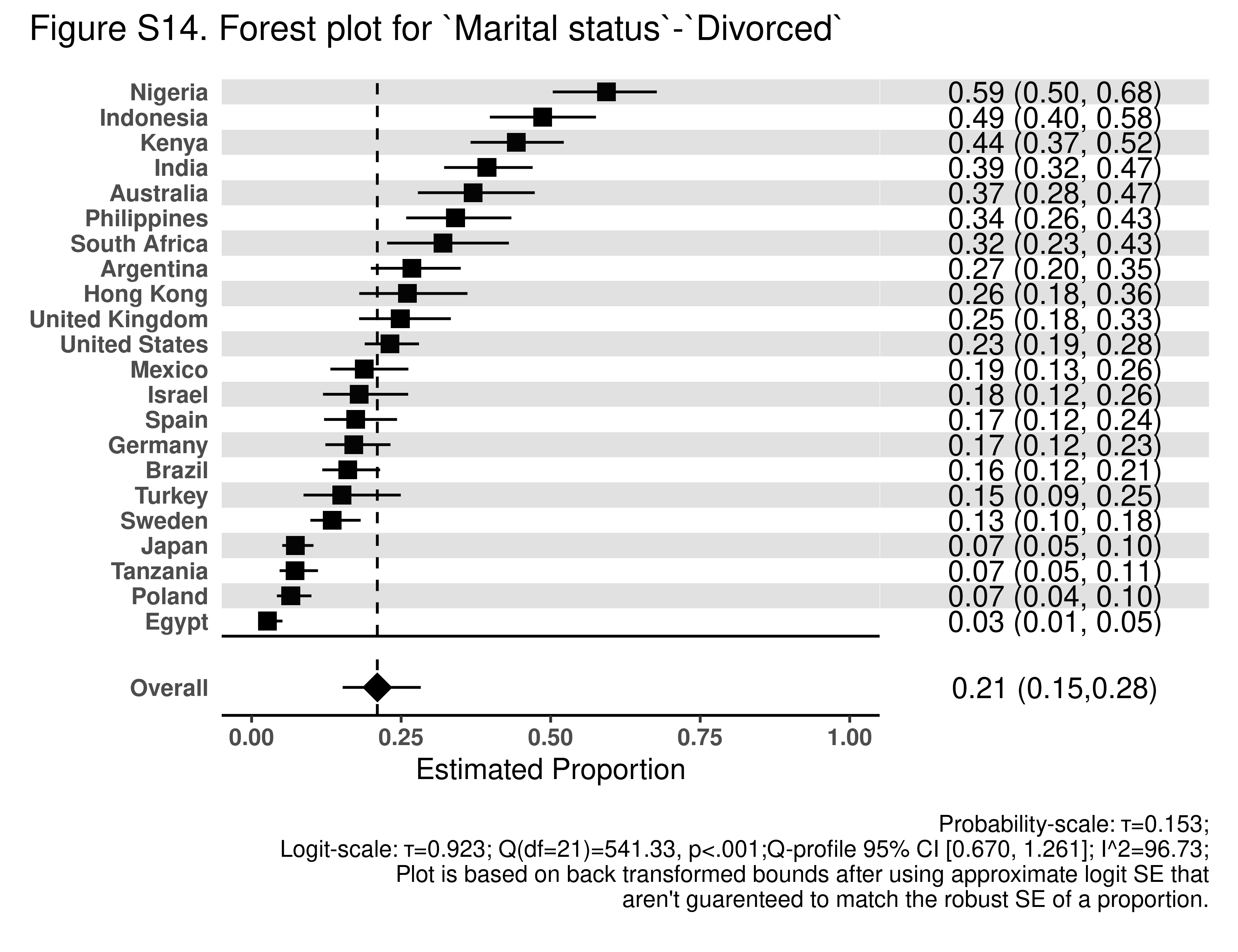

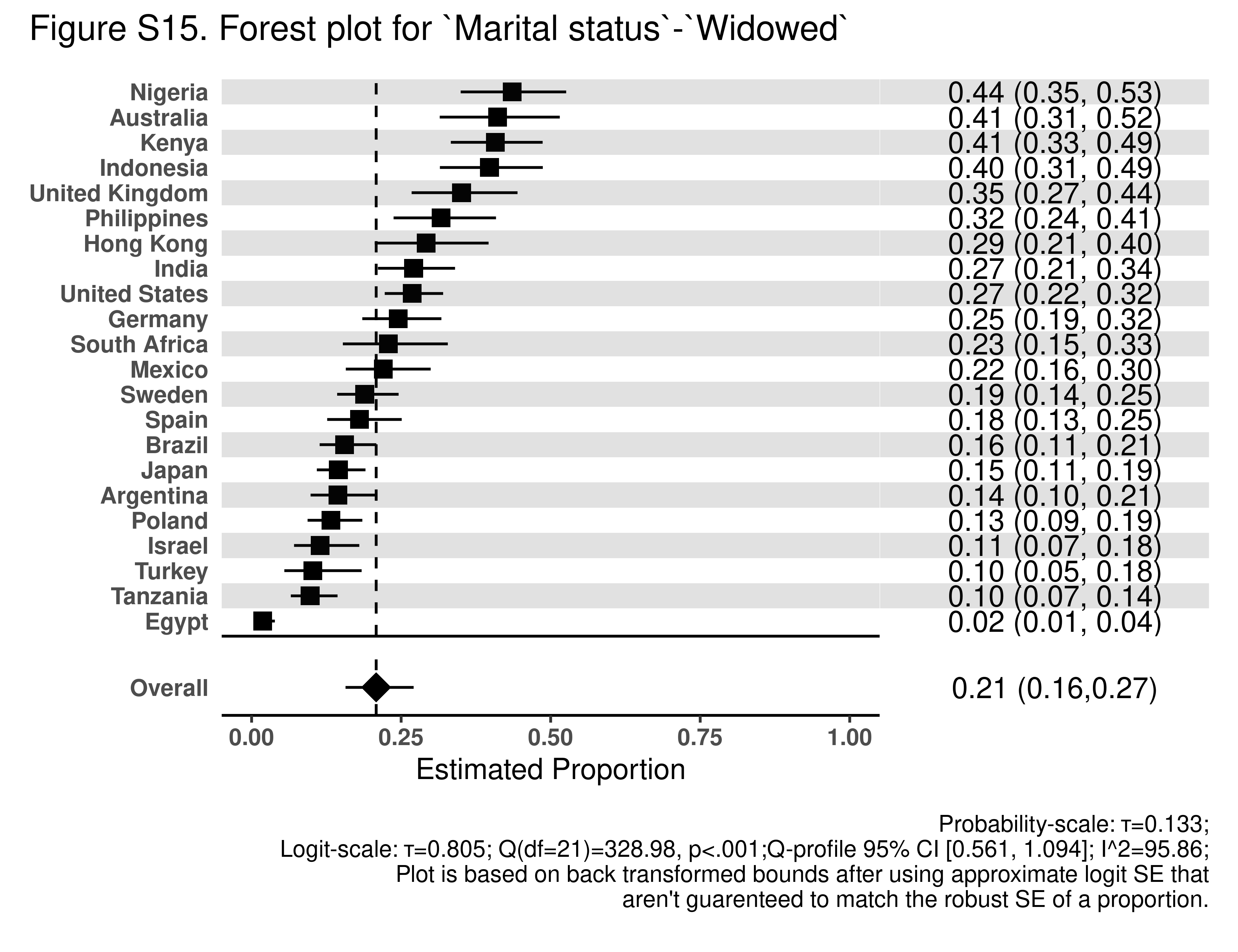

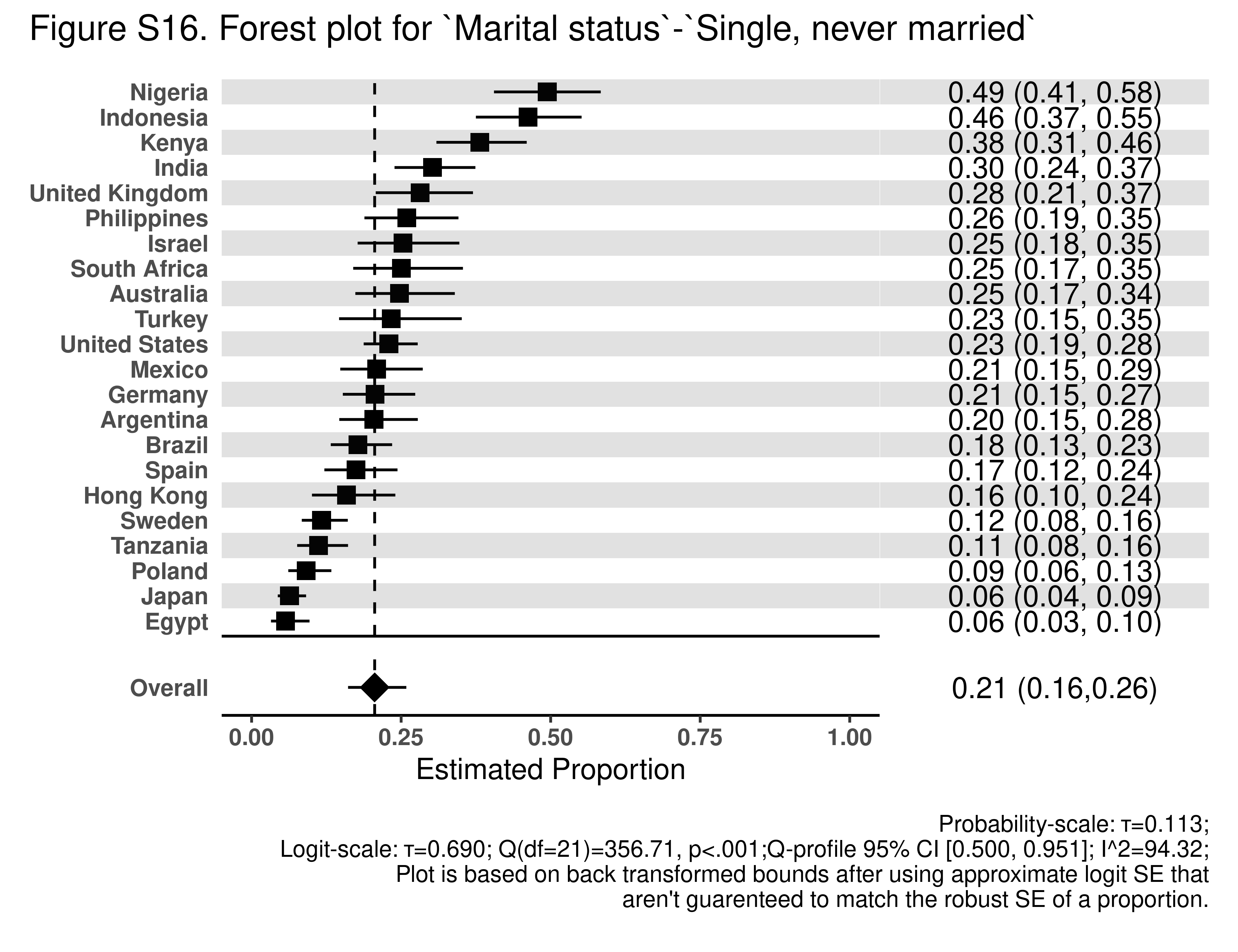

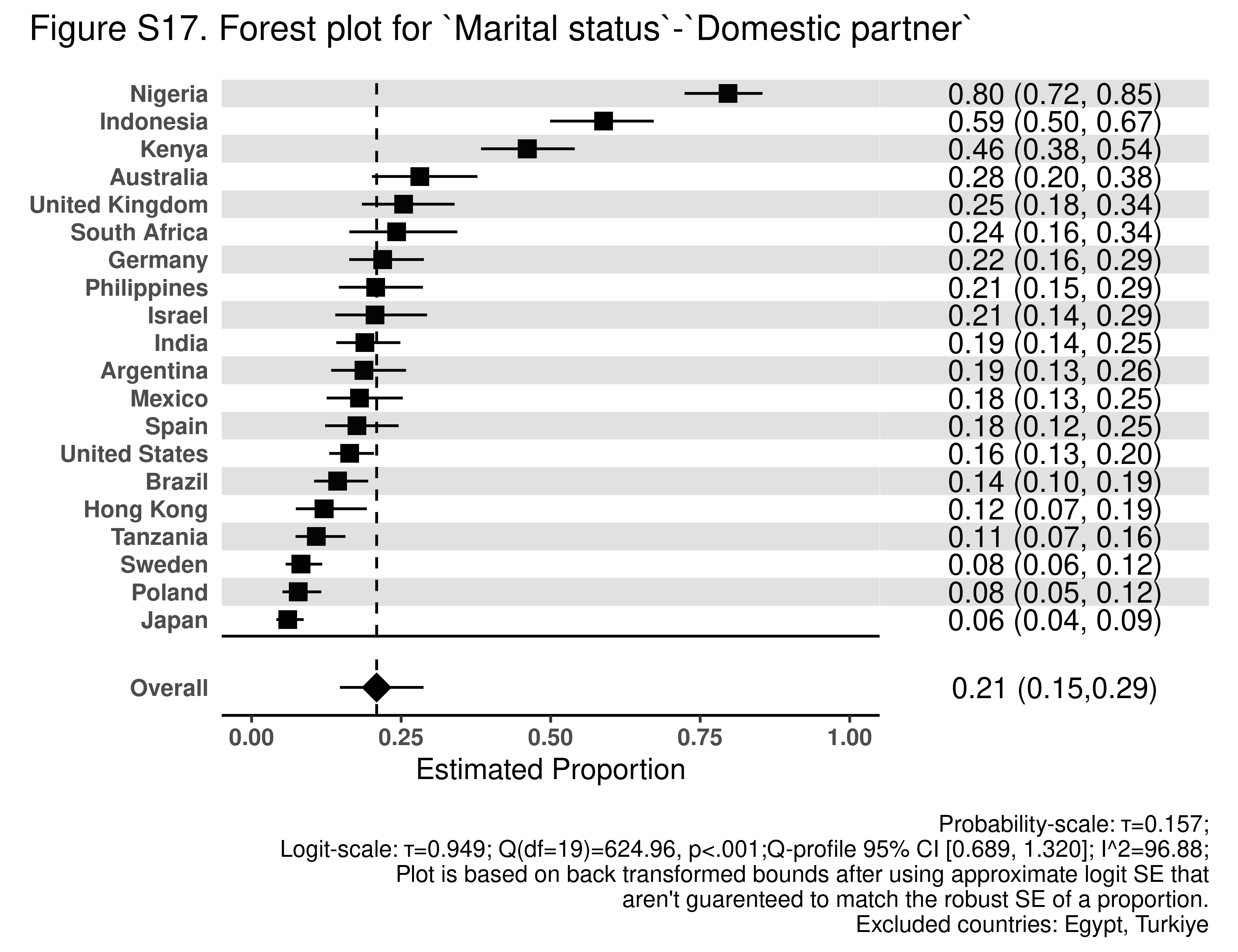

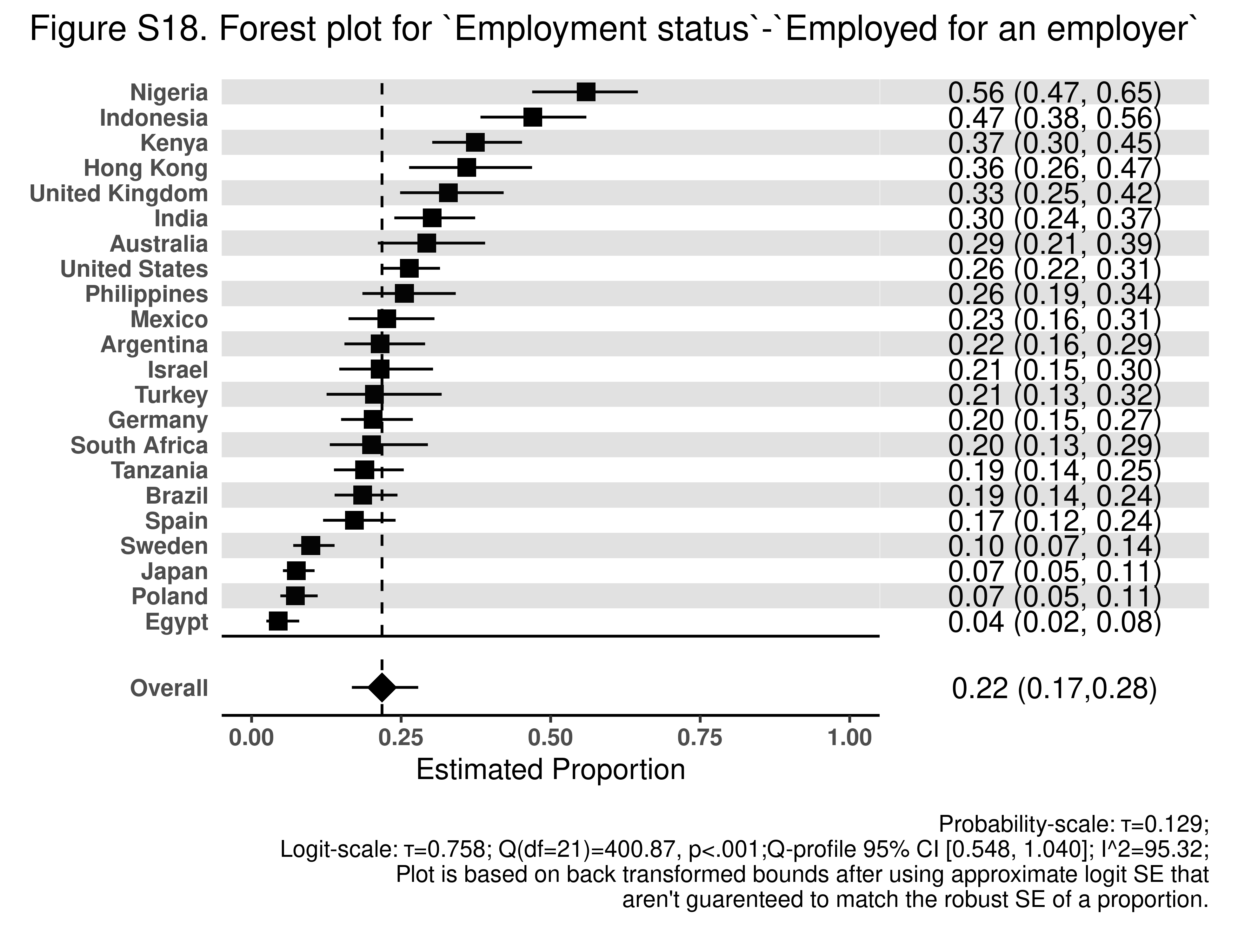

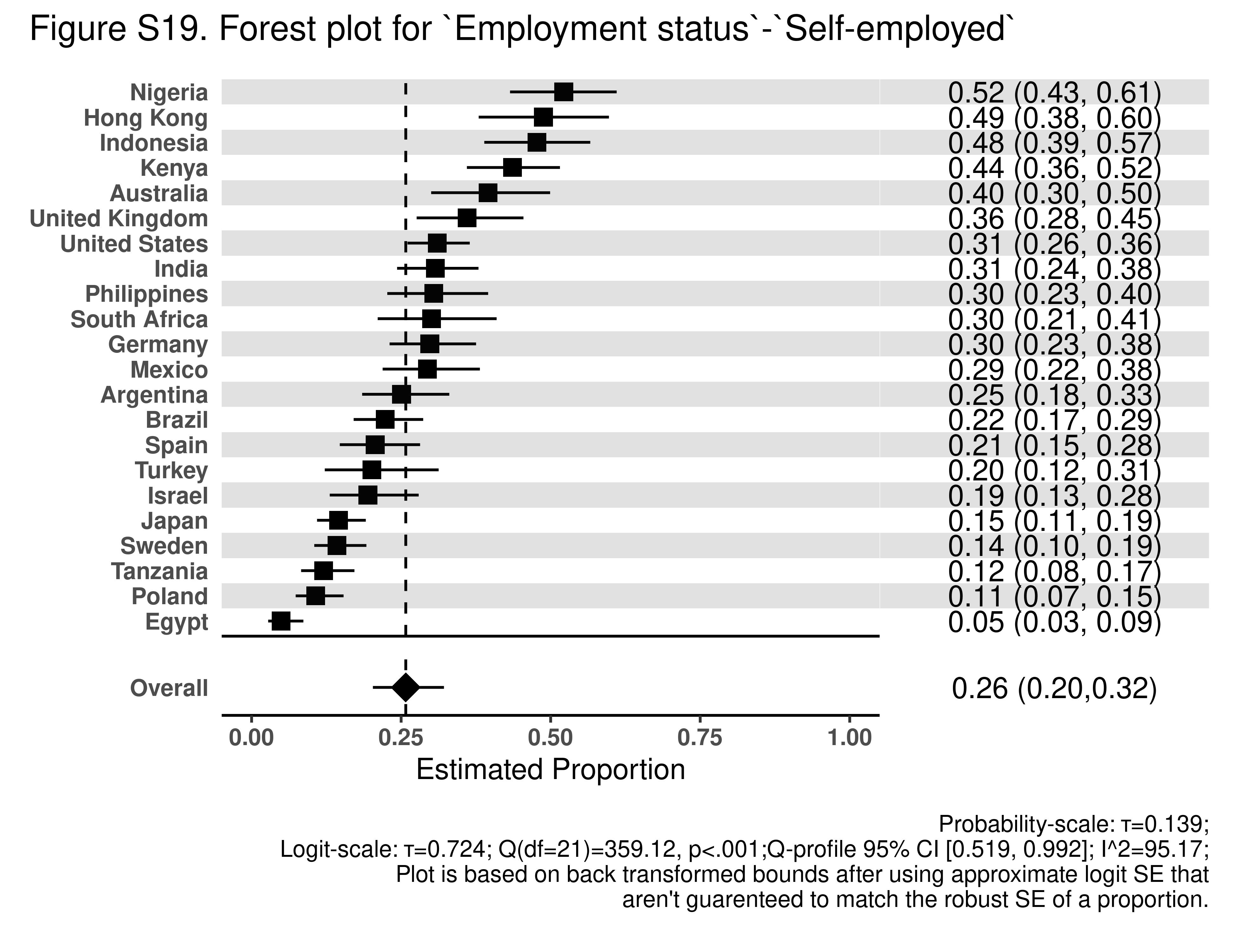

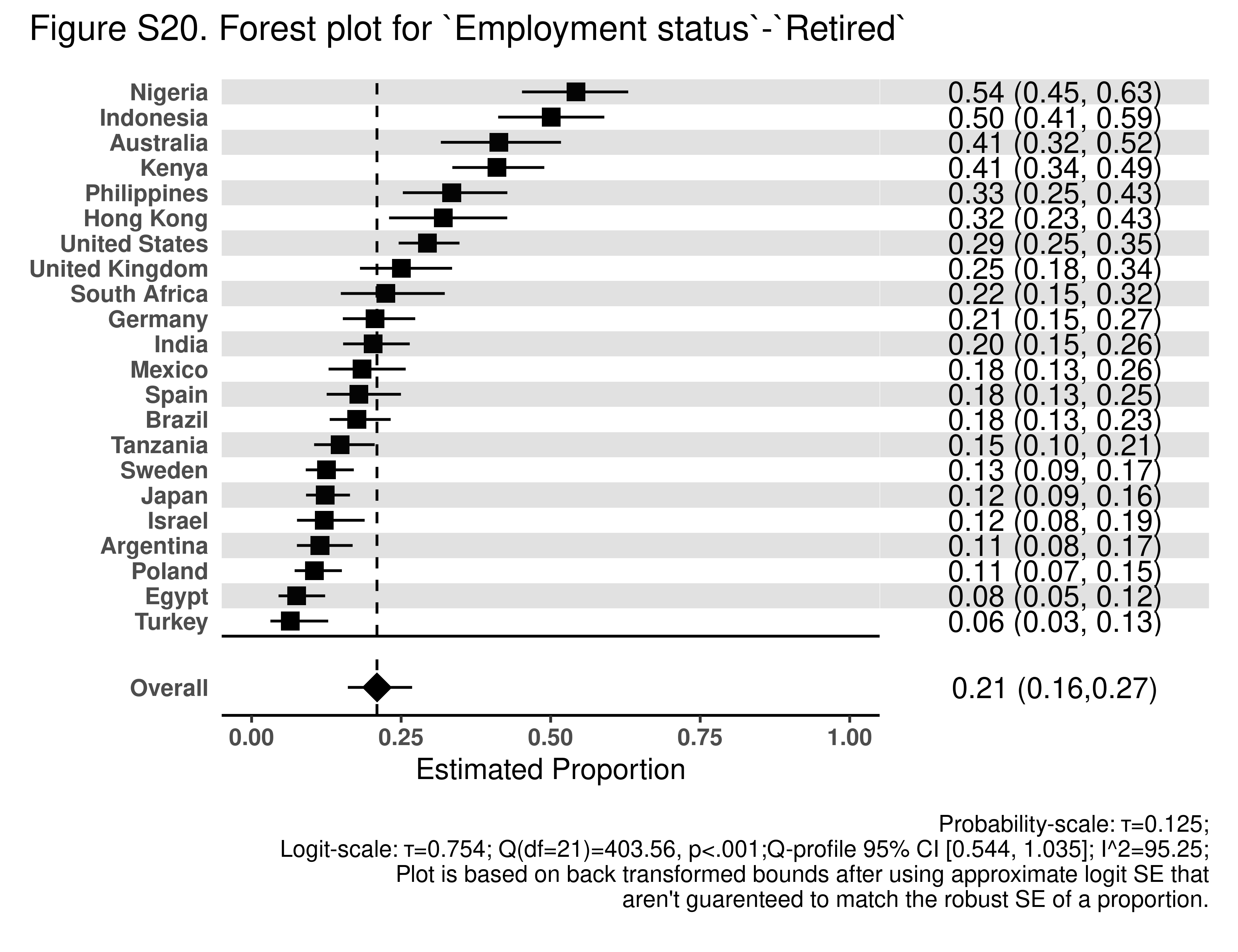

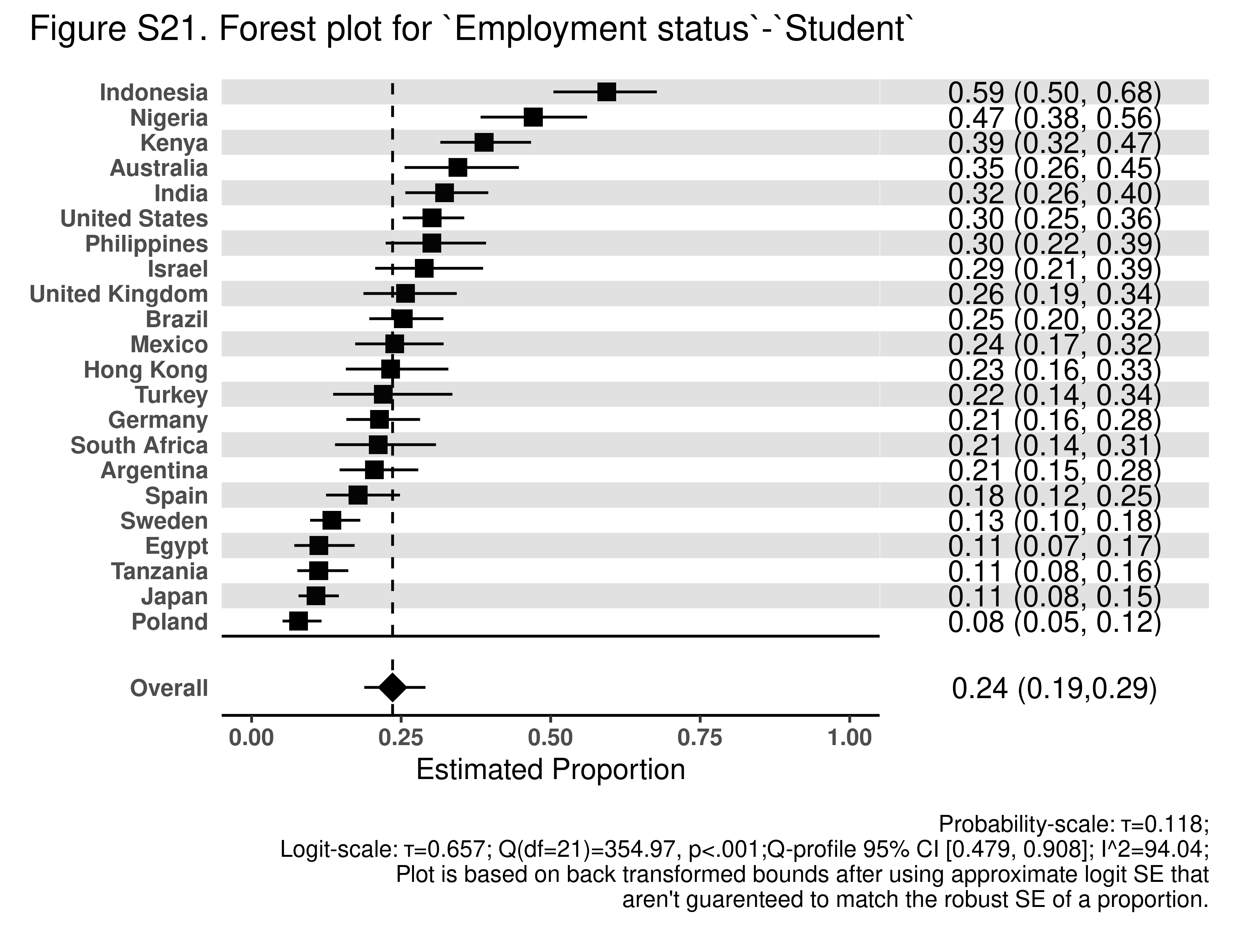

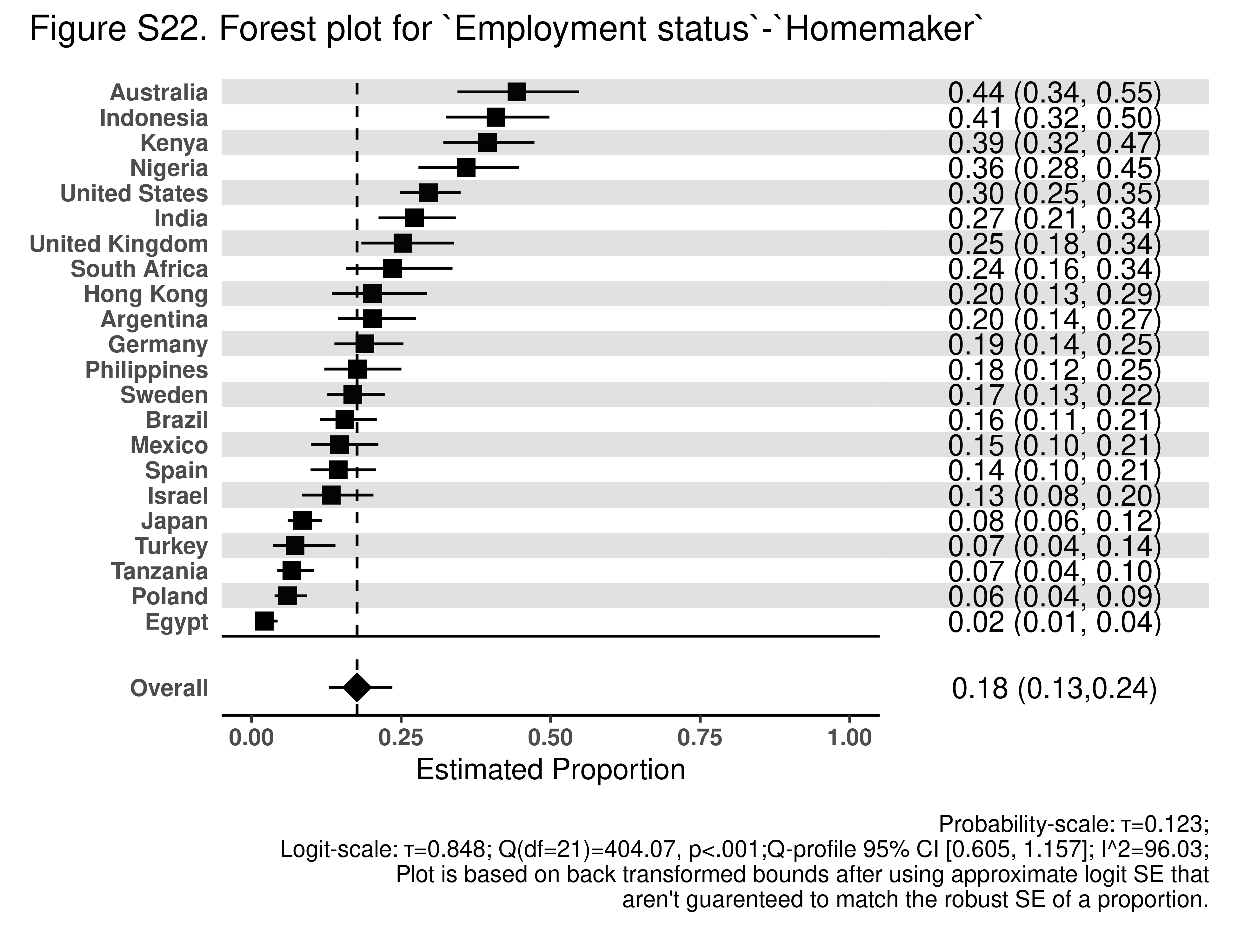

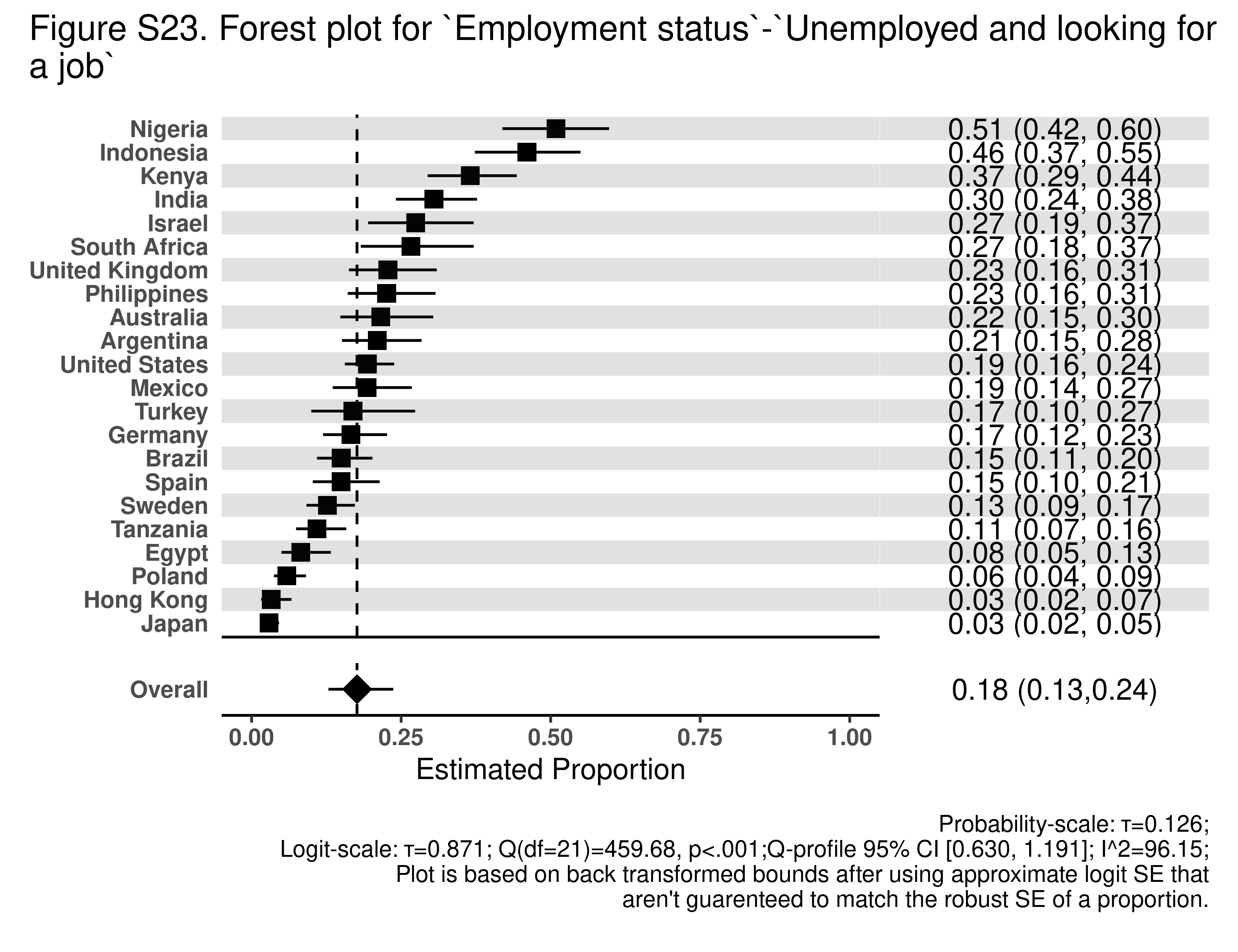

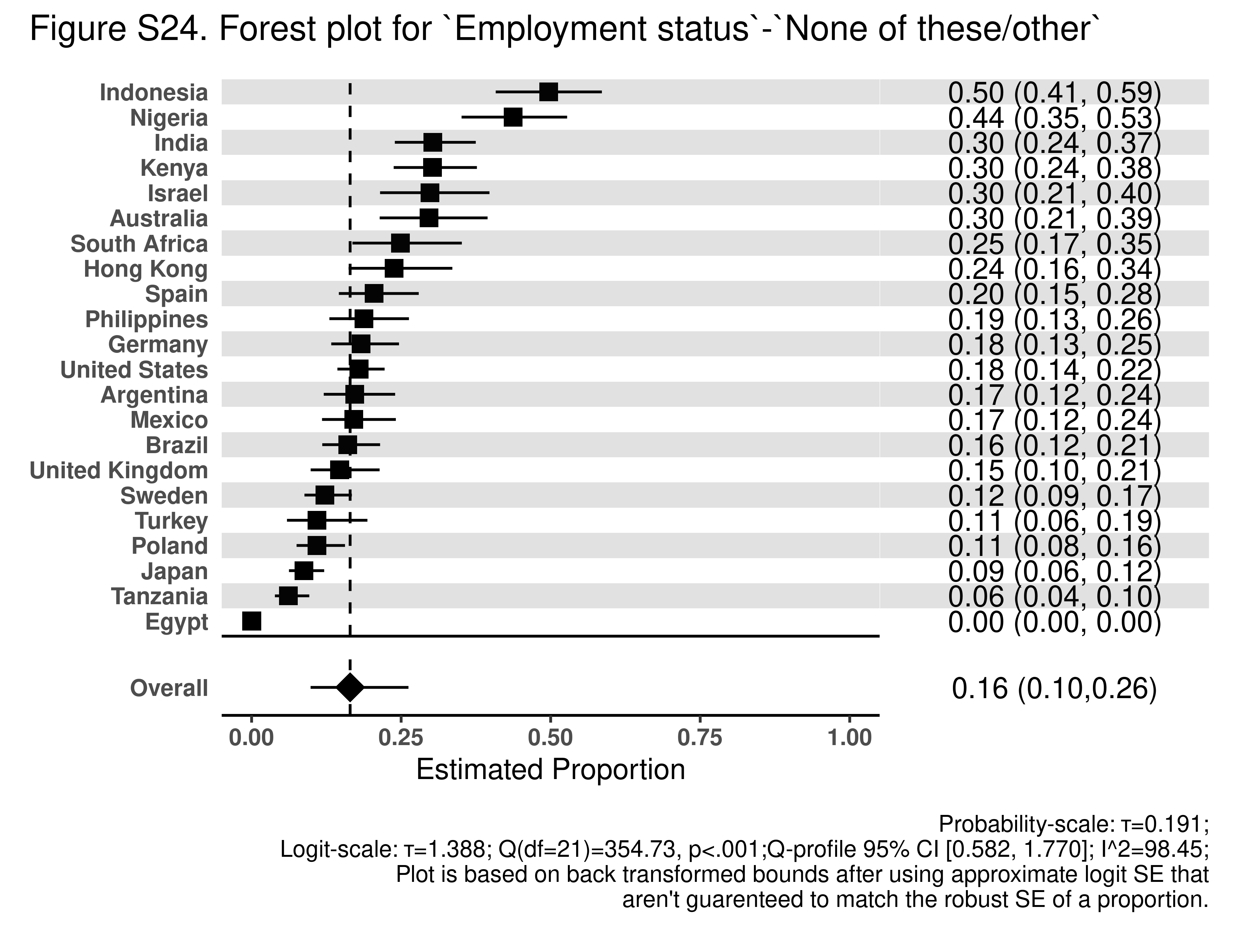

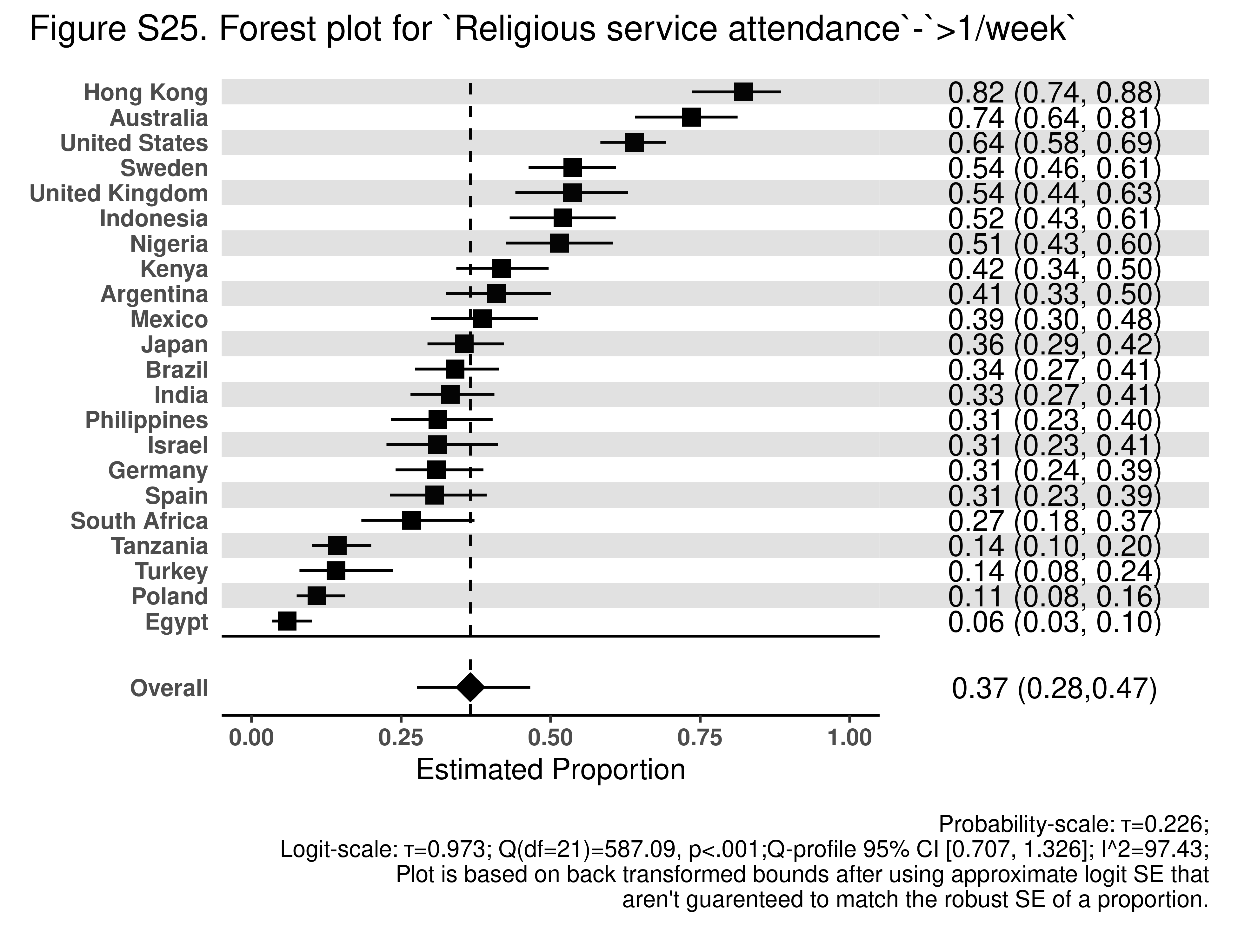

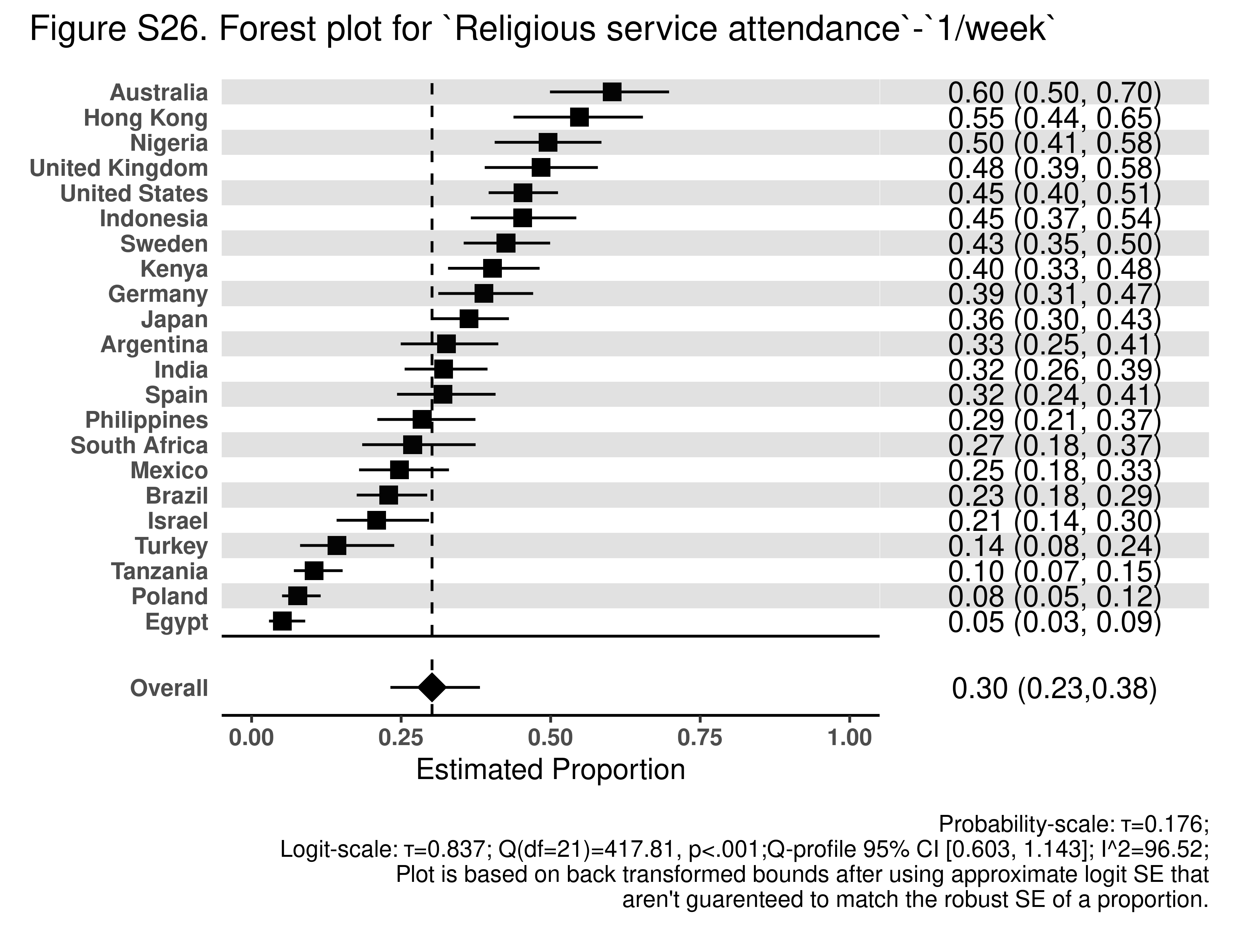

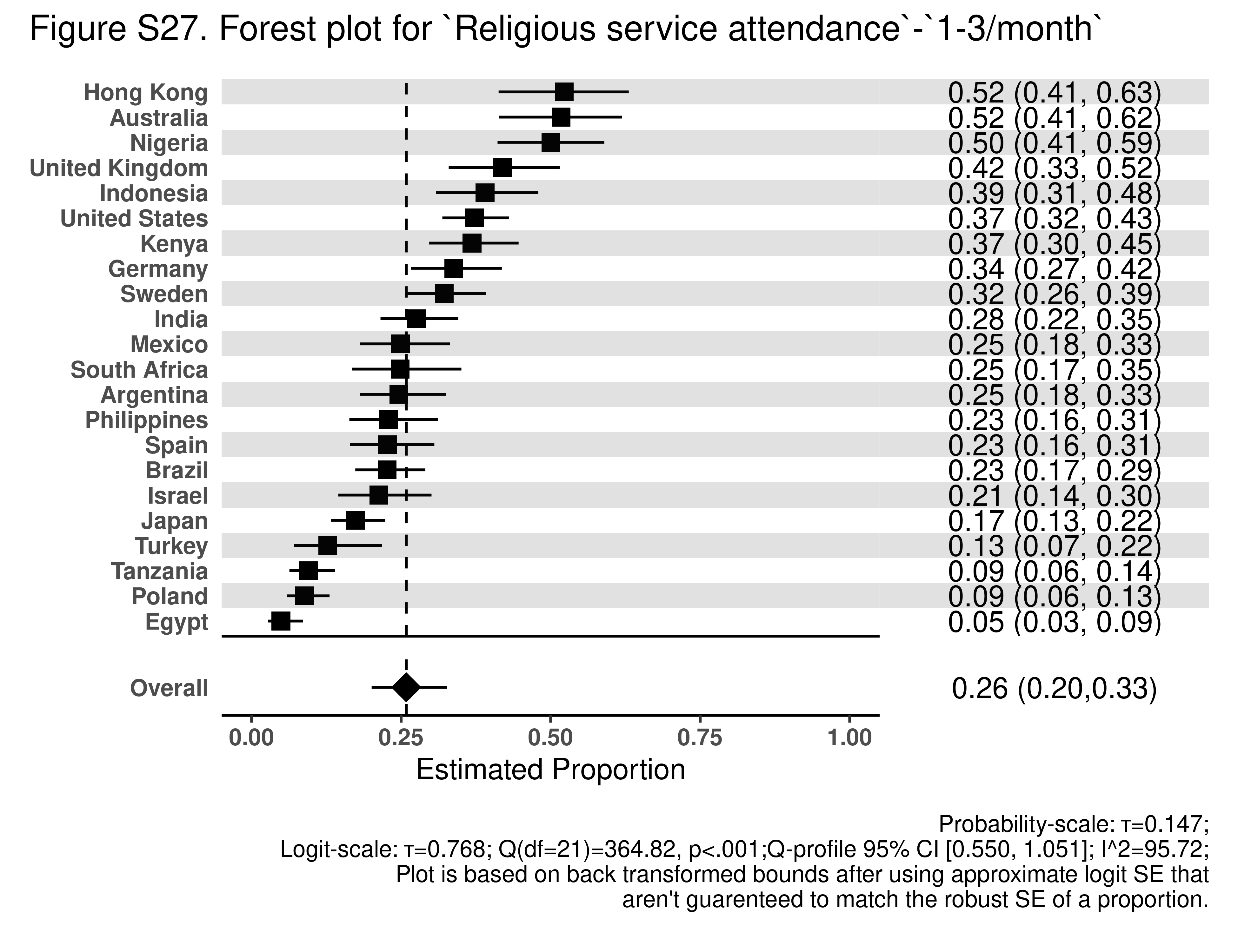

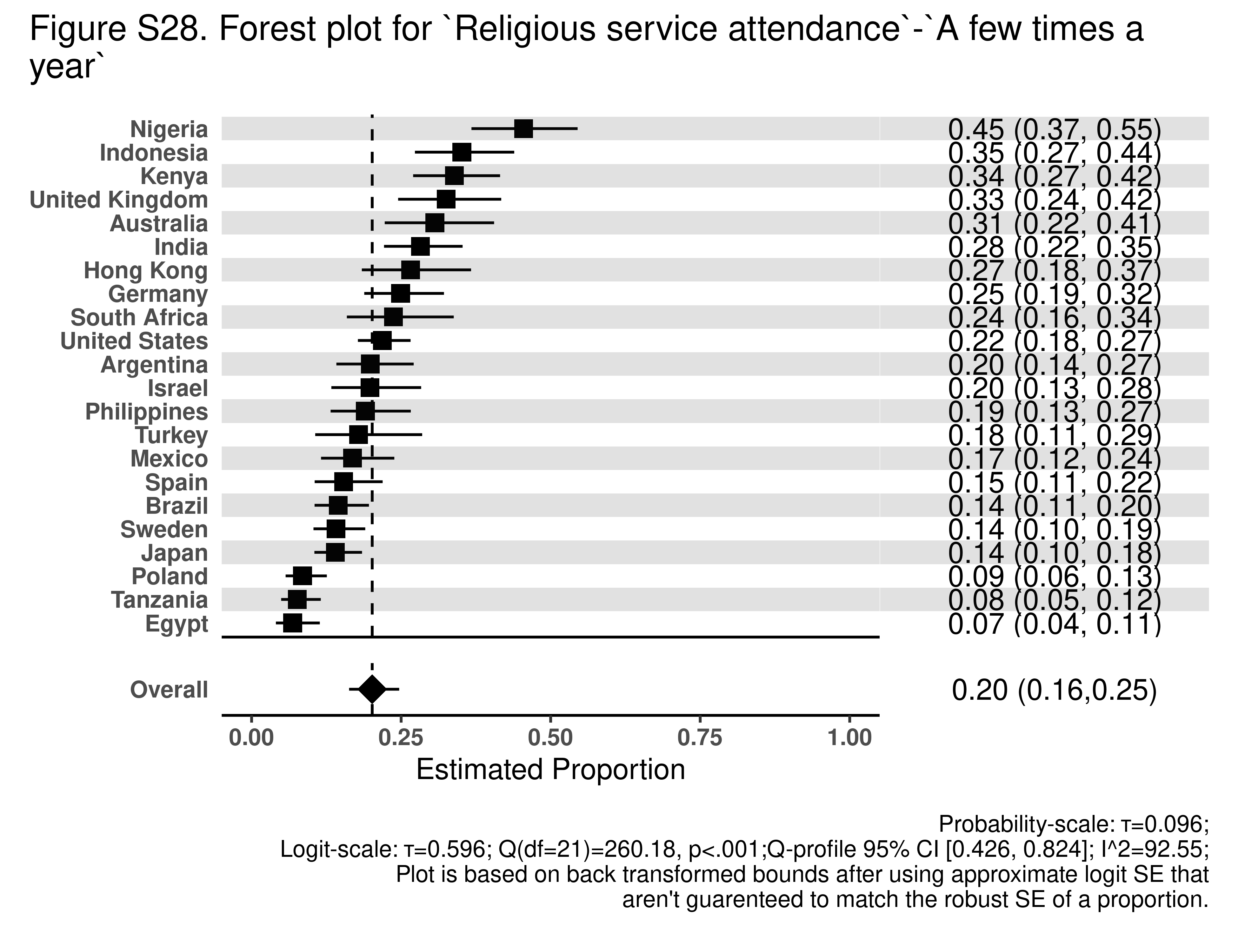

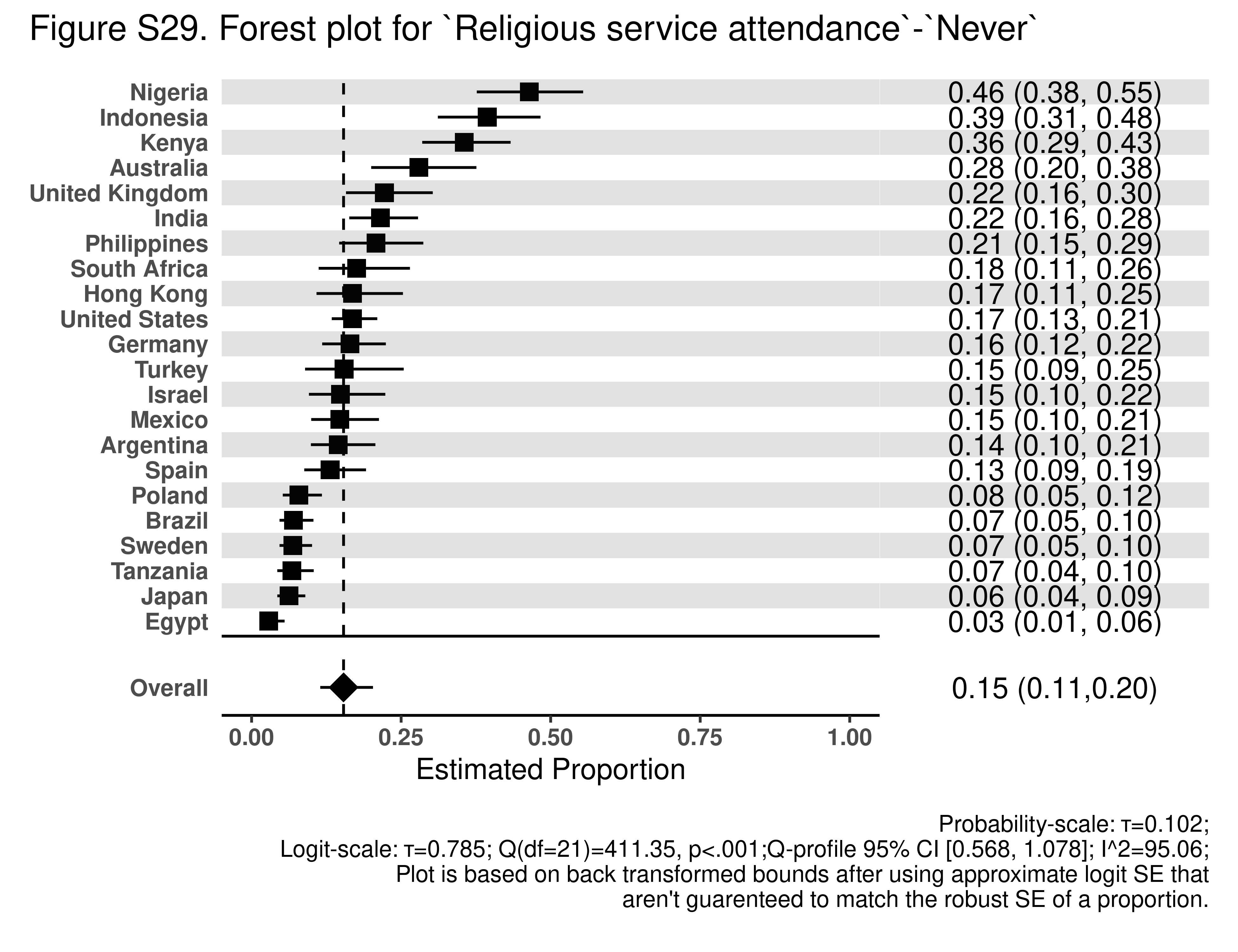

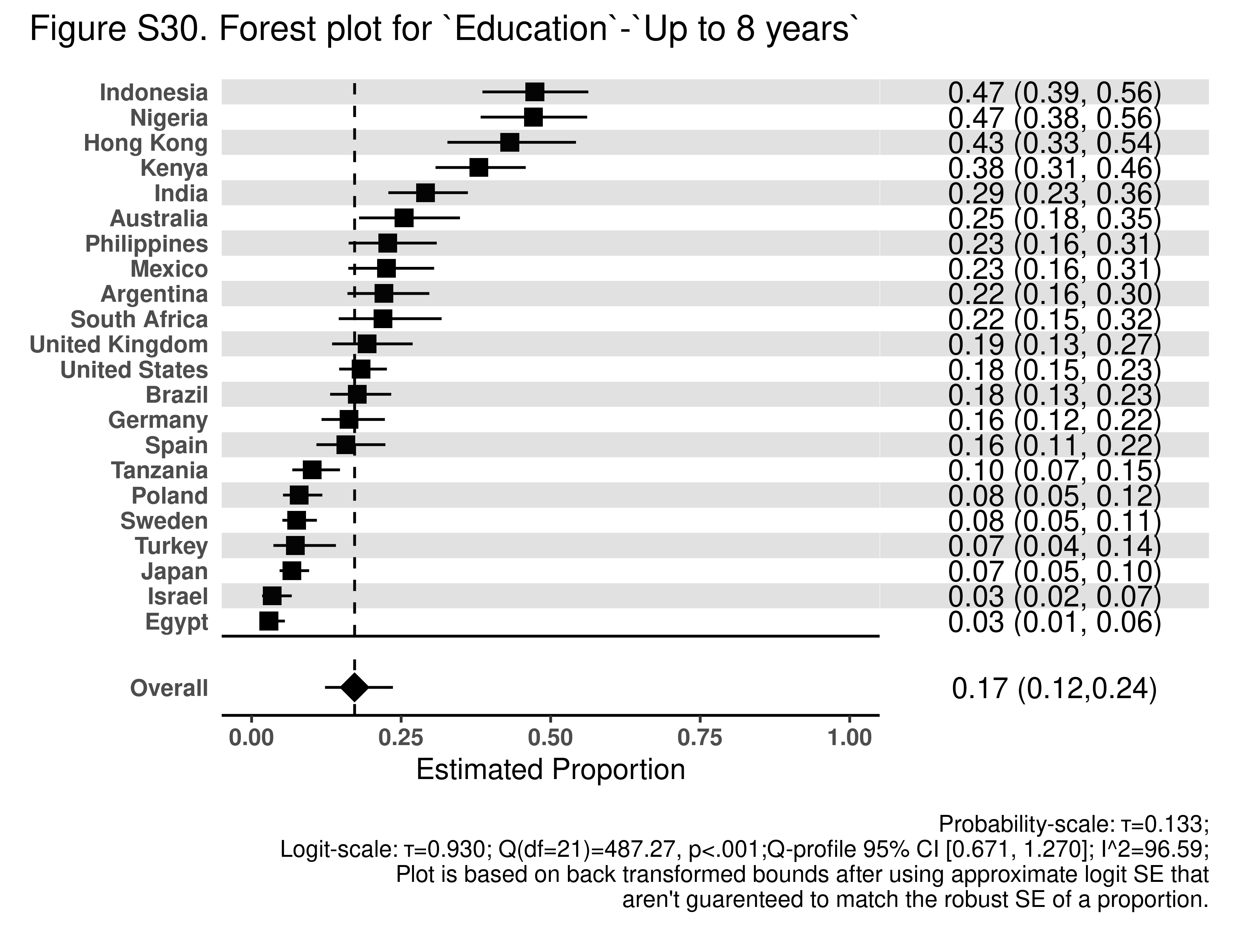

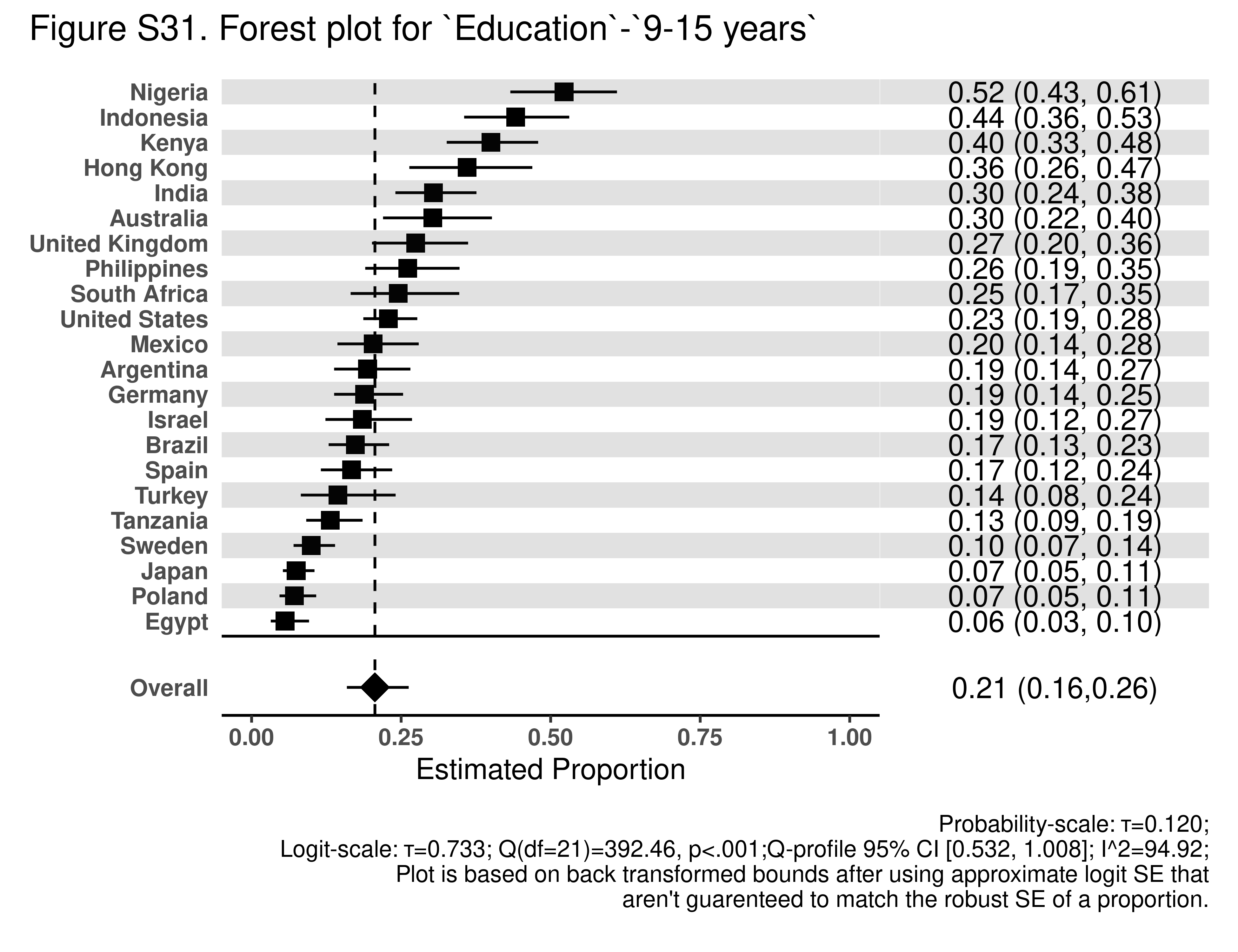

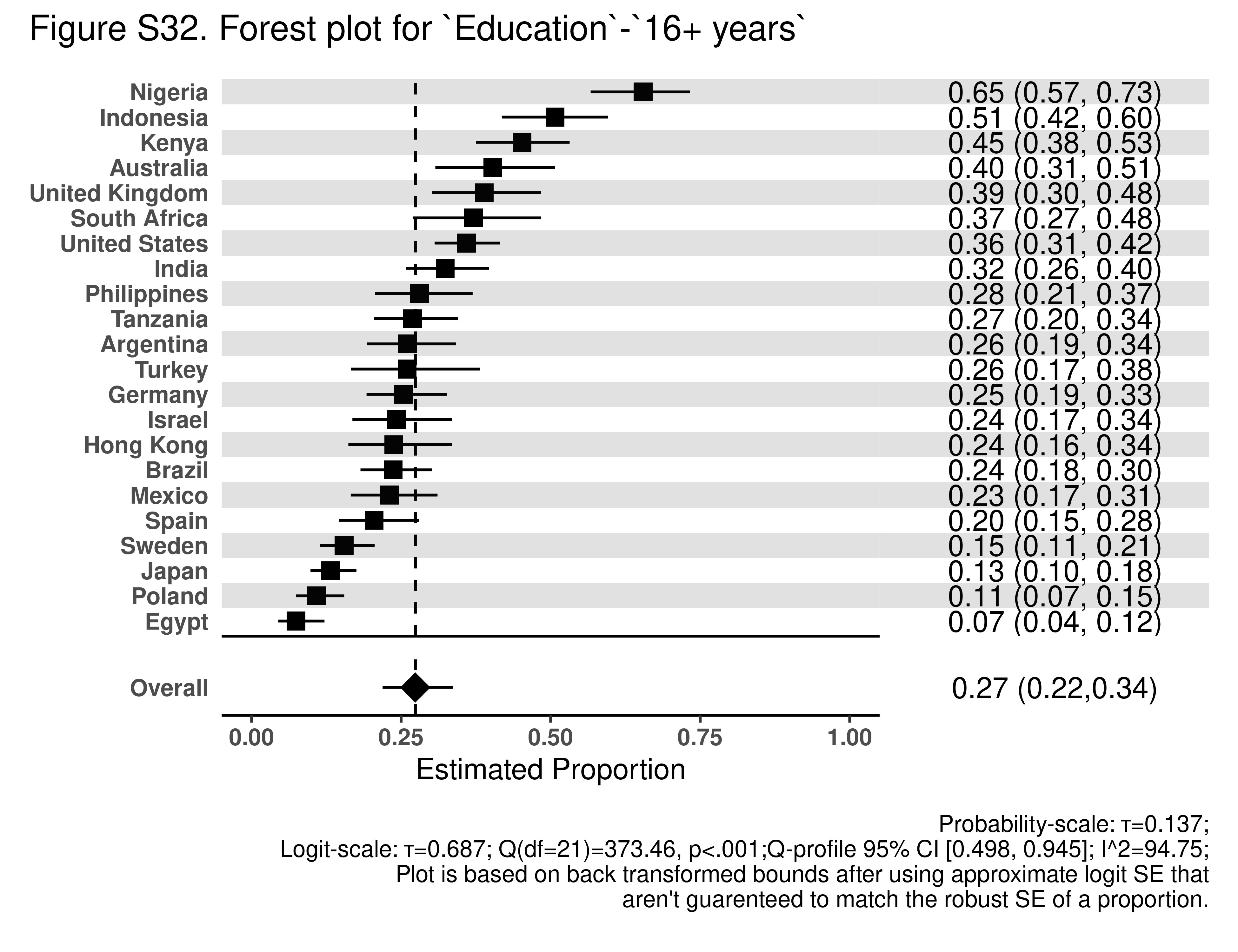

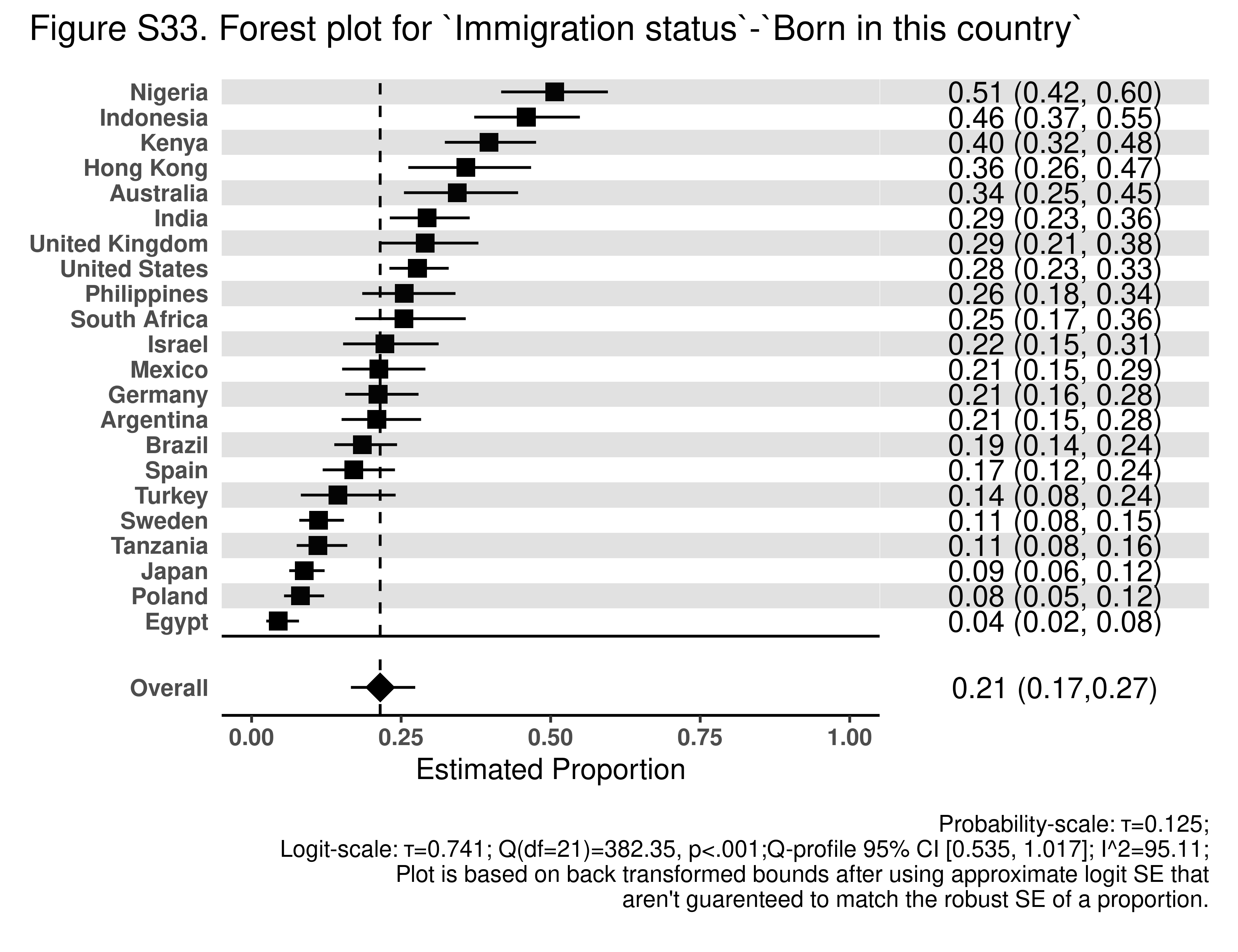

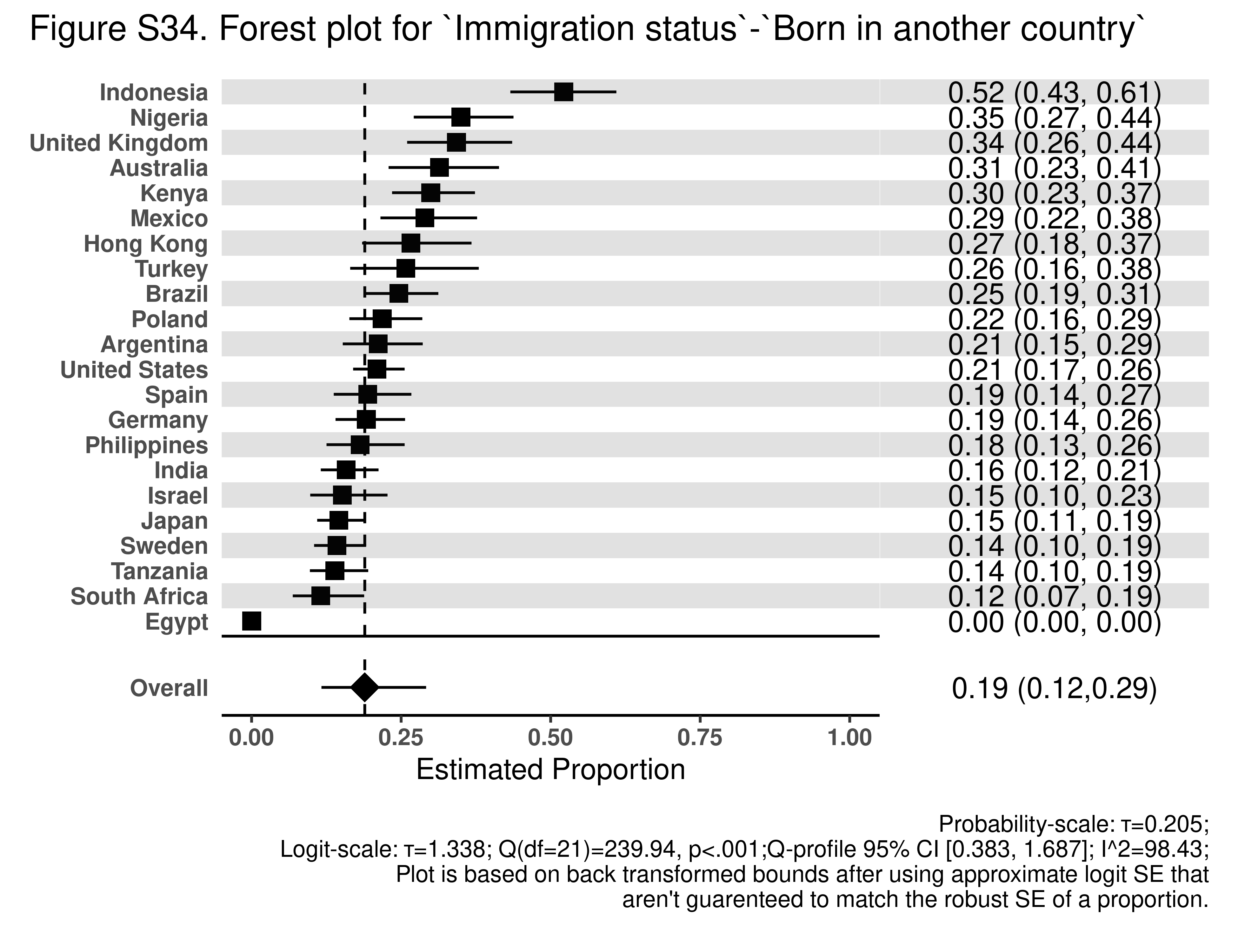

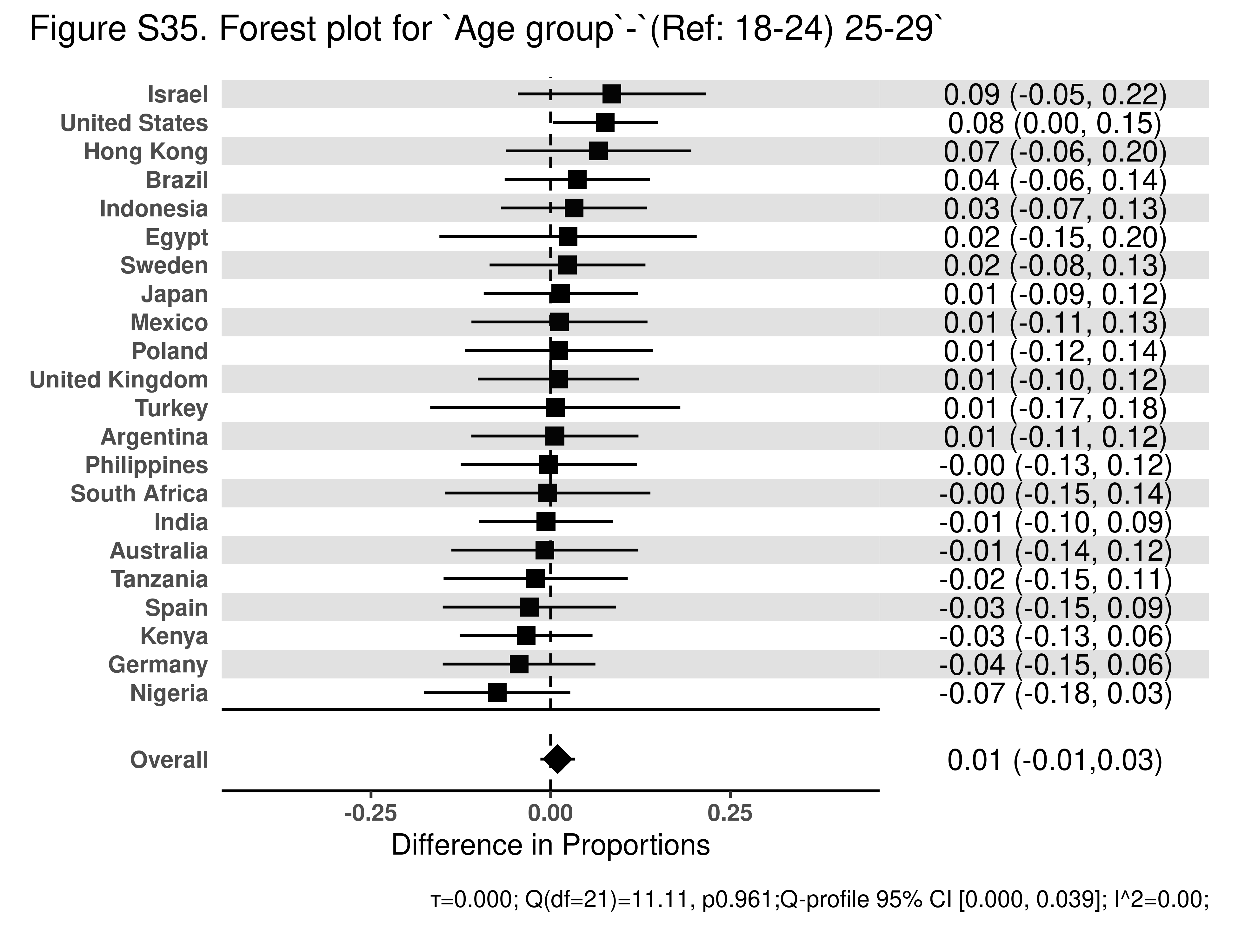

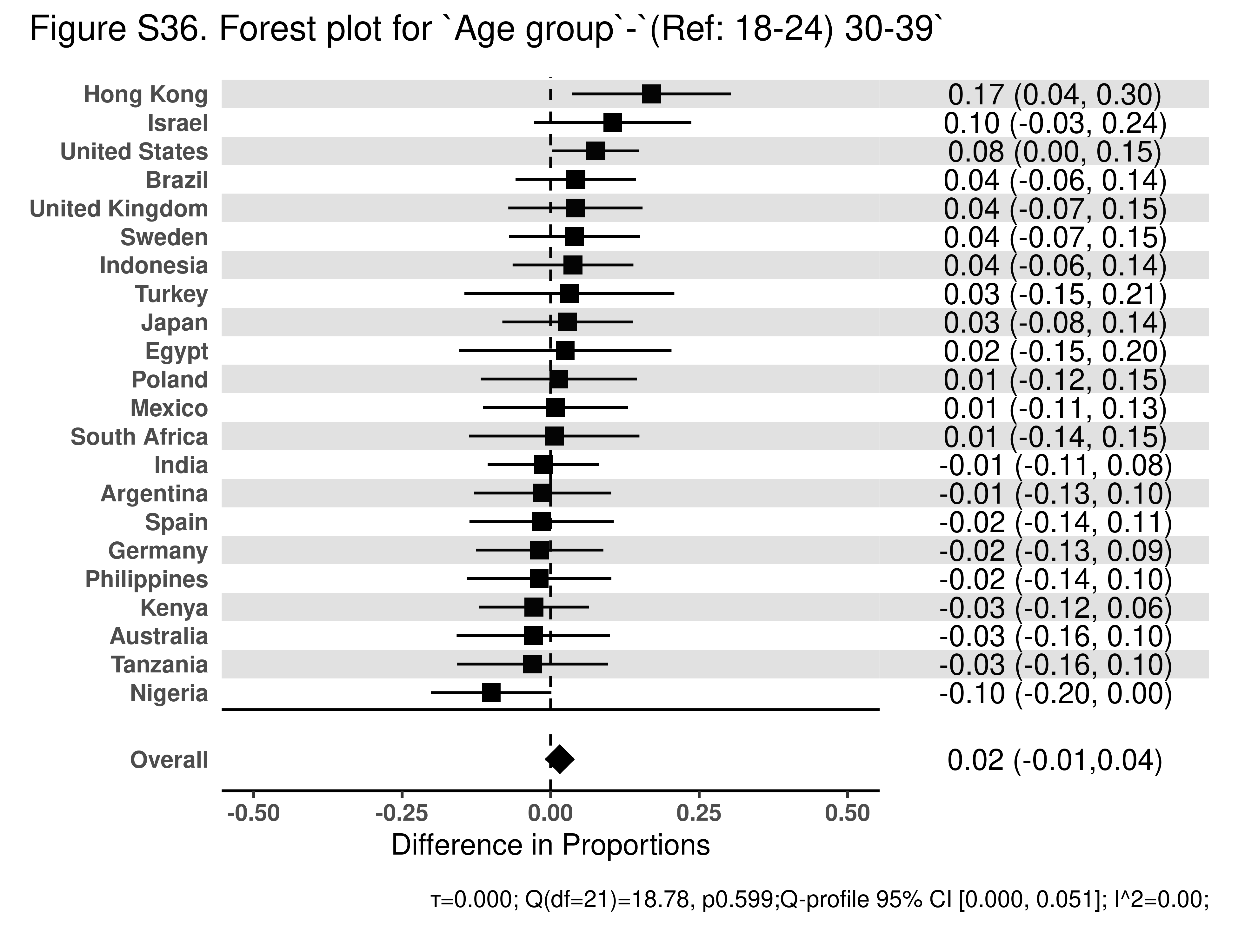

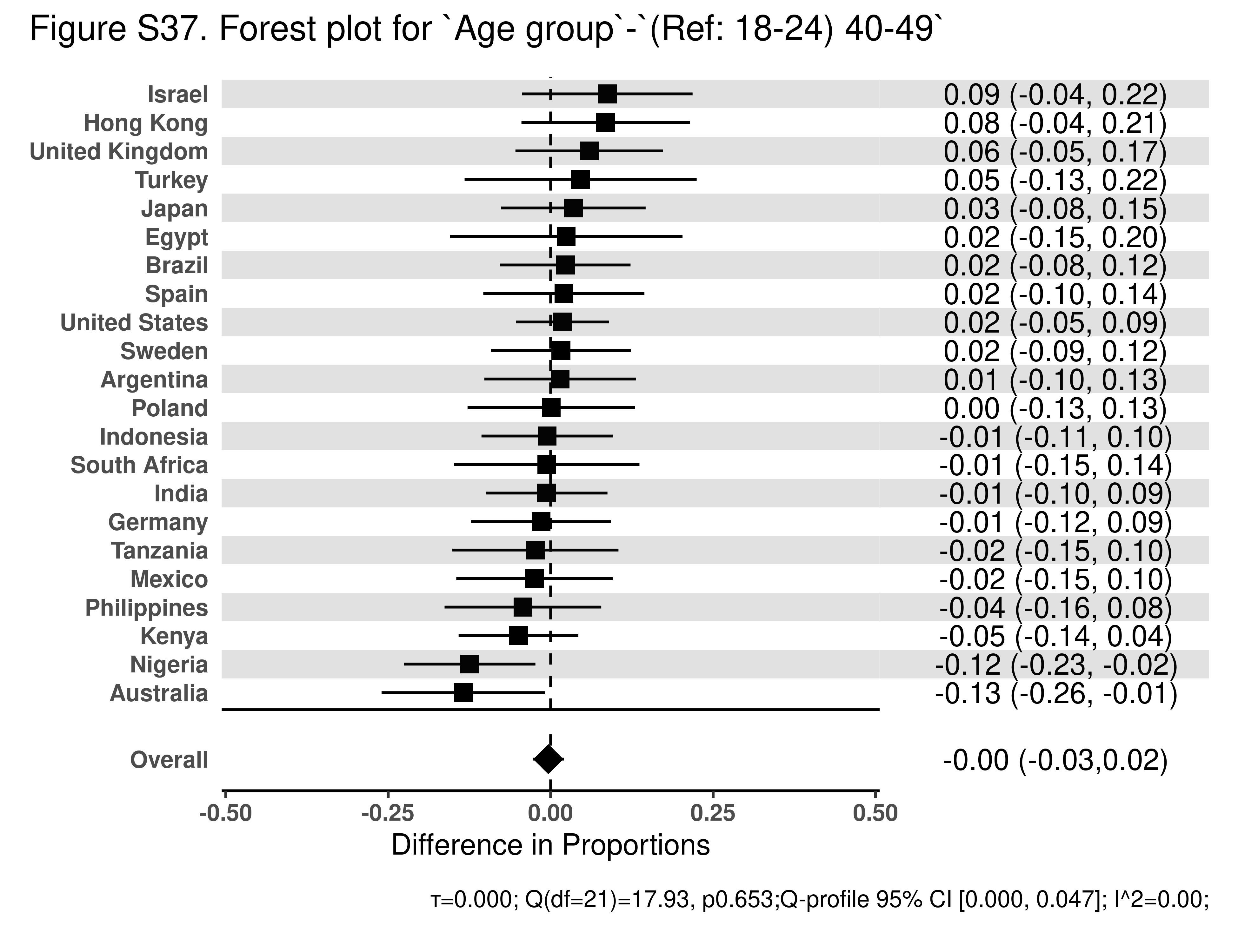

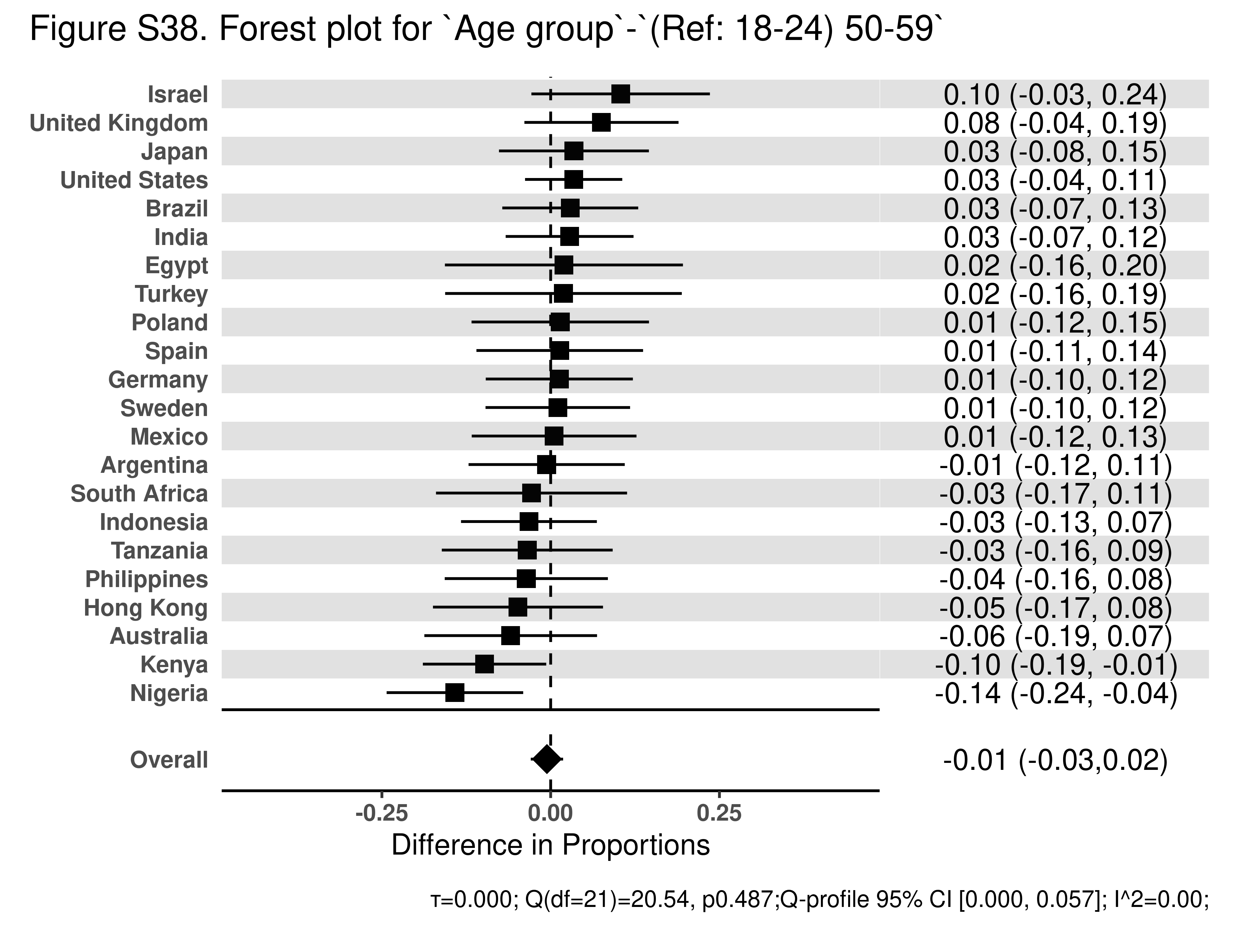

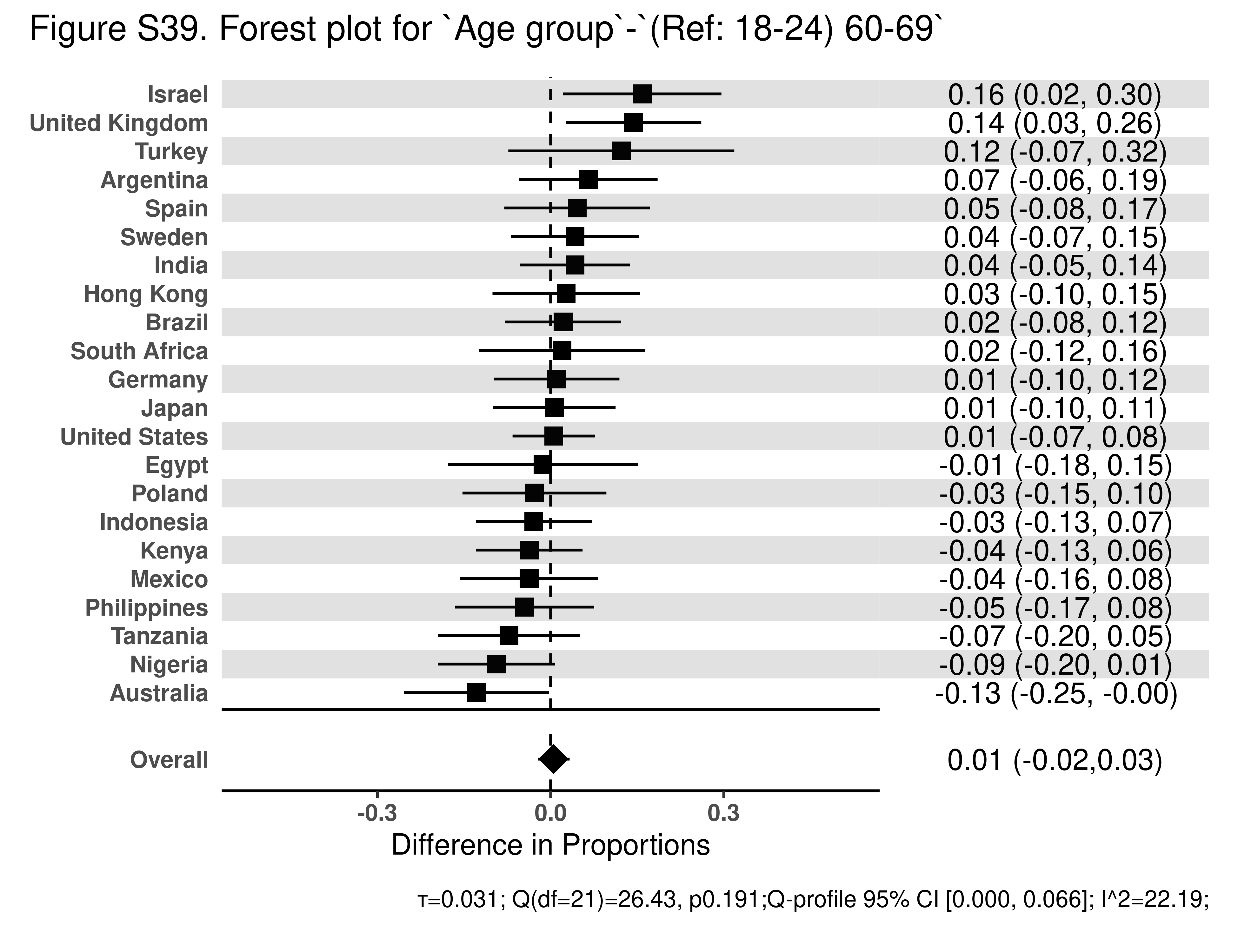

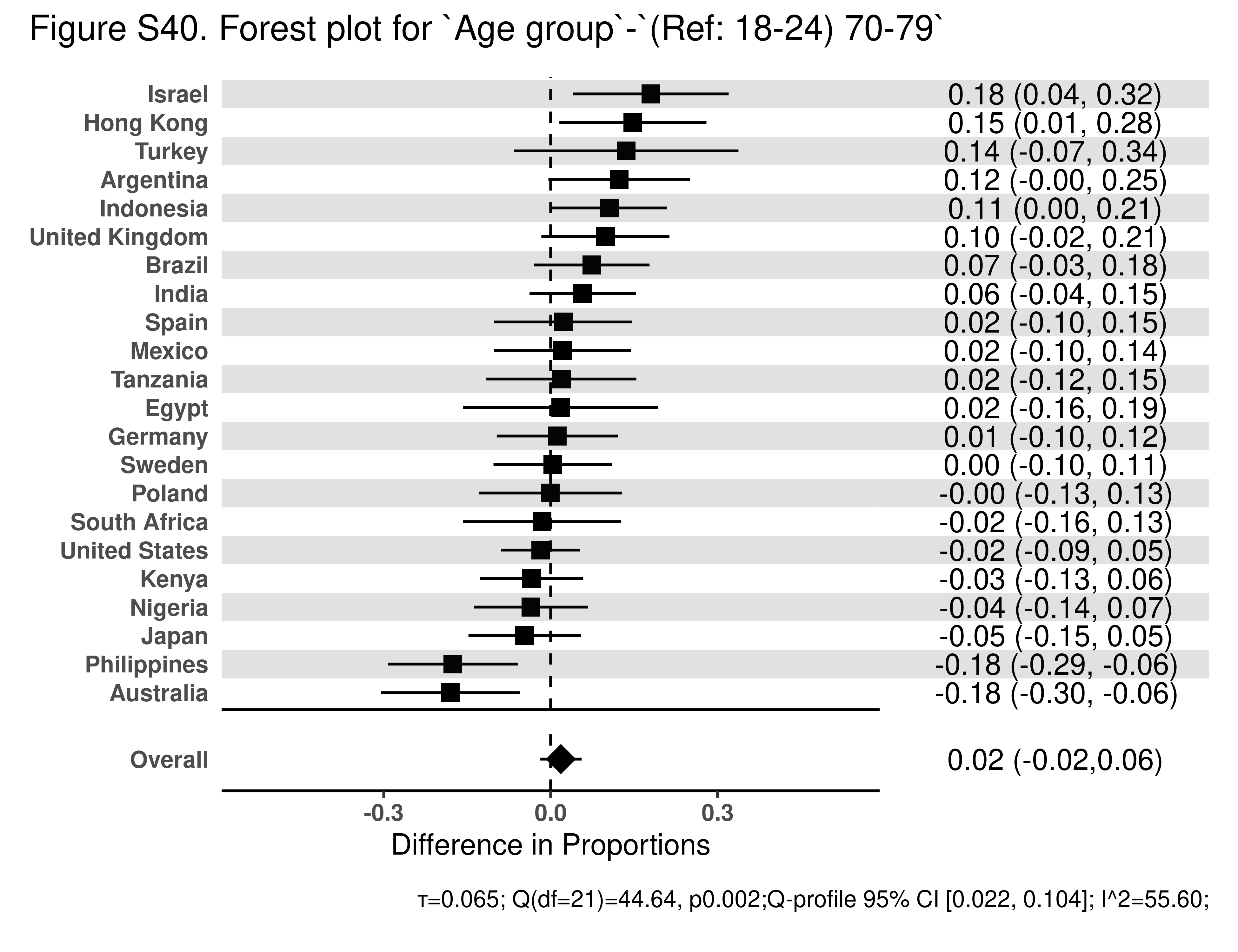

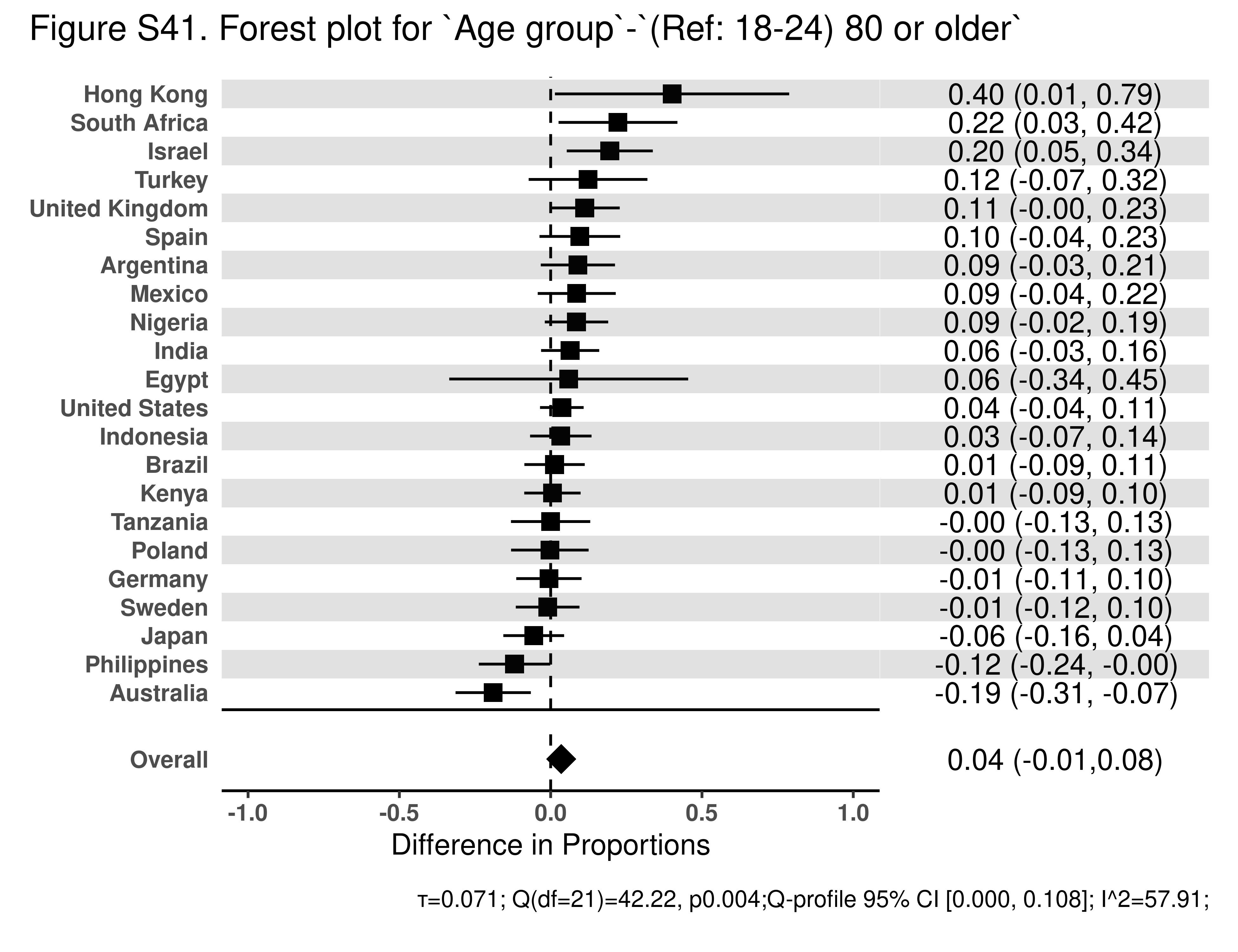

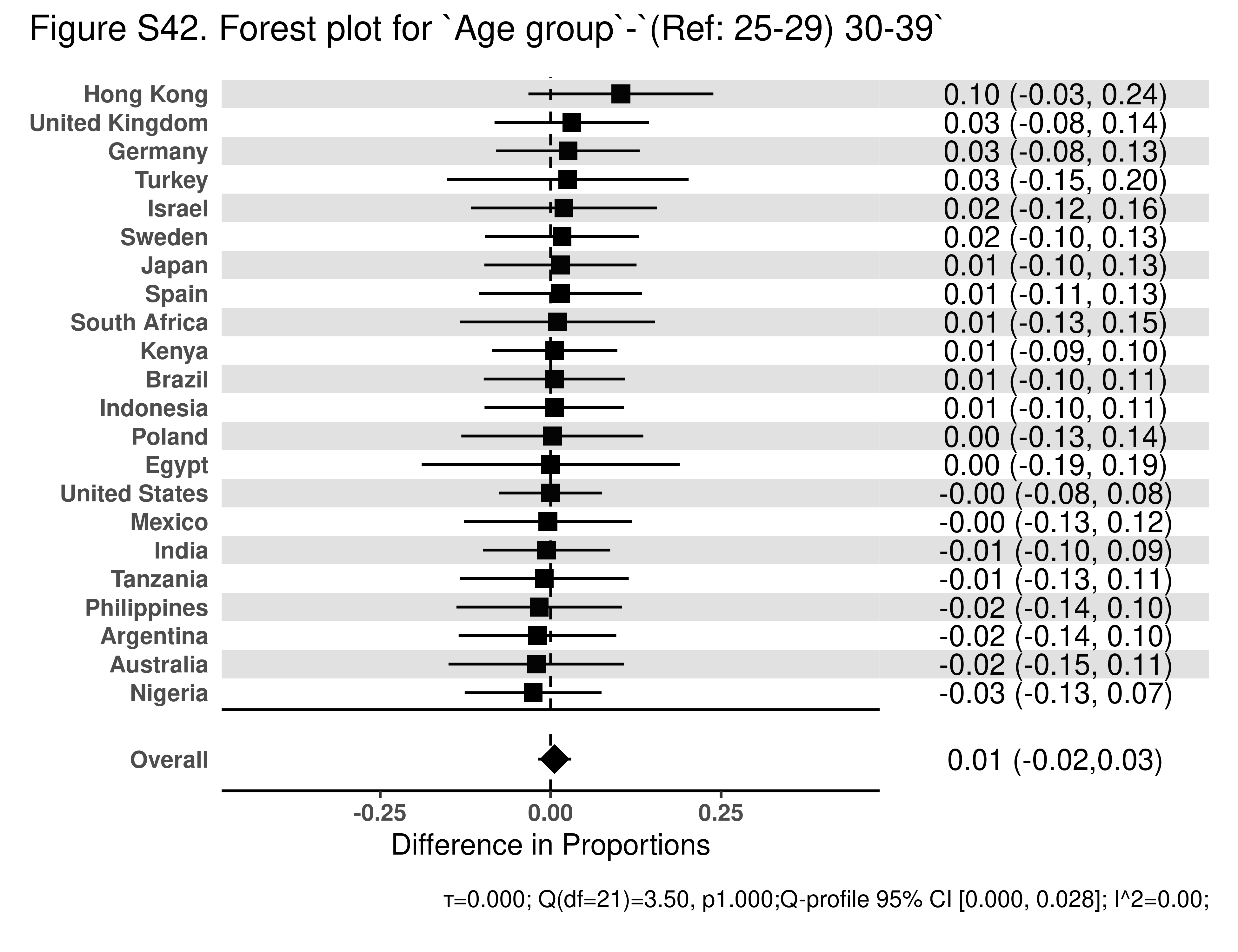

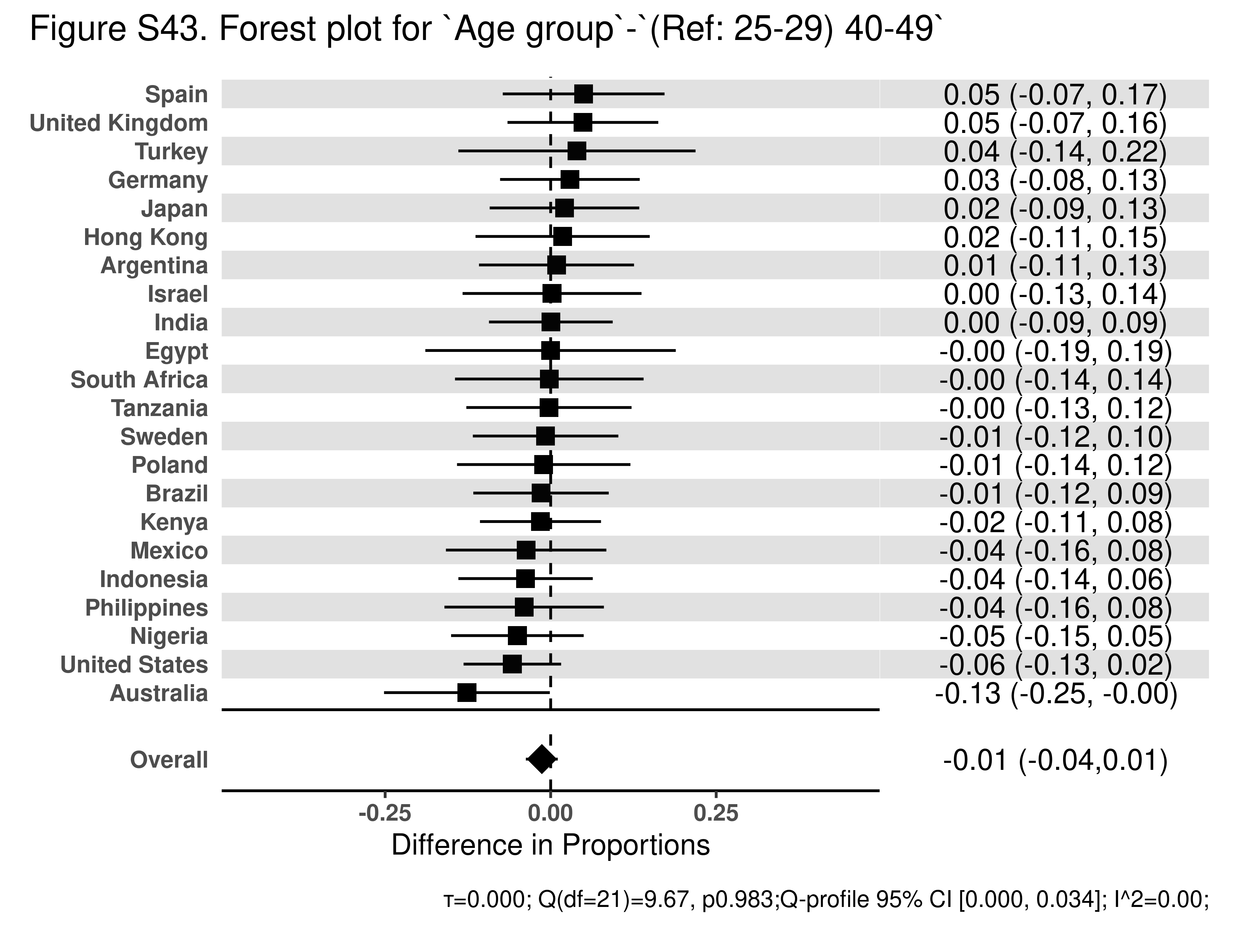

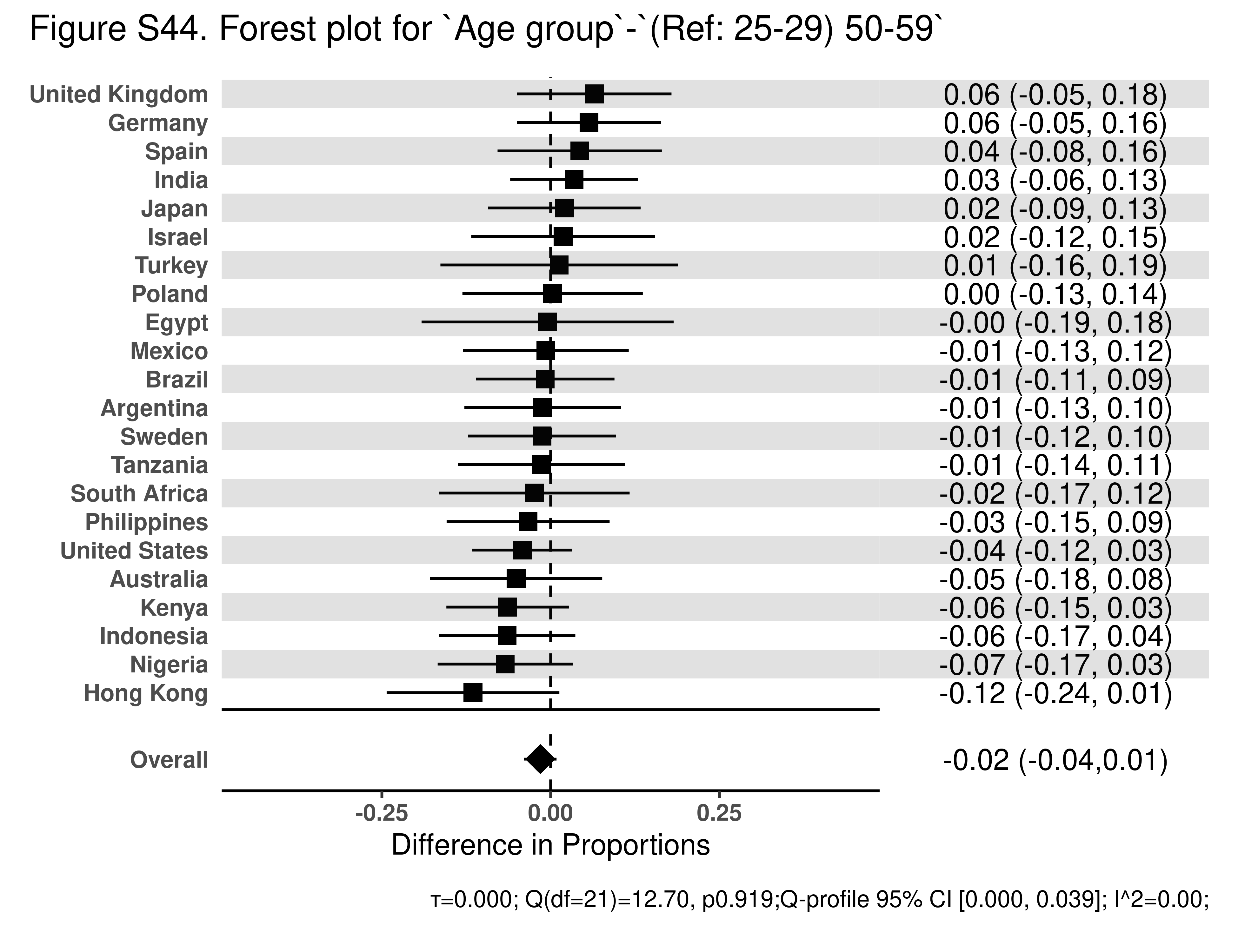

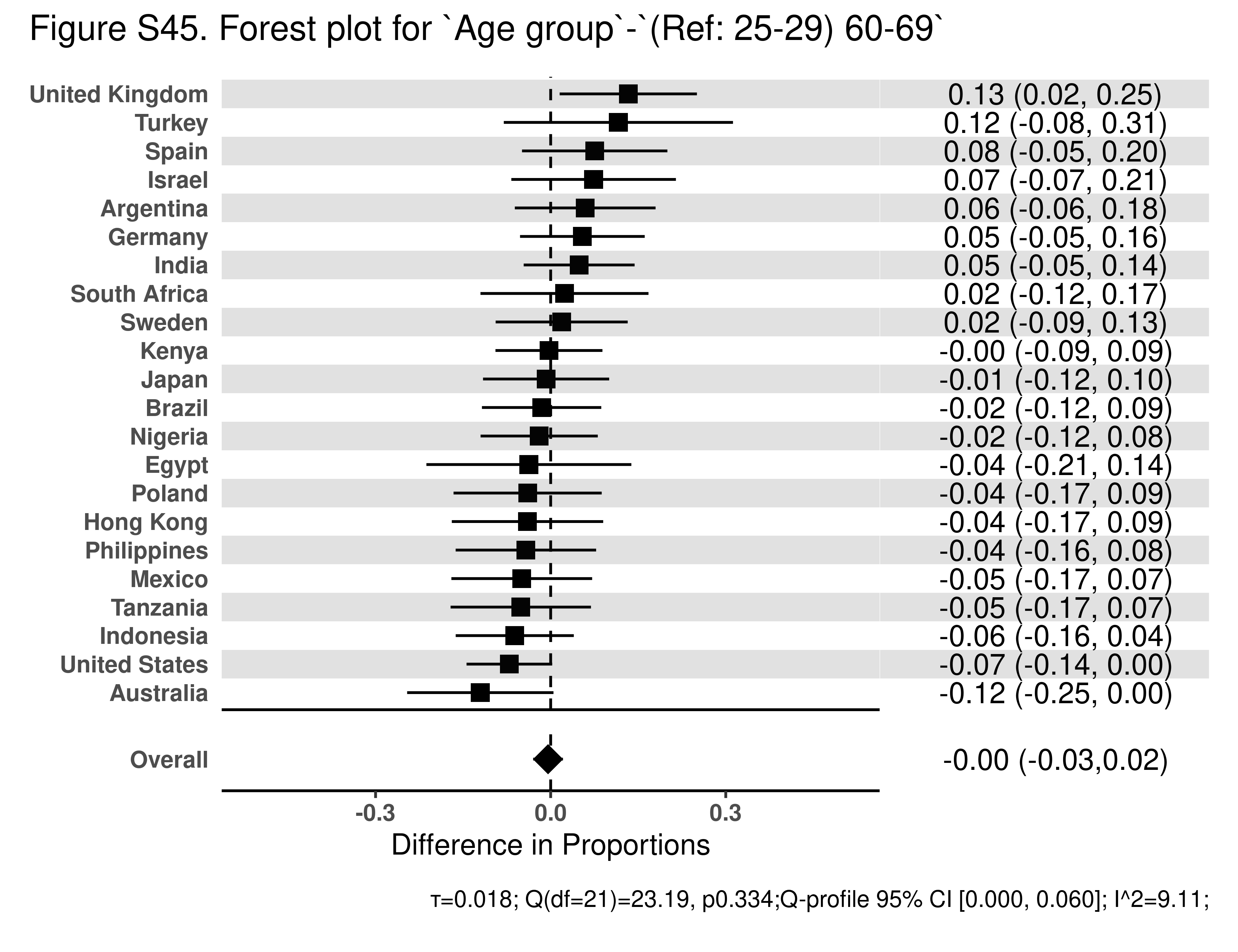

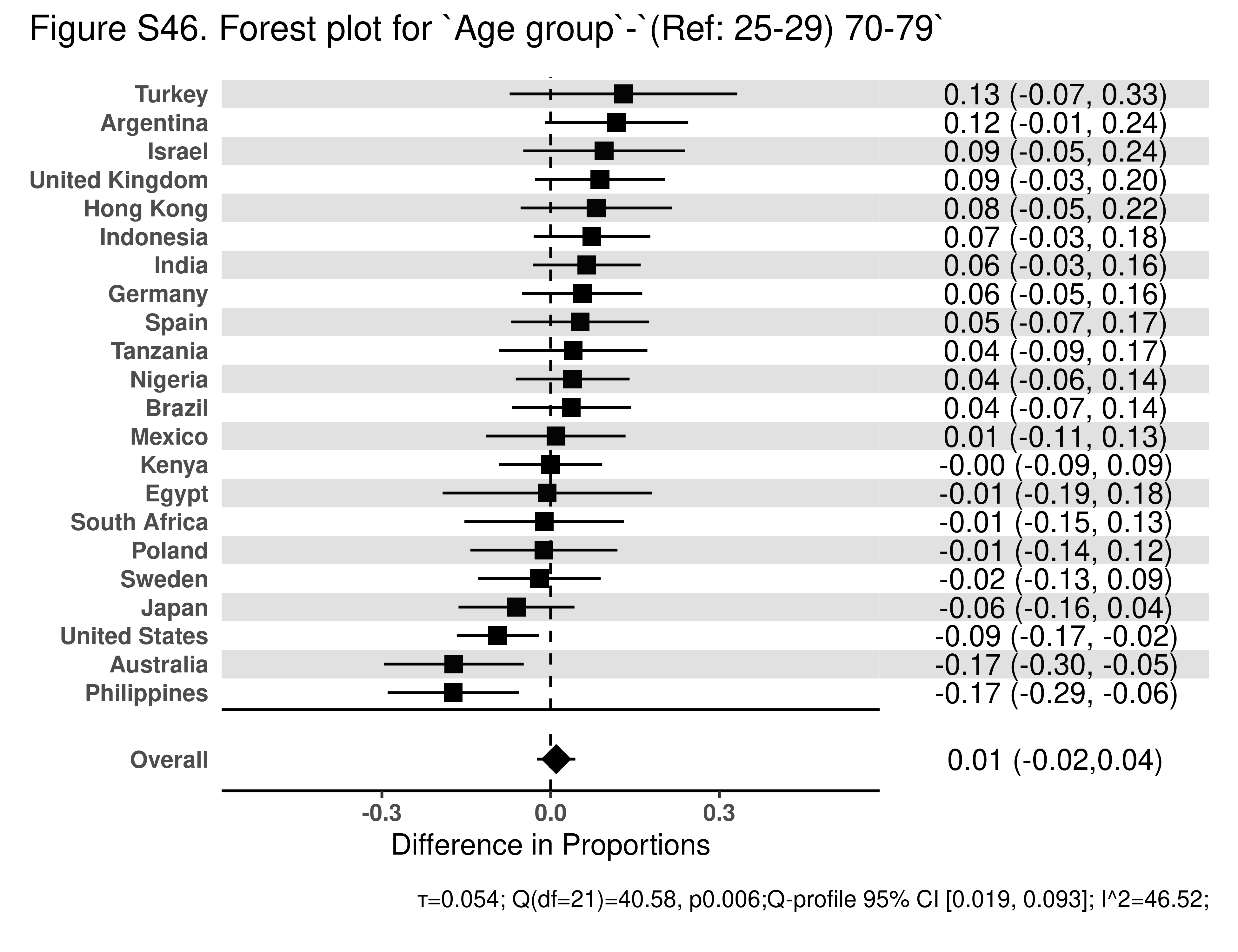

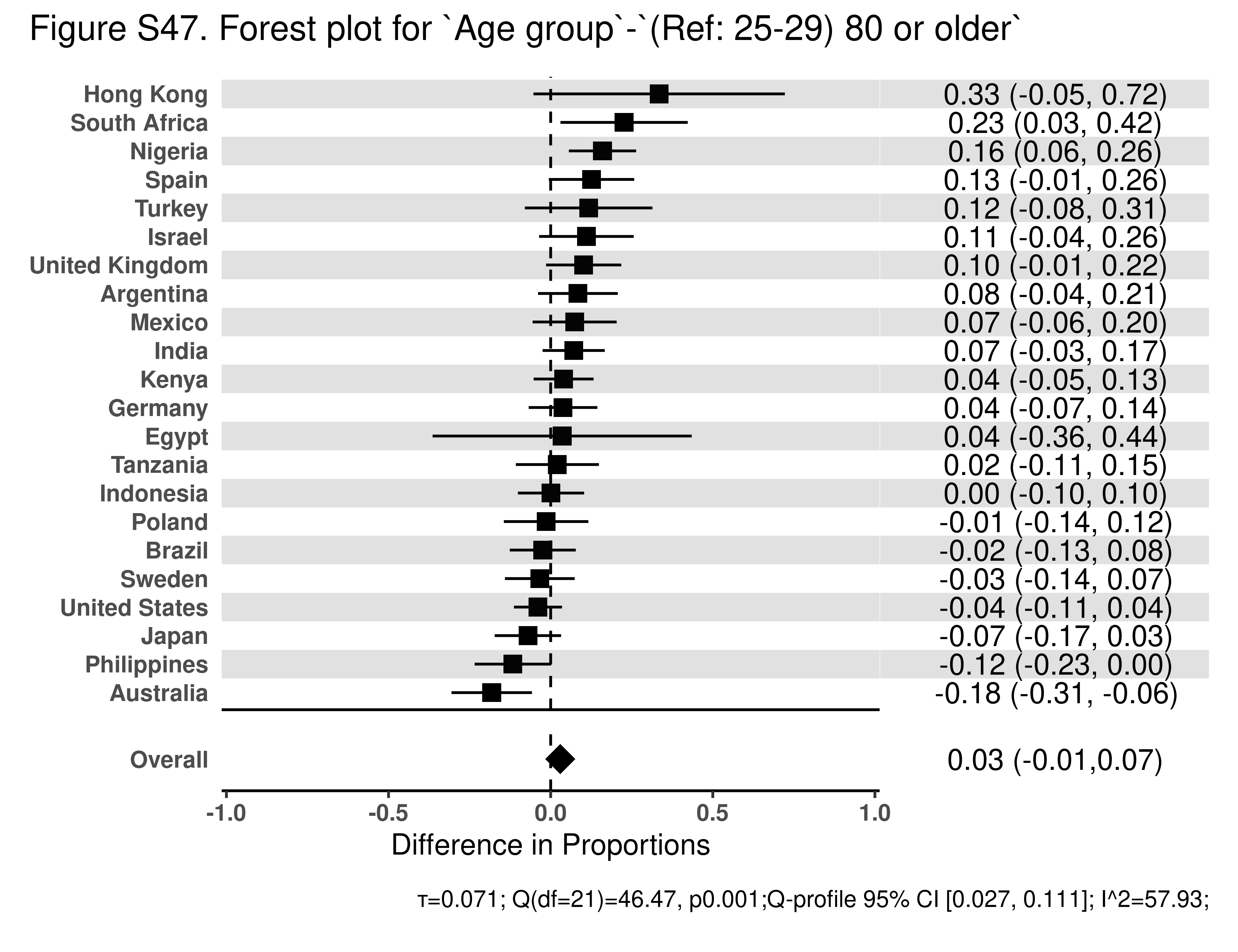

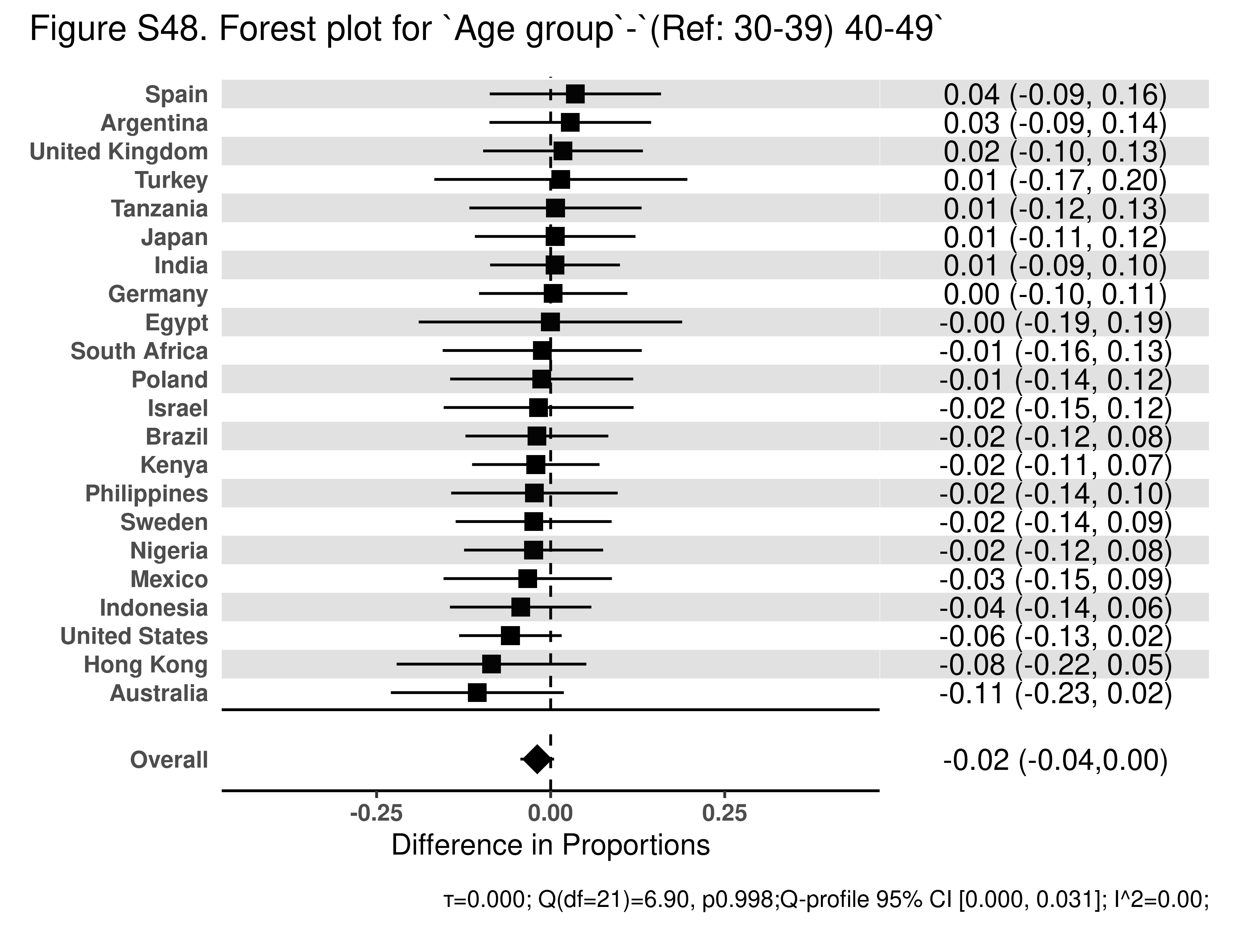

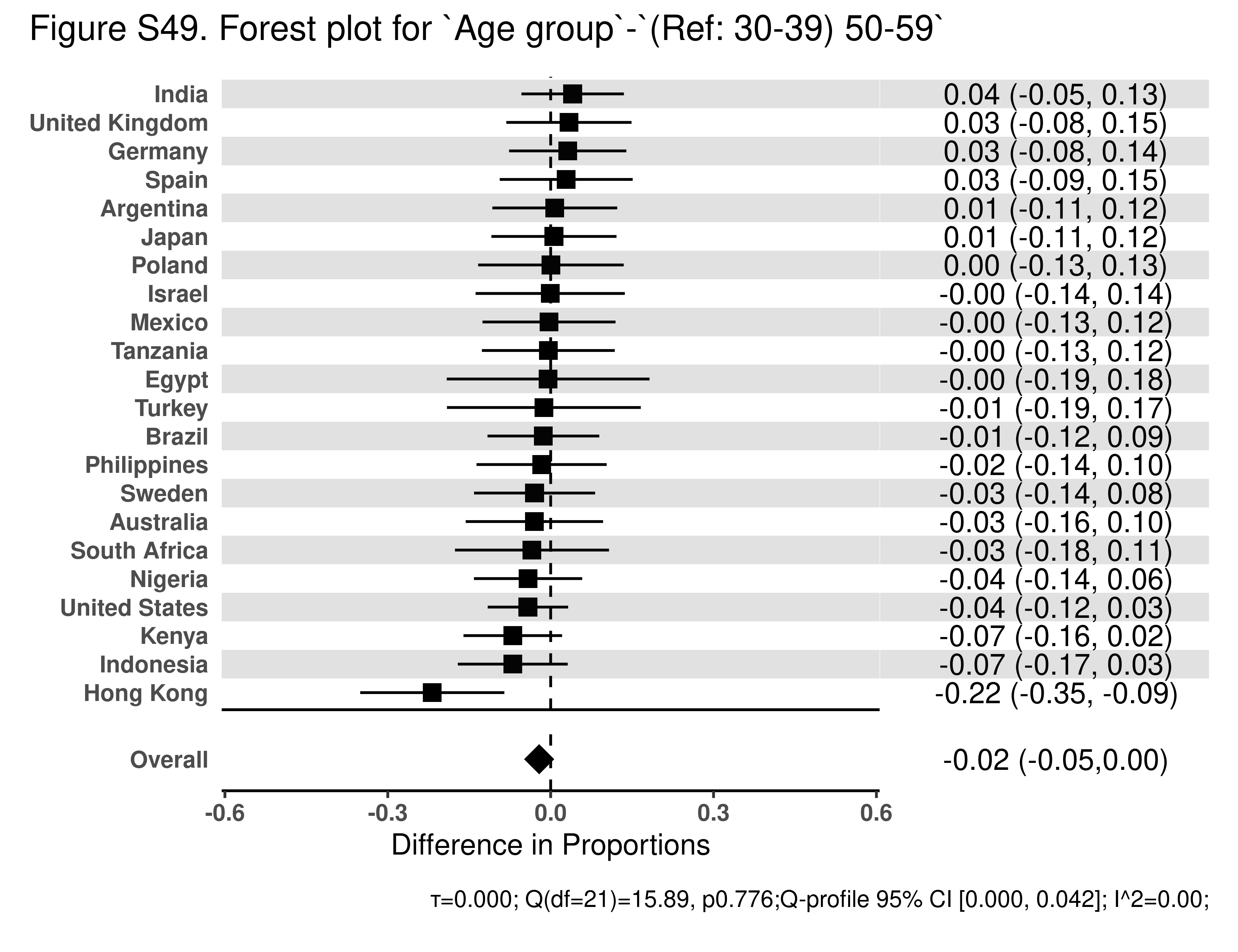

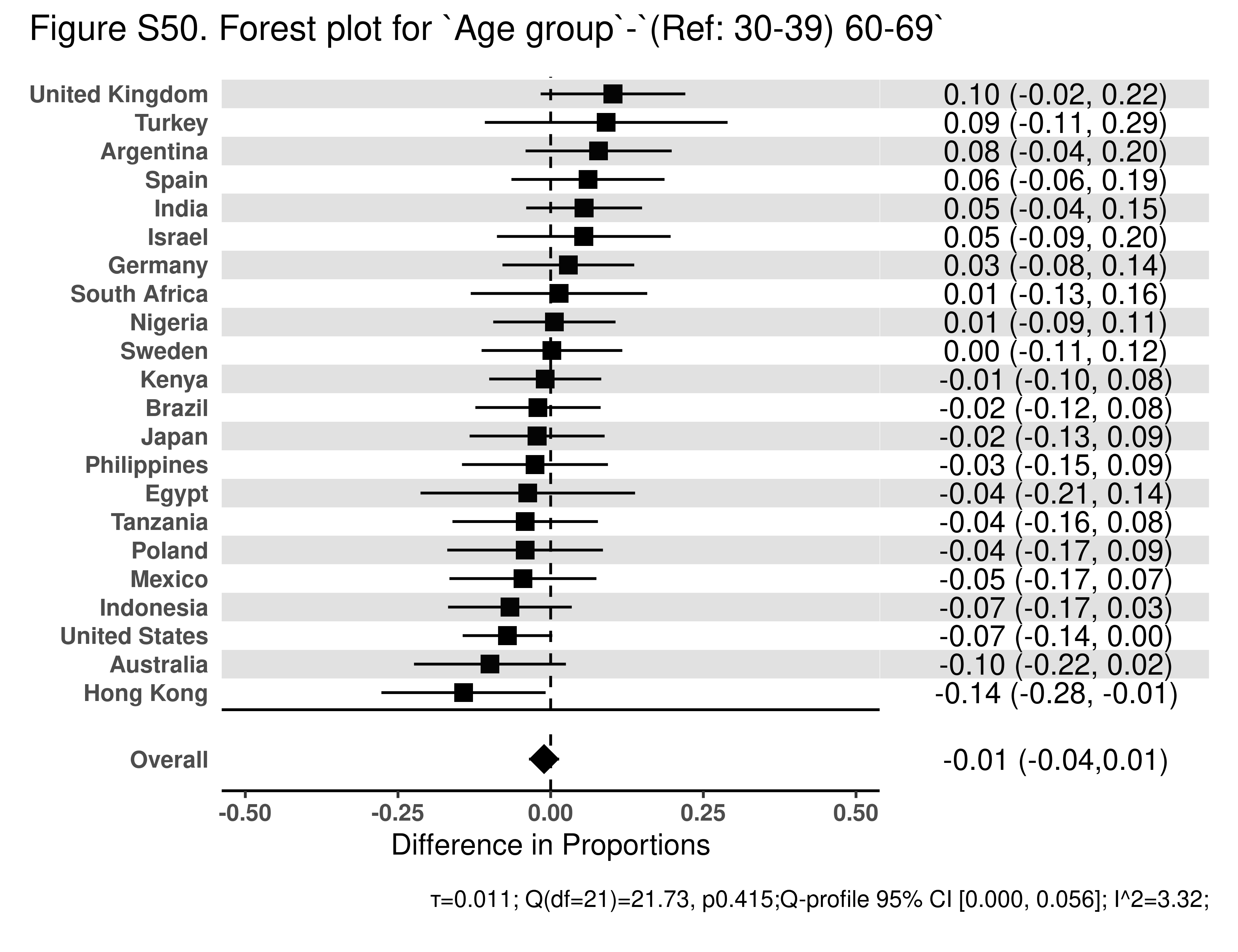

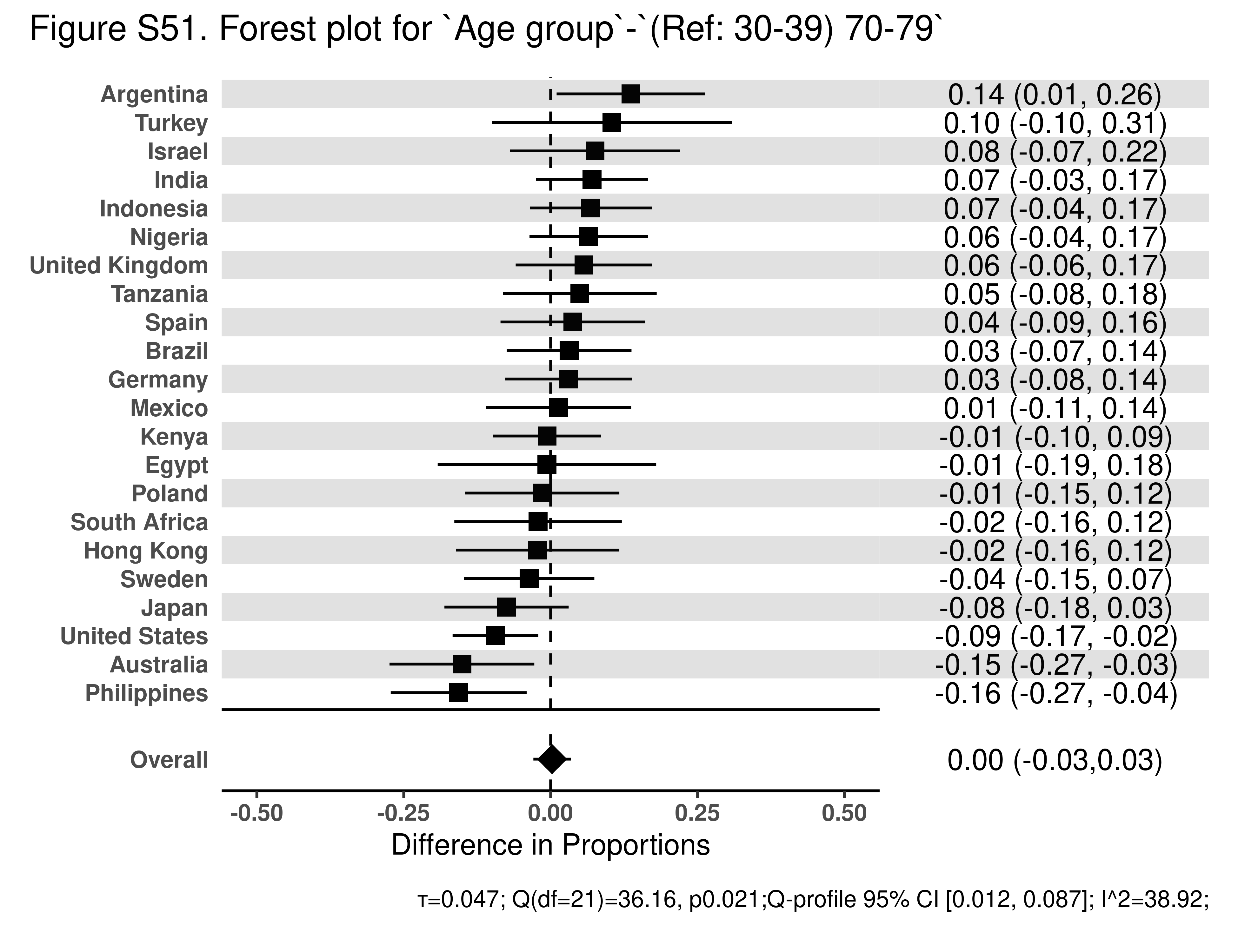

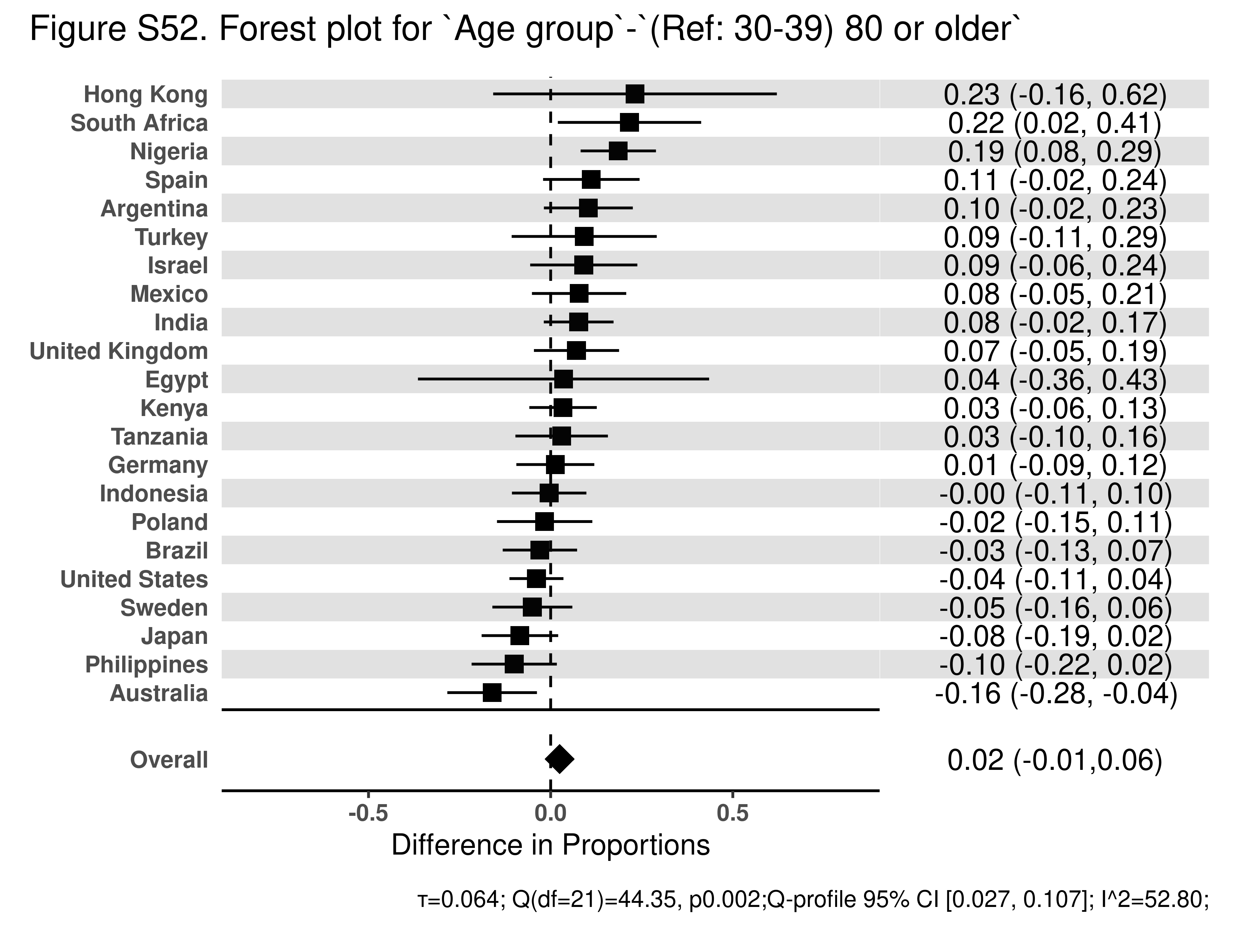

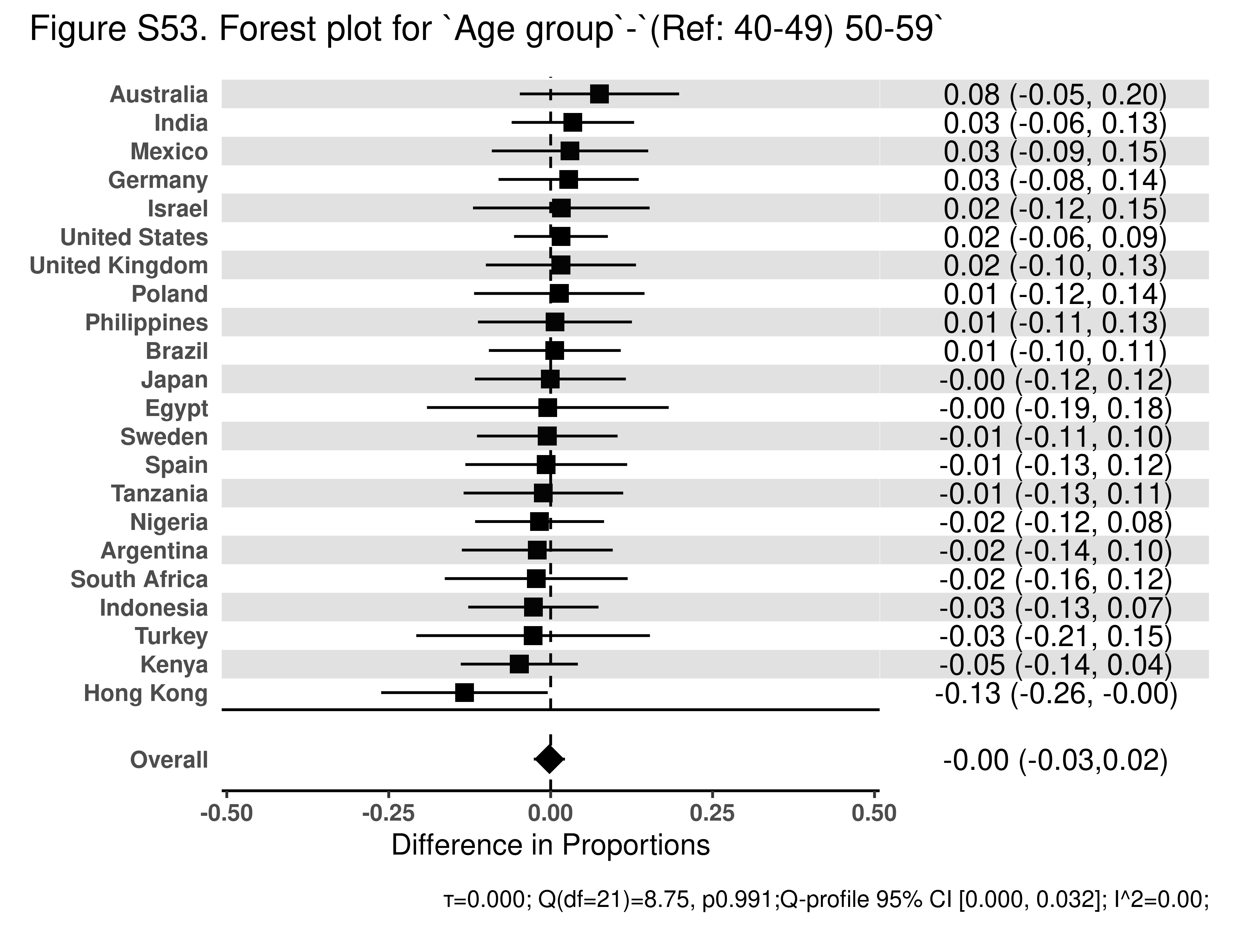

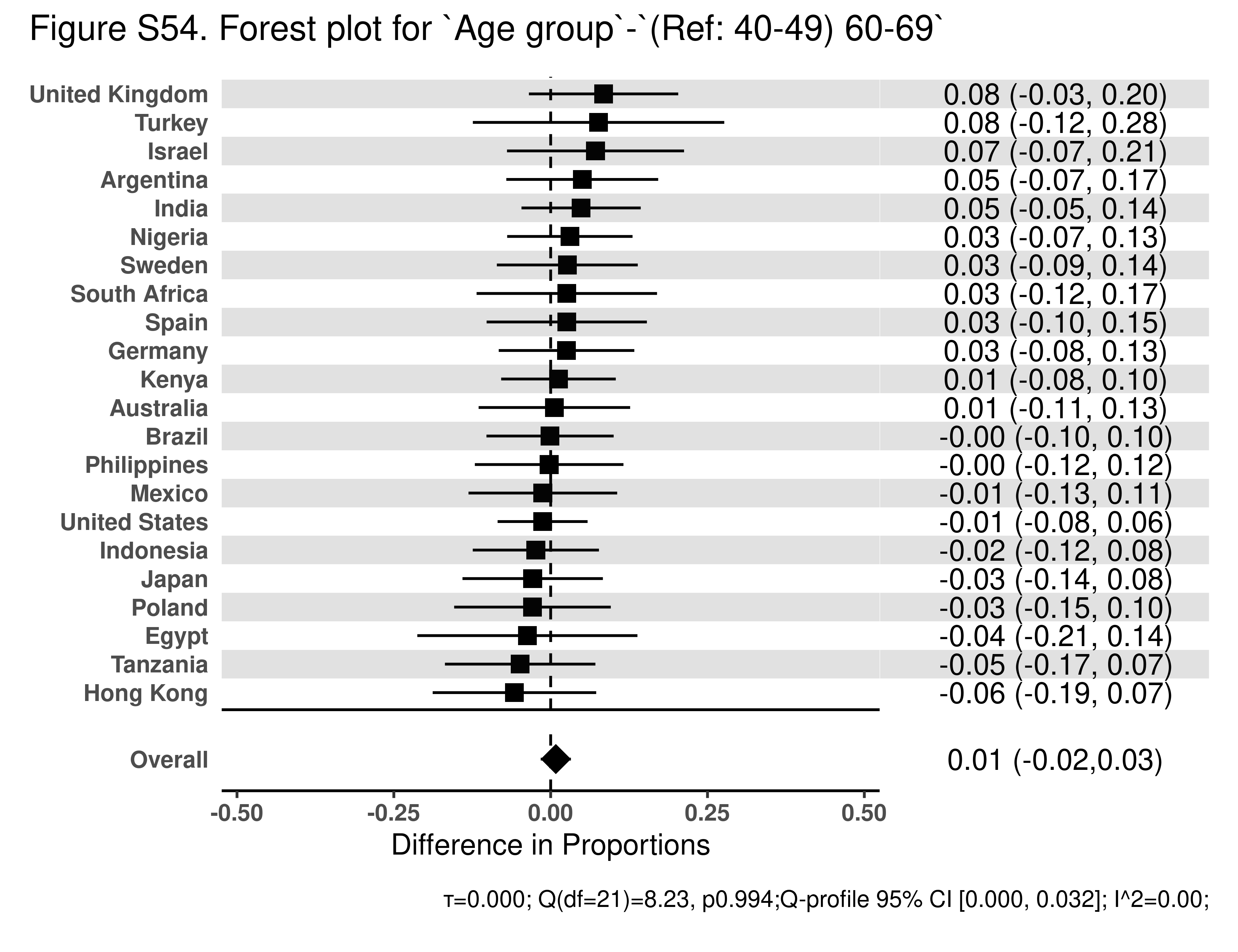

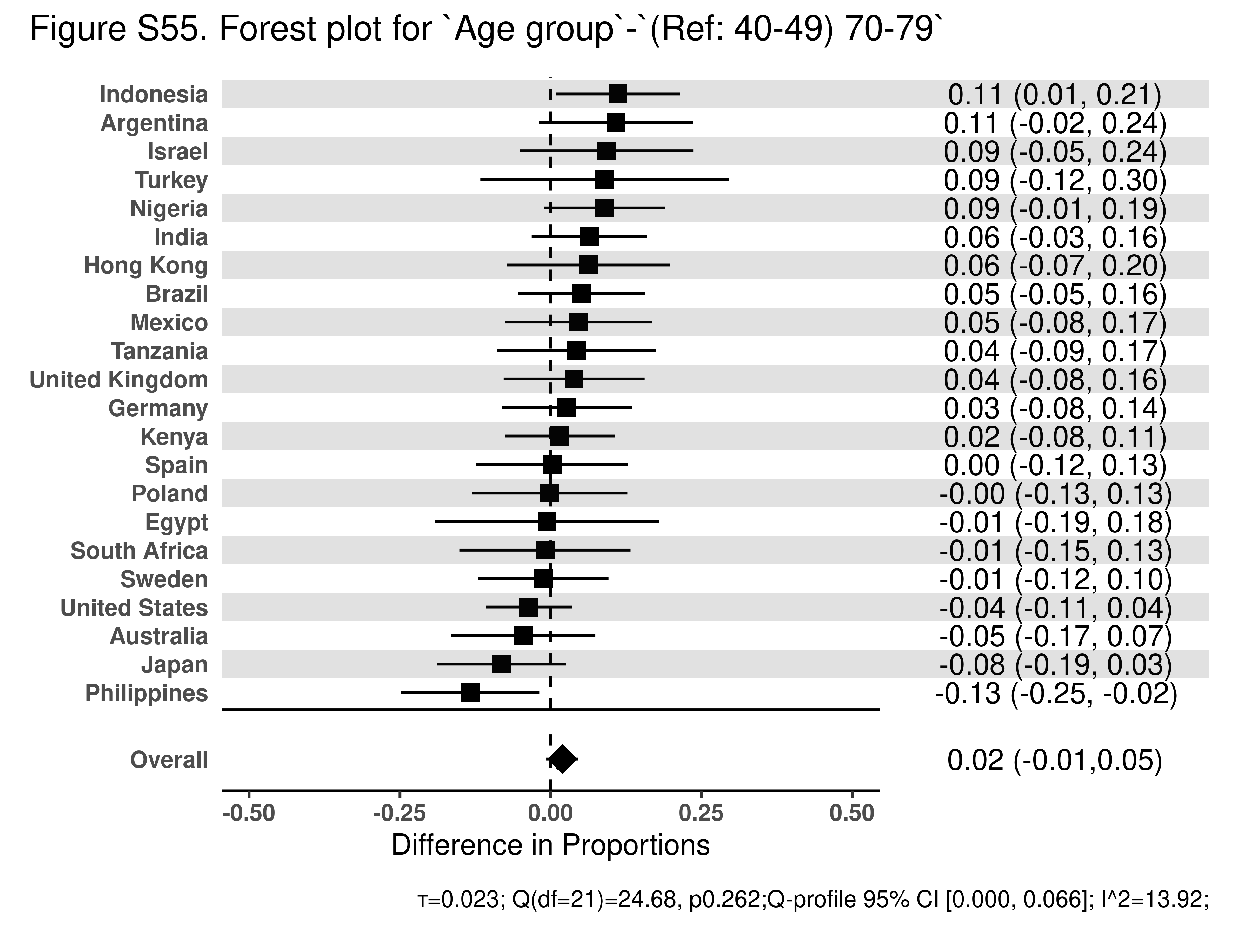

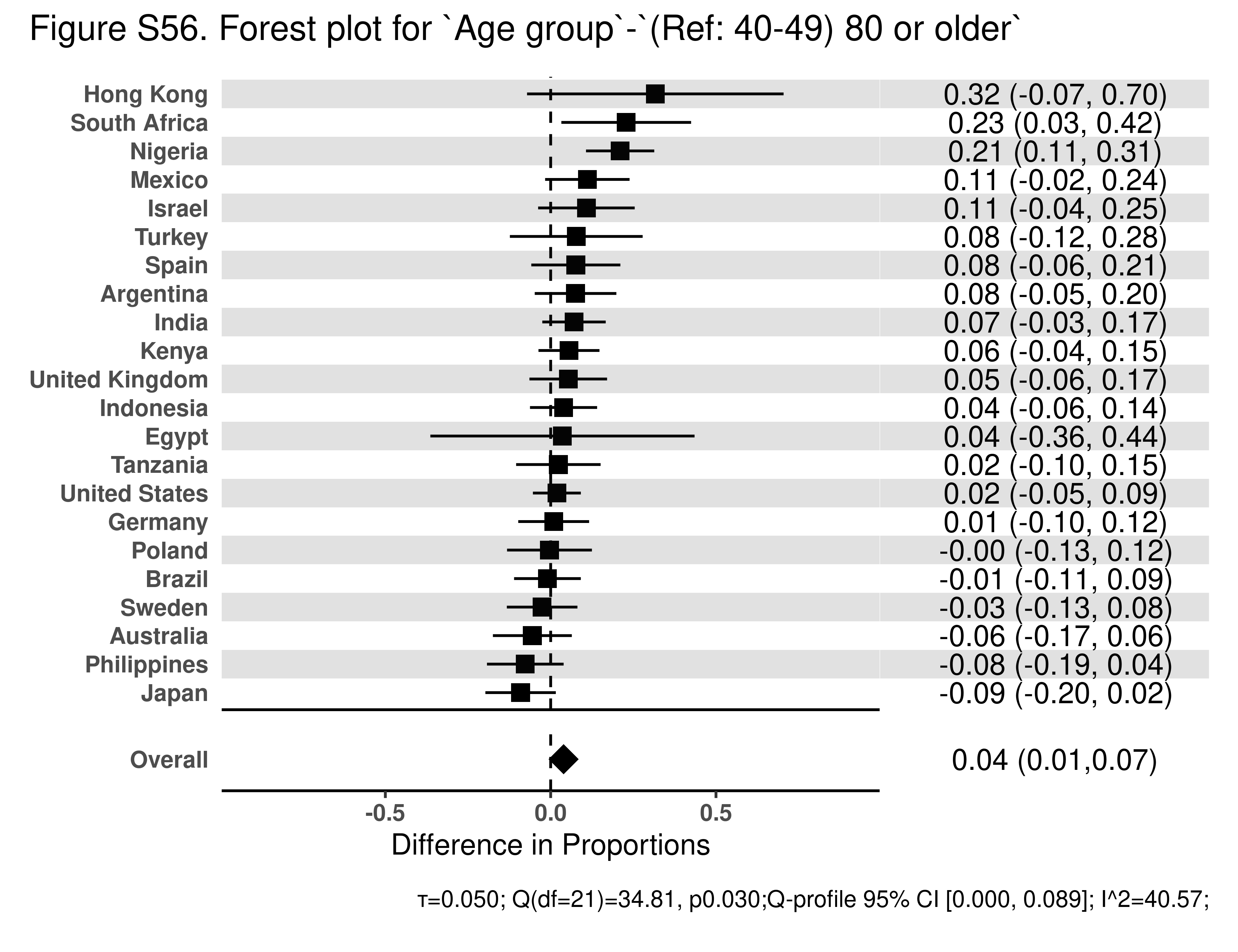

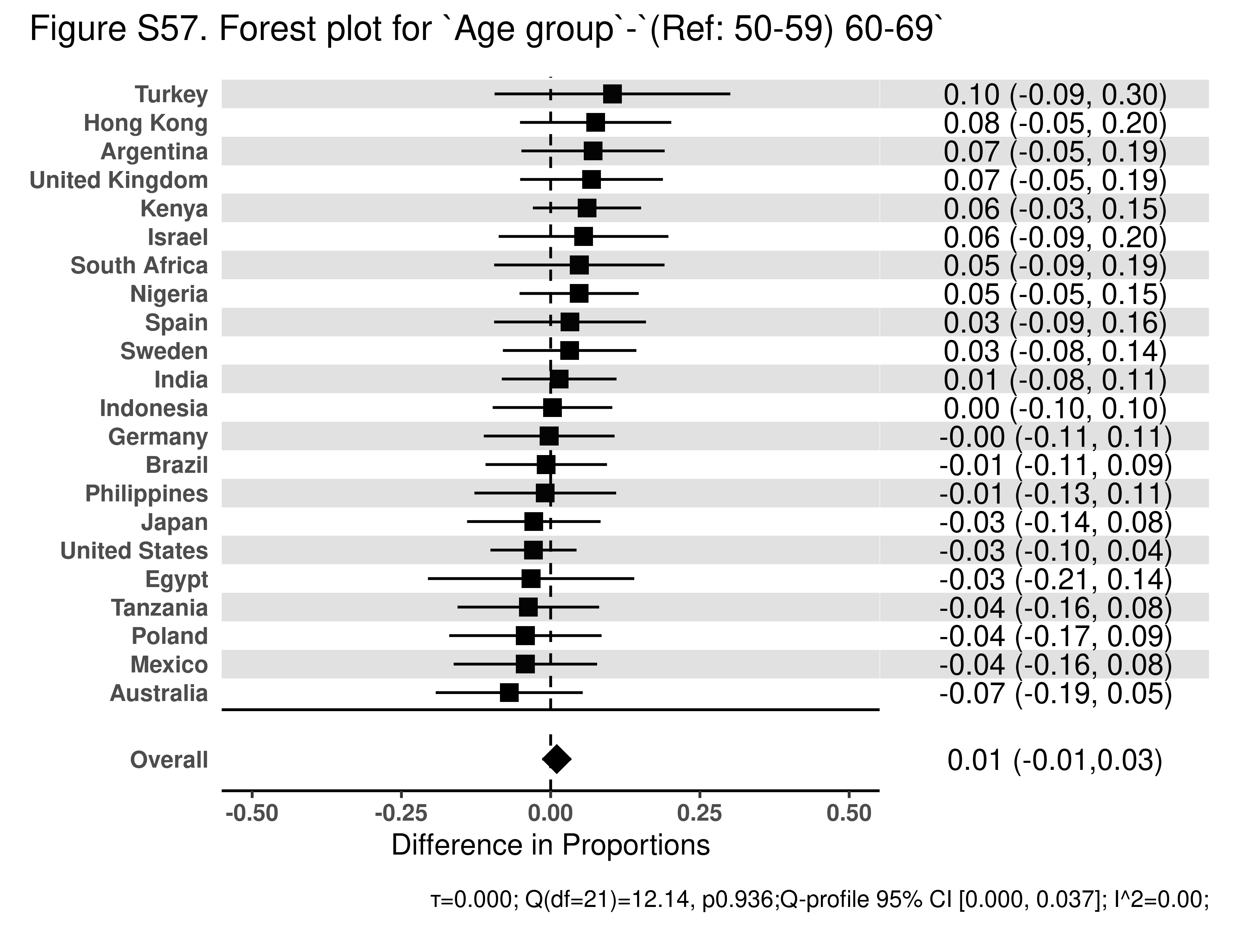

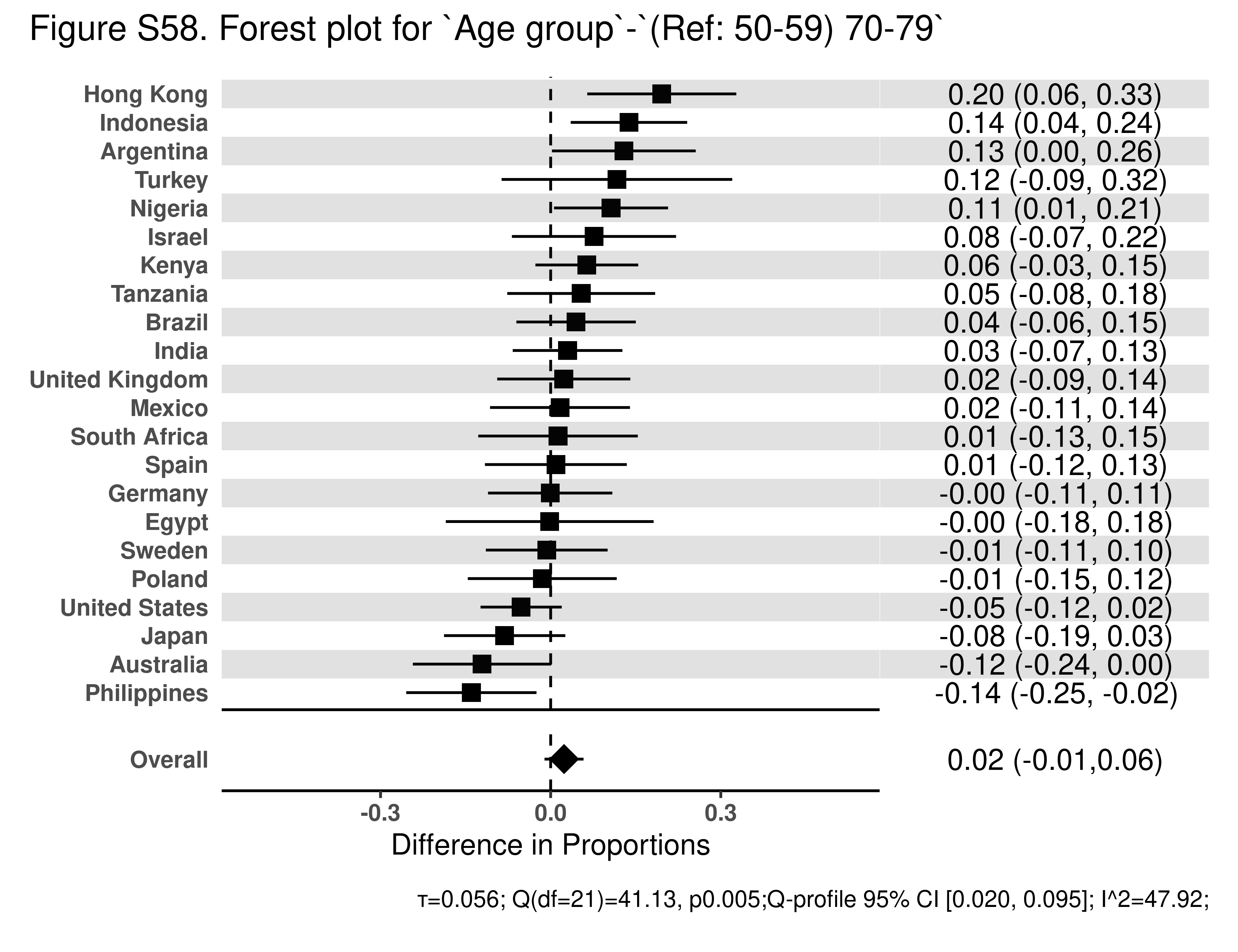

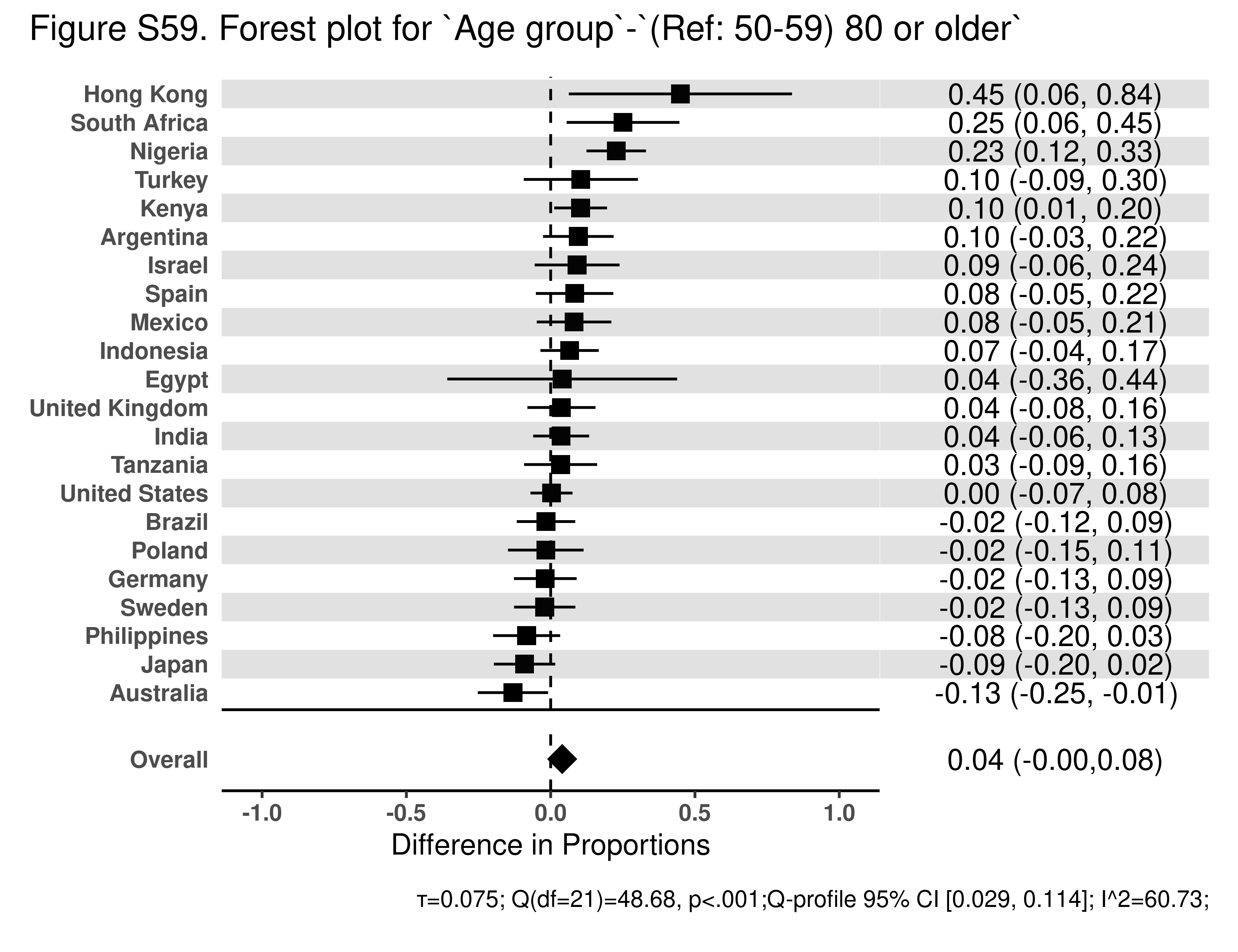

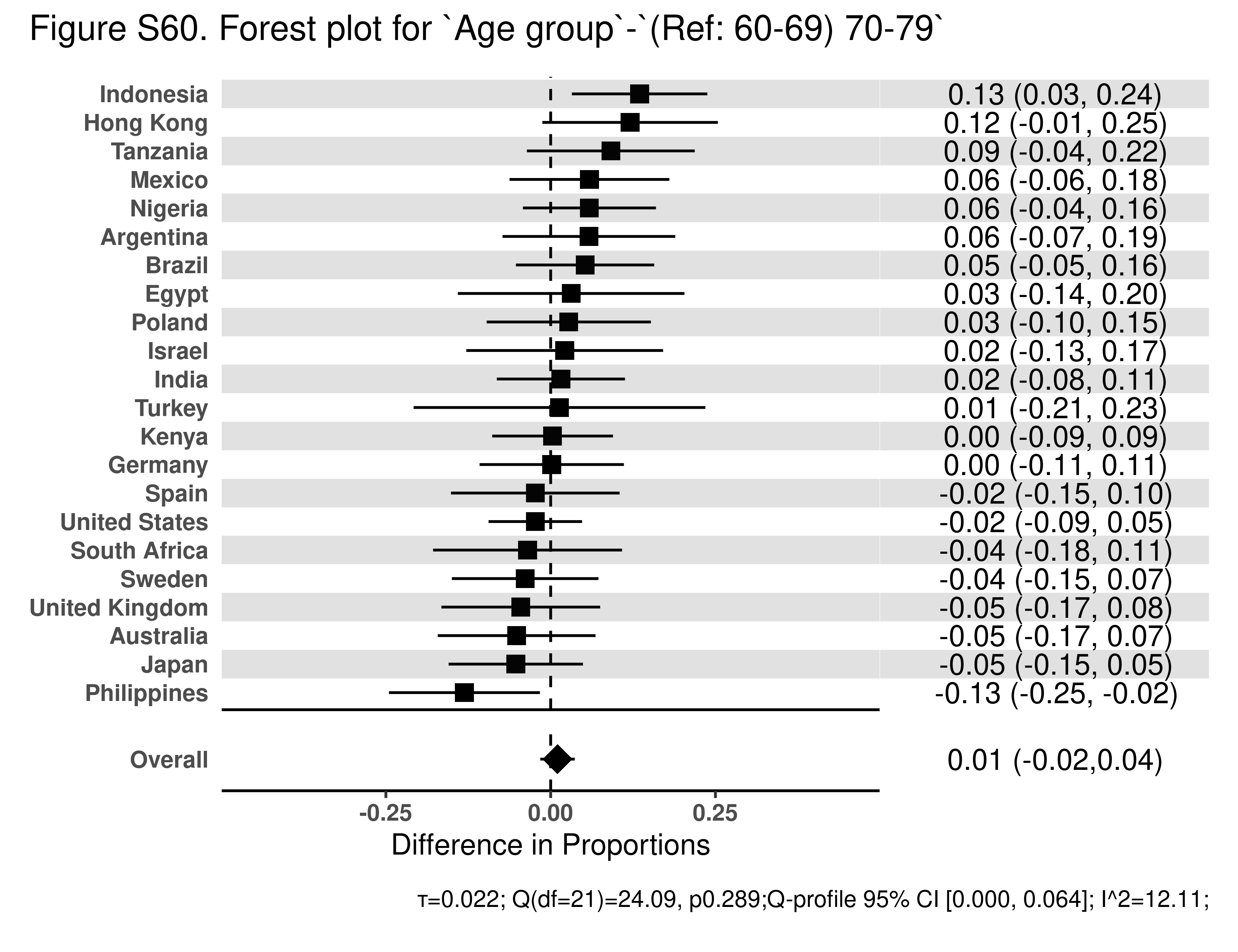

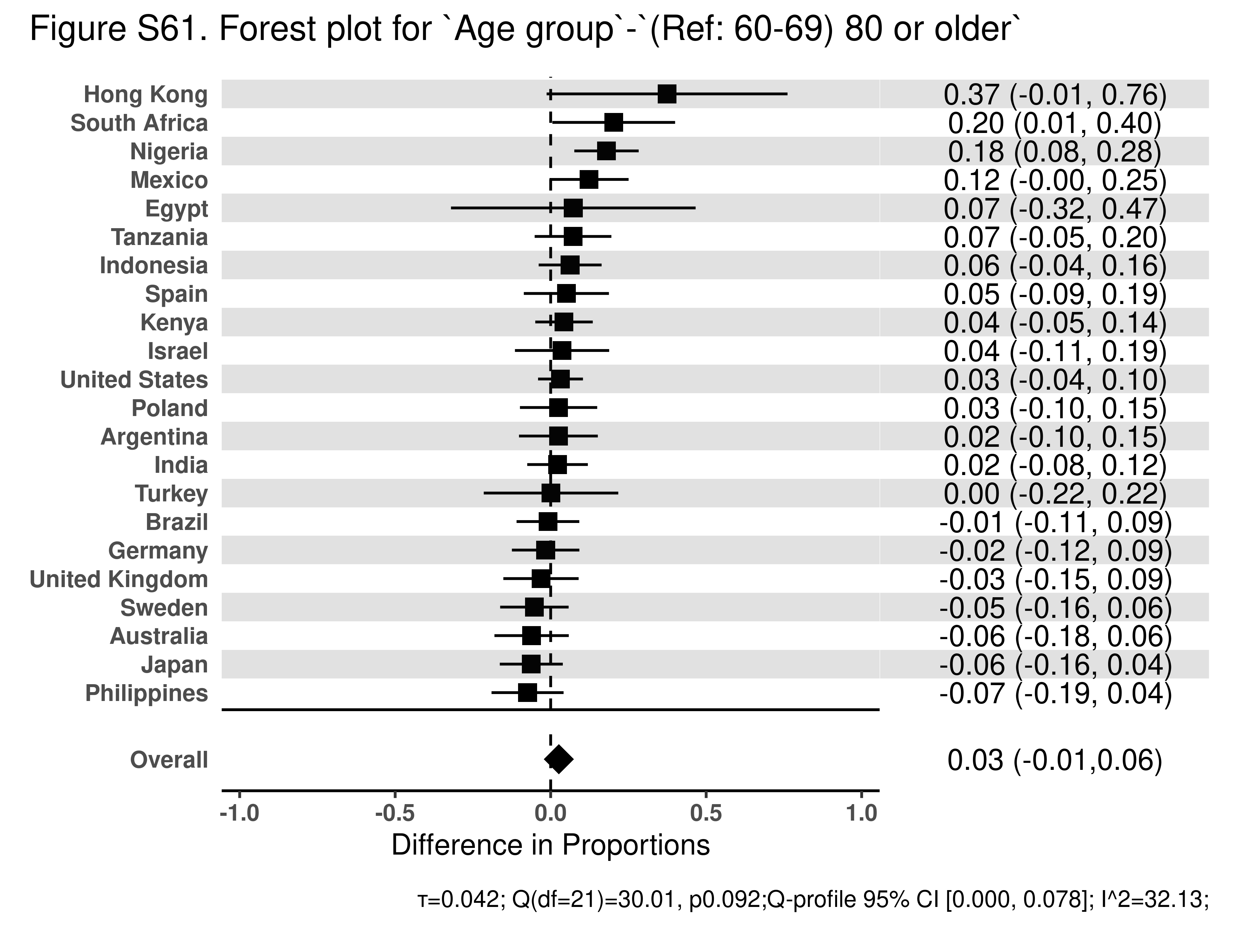

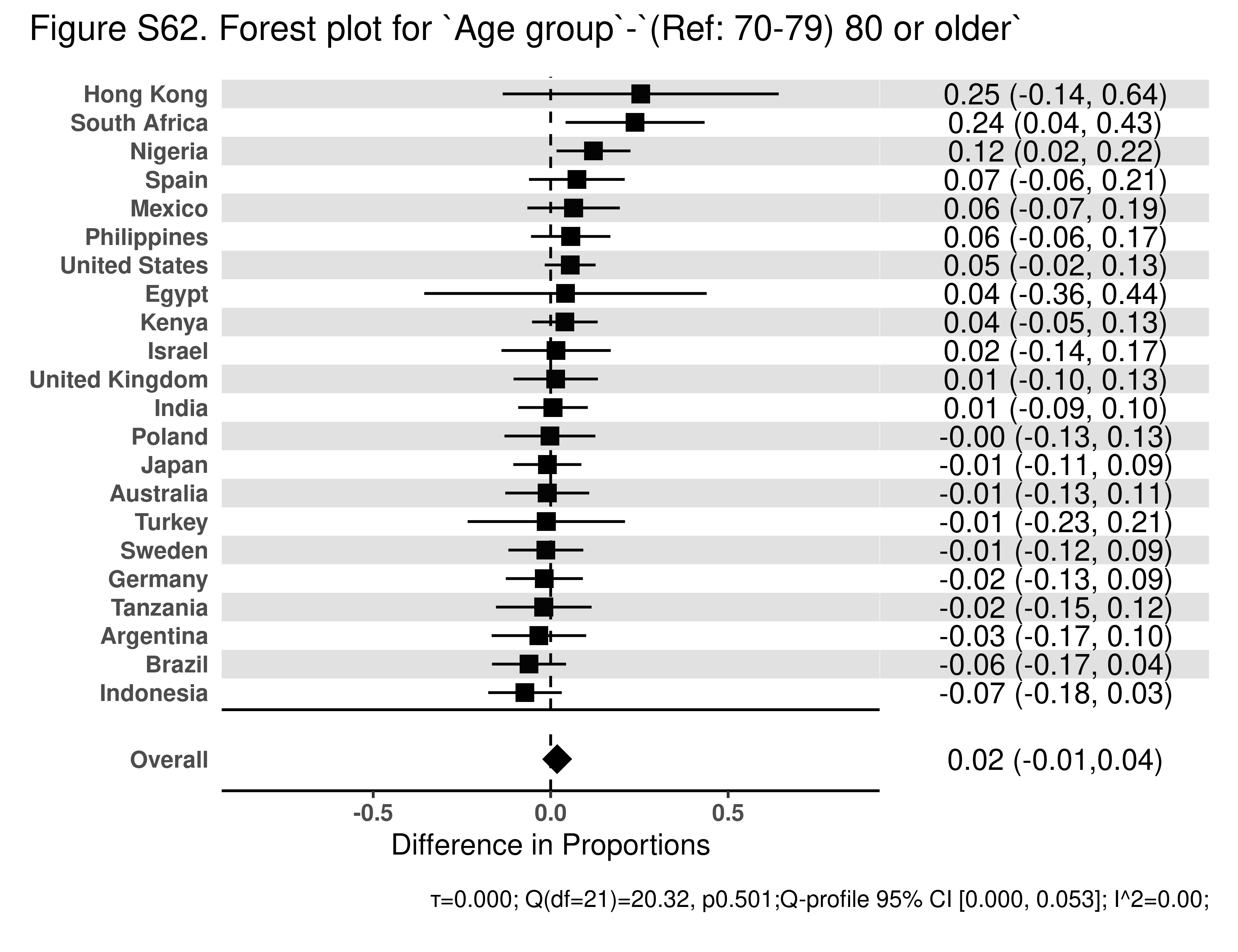

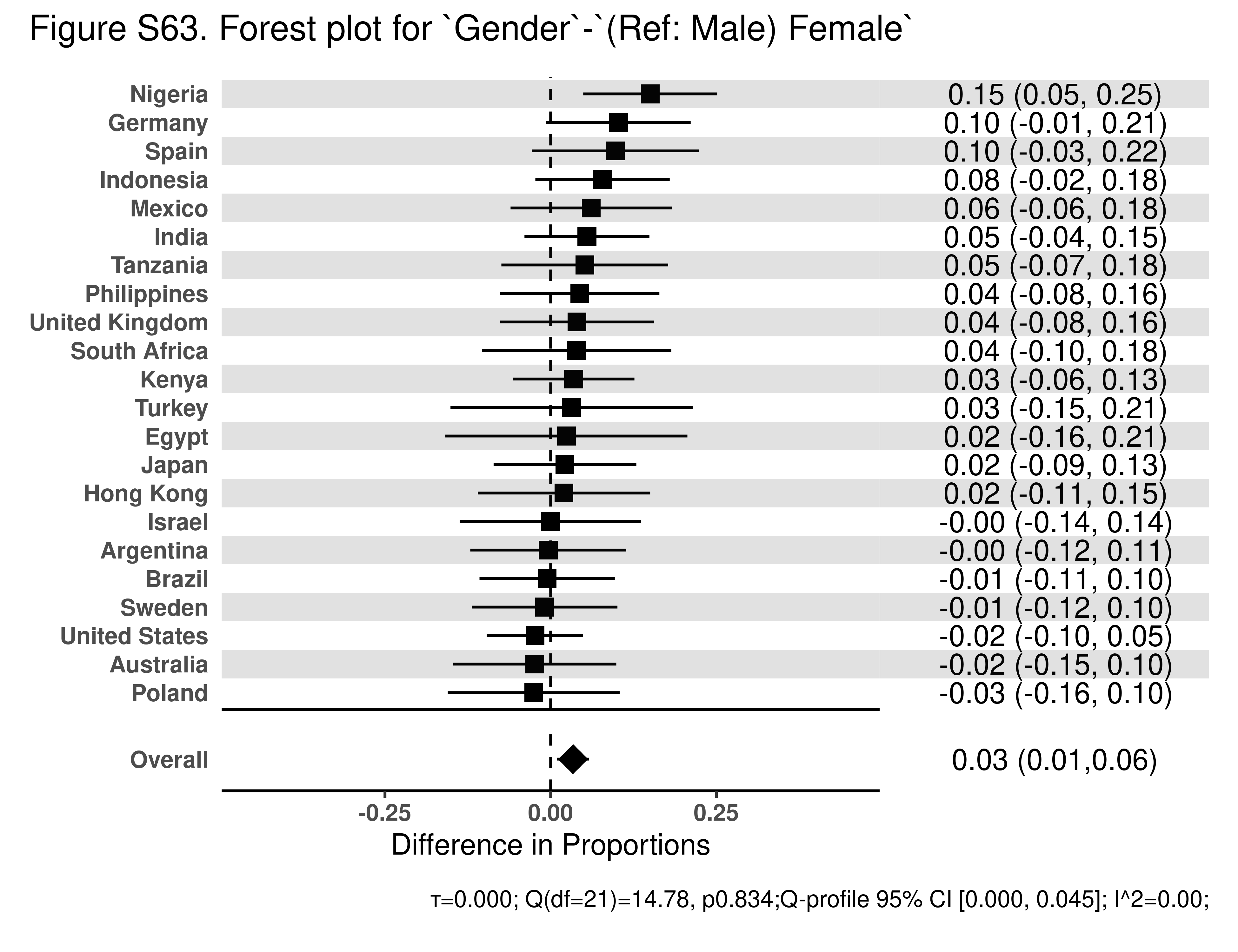

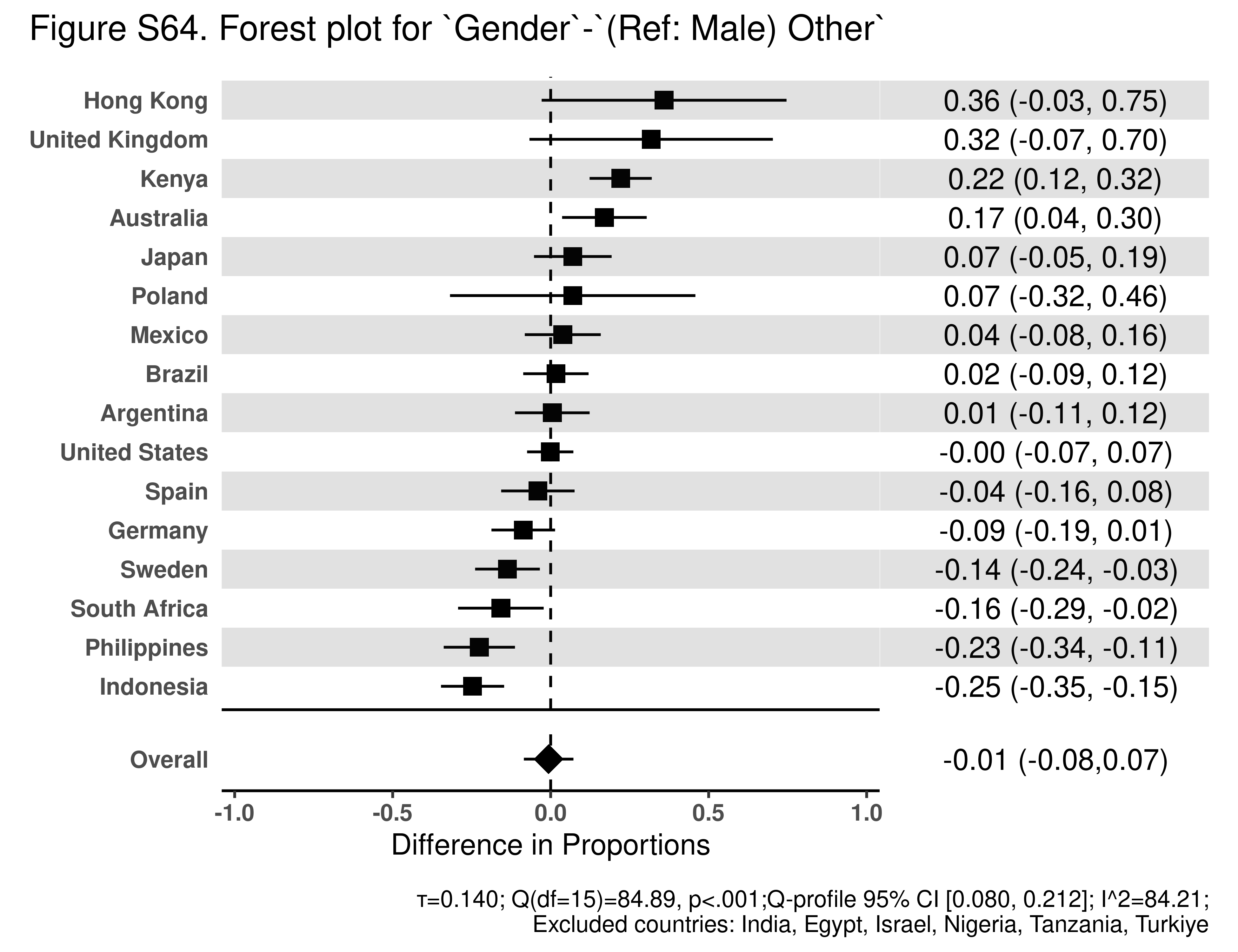

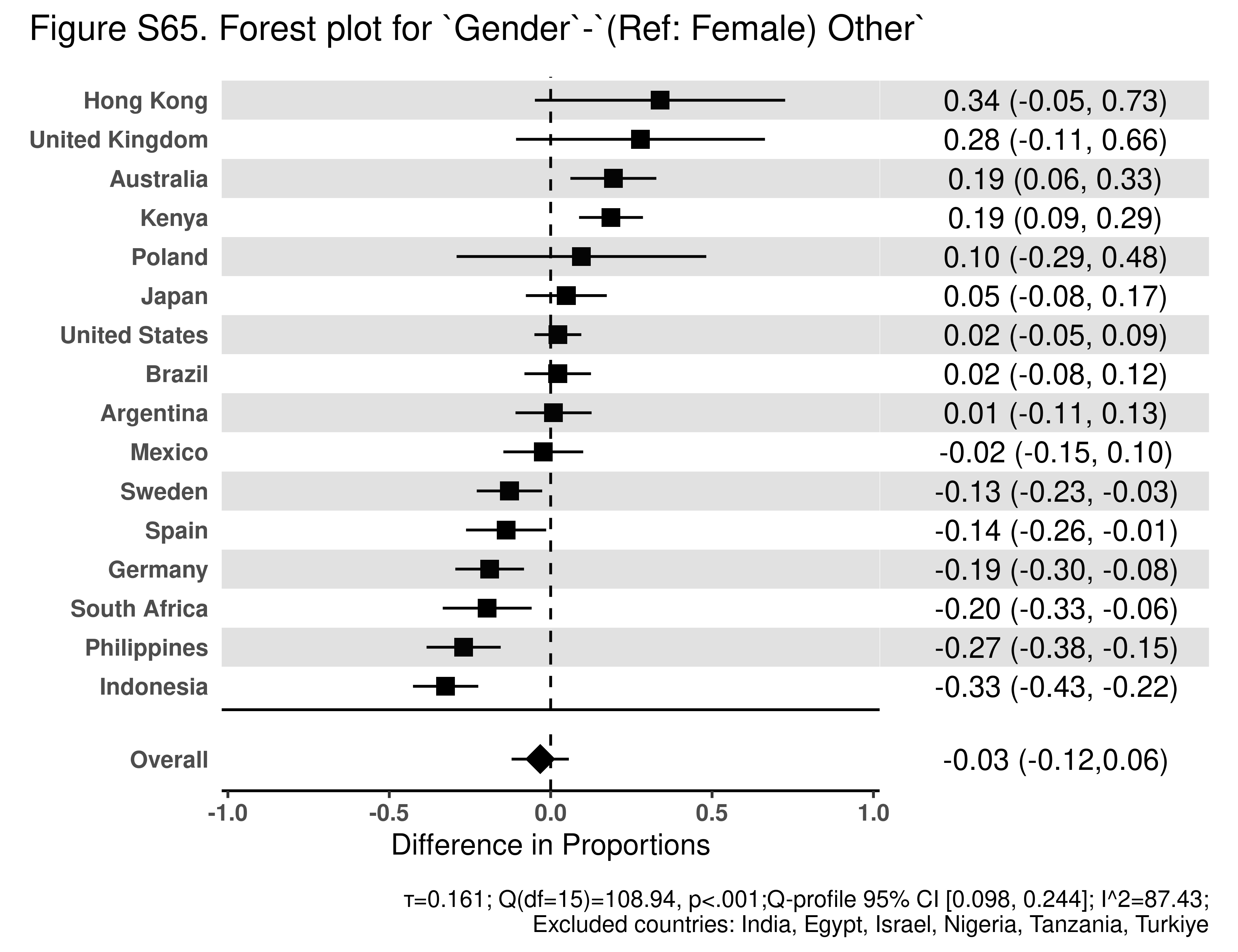

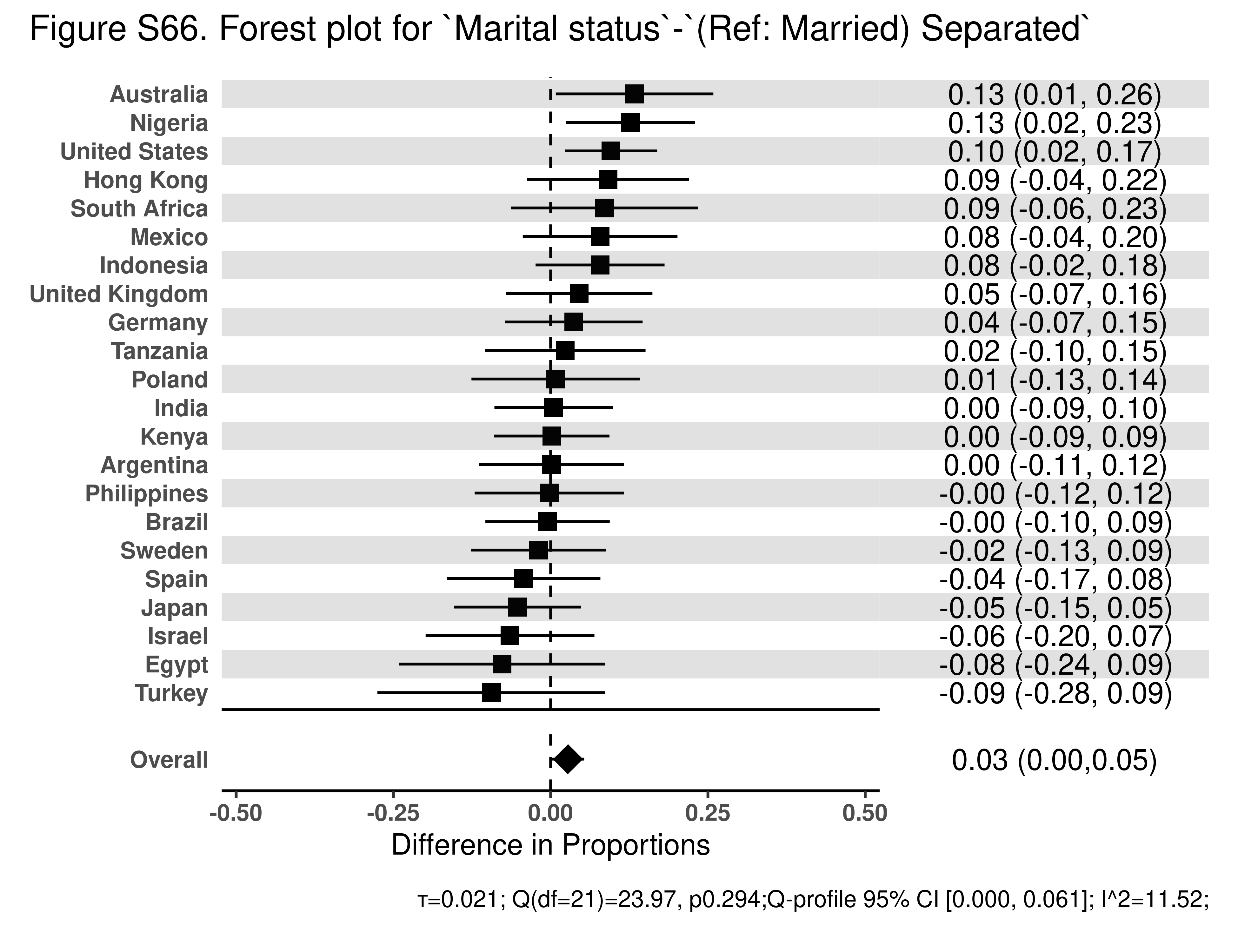

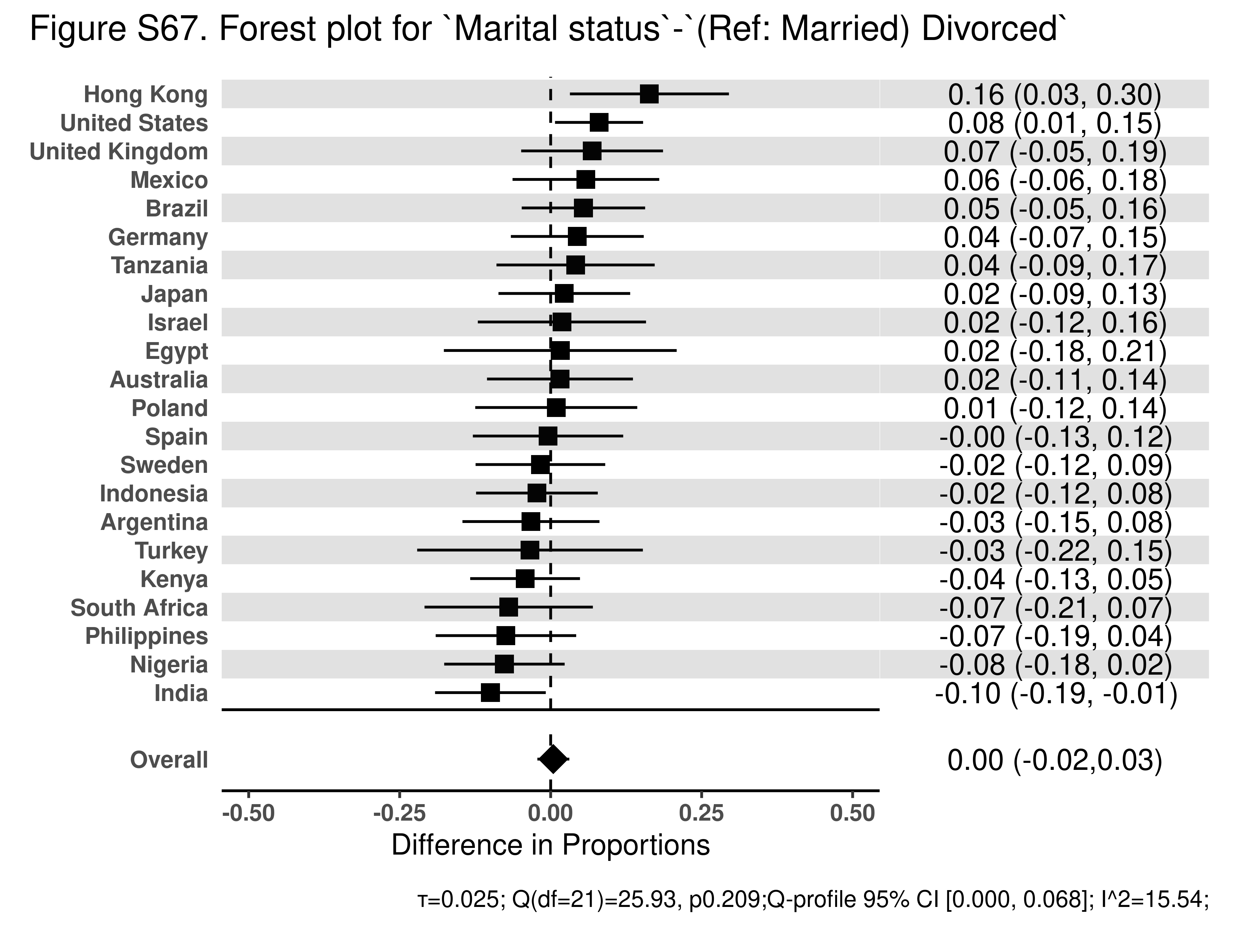

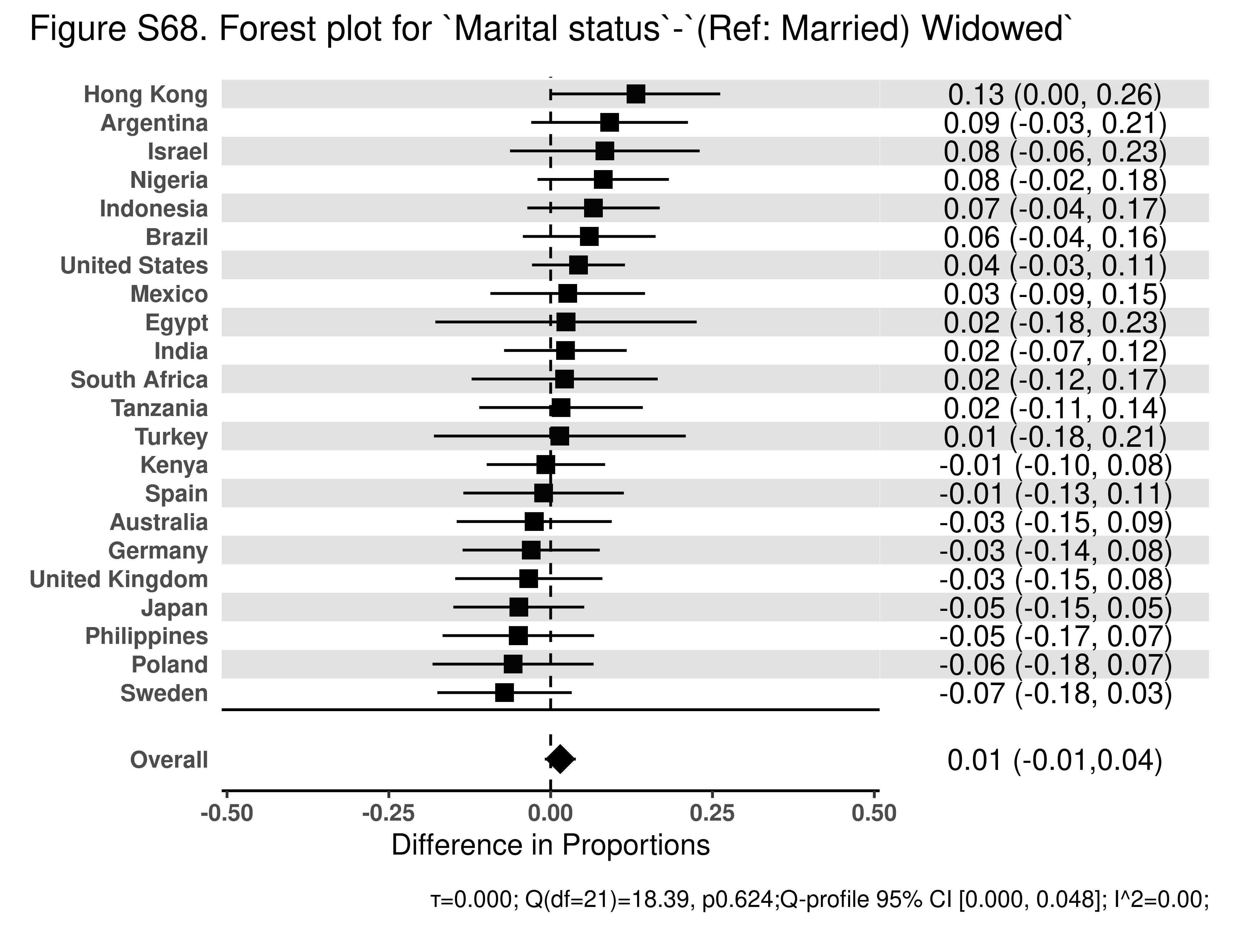

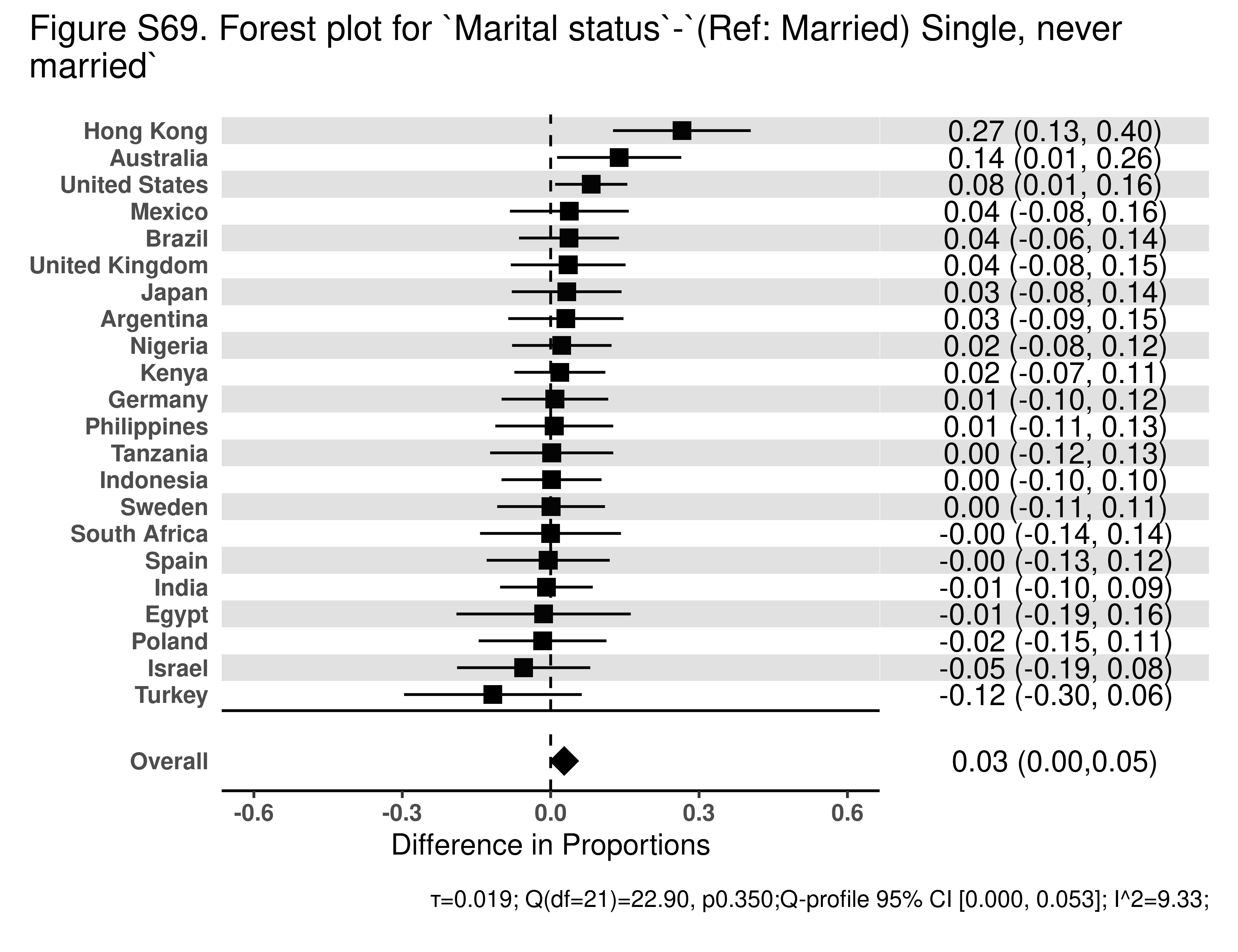

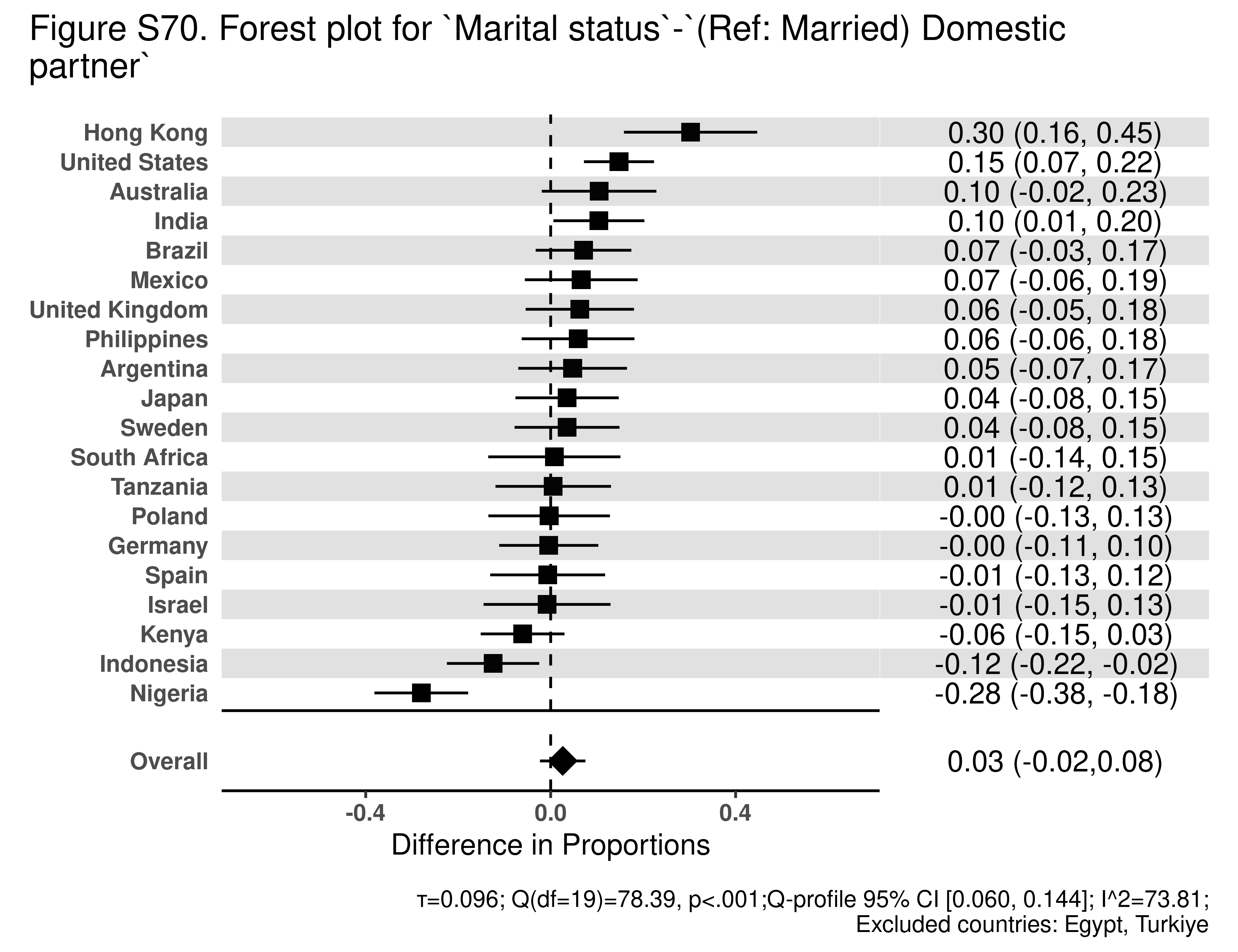

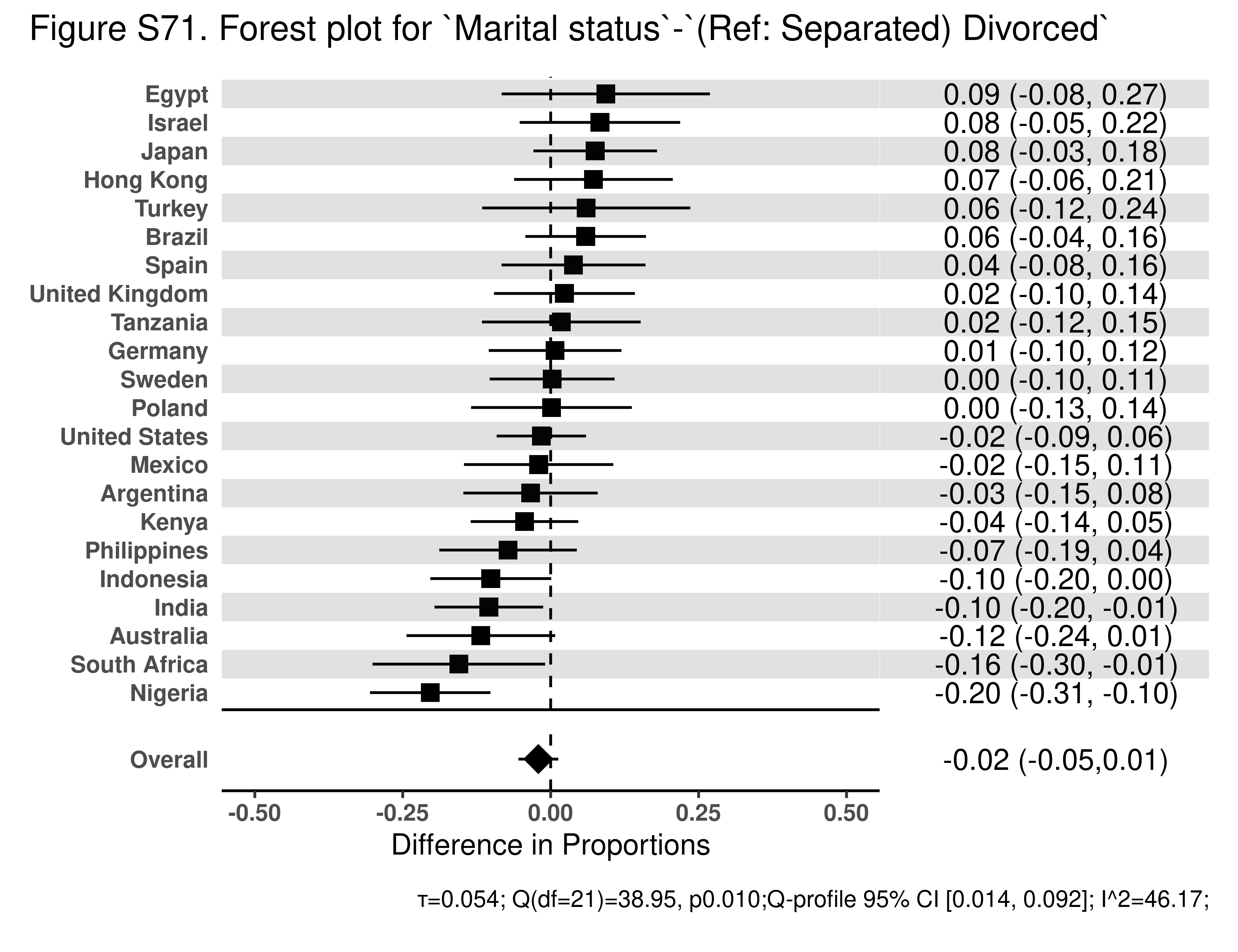

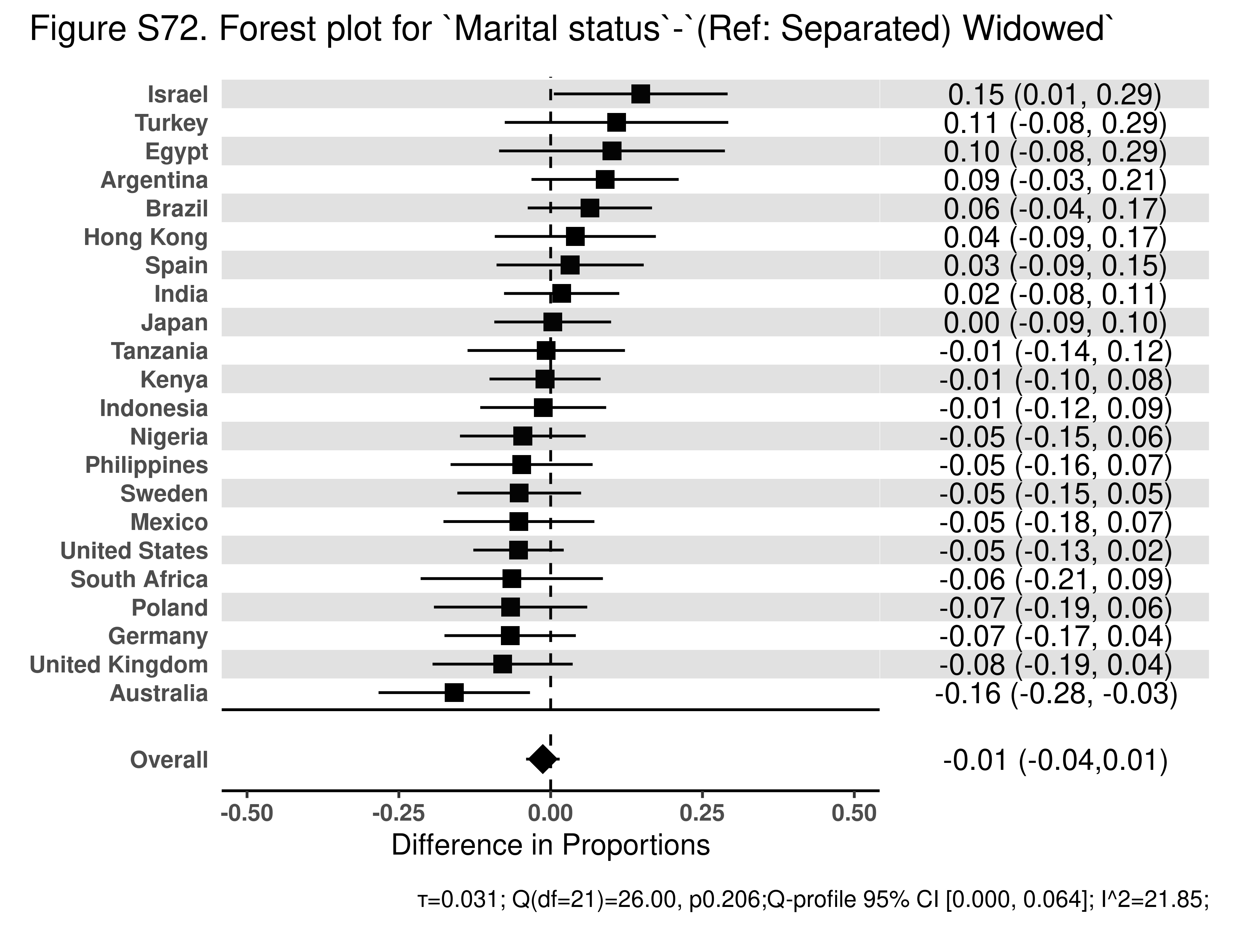

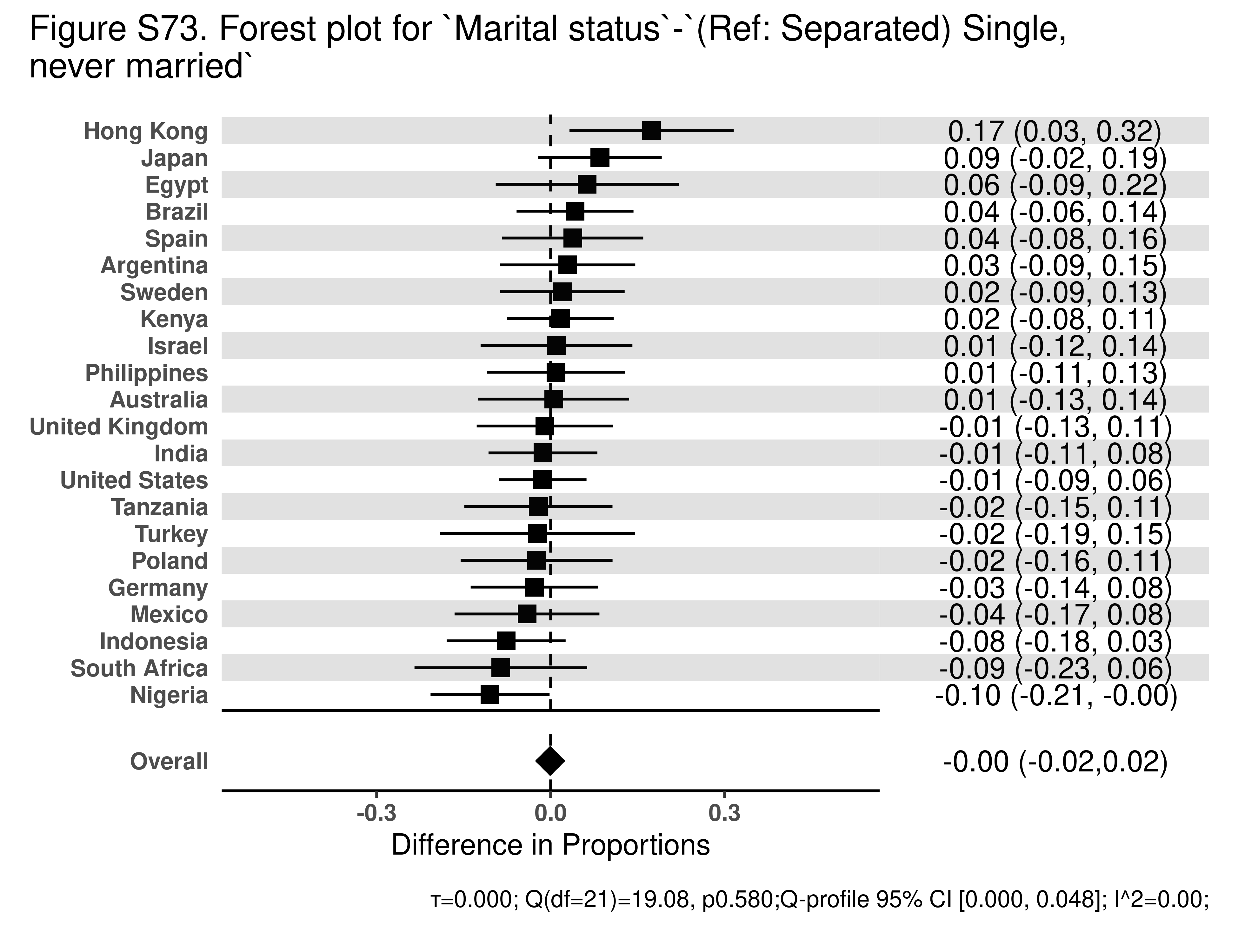

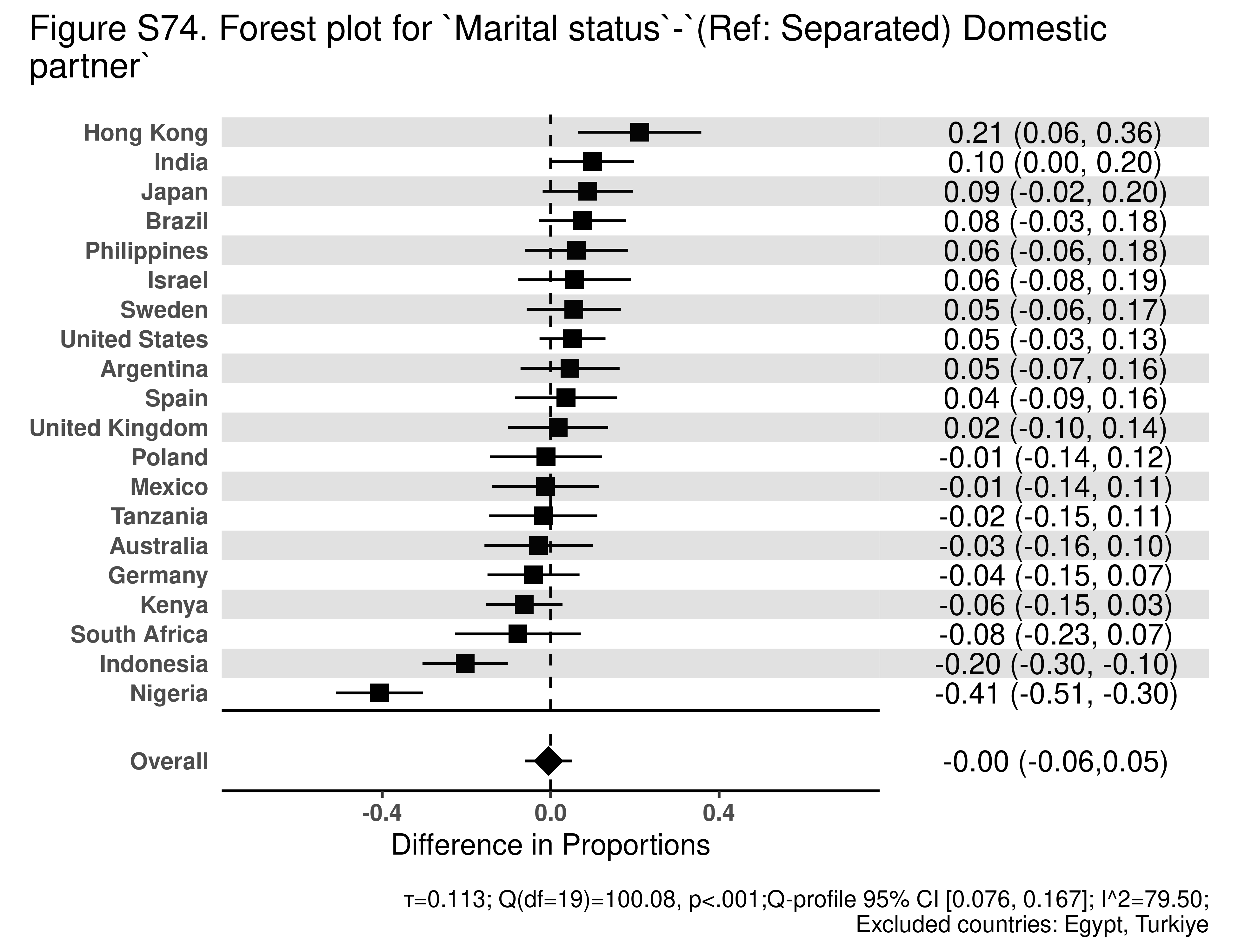

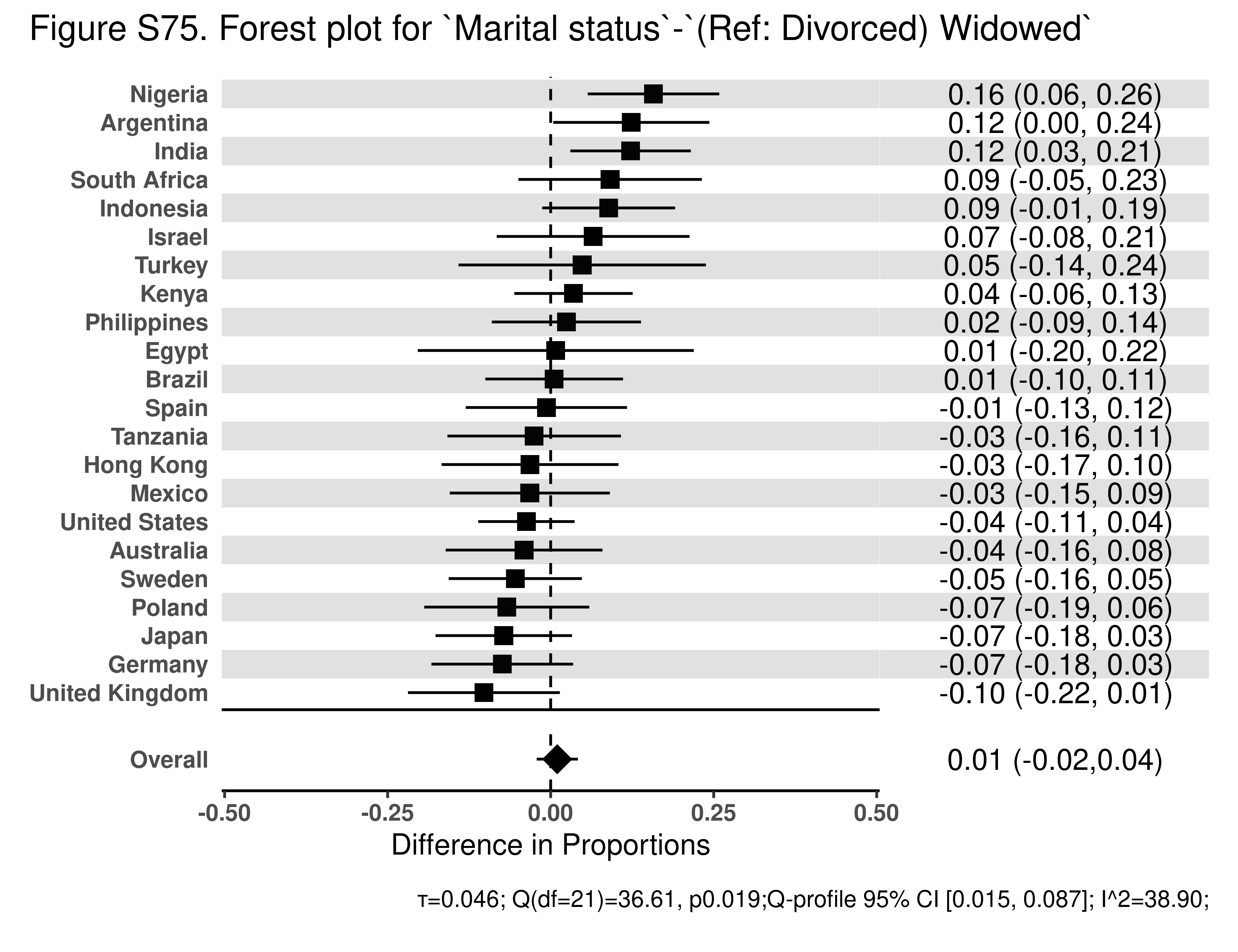

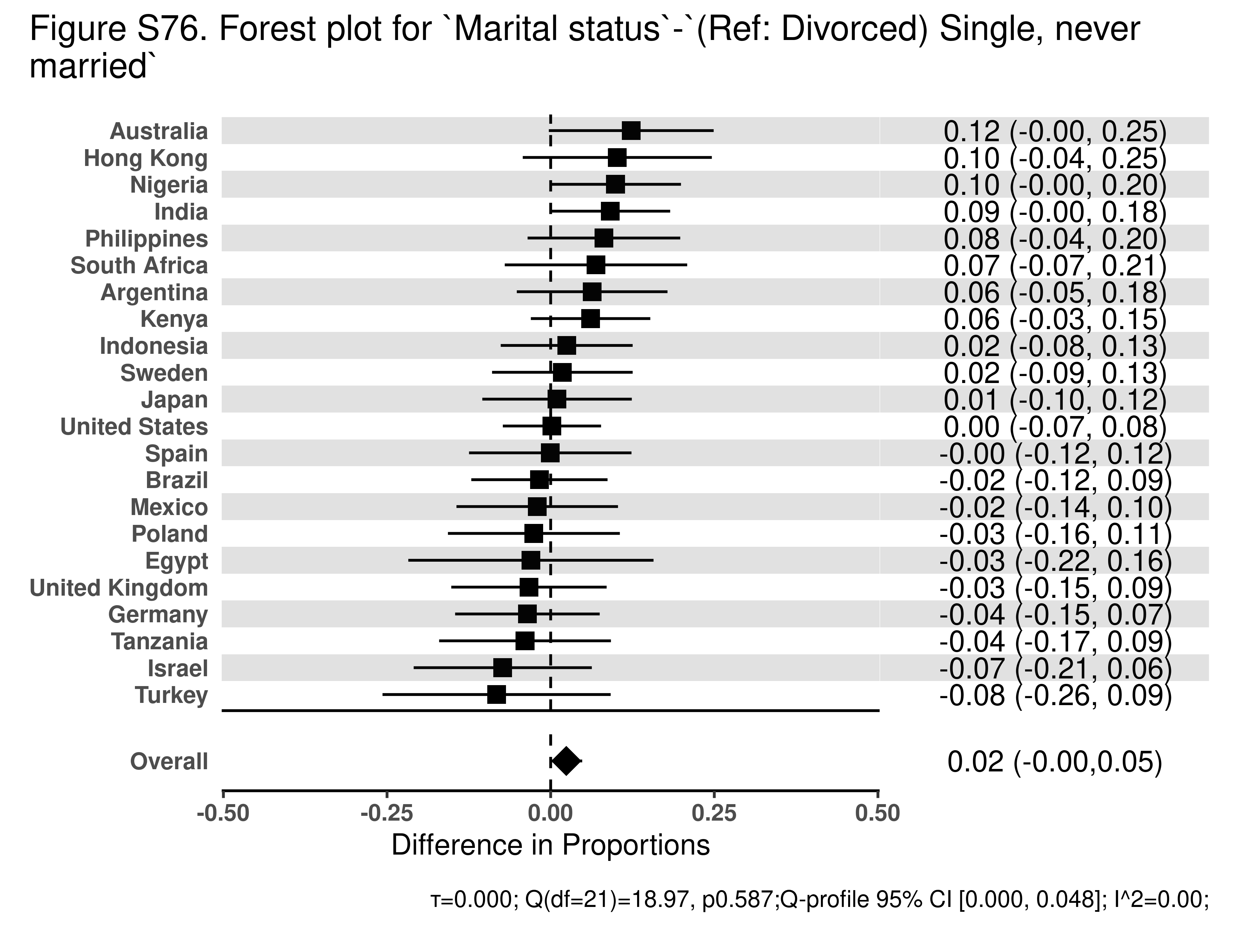

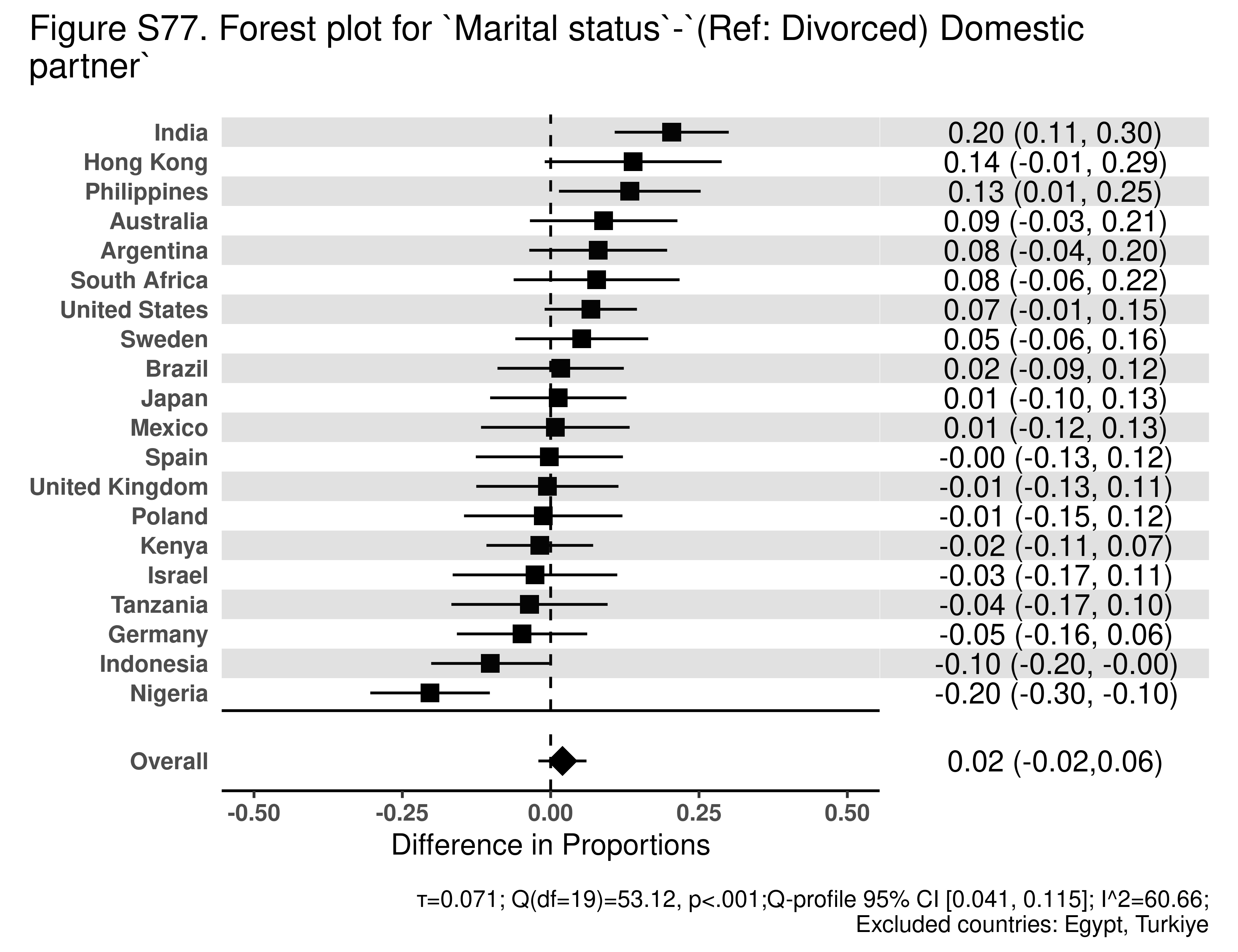

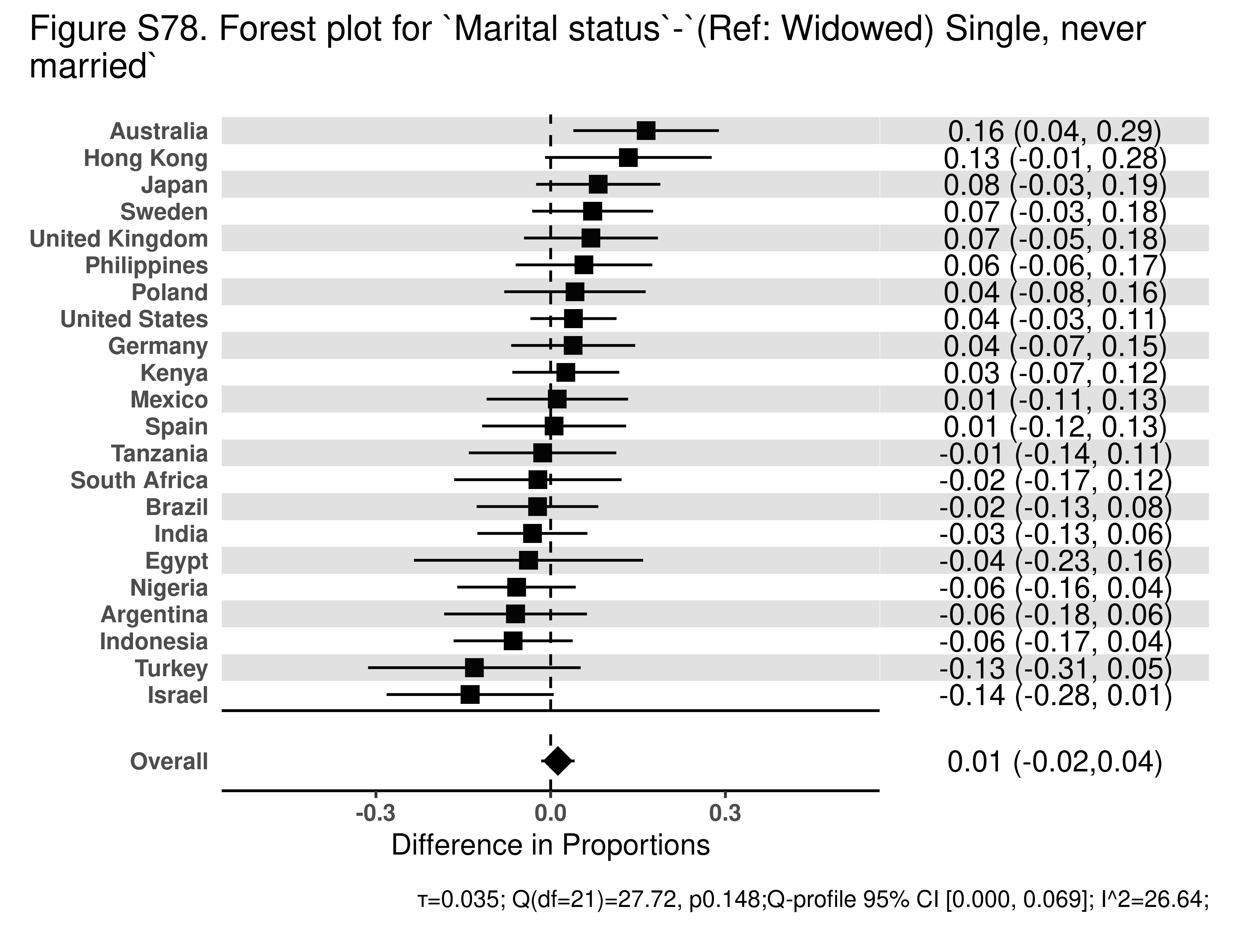

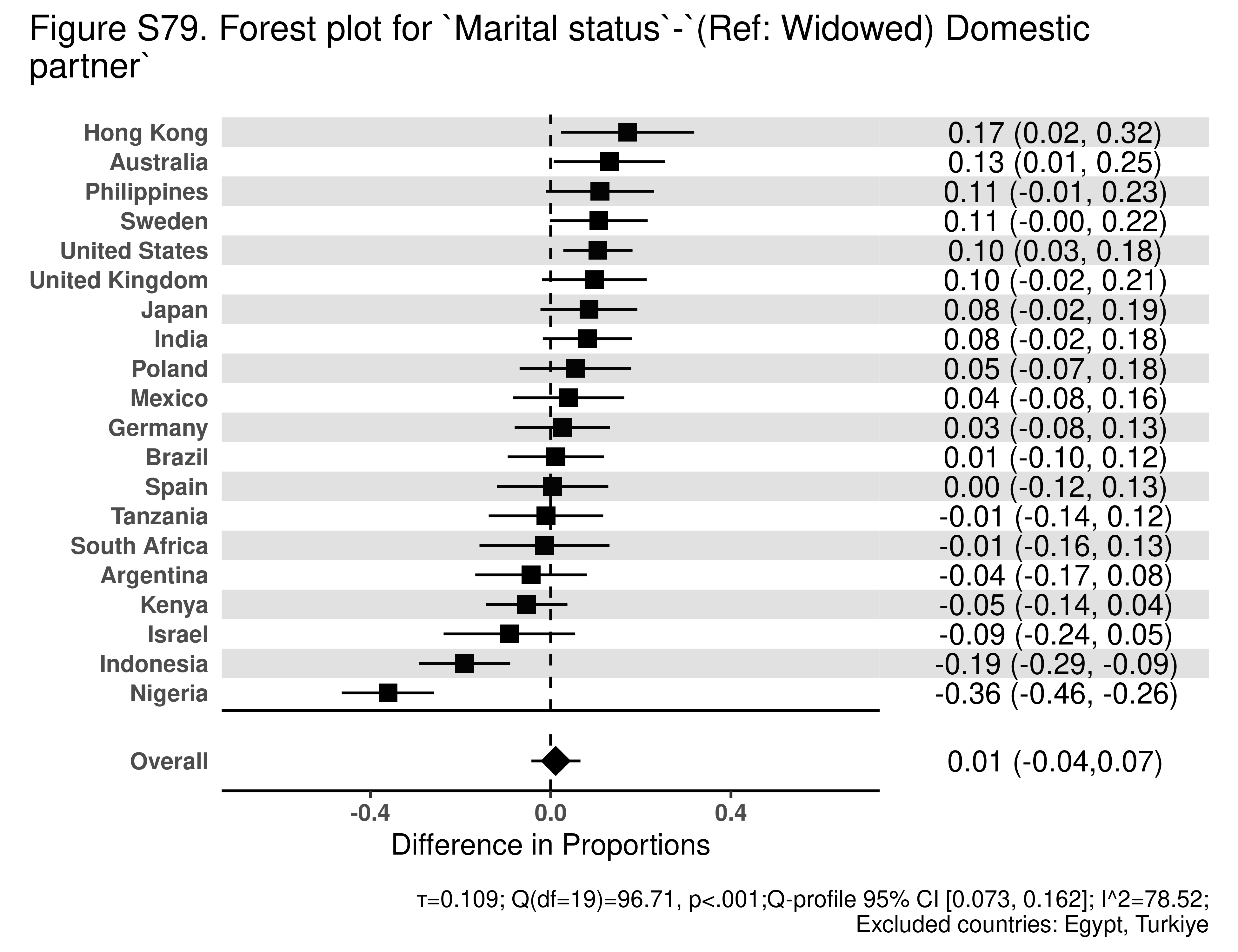

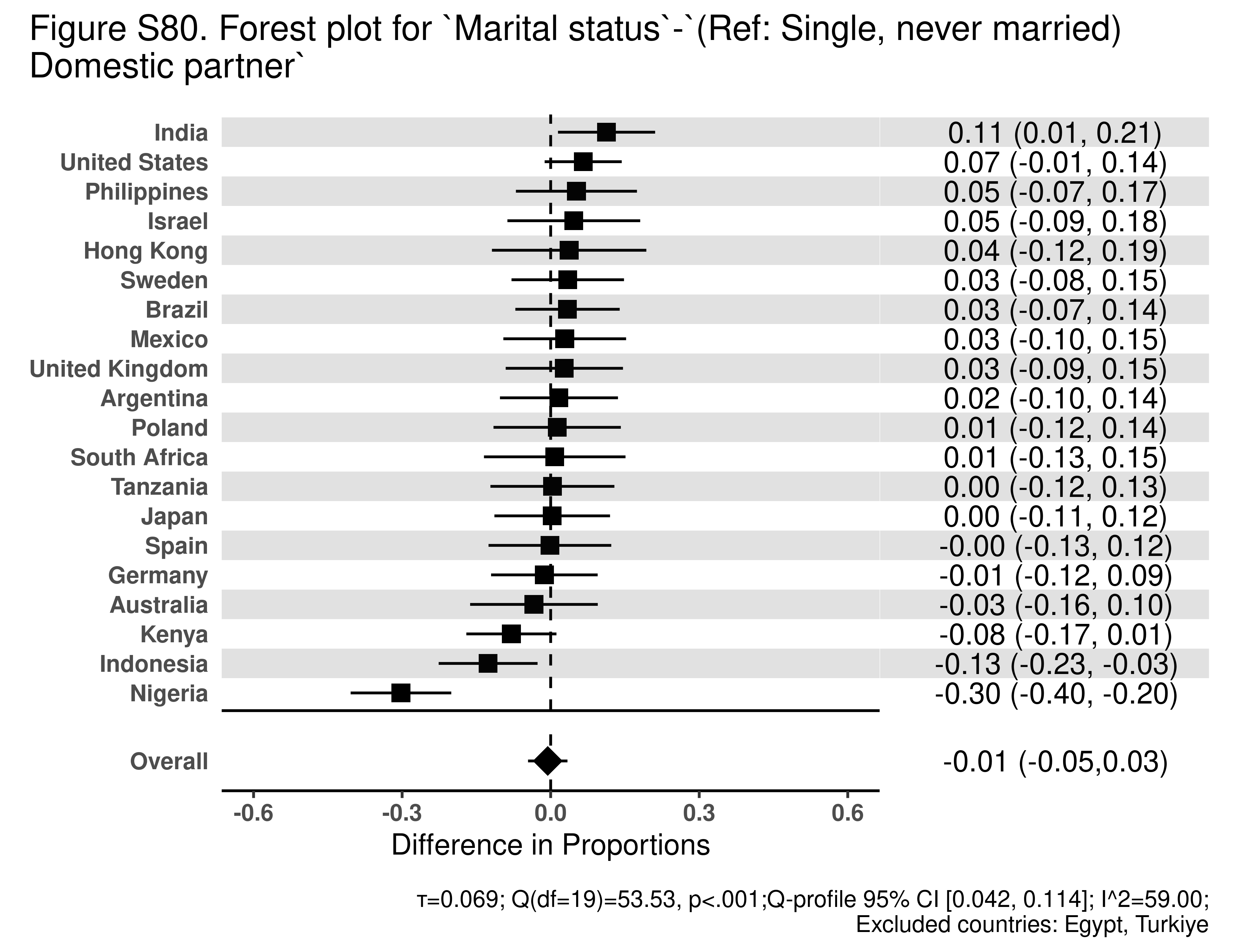

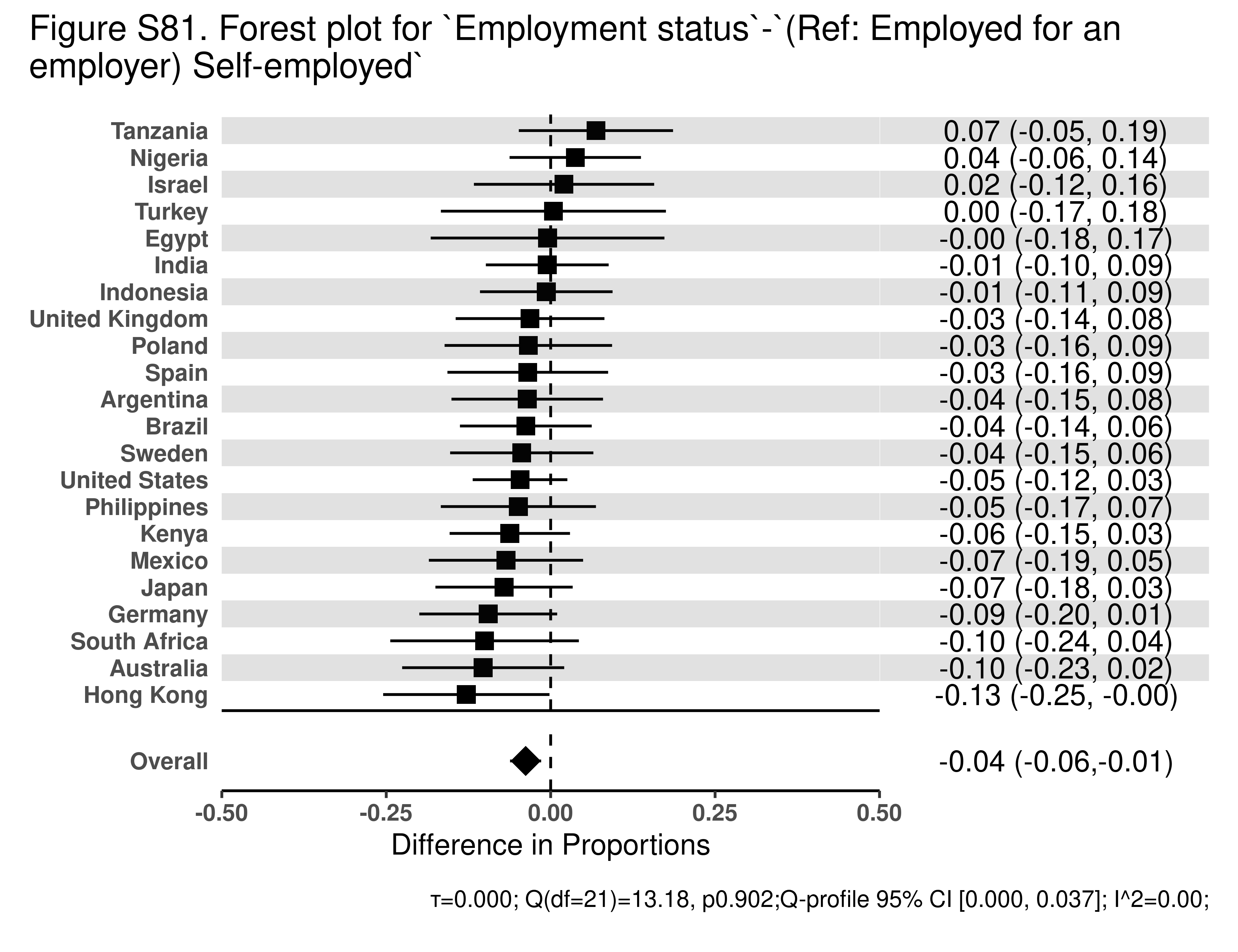

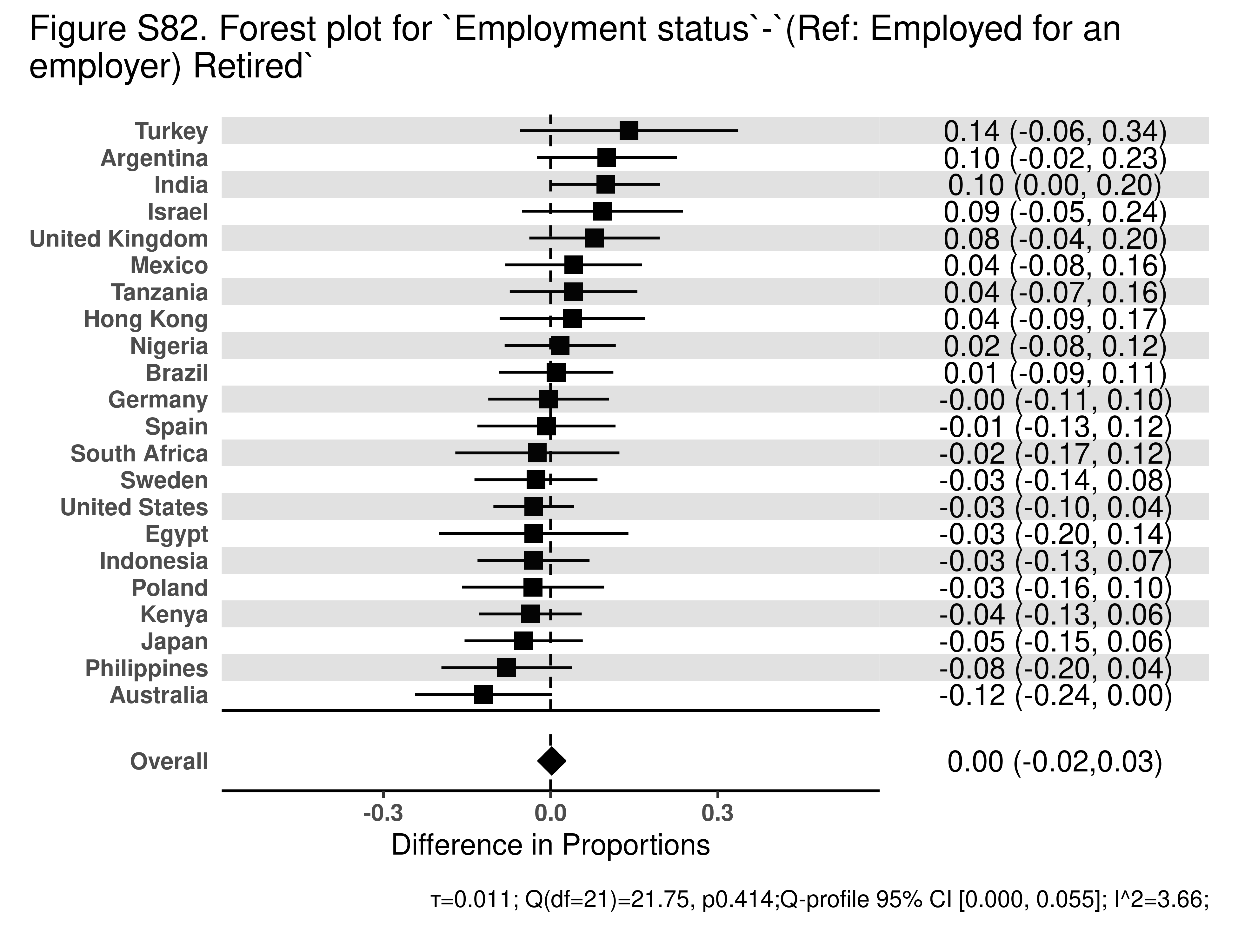

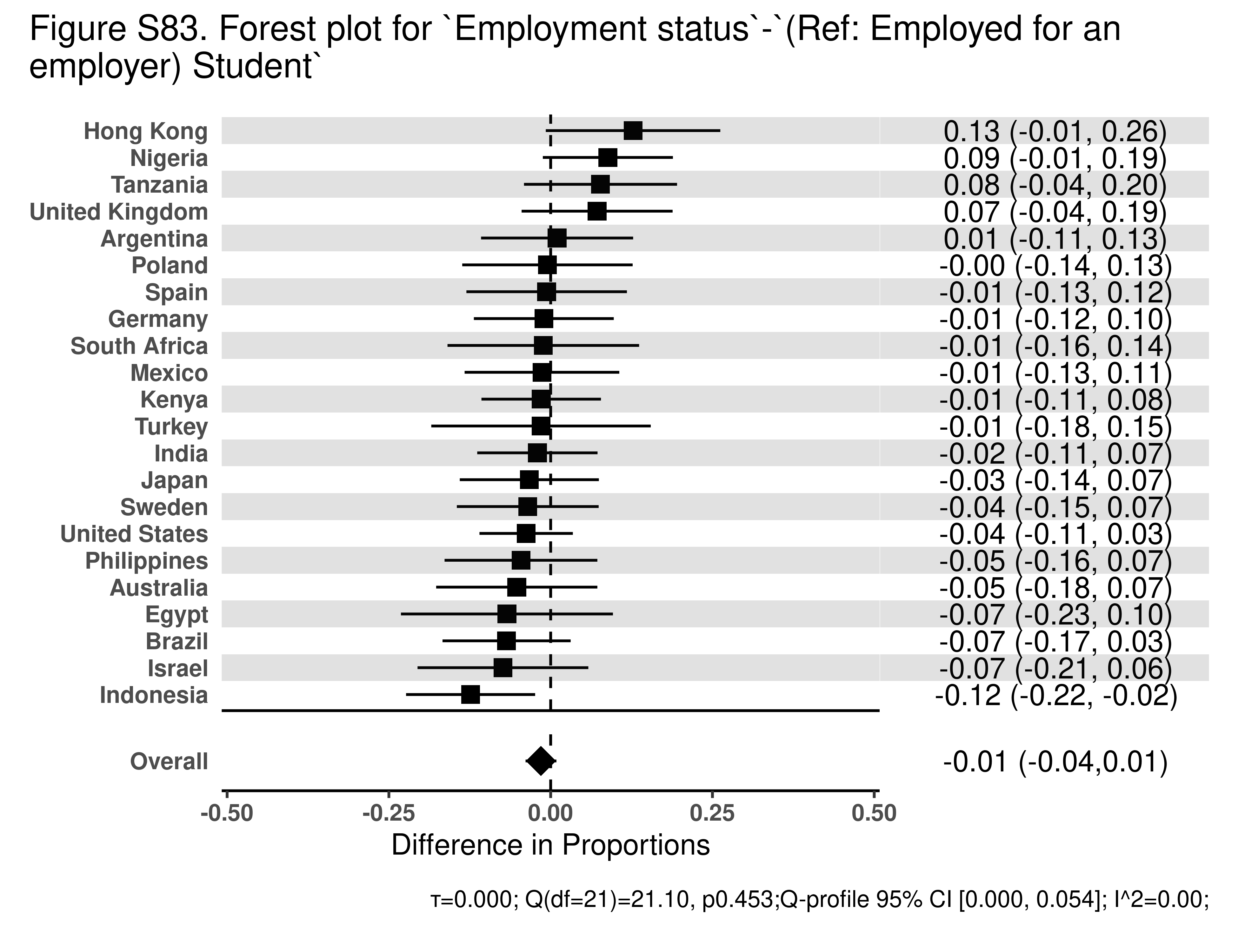

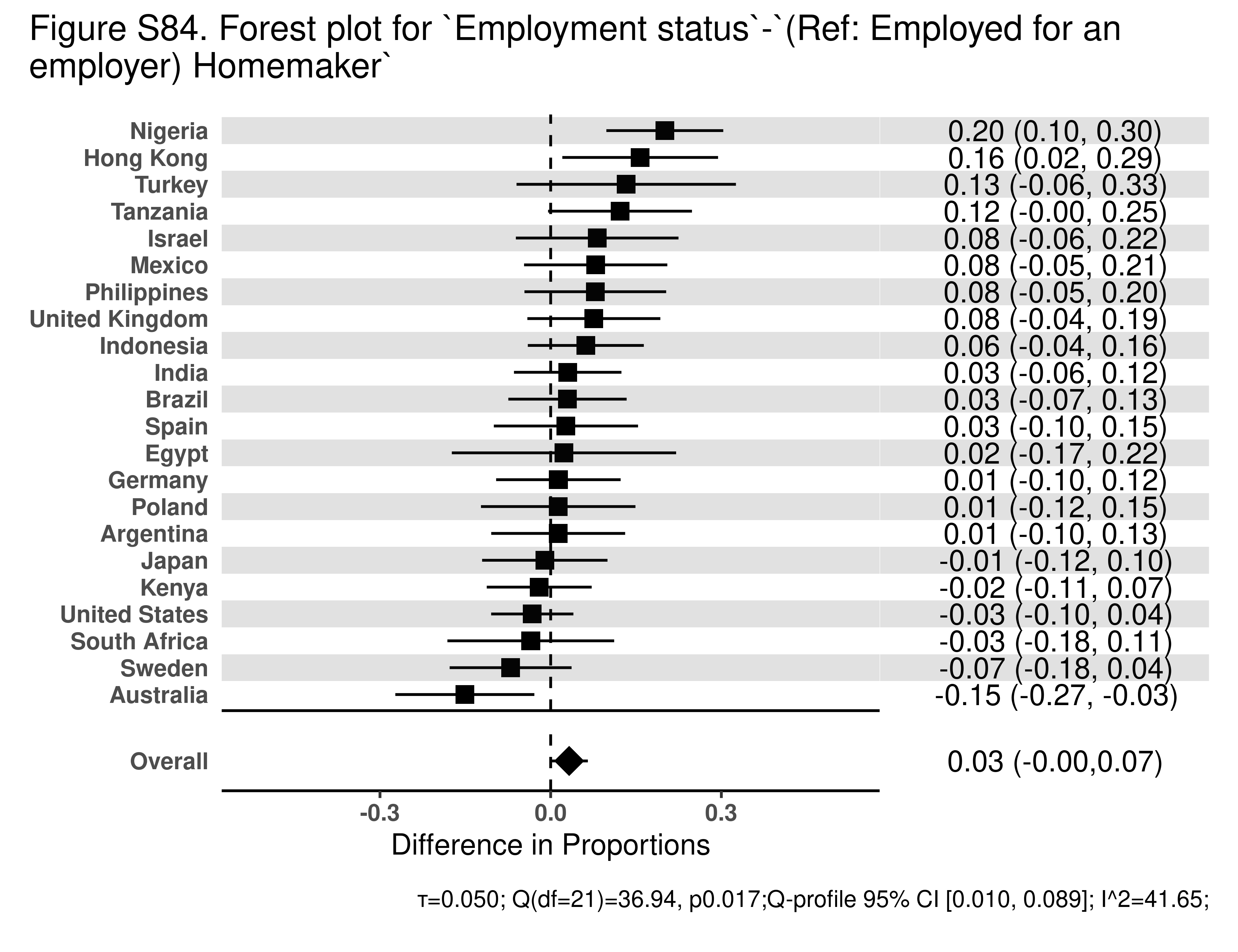

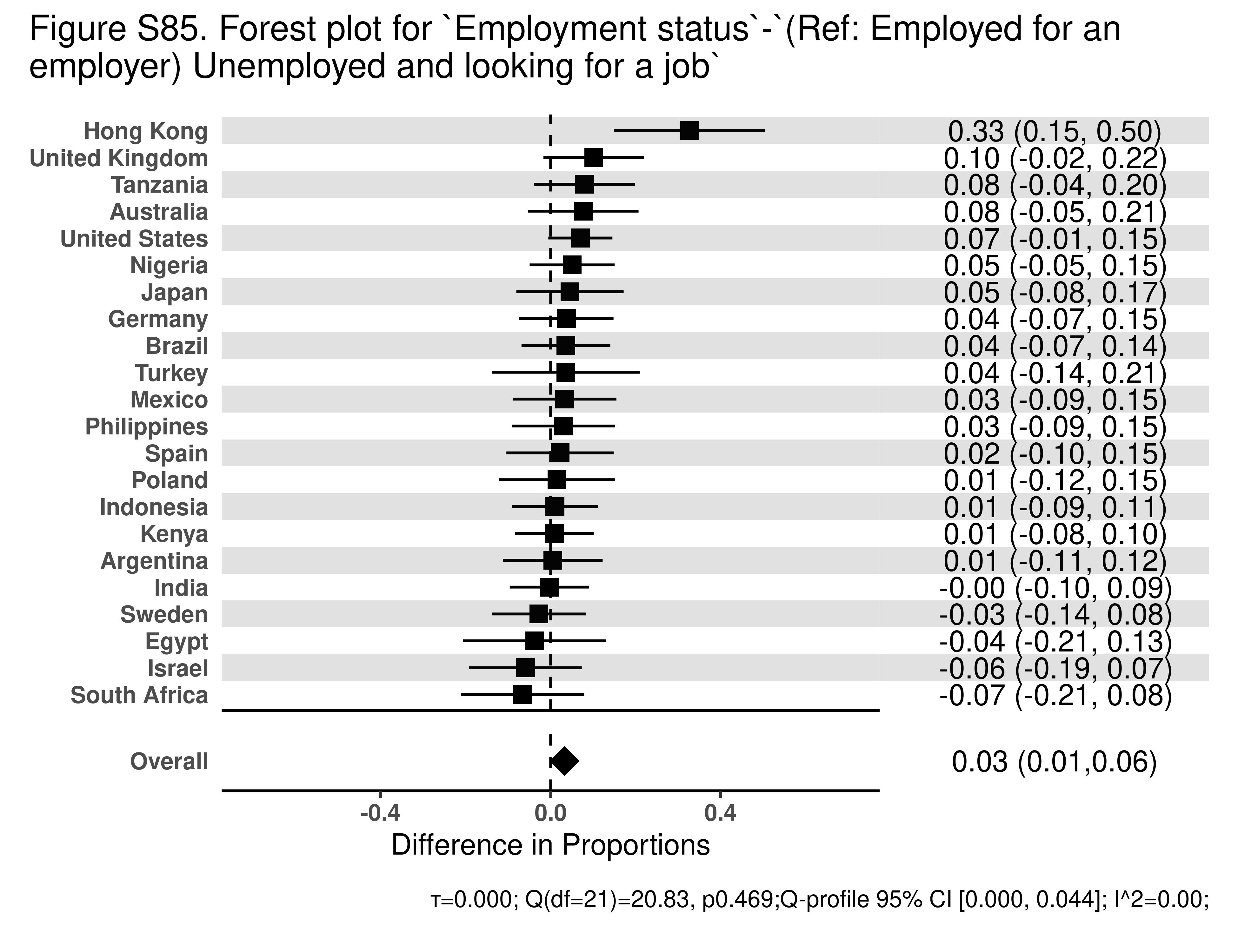

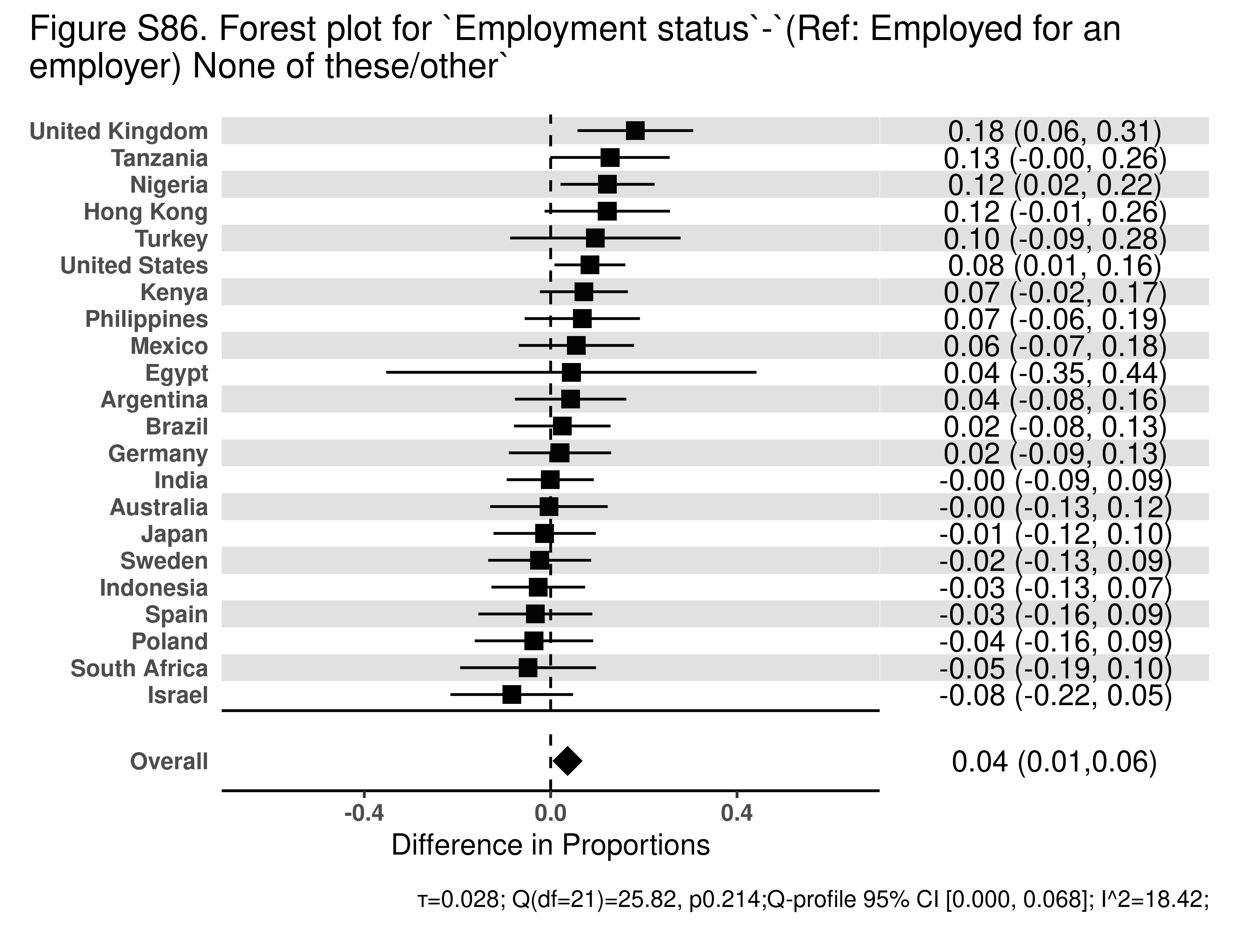

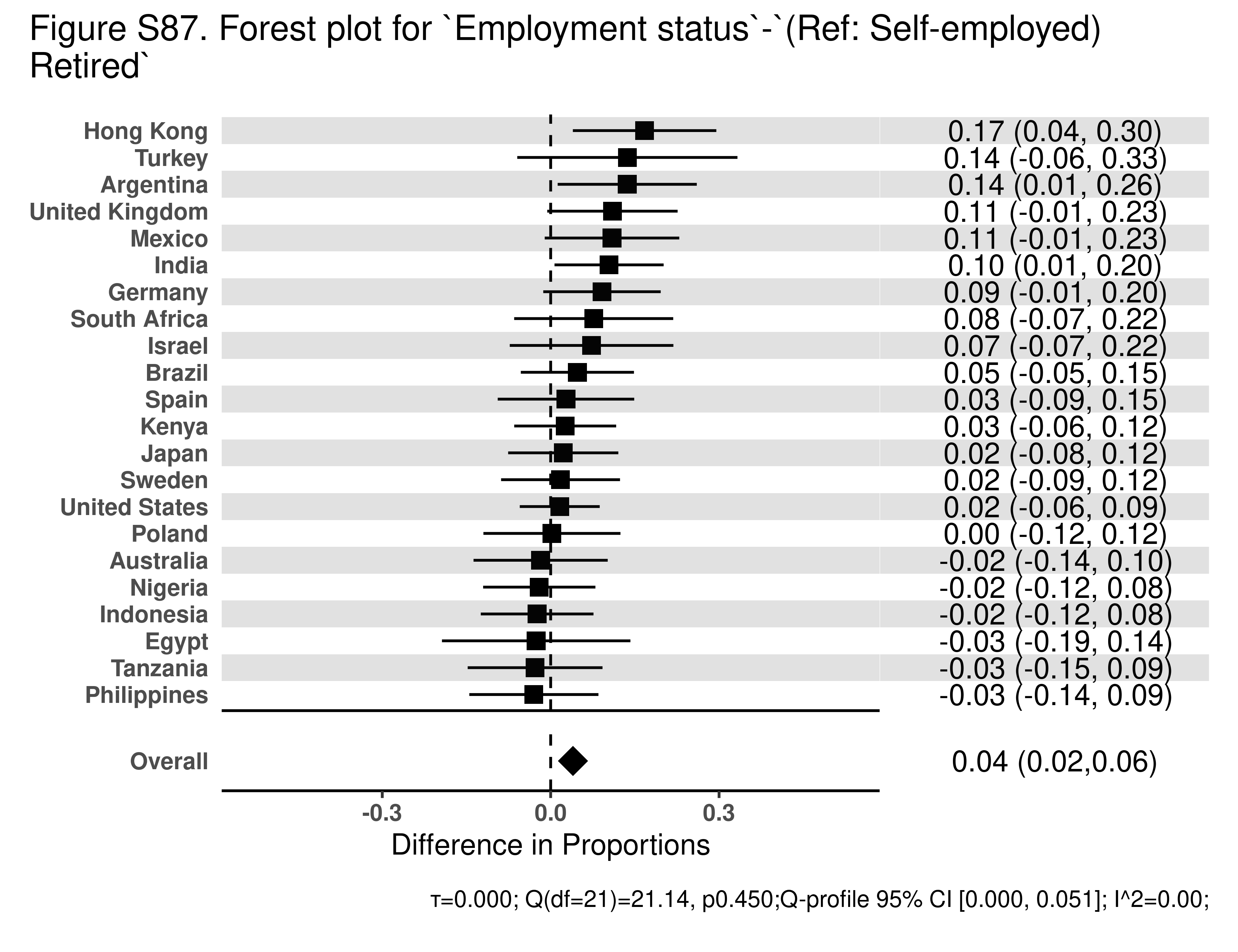

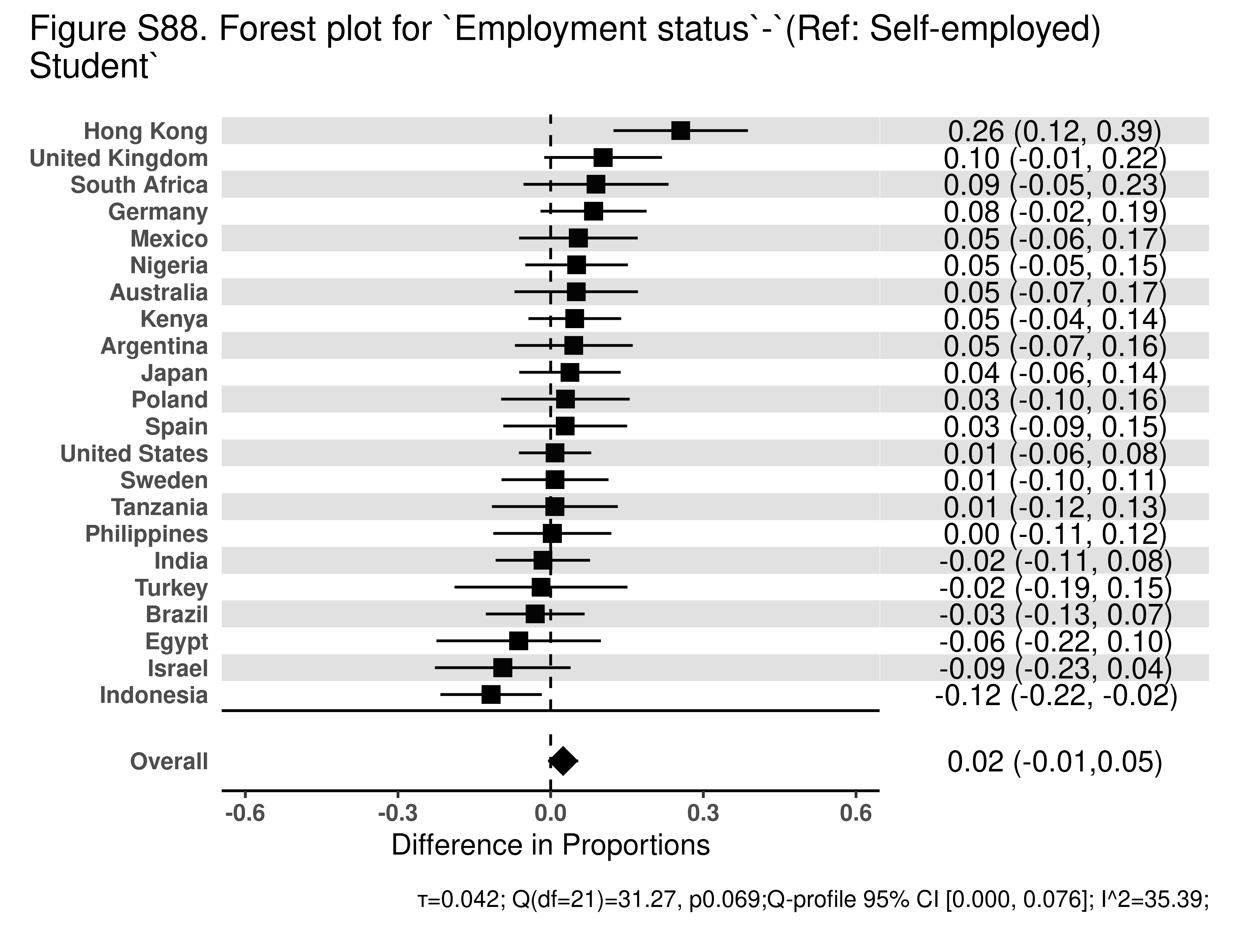

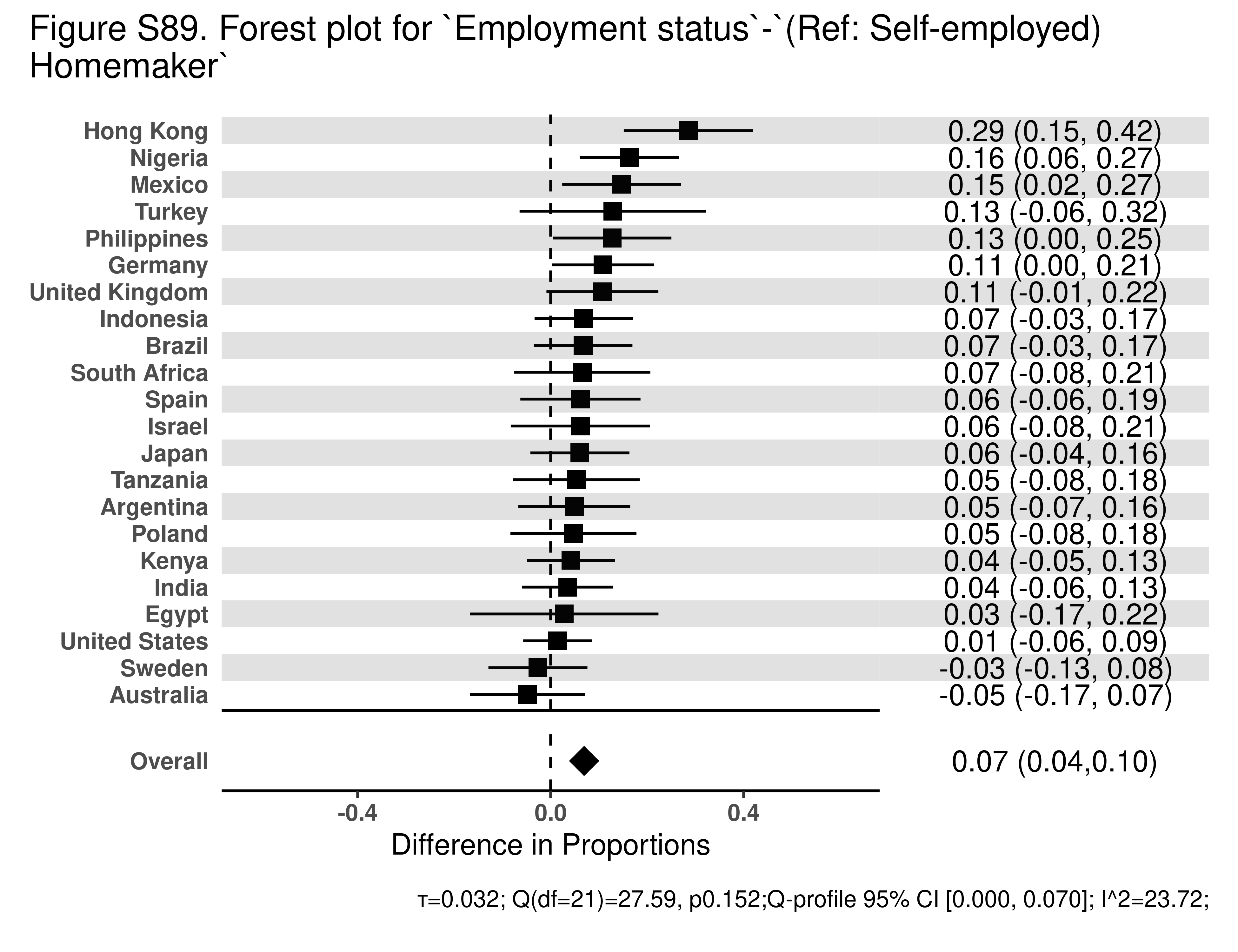

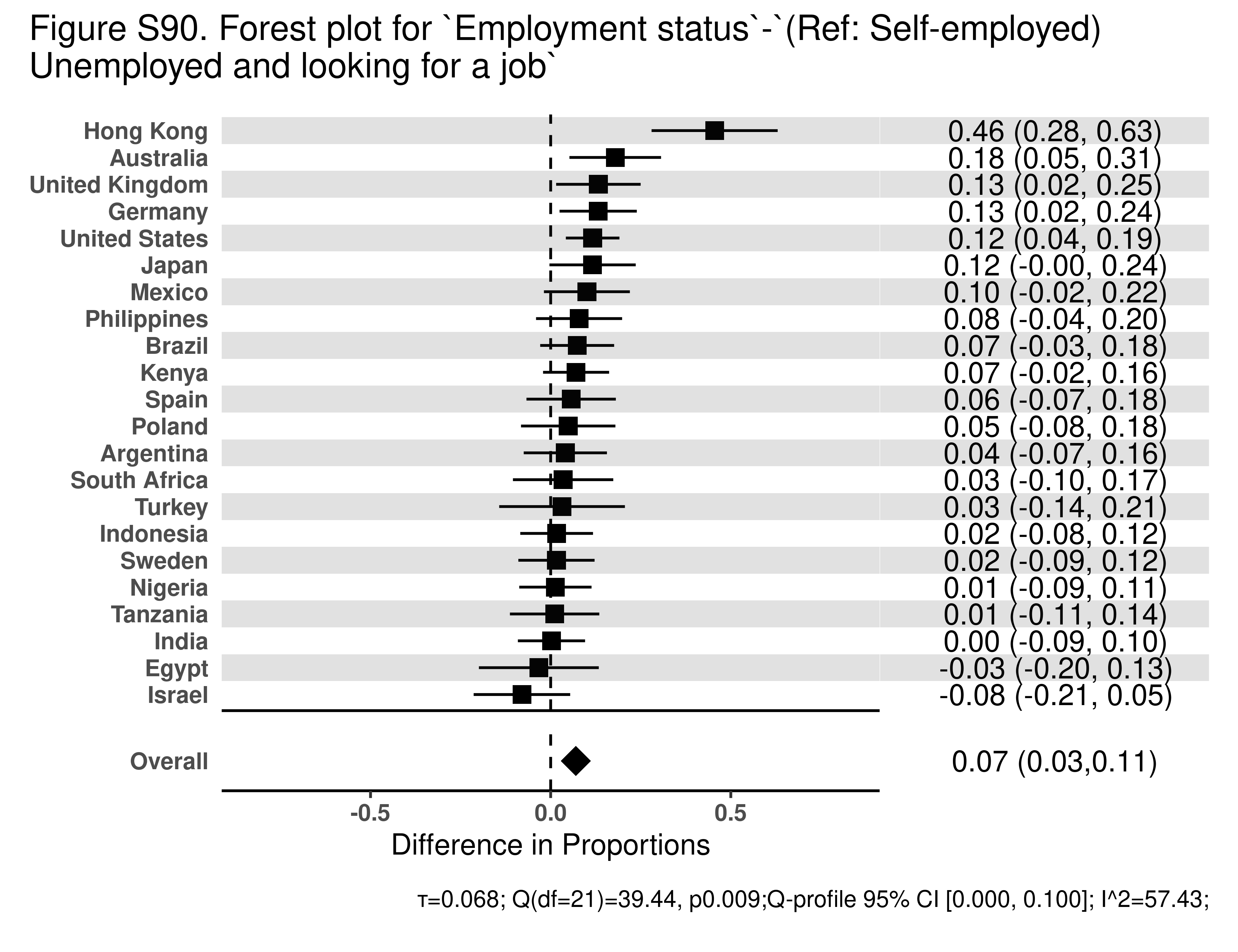

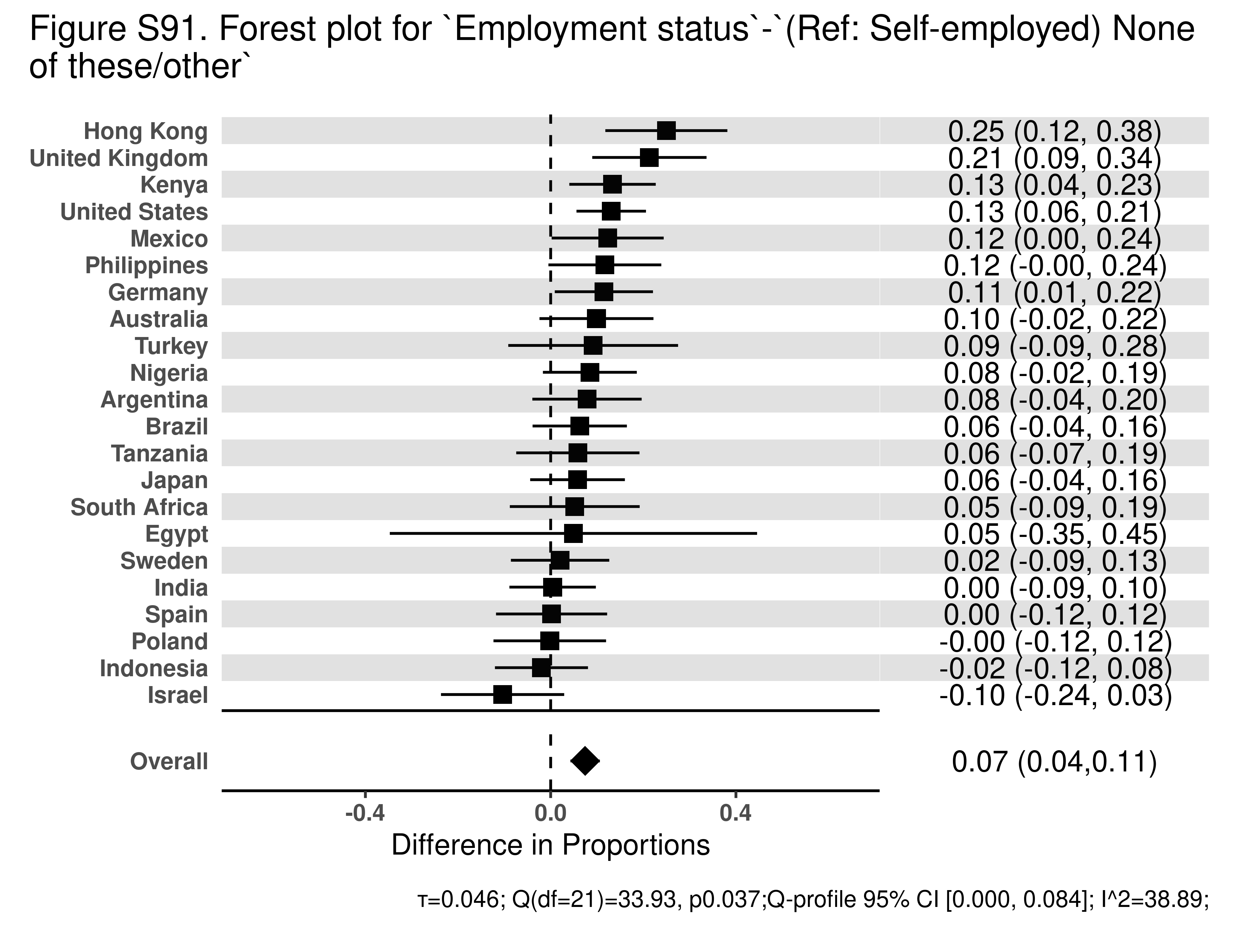

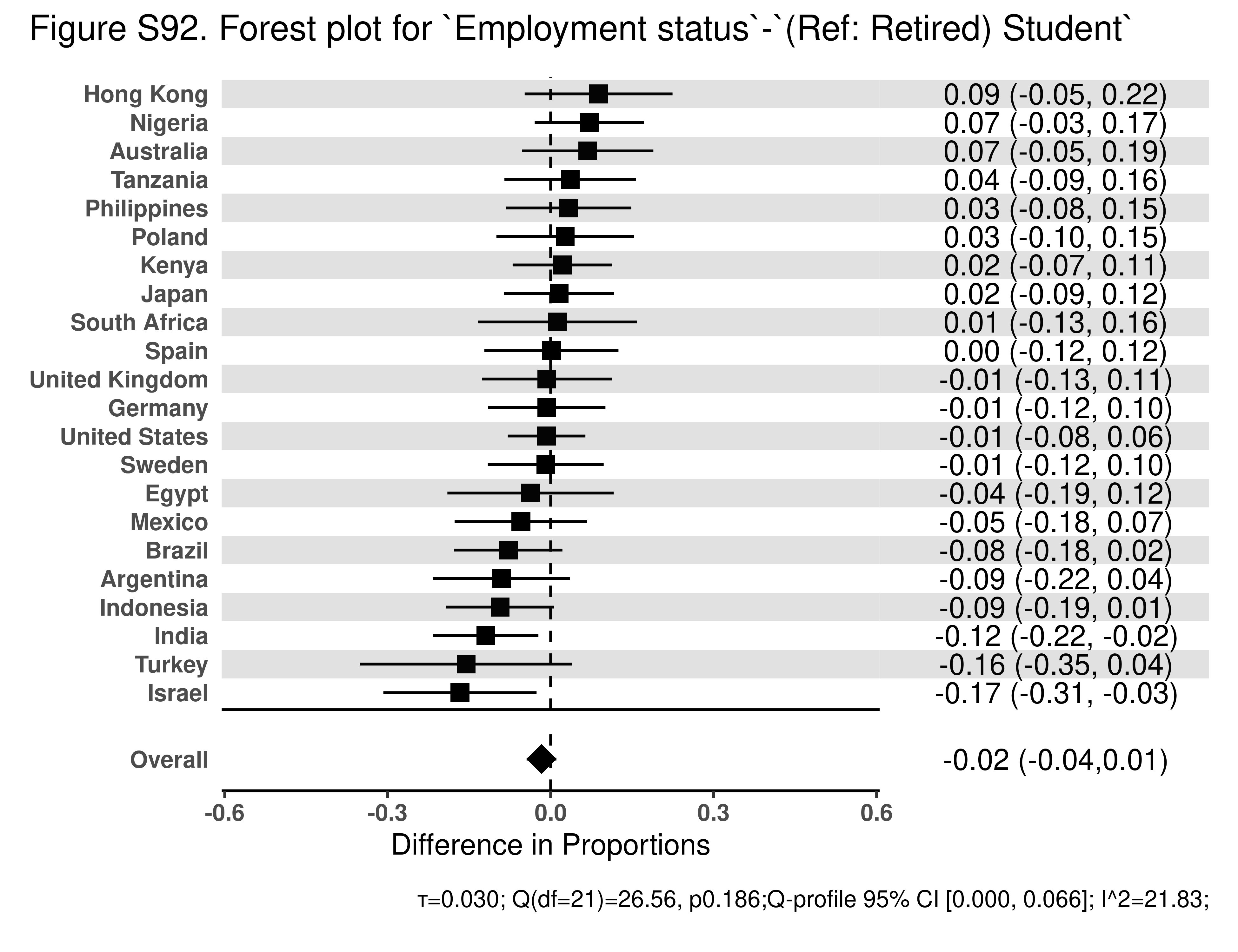

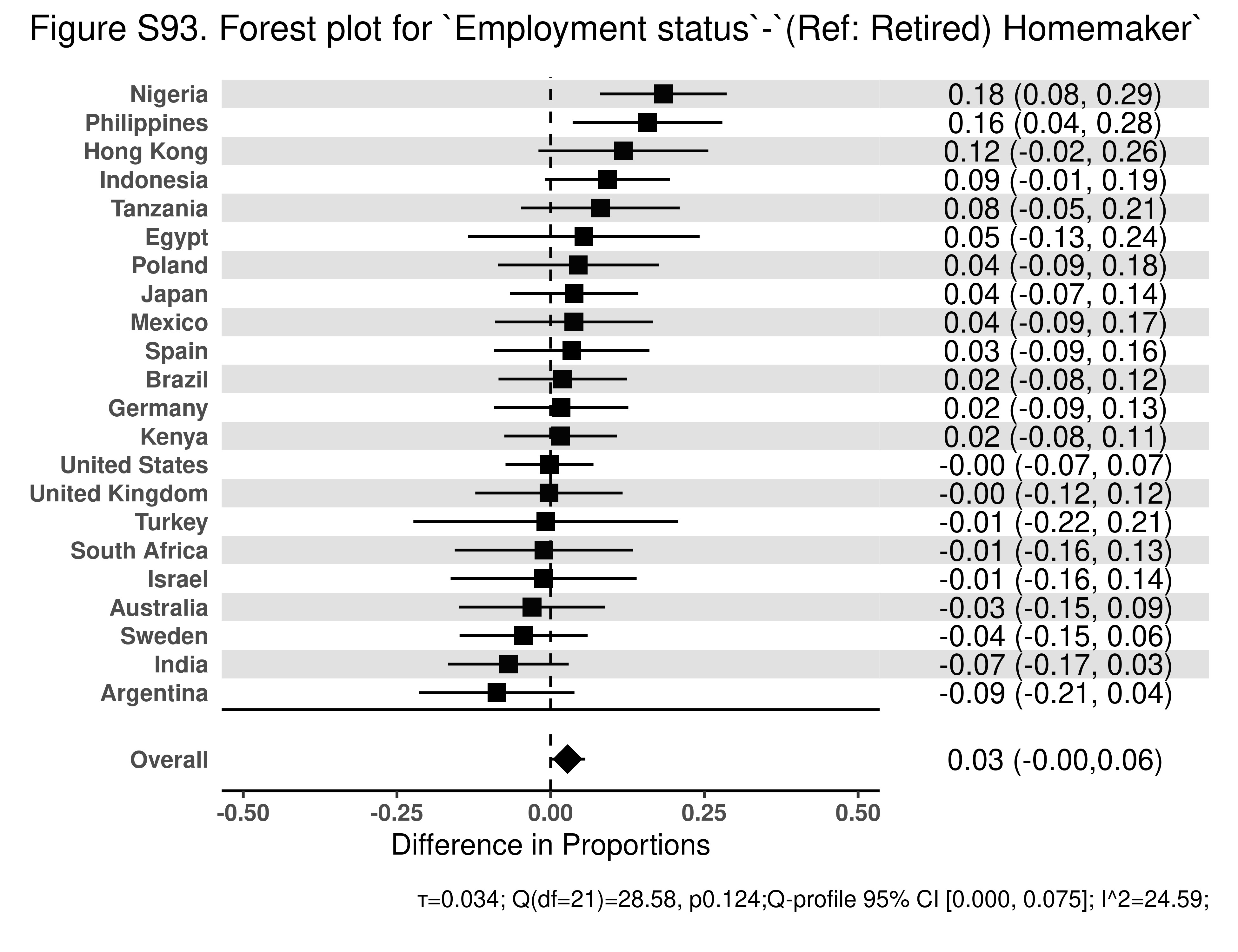

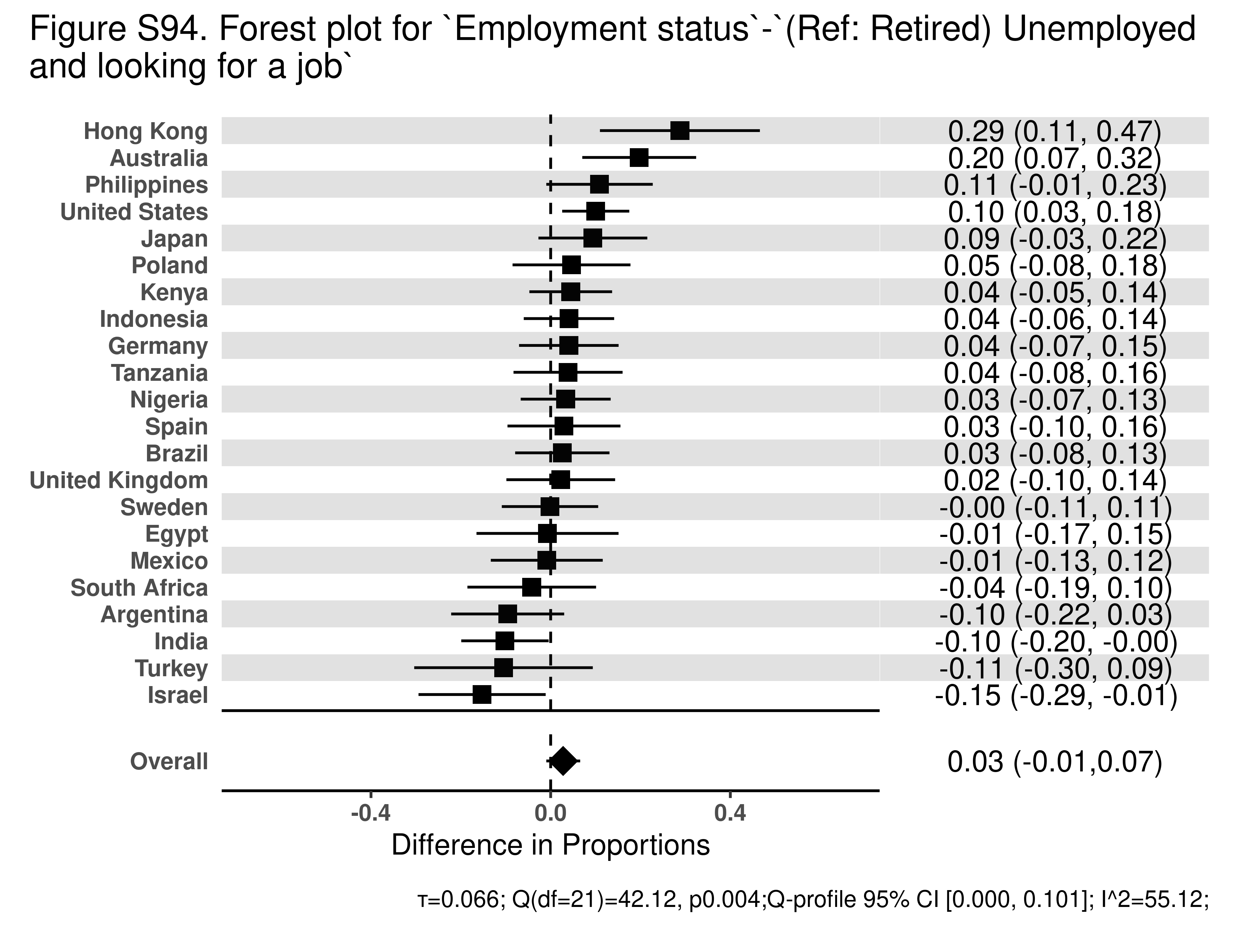

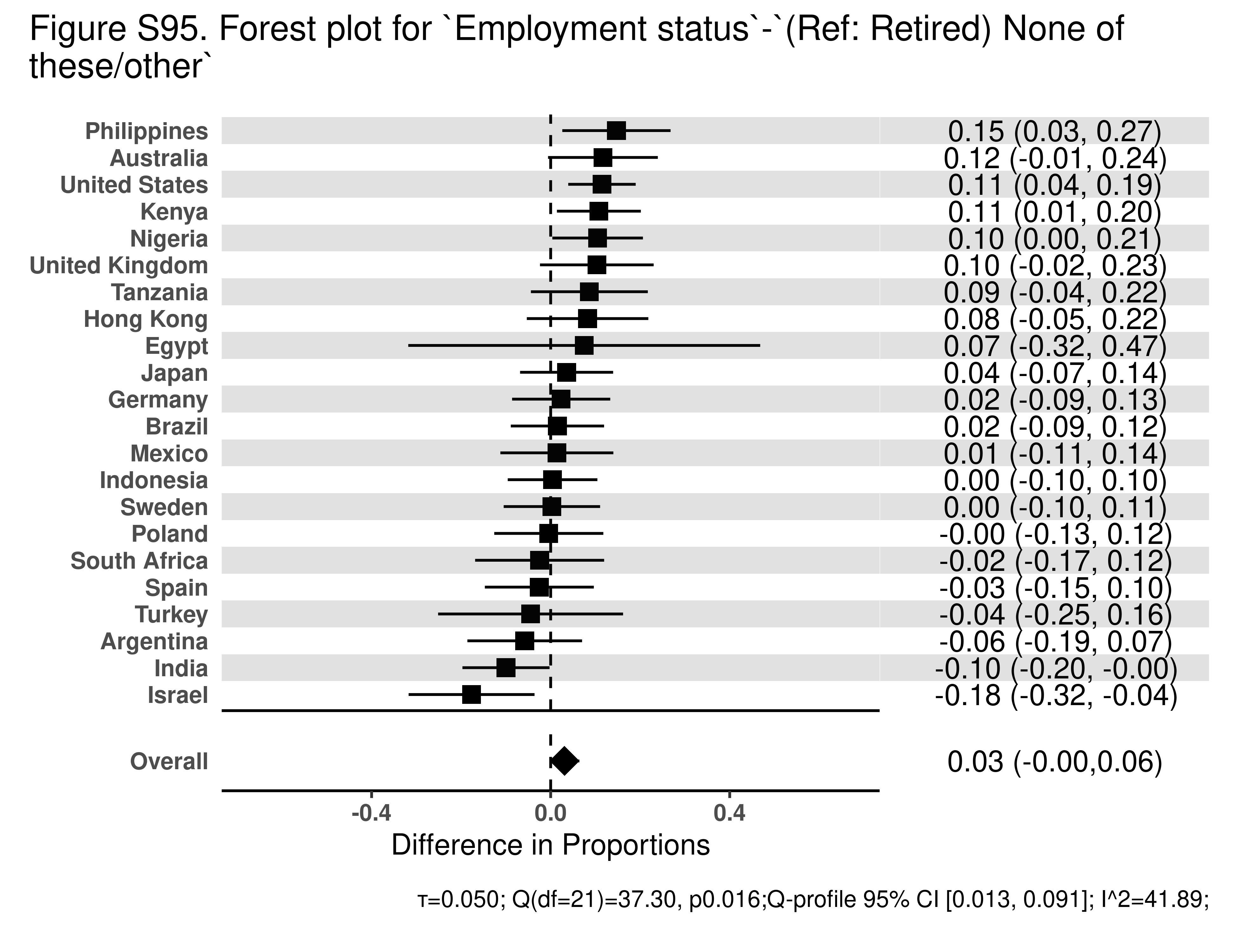

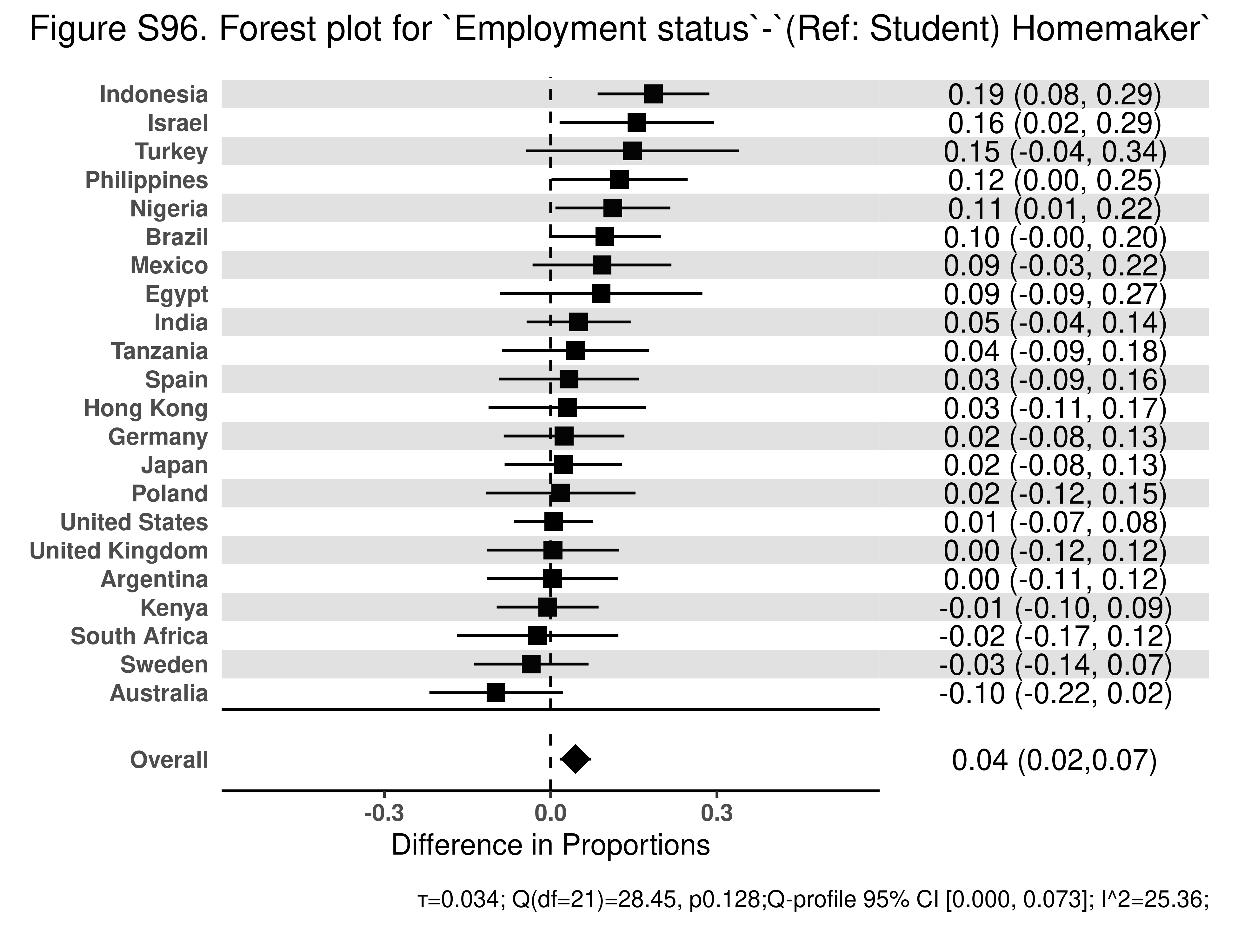

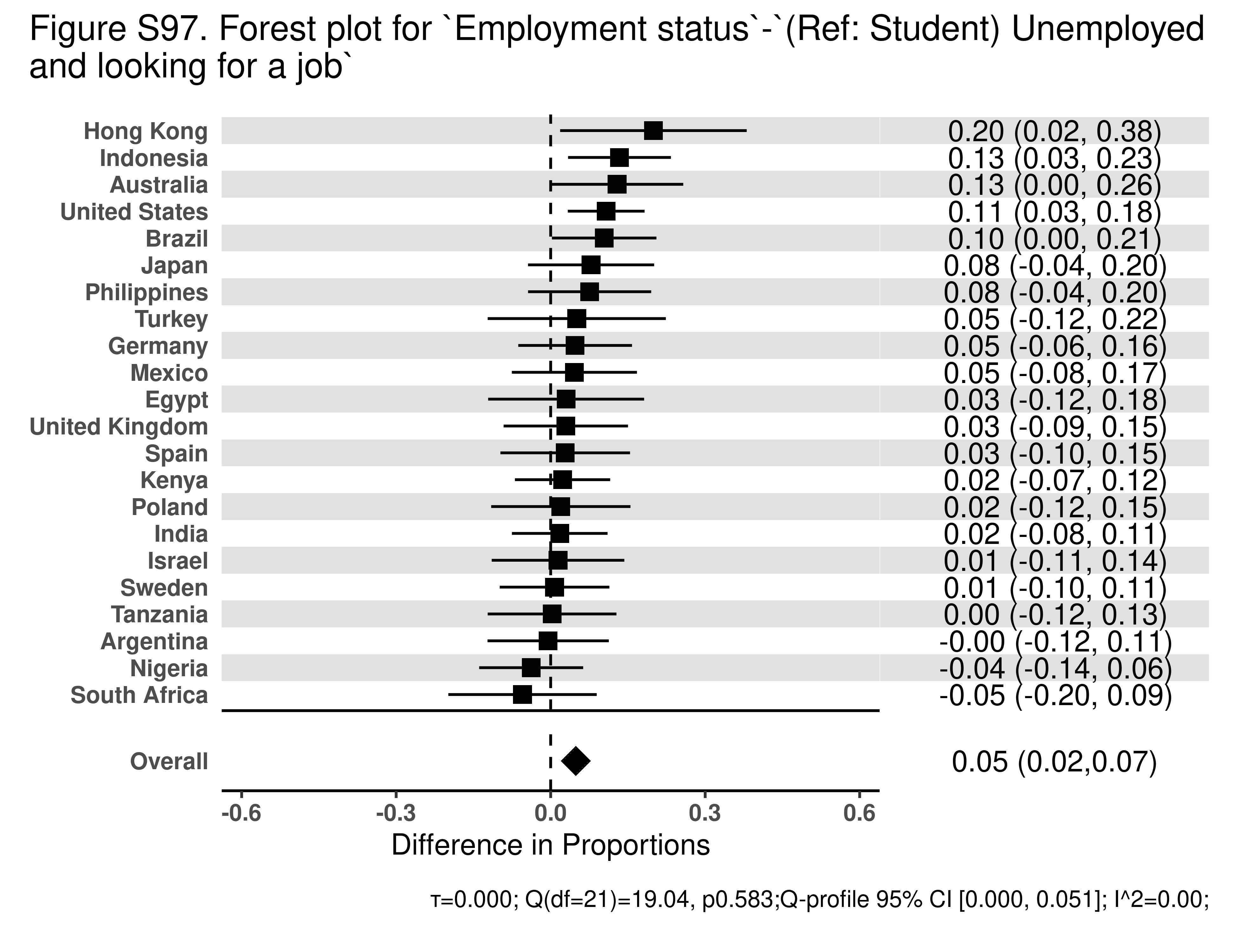

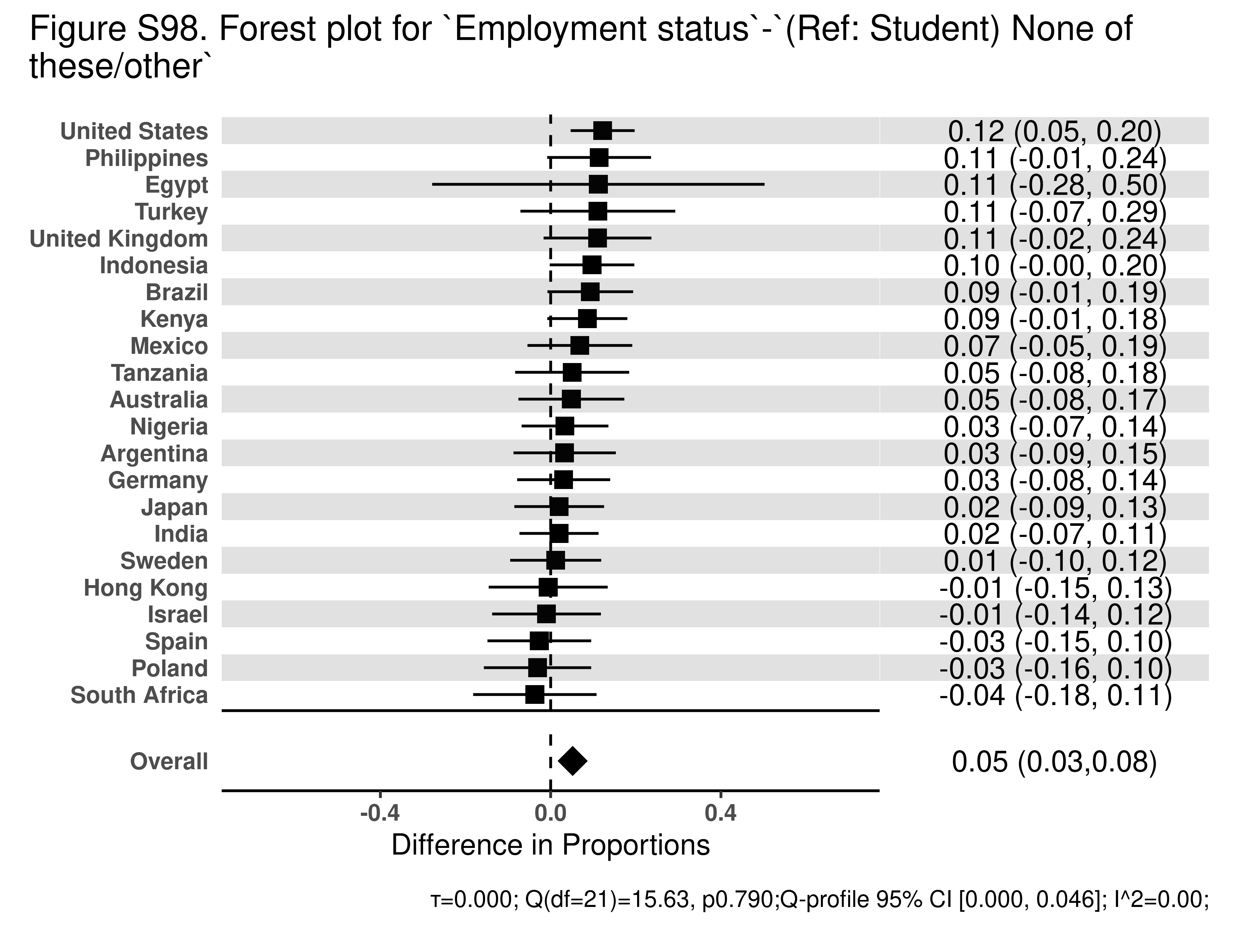

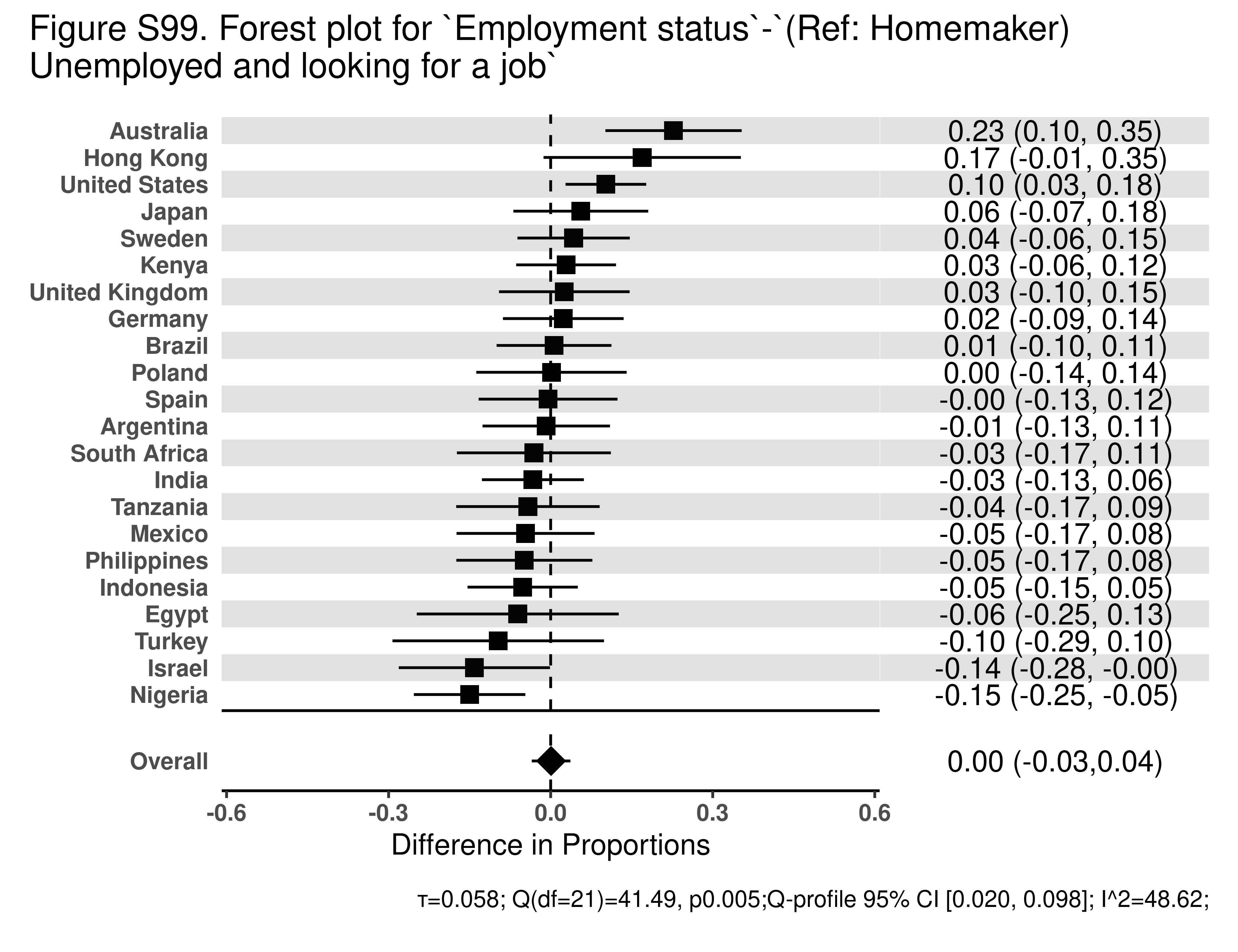

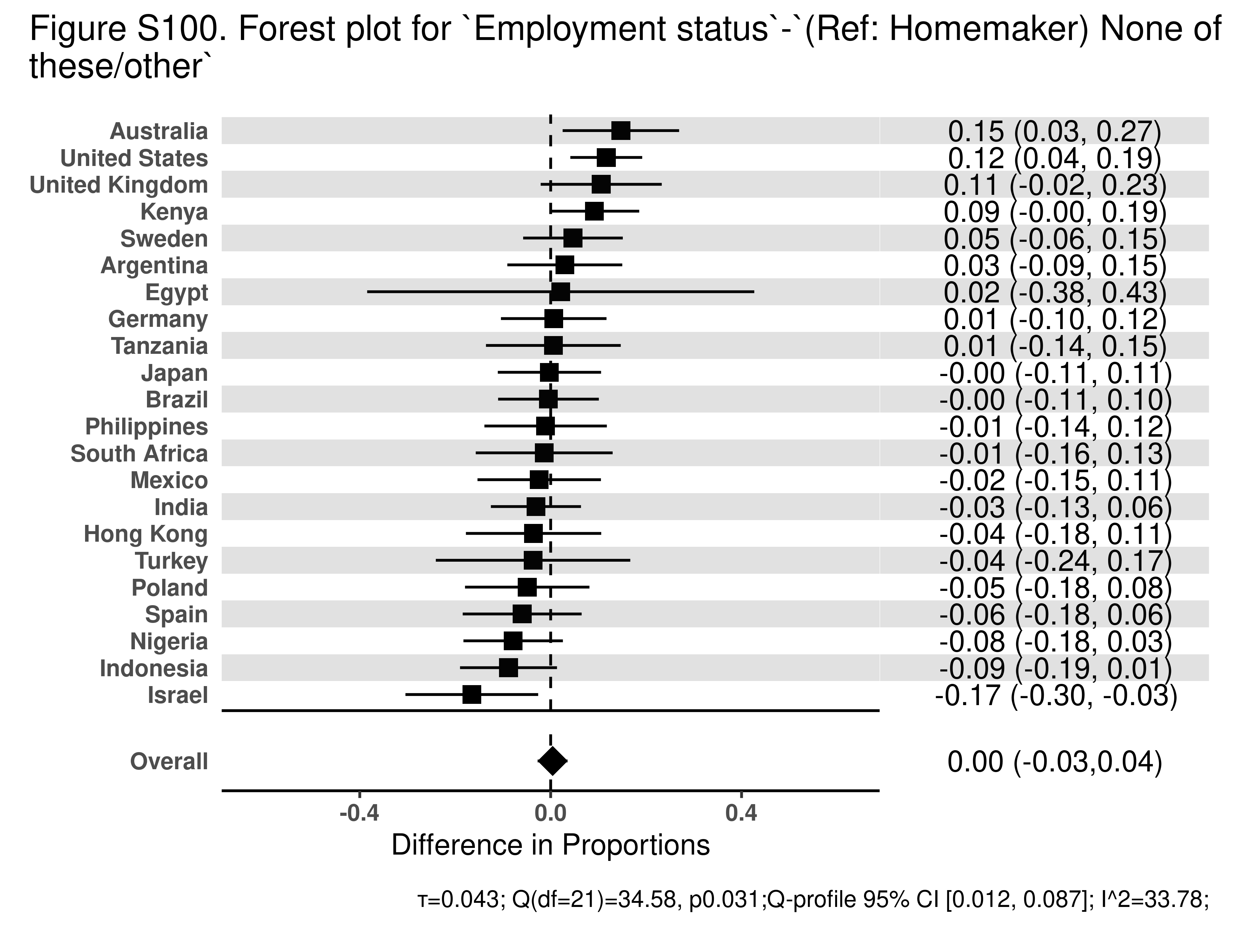

Supplement: Supplementary file 1 — Supplementary Material 1 [file 41598_2025_5459_MOESM1_ESM.docx]
